# Supplementary material for: Kinetic resolution of cyclic benzylic azides enabled by site- and enantioselective C(sp3)–H oxidation
Source: Nat Commun. 2022 Mar 25;13:1621. doi: 10.1038/s41467-022-29319-z (PMC8956603; doi:10.1038/s41467-022-29319-z)
Supplement: Supplementary file 1 — Supplementary Information [file 41467_2022_29319_MOESM1_ESM.pdf]

Supplementary Information

Kinetic Resolution of Cyclic Benzylic Azides Enabled by Site- and Enantioselective C(sp^3)–H Oxidation

Ye et al.

Supplementary Methods

General information

Proton (^{1}H NMR) nuclear magnetic resonance spectra were recorded at 500 MHz (spectral width: 20 ppm; temperature: 23 °C; number of scans: 16), carbon (^{13}C NMR) nuclear magnetic resonance spectra were recorded at 126 MHz (spectral width: 237 ppm; temperature: 23 °C; number of scans: 256), unless otherwise noted in the section of Analytical Data for Products. The chemical shifts are given in parts per million (ppm) on the delta (δ) scale. The solvent peak was used as a reference value, for ^{1}H NMR: $CDCl_3$ δ 7.26; for ^{13}C NMR: $CDCl_3$ at 77.23 ppm. Analytical TLC was performed on precoated silica gel GF254 plates. Column chromatography was carried out on silica gel (200–300 mesh). HRMS were carried out on an Orbitrap analyzer. CD spectra were obtained on a Chirascan spectropolarimeter. Optical rotations were measured using a 2.5 mL cell with a 10 cm path length on Hanon P850 polarimeter and concentrations (c) were reported in g× (100 mL)$^{-1}$. Enantiomeric excesses were determined by HPLC using a Chiralpak AD-H, OD-H, IB, IG or NY (2) 5u column with hexane/*i*-PrOH as the eluent.

General procedure

General procedure A: oxidative kinetic resolution of tetrahydroquinoline-based organic azides.

To a solution of rac-**1a** (0.1 mmol, 1.0 equiv) in ethyl acetate (1.0 mL) was added **C12** (0.005 mmol, 0.05 equiv) at r.t. Then PhIO (0.08 mmol, 0.8 equiv) was added as 8 portions in 30-min intervals over 3.5 h. After that, the solvent was removed under vacuum and the residue was purified by flash chromatography on silica gel using ethyl acetate/petroleum ether as eluent to give the product (*S*)-**1a**.

General procedure B: oxidative kinetic resolution of indoline-based organic azides.

To a solution of rac-**3a** (0.1 mmol, 1.0 equiv) in ethyl acetate (1.0 mL) was added **C7** (0.005 mmol, 0.05 equiv) at r.t. Then PhIO (0.08 mmol, 0.8 equiv) was added as 8

portions in 30-min intervals over 3.5 h. After that, the solvent was removed under vacuum and the residue was purified by flash chromatography on silica gel using ethyl acetate/petroleum ether as eluent to give the product (*S*)-**3a**.

Catalyst synthesis

Mn(salen) **C1-C12** were synthesized following the reported method. [1,2]

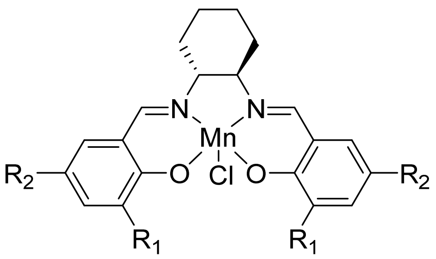

C7: R_1 = OMe, R_2 = H
C12: R_1= 2,4-F_2-C_6H_3, R_2 = H

Mn(salen) **C7**: Brown solid; IR (KBr): 2942, 1620, 1601, 1470, 1444, 1314, 1249, 1222, 1080, 1023, 972, 857, 762, 740, 670, 590 cm^{-1}. HRMS (ESI) *m/z* calculated for $C_{22}H_{24}MnN_2O_4^+$: 435.1117, found 435.1127.

Mn(salen) **C12**: Brown solid; IR (KBr): 2944, 2862, 1614, 1589, 1556, 1506, 1413, 1327, 1224, 1140, 1091, 973, 851, 840, 761, 571 $cm^{-1.}$ HRMS (ESI) *m/z* calculated for $C_{32}H_{24}F_4MnN_2O_2^+$: 599.1149, found 599.1137.

Substrate synthesis

Synthesis of THQ-based organic azides. [3]

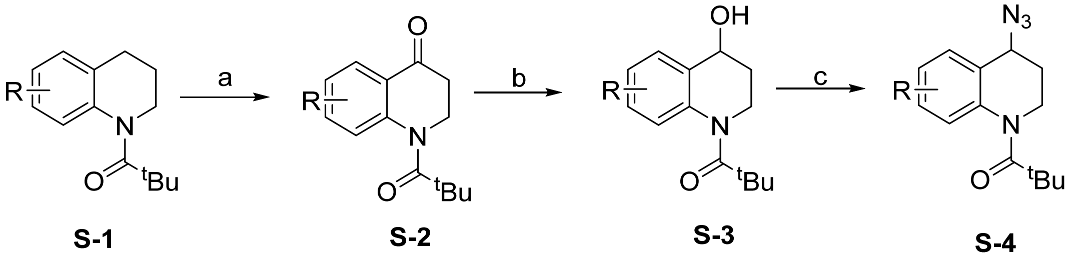

a) To a mixture of **S-1** (20 mmol, 1.0 equiv) and $MgSO_4$ (100 mmol, 5.0 equiv) in CH_3COCH_3/H_2O (40 mL, v/v = 4:1) was added $KMnO_4$ (100 mmol, 5.0 equiv) in

batches at 0 °C. Then the reaction mixture was allowed to warm up to room temperature and stirred until **S-1** was consumed monitored by TLC. The reaction was quenched with saturated $NaHSO_3$ (50 mL) at 0 °C. Then the mixture was filtered with a Buchner funnel, and the filtrate was extracted with ethyl acetate (3 x 30 mL). The organic phases were combined and dried over anhydrous $MgSO_4$. The solvent was removed in vacuo and the residue was purified by silica chromatography to afford **S-2**.

b) To a solution of **S-2** (10 mmol, 1.0 equiv) in dry CH_3OH (50 mL) was added $NaBH_4$ (15 mmol, 1.5 equiv) in batches at 0 °C. The reaction was monitored by TLC. Upon completion, the reaction was quenched with water and extracted with ethyl acetate (3 x 50 mL). The organic phases were combined and dried over $MgSO_4$. The solvent was removed under vacuum and the residue was purified by silica chromatography to afford **S-3**.

c) To a solution of **S-3** (10 mmol, 1.0 equiv) in dry toluene (50 mL) was added DBU (15 mmol, 1.5 equiv) and DPPA (15 mmol, 1.5 equiv) dropwise at 0 °C. Then the reaction mixture was allowed to warm up to room temperature and stirred until **S-3** was consumed monitored by TLC. The reaction was quenched with saturated NH_4Cl (30 mL) at 0 °C and extracted with ethyl acetate (3 x 30 mL). The organic phases were combined and dried over $MgSO_4$. The solvent was removed under vacuum and the residue was purified by silica chromatography to afford **S-4**.

Synthesis of indoline-based organic azides.[4,5]

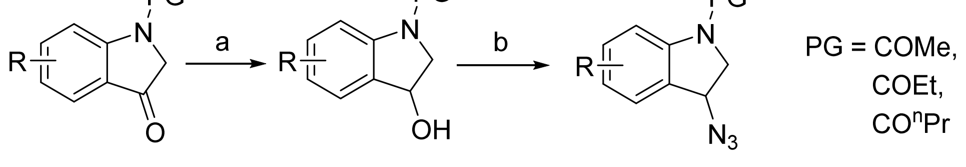

S-5 **S-6** **S-7**

a) To a solution of **S-5** (10 mmol, 1.0 equiv) in dry CH_3OH (50 mL) was added $NaBH_4$ (15 mmol, 1.5 equiv) in batches at 0 °C. The reaction was monitored by TLC. Upon completion, the reaction was quenched with water. The mixture was

concentrated in vacuo and extracted with ethyl acetate (3 x 50 mL). The organic phases were combined and dried over $MgSO_4$. The solvent was removed under vacuum and the residue was purified by silica chromatography to afford **S-6**.

b) To a solution of **S-6** (10 mmol, 1.0 equiv) in dry toluene (50 mL) was added DBU (15 mmol, 1.5 equiv) and DPPA (15 mmol, 1.5 equiv) dropwise at 0 °C. Then the reaction mixture was allowed to warm up to room temperature and stirred until **S-6** was consumed monitored by TLC. The reaction was quenched with saturated NH_4Cl (30 mL) at 0 °C and extracted with ethyl acetate (3 x 30 mL). The organic phases were combined and dried over $MgSO_4$. The solvent was removed under vacuum and the residue was purified by silica chromatography to afford **S-7**.

Analytical Data for Products

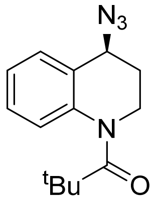

(*S*)-1-(4-Azido-3,4-dihydroquinolin-1(2*H*)-yl)-2,2-dimethylpropan-1-one (1a)

Yield: 48%, 12.4 mg; ^{1}H NMR (500 MHz, $CDCl_3$) δ 7.54 (d, *J* = 8.2 Hz, 1H), 7.38–7.35 (m, 1H), 7.32–7.28 (m, 1H), 7.21–7.17 (m, 1H), 4.63 (t, *J* = 4.9 Hz, 1H), 4.13–4.06 (m, 1H), 3.73–3.64 (m, 1H), 2.25–2.18 (m, 1H), 2.17–2.10 (m, 1H), 1.37 (s, 9H); ^{13}C NMR (126 MHz, $CDCl_3$) δ 178.8, 139.9, 128.7, 128.3, 127.6, 126.1, 125.2, 57.0, 42.6, 40.6, 30.6, 29.1; HPLC: the ee value was determined by HPLC analysis (Chiralcel OD-H, *i*-PrOH/Hexane = 20/80, 1.0 mL/min, 252 nm), retention time: t_{minor} =5.577 min, t_{major} = 5.937 min, ee = 98%; $[\alpha]_D^{25}$ = – 17.50 (c = 0.26, $CHCl_3$); HRMS m/z $[M + H]^+$ calculated for $C_{14}H_{19}N_4O$: 259.1553, found 259.1558.

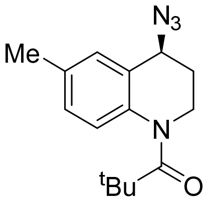

(*S*)-1-(4-Azido-6-methyl-3,4-dihydroquinolin-1(2*H*)-yl)-2,2-dimethylpropan-1-one (1b)

Yield: 50%, 13.6 mg; ^{1}H NMR (500 MHz, $CDCl_3$) δ 7.41 (d, *J* = 8.3 Hz, 1H), 7.15 (s, 1H), 7.10 (d, *J* = 8.3 Hz, 1H), 4.57 (t, *J* = 5.0 Hz, 1H), 4.10–4.04 (m, 1H), 3.70–3.62 (m, 1H), 2.36 (s, 3H), 2.25–2.17 (m, 1H), 2.14–2.07 (m, 1H), 1.35 (s, 9H); ^{13}C NMR (126 MHz, $CDCl_3$) δ 178.4, 137.3, 134.8, 129.0, 128.8, 127.5, 125.9, 57.0, 42.5, 40.5, 30.6, 29.0, 21.0; HPLC: the ee value was determined by HPLC analysis (Chiralcel IG, *i*-PrOH/Hexane = 20/80, 1.0 mL/min, 254 nm), retention time: t_{minor} = 8.003 min, t_{major} = 6.427 min, ee = 92%; $[\alpha]_D^{25}$ = – 15.68 (c = 0.60, $CHCl_3$); HRMS m/z $[M + H]^+$ calculated for $C_{15}H_{21}N_4O$: 273.1710, found 273.1718.

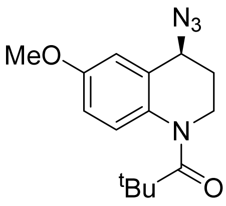

(*S*)-1-(4-Azido-6-methoxy-3,4-dihydroquinolin-1(2*H*)-yl)-2,2-dimethylpropan-1-one (1c)

Yield: 50%, 14.4 mg; ^{1}H NMR (500 MHz, $CDCl_3$) δ 7.42 (d, *J* = 8.7 Hz, 1H), 6.86–6.82 (m, 2H), 4.54 (t, *J* = 5.3 Hz, 1H), 4.11–4.06 (m, 1H), 3.81 (s, 3H), 3.65–3.59 (m, 1H), 2.26–2.20 (m, 1H), 2.11–2.05 (m, 1H), 1.32 (s, 9H); ^{13}C NMR (126 MHz, $CDCl_3$) δ 178.3, 157.0, 133.0, 129.3, 127.4, 114.4, 112.5, 57.1, 55.8, 42.7, 40.4, 30.8, 29.1; HPLC: the ee value was determined by HPLC analysis (Chiralcel IG, *i*-PrOH/Hexane = 20/80, 1.0 mL/min, 252 nm), retention time: t_{minor} = 13.367 min, t_{major} = 9.993 min, ee = 90%; $[\alpha]_D^{25}$ = – 20.53 (c = 0.55, $CHCl_3$); HRMS m/z $[M + H]^+$ calculated for $C_{15}H_{21}N_4O_2$: 289.1659, found 289.1663.

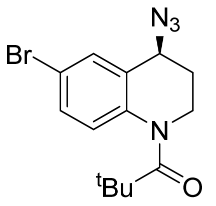

(*S*)-1-(4-Azido-6-bromo-3,4-dihydroquinolin-1(2*H*)-yl)-2,2-dimethylpropan-1-one (1d)

Yield: 49%, 16.5 mg; ^{1}H NMR (500 MHz, $CDCl_3$) δ 7.47 (d, *J* = 2.1 Hz, 1H), 7.42 (d, *J* = 8.8 Hz, 1H), 7.36 (dd, *J* = 8.8, 2.2 Hz, 1H), 4.55 (t, *J* = 5.0 Hz, 1H), 4.11–4.05 (m, 1H), 3.67–3.60 (m, 1H), 2.23–2.15 (m, 1H), 2.15–2.08 (m, 1H), 1.35 (s, 9H); ^{13}C NMR (126 MHz, $CDCl_3$) δ 178.5, 138.8, 131.4, 131.2, 129.4, 127.8, 117.8, 56.3, 42.4, 40.5, 30.3, 28.9; HPLC: the ee value was determined by HPLC analysis (Chiralcel IG, *i*-PrOH/Hexane = 20/80, 1.0 mL/min, 277 nm), retention time: t_{minor} = 9.600 min, t_{major} = 7.077 min, ee = 91%; $[\alpha]_D^{25}$ = – 14.30 (c = 0.48, $CHCl_3$); HRMS m/z $[M + H]^+$ calculated for $C_{14}H_{18}{}^{79}BrN_4O$: 337.0659, found 337.0655; calculated for $C_{14}H_{18}{}^{81}BrN_4O$: 339.0639, found 339.0643.

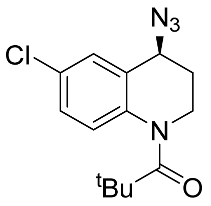

(*S*)-1-(4-Azido-6-chloro-3,4-dihydroquinolin-1(2*H*)-yl)-2,2-dimethylpropan-1-one (1e)

Yield: 48%, 14.1 mg; ^{1}H NMR (500 MHz, $CDCl_3$) δ 7.47 (d, *J* = 8.8 Hz, 1H), 7.32 (d, *J* = 2.4 Hz, 1H), 7.22 (dd, *J* = 8.8, 2.5 Hz, 1H), 4.55 (t, *J* = 5.1 Hz, 1H), 4.11–4.05 (m, 1H), 3.66–3.60 (m, 1H), 2.23–2.16 (m, 1H), 2.15–2.08 (m, 1H), 1.34 (s, 9H); ^{13}C NMR (126 MHz, $CDCl_3$) δ 178.5, 138.3, 130.1, 129.1, 128.4, 128.3, 127.5, 56.4, 42.5, 40.5, 30.3, 28.9; HPLC: the ee value was determined by HPLC analysis (Chiralcel IG, *i*-PrOH/Hexane = 20/80, 1.0 mL/min, 277 nm), retention time: t_{minor} = 7.537 min, t_{major} = 5.957 min, ee = 94%; $[\alpha]_D^{25}$ = –15.66 (c = 0.45, $CHCl_3$); HRMS m/z $[M + H]^+$ calculated for $C_{14}H_{18}ClN_4O$: 293.1164, found 293.1168.

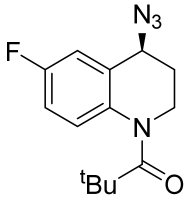

(*S*)-1-(4-Azido-6-fluoro-3,4-dihydroquinolin-1(2*H*)-yl)-2,2-dimethylpropan-1-one (1f)

Yield: 49%, 13.5 mg; ^{1}H NMR (500 MHz, $CDCl_3$) δ 7.52–7.47 (m, 1H), 7.08–7.03 (m, 1H), 7.01–6.95 (m, 1H), 4.55 (t, J = 5.2 Hz, 1H), 4.14–4.07 (m, 1H), 3.66–3.60 (m, 1H), 2.27–2.20 (m, 1H), 2.15–2.08 (m, 1H), 1.34 (s, 9H); ^{13}C NMR (126 MHz, $CDCl_3$) δ 178.4, 160.6, 158.7, 135.8, 135.8, 129.7, 129.6, 128.1, 128.0, 115.5, 115.3, 114.7, 114.6, 56.6, 42.6, 40.4, 30.5, 29.0; ^{19}F NMR (471 MHz, $CDCl_3$) δ -116.46; HPLC: the ee value was determined by HPLC analysis (Chiralcel IG, *i*-PrOH/Hexane = 20/80, 1.0 mL/min, 210 nm), retention time: t_{minor} = 6.827 min, t_{major} = 5.903 min, ee = 94%; $[\alpha]_D^{25}$ = –16.45 (c = 0.56, $CHCl_3$); HRMS m/z $[M + H]^+$ calculated for $C_{14}H_{18}FN_4O$: 277.1459, found 277.1455.

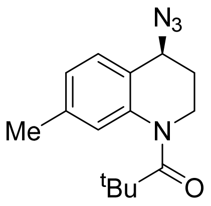

(*S*)-1-(4-Azido-7-methyl-3,4-dihydroquinolin-1(2H)-yl)-2,2-dimethylpropan-1-one (1g)

Yield: 48%, 13.1 mg; ^{1}H NMR (500 MHz, $CDCl_3$) δ 7.35 (s, 1H), 7.21 (d, *J* = 7.8 Hz, 1H), 6.98 (d, *J* = 7.8 Hz, 1H), 4.57 (t, *J* = 4.8 Hz, 1H), 4.08–4.01 (m, 1H), 3.67–3.61 (m, 1H), 2.33 (s, 3H), 2.19–2.12 (m, 1H), 2.12–2.06 (m, 1H), 1.35 (s, 9H); ^{13}C NMR (126 MHz, $CDCl_3$) δ 178.7, 139.7, 138.3, 128.6, 126.4, 126.1, 124.5, 56.9, 42.6, 40.6, 30.8, 29.1, 21.5; HPLC: the ee value was determined by HPLC analysis (Chiralcel OD-H, *i*-PrOH/Hexane = 10/90, 1.0 mL/min, 217 nm), retention time: t_{minor} = 5.527 min, t_{major} = 6.020 min, ee = 92%; $[\alpha]_D^{25}$ = – 17.20 (c = 0.49, $CHCl_3$); HRMS m/z $[M + H]^+$ calculated for $C_{15}H_{21}N_4O$: 273.1710, found 273.1716.

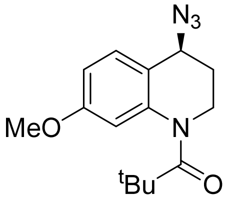

(*S*)-1-(4-Azido-7-methoxy-3,4-dihydroquinolin-1(2*H*)-yl)-2,2-dimethylpropan-1-one (1h)

Yield: 48%, 13.8 mg; ^{1}H NMR (500 MHz, $CDCl_3$) δ 7.24–7.21 (m, 1H), 7.13 (d, *J* = 2.6 Hz, 1H), 6.74 (dd, *J* = 8.5, 2.6 Hz, 1H), 4.60–4.55 (m, 1H), 4.07–4.01 (m, 1H), 3.79 (s, 3H), 3.69–3.62 (m, 1H), 2.18–2.07 (m, 2H), 1.36 (s, 9H); ^{13}C NMR (126 MHz, $CDCl_3$) δ 178.6, 159.4, 141.0, 130.0, 119.6, 111.9, 110.7, 56.8, 55.6, 42.6, 40.7, 30.6, 29.0; HPLC: the ee value was determined by HPLC analysis (Chiralcel AD-H, *i*-PrOH/Hexane = 20/80, 1.0 mL/min, 222 nm), retention time: t_{minor} = 5.340 min, t_{major} = 6.257 min, ee = 91%; $[\alpha]_D^{25}$ = – 14.35 (c = 0.48, $CHCl_3$); HRMS m/z $[M + H]^+$ calculated for $C_{15}H_{21}N_4O_2$: 289.1659, found 289.1655.

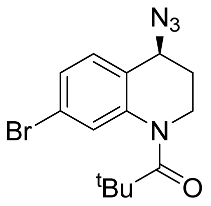

(*S*)-1-(4-Azido-7-bromo-3,4-dihydroquinolin-1(2*H*)-yl)-2,2-dimethylpropan-1-one (1i)

Yield: 49%, 16.5 mg; ^{1}H NMR (500 MHz, $CDCl_3$) δ 7.76 (d, *J* = 1.9 Hz, 1H), 7.28 (dd, *J* = 8.3, 1.9 Hz, 1H), 7.20 (d, *J* = 8.3 Hz, 1H), 4.56 (t, *J* = 4.8 Hz, 1H), 4.12–4.06 (m, 1H), 3.66–3.60 (m, 1H), 2.18–2.10 (m, 2H), 1.37 (s, 9H); ^{13}C NMR (126 MHz, $CDCl_3$) δ 178.6, 140.9, 130.2, 128.9, 128.0, 126.0, 121.9, 56.4, 42.4, 40.6, 30.3, 28.9; HPLC: the ee value was determined by HPLC analysis (Chiralcel IB, *i*-PrOH/Hexane = 20/80, 1.0 mL/min, 233 nm), retention time: t_{minor} = 4.907 min, t_{major} = 5.373 min, ee = 92%; $[\alpha]_D^{25}$ = –19.30 (c = 0.32, $CHCl_3$); HRMS m/z $[M + H]^+$ calculated for $C_{14}H_{18}{}^{79}BrN_4O$: 337.0659, found 337.0651; calculated for $C_{14}H_{18}{}^{81}BrN_4O$: 339.0639, found 339.0647.

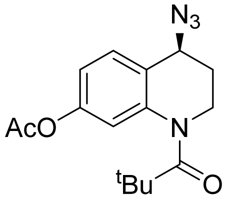

(*S*)-4-Azido-1-pivaloyl-1,2,3,4-tetrahydroquinolin-7-yl acetate (1j)

Yield: 47%, 14.9 mg; ^{1}H NMR (500 MHz, $CDCl_3$) δ 7.41 (d, *J* = 2.3 Hz, 1H), 7.35–7.32 (m, 1H), 6.92 (dd, *J* = 8.4, 2.3 Hz, 1H), 4.61 (t, *J* = 4.8 Hz, 1H), 4.11–4.06 (m, 1H), 3.72–3.65 (m, 1H), 2.27 (s, 3H), 2.19–2.10 (m, 2H), 1.36 (s, 9H); ^{13}C NMR (126 MHz, $CDCl_3$) δ 178.5, 169.5, 150.2, 140.6, 129.7, 124.7, 119.2, 118.4, 56.6, 42.5, 40.6, 30.3, 28.9, 21.3; HPLC: the ee value was determined by HPLC analysis (Chiralcel AD-H, *i*-PrOH/Hexane = 20/80, 1.0 mL/min, 254 nm), retention time: t_{minor} = 5.843 min, t_{major} = 8.930 min, ee = 94%; $[\alpha]_D^{25}$ = –12.13 (c = 0.40, $CHCl_3$); HRMS m/z $[M + H]^+$ calculated for $C_{16}H_{21}N_4O_3$: 317.1608, found 317.1612.

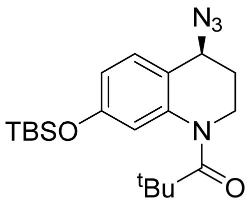

(*S*)-1-(4-Azido-7-((tert-butyldimethylsilyl)oxy)-3,4-dihydroquinolin-1(2*H*)-yl)-2,2-dimethylpropan-1-one (1k)

Yield: 49%, 19.0 mg; ^{1}H NMR (500 MHz, $CDCl_3$) δ 7.17 (d, *J* = 8.4 Hz, 1H), 7.03 (d, *J* = 2.4 Hz, 1H), 6.66 (dd, *J* = 8.4, 2.4 Hz, 1H), 4.59–4.54 (m, 1H), 4.05–3.99 (m, 1H), 3.67–3.61 (m, 1H), 2.16–2.07 (m, 2H), 1.34 (s, 9H), 0.98 (s, 9H), 0.21 (s, 3H), 0.21 (s, 3H); ^{13}C NMR (126 MHz, $CDCl_3$) δ 178.7, 155.6, 140.9, 129.6, 120.4, 117.3, 117.2, 56.9, 42.6, 40.7, 30.6, 29.1, 25.9, 18.4, -4.1, -4.2; HPLC: the ee value was determined by HPLC analysis (Chiralcel IG, *i*-PrOH/Hexane = 3/97, 1.0 mL/min, 219 nm), retention time: t_{minor} = 6.153 min, t_{major} = 6.860 min, ee = 95%; $[\alpha]_D^{25}$ = –18.00 (c = 1.10, $CHCl_3$); HRMS m/z $[M + H]^+$ calculated for $C_{20}H_{33}N_4O_2Si$: 389.2367, found 389.2357.

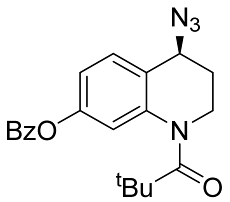

(*S*)-4-Azido-1-pivaloyl-1,2,3,4-tetrahydroquinolin-7-yl benzoate (1l)

Yield: 47%, 17.8 mg; ^{1}H NMR (500 MHz, $CDCl_3$) δ 8.23–8.14 (m, 2H), 7.66–7.60 (m, 1H), 7.56–7.47 (m, 3H), 7.39 (d, *J* = 8.4 Hz, 1H), 7.06 (dd, *J* = 8.4, 2.3 Hz, 1H), 4.65 (t, *J* = 4.8 Hz, 1H), 4.13–4.08 (m, 1H), 3.75–3.68 (m, 1H), 2.23–2.12 (m, 2H), 1.37 (s, 9H); ^{13}C NMR (126 MHz, $CDCl_3$) δ 178.5, 165.2, 150.5, 140.7, 133.9, 130.4, 129.7, 129.5, 128.8, 124.8, 119.3, 118.6, 56.6, 42.5, 40.6, 30.3, 28.9; HPLC: the ee value was determined by HPLC analysis (Chiralcel IG, *i*-PrOH/Hexane = 35/65, 1.0 mL/min, 271 nm), retention time: t_{minor} = 10.003 min, t_{major} = 13.503 min, ee = 92%; $[\alpha]_D^{25}$ = –11.76 (c = 0.75, $CHCl_3$); HRMS m/z $[M + H]^+$ calculated for $C_{21}H_{23}N_4O_3$: 379.1765, found 379.1761.

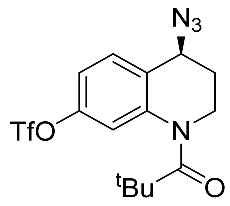

(*S*)-4-Azido-1-pivaloyl-1,2,3,4-tetrahydroquinolin-7-yl trifluoromethanesulfonate (1m)

Yield: 50%, 20.3 mg; ^{1}H NMR (500 MHz, $CDCl_3$) δ 7.65 (d, *J* = 2.5 Hz, 1H), 7.41 (d, *J* = 8.7 Hz, 1H), 7.07 (dd, *J* = 8.6, 2.5 Hz, 1H), 4.63 (t, *J* = 4.7 Hz, 1H), 4.17–4.10 (m, 1H), 3.71–3.63 (m, 1H), 2.20–2.14 (m, 2H), 1.38 (s, 9H); ^{13}C NMR (126 MHz, $CDCl_3$) δ 178.5, 148.7, 141.3, 130.6, 127.0, 119.2 117.5, 56.2, 42.3, 40.6, 29.8, 28.8; ^{19}F NMR (471 MHz, $CDCl_3$) δ -72.88; HPLC: the ee value was determined by HPLC analysis (Chiralcel IG, *i*-PrOH/Hexane = 5/95, 1.0 mL/min, 255 nm), retention time: t_{minor} = 9.300 min, t_{major} = 8.497 min, ee = 90%; $[\alpha]_D^{25}$ = –13.90 (c = 0.30, $CHCl_3$); HRMS m/z $[M + H]^+$ calculated for $C_{15}H_{18}F_3N_4O_4S$: 407.0995, found 407.0987.

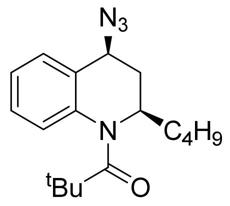

1-((2*R*,4*S*)-4-Azido-2-butyl-3,4-dihydroquinolin-1(2*H*)-yl)-2,2-dimethylpropan-1-one (1n)

Yield: 47%, 14.8 mg; ^{1}H NMR (500 MHz, $CDCl_3$) δ 7.49–7.41 (m, 1H), 7.33–7.27 (m, 2H), 7.23–7.18 (m, 1H), 4.79–4.68 (m, 1H), 4.41–4.28 (m, 1H), 2.79–2.68 (m, 1H), 1.48–1.42 (m, 1H), 1.40–1.35 (m, 1H), 1.29–1.23 (m, 5H), 1.11 (s, 9H), 0.84 (t, *J* = 6.7 Hz, 3H); ^{13}C NMR (126 MHz, $CDCl_3$) δ 178.5, 137.4, 134.7, 127.5, 127.4, 127.0, 124.0, 56.7, 52.0, 41.6, 37.5, 35.7, 29.6, 28.0, 22.7, 14.2; HPLC: the ee value was determined by HPLC analysis (Chiralcel IG, *i*-PrOH/Hexane = 5/95, 1.0 mL/min, 254 nm), retention time: t_{minor} = 6.330 min, t_{major} = 5.587 min, ee = 92%; $[\alpha]_D^{25}$ = – 48.64 (c = 0.43, $CHCl_3$); HRMS m/z $[M + H]^+$ calculated for $C_{18}H_{27}N_4O$: 315.2179, found 315.2177.

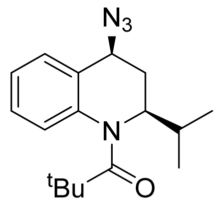

1-((2*S*,4*S*)-4-Azido-2-isopropyl-3,4-dihydroquinolin-1(2*H*)-yl)-2,2-dimethylpropan-1-one (1o)

Yield: 47%, 14.1mg; ^{1}H NMR (500 MHz, $CDCl_3$) δ 7.48–7.43 (m, 1H), 7.36–7.29 (m, 2H), 7.28–7.25 (m, 1H), 4.65–4.59 (m, 1H), 4.34–4.27 (m, 1H), 2.72–2.65 (m, 1H), 1.50–1.43 (m, 1H), 1.41–1.34 (m, 1H), 1.08 (s, 9H), 0.92 (d, *J* = 6.7 Hz, 3H), 0.80 (d, *J* = 6.7 Hz, 3H); ^{13}C NMR (126 MHz, $CDCl_3$) δ 178.3, 137.9, 135.7, 127.5, 127.3, 123.8, 57.2, 57.1, 41.7, 36.0, 33.7, 29.6, 19.5, 18.4; HPLC: the ee value was determined by HPLC analysis (Chiralcel AD-H, *i*-PrOH/Hexane = 1/99, 1.0 mL/min, 243 nm), retention time: t_{minor} = 6.880 min, t_{major} = 5.133 min, ee = 92%; $[\alpha]_D^{25}$ = – 44.23 (c = 1.37, $CHCl_3$); HRMS m/z [M + H]$^+$ calculated for $C_{17}H_{25}N_4O$: 301.2023, found 301.2029.

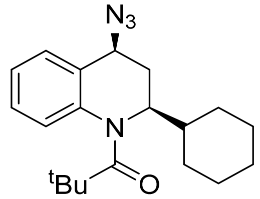

1-((2*S*,4*S*)-4-Azido-2-cyclohexyl-3,4-dihydroquinolin-1(2*H*)-yl)-2,2-dimethylpropan-1-one (1p)

Yield: 45%, 15.3 mg; ^{1}H NMR (500 MHz, $CDCl_3$) δ 7.48–7.42 (m, 1H), 7.35–7.28 (m, 2H), 7.27–7.26 (m, 1H), 4.68–4.61 (m, 1H), 4.32–4.25 (m, 1H), 2.71–2.60 (m, 1H), 1.74–1.63 (m, 3H), 1.60–1.53 (m, 2H), 1.50–1.43 (m, 1H), 1.17–1.09 (m, 2H), 1.07 (s, 9H), 1.05–0.94 (m, 3H), 0.94–0.87 (m, 1H); ^{13}C NMR (126 MHz, $CDCl_3$) δ 178.4, 137.9, 135.9, 127.5, 127.3, 123.7, 57.1, 56.3, 43.3, 41.8, 36.2, 29.7, 29.6, 29.1, 26.5, 26.2, 26.1; HPLC: the ee value was determined by HPLC analysis (Chiralcel IG, *i*-PrOH/Hexane = 5/95, 1.0 mL/min, 250 nm), retention time: t_{minor} = 6.953 min, t_{major} = 5.473 min, ee = 90%; $[\alpha]_D^{25}$ = –24.40 (c = 2.14, $CHCl_3$); HRMS m/z [M + H]$^+$ calculated for $C_{20}H_{29}N_4O$: 341.2336, found 341.2332.

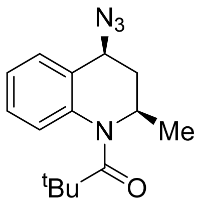

1-((2*R*,4*S*)-4-Azido-2-methyl-3,4-dihydroquinolin-1(2*H*)-yl)-2,2-dimethylpropan-1-one (1q)

Yield: 46%, 12.5 mg; ^{1}H NMR (500 MHz, $CDCl_3$) δ 7.46–7.41 (m, 1H), 7.30–7.26 (m, 2H), 7.22–7.17 (m, 1H), 4.79–4.71 (m, 1H), 4.40–4.33 (m, 1H), 2.78–2.69 (m, 1H), 1.43–1.34 (m, 1H), 1.14 (s, 9H), 1.08 (d, *J* = 6.5 Hz, 3H); ^{13}C NMR (126 MHz, $CDCl_3$) δ 178.7, 136.8, 133.8, 127.5, 127.4, 126.6, 124.3, 56.4, 48.3, 41.5, 39.0, 29.6, 21.1; HPLC: the ee value was determined by HPLC analysis (Chiralcel IG, *i*-PrOH/Hexane = 10/90, 1.0 mL/min, 247 nm), retention time: t_{minor} = 5.783 min, t_{major} = 5.420 min, ee = 95%; $[\alpha]_D^{25}$ = – 20.59 (c = 0.31, $CHCl_3$). HRMS m/z [M + H] $^+$ calculated for $C_{15}H_{21}N_4O$: 273.1710, found 273.1716.

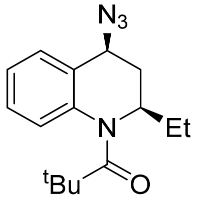

1-((2*R*,4*S*)-4-Azido-2-ethyl-3,4-dihydroquinolin-1(2*H*)-yl)-2,2-dimethylpropan-1-one (1r)

Yield: 45%, 12.9 mg; ^{1}H NMR (500 MHz, $CDCl_3$) δ 7.47–7.42 (m, 1H), 7.32–7.26 (m, 2H), 7.24–7.19 (m, 1H), 4.72–4.63 (m, 1H), 4.38–4.31 (m, 1H), 2.78–2.68 (m, 1H), 1.47–1.36 (m, 2H), 1.23–1.16 (m, 1H), 1.12 (s, 9H), 0.86 (t, *J* = 7.4 Hz, 3H); ^{13}C NMR (126 MHz, $CDCl_3$) δ 178.6, 137.4, 134.6, 127.5, 127.4, 127.0, 124.1, 56.7, 53.3, 41.6, 37.3, 29.6, 29.0, 10.3; HPLC: the ee value was determined by HPLC analysis (Chiralcel IG, *i*-PrOH/Hexane = 5/95, 1.0 mL/min, 249 nm), retention time: t_{minor} = 7.053 min, t_{major} = 5.970 min, ee = 95%. $[\alpha]_D^{25}$ = – 31.83 (c = 0.63, $CHCl_3$). HRMS m/z $[M + H]^+$ calculated for $C_{16}H_{23}N_4O$: 287.1866, found 287.1864.

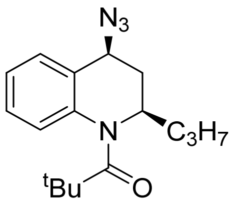

1-((2*R*,4*S*)-4-Azido-2-propyl-3,4-dihydroquinolin-1(2*H*)-yl)-2,2-dimethylpropan-1-one (1s)

Yield: 46%, 13.8 mg; ^{1}H NMR (500 MHz, $CDCl_3$) δ 7.46–7.41 (m, 1H), 7.33–7.27 (m, 2H), 7.22–7.18 (m, 1H), 4.79–4.72 (m, 1H), 4.36–4.30 (m, 1H), 2.77–2.69 (m, 1H), 1.43–1.35 (m, 2H), 1.33–1.27 (m, 2H), 1.26–1.23 (m, 1H), 1.10 (s, 9H), 0.88–0.84 (m, 3H); ^{13}C NMR (126 MHz, $CDCl_3$) δ 178.5, 137.4, 134.7, 127.5, 127.4, 127.0, 124.0, 56.7, 51.8, 41.6, 38.2, 37.5, 29.6, 19.1, 14.1; HPLC: the ee value was determined by HPLC analysis (Chiralcel AD-H, *i*-PrOH/Hexane = 2/98, 0.8 mL/min, 210 nm), retention time: t_{minor} = 7.983 min, t_{major} = 6.840 min, ee = 91%; $[\alpha]_D^{25}$ = – 71.19 (c = 0.22, $CHCl_3$). HRMS m/z $[M + H]^+$ calculated for $C_{17}H_{25}N_4O$: 301.2023, found 301.2027.

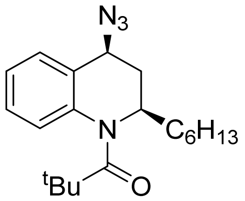

1-((2*R*,4*S*)-4-Azido-2-hexyl-3,4-dihydroquinolin-1(2*H*)-yl)-2,2-dimethylpropan-1-one (1t)

Yield: 45%, 15.4 mg; ^{1}H NMR (500 MHz, $CDCl_3$) δ 7.48–7.40 (m, 1H), 7.33–7.26 (m, 2H), 7.23–7.18 (m, 1H), 4.78–4.68 (m, 1H), 4.38–4.30 (m, 1H), 2.78–2.69 (m, 1H), 1.47–1.41 (m, 1H), 1.39–1.34 (m, 1H), 1.26–1.15 (m, 9H), 1.11 (s, 9H), 0.86–0.82 (m, 3H); ^{13}C NMR (126 MHz, $CDCl_3$) δ 178.5, 137.4, 134.6, 127.5, 127.4, 127.0, 124.0, 56.7, 52.0, 41.6, 37.5, 36.0, 31.9, 29.7, 29.6, 29.3, 25.8, 22.7, 14.2; HPLC: the ee value was determined by HPLC analysis (Chiralcel AD-H, *i*-PrOH/Hexane = 2/98, 0.8 mL/min, 259 nm), retention time: t_{minor} = 7.163 min, t_{major} = 6.370 min, ee = 90%; $[\alpha]_D^{25}$ = – 56.82 (c = 0.51, $CHCl_3$). HRMS m/z $[M + H]^+$ calculated for $C_{20}H_{31}N_4O$:343.2492, found 343.2496.

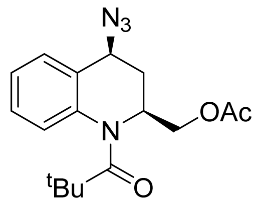

((2*S*,4*S*)-4-Azido-1-pivaloyl-1,2,3,4-tetrahydroquinolin-2-yl)methyl acetate (1u)

Yield: 44%, 14.5 mg; ^{1}H NMR (500 MHz, $CDCl_3$) δ 7.49–7.40 (m, 1H), 7.33–7.28 (m, 2H), 7.24–7.21 (m, 1H), 5.05–4.97 (m, 1H), 4.44–4.32 (m, 1H), 4.01–3.93 (m, 1H), 3.91–3.85 (m, 1H), 2.77–2.68 (m, 1H), 1.92 (s, 3H), 1.53–1.43 (m, 1H), 1.11 (s, 9H); ^{13}C NMR (126 MHz, $CDCl_3$) δ 179.0, 170.9, 137.2, 134.1, 127.8, 127.3, 127.2, 124.1, 65.0, 56.4, 50.5, 41.7, 34.5, 29.6, 20.9; HPLC: the ee value was determined by HPLC analysis (Chiralcel AD-H, *i*-PrOH/Hexane = 10/90, 1.0 mL/min, 243 nm), retention time: t_{minor} = 8.467 min, t_{major} = 7.410 min, ee = 95%; $[\alpha]_D^{25}$ = – 70.40 (c = 1.17, $CHCl_3$). HRMS m/z $[M + H]^+$ calculated for $C_{17}H_{23}N_4O_3$: 331.1765, found 331.1767.

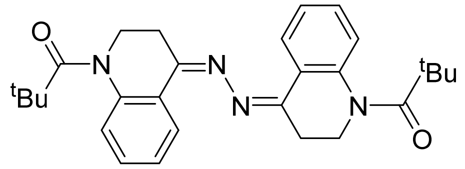

2a

1,1'-((4*Z*,4'*Z*)-Hydrazine-1,2-diylidenebis(3,4-dihydroquinoline-1(2*H*)-yl-4(2*H*)-yl idene)) bis(2,2-dimethylpropan-1-one) (2a)

Yield: 44%, 20.2 mg; ^{1}H NMR (500 MHz, $CDCl_3$) δ 8.22 (dd, *J* = 7.9, 1.1 Hz, 1H), 7.53 (d, *J* = 9.4 Hz, 1H), 7.39–7.32 (m, 1H), 7.22–7.15 (m, 1H), 4.01 (t, *J* = 6.1 Hz, 2H), 2.94 (t, *J* = 6.1 Hz, 2H), 1.38 (s, 9H); ^{13}C NMR (126 MHz, $CDCl_3$, number of scans: 512) δ 177.8, 156.0, 142.7, 130.0, 127.0, 126.2, 125.7, 125.3, 44.8, 40.1, 29.9, 28.9; HRMS m/z $[M + H]^+$ calculated for $C_{28}H_{35}N_4O_2$: 459.2755, found 459.2749.

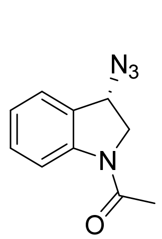

(*S*)-1-(3-Azidoindolin-1-yl)ethan-1-one (3a)

Yield: 48%, 9.7 mg; ^{1}H NMR (500 MHz, $CDCl_3$) δ 8.27 (d, *J* = 8.2 Hz, 1H), 7.39 (t, *J* =

7.4 Hz, 2H), 7.14 (t, J = 7.5 Hz, 1H), 5.01 (d, J = 6.5 Hz, 1H), 4.21–4.14 (m, 1H), 4.01–3.90 (m, 1H), 2.25 (s, 3H); ^{13}C NMR (126 MHz, $CDCl_3$) δ 168.6, 143.1, 131.0, 127.9, 125.06, 124.3, 117.8, 60.0, 55.5, 24.3; HPLC: the ee value was determined by HPLC analysis (Chiralcel OD-H, *i*-PrOH/Hexane = 35/65, 1.0 mL/min, 253 nm), retention time: tminor = 9.783 min, tmajor = 7.627 min, ee = 96%; $[\alpha]_D^{25}$ = 102.41 (c = 0.20, $CHCl_3$); HRMS m/z $[M + H]^+$ calculated for $C_{10}H_{11}N_4O$: 203.0927, found 203.0925.

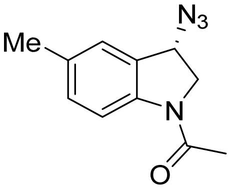

(*S*)-1-(3-Azido-5-methylindolin-1-yl)ethan-1-one (3b)

Yield: 49%, 10.6 mg; ^{1}H NMR (500 MHz, $CDCl_3$) δ 8.21–8.08 (m, 1H), 7.20–7.16 (m, 2H), 5.01–4.92 (m, 1H), 4.19–4.13 (m, 1H), 3.98–3.91 (m, 1H), 2.36 (s, 3H), 2.23 (s, 3H); ^{13}C NMR (126 MHz, $CDCl_3$) δ 168.3, 140.8, 134.1, 131.6, 128.0, 125.4, 117.4, 60.1, 55.6, 24.2, 21.2; HPLC: the ee value was determined by HPLC analysis (Chiralcel OD-H, *i*-PrOH/Hexane = 20/80, 1.0 mL/min, 265 nm), retention time: tminor = 10.690 min, tmajor = 11.283 min, ee = 92%; $[\alpha]_D^{25}$ = 62.33 (c = 0.17, $CHCl_3$); HRMS m/z $[M + H]^+$ calculated for $C_{11}H_{13}N_4O$: 217.1084, found 217.1092.

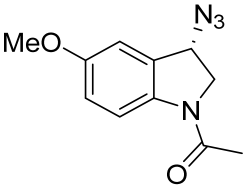

(*S*)-1-(3-Azido-5-methoxyindolin-1-yl)ethan-1-one (3c)

Yield: 48%, 11.1 mg; ^{1}H NMR (500 MHz, $CDCl_3$) δ 8.21–8.16 (m, 1H), 6.92–6.89 (m, 2H), 4.98–4.93 (m, 1H), 4.21–4.16 (m, 1H), 3.97–3.93 (m, 1H), 3.81 (s, 3H), 2.22 (s, 3H); ^{13}C NMR (126 MHz, $CDCl_3$) δ 167.9, 156.6, 136.8, 129.2, 118.5, 116.0, 110.5, 60.1, 56.0, 55.7, 24.1; HPLC: the ee value was determined by HPLC analysis (Chiralcel AD-H, *i*-PrOH/Hexane = 35/65, 1.0 mL/min, 259 nm), retention time: tminor = 8.503 min, tmajor = 7.760 min, ee = 91%; $[\alpha]_D^{25}$ = 16.80 (c = 0.34, $CHCl_3$); HRMS m/z [M +

H]$^+$ calculated for $C_{11}H_{13}N_4O_2$: 233.1033, found 233.1039.

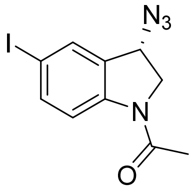

(*S*)-1-(3-Azido-5-iodoindolin-1-yl)ethan-1-one (3d)

Yield: 48%, 15.7 mg; ^{1}H NMR (500 MHz, CDCl$_3$) δ 8.04 (d, *J* = 8.5 Hz, 1H), 7.70–7.65 (m, 2H), 5.01–4.94 (m, 1H), 4.22–4.17 (m, 1H), 3.98–3.93 (m, 1H), 2.24 (s, 3H); ^{13}C NMR (126 MHz, CDCl$_3$) δ 168.7, 142.8, 139.8, 133.9, 130.5, 119.6, 86.7, 59.3, 55.4, 24.3; HPLC: the ee value was determined by HPLC analysis (Chiralcel OD-H, *i*-PrOH/Hexane = 35/65, 1.0 mL/min, 264 nm), retention time: t_{minor} = 10.083 min, t_{major} = 7.617 min, ee = 94%; $[\alpha]_D^{25}$ = 12.70 (c = 0.44, CHCl$_3$); HRMS m/z [M + H]$^+$ calculated for $C_{10}H_{10}IN_4O$: 328.9894, found 328.9886.

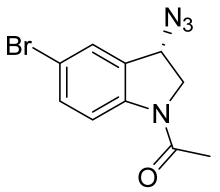

(*S*)-1-(3-Azido-5-bromoindolin-1-yl)ethan-1-one (3e)

Yield: 48%, 13.5 mg; ^{1}H NMR (500 MHz, CDCl$_3$) δ 8.13 (d, *J* = 8.6 Hz, 1H), 7.49–7.43 (m, 2H), 5.00–4.93 (m, 1H), 4.22–4.16 (m, 1H), 3.99–3.91 (m, 1H), 2.22 (s, 3H); ^{13}C NMR (126 MHz, CDCl$_3$) δ 168.5, 142.0, 133.7, 130.1, 127.9, 119.0, 116.3, 59.3, 55.4, 24.1; HPLC: the ee value was determined by HPLC analysis (Chiralcel IG, *i*-PrOH/Hexane = 35/65, 1.0 mL/min, 258 nm), retention time: t_{minor} = 10.323 min, t_{major} = 8.493 min, ee = 97%; $[\alpha]_D^{25}$ = 31.70 (c = 0.24, CHCl$_3$); HRMS m/z [M + H]$^+$ calculated for $C_{10}H_{10}{}^{79}BrN_4O$: 281.0033, found 281.0039, calculated for $C_{10}H_{10}{}^{81}BrN_4O$: 283.0013, found 283.0017.

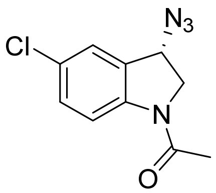

(*S*)-1-(3-Azido-5-chloroindolin-1-yl)ethan-1-one (3f)

Yield: 47%, 11.1 mg; ^{1}H NMR (500 MHz, $CDCl_3$) δ 8.21 (d, *J* = 8.5 Hz, 1H), 7.36–7.32 (m, 2H), 5.02–4.94 (m, 1H), 4.25–4.17 (m, 1H), 4.02–3.94 (m, 1H), 2.24 (s, 3H); ^{13}C NMR (126 MHz, $CDCl_3$) δ 168.6, 141.7, 131.0, 129.7, 129.2, 125.1, 118.7, 59.5, 55.6, 24.2; HPLC: the ee value was determined by HPLC analysis (Chiralcel IG, *i*-PrOH/Hexane = 35/65, 1.0 mL/min, 272 nm), retention time: t_{minor} = 8.843 min, t_{major} = 10.430 min, ee = 97%; $[\alpha]_D^{25}$ = 24.01 (c = 0.28, $CHCl_3$); HRMS m/z $[M + H]^+$ calculated for $C_{10}H_{10}ClN_4O$: 237.0538, found 237.0532.

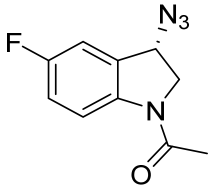

(*S*)-1-(3-Azido-5-fluoroindolin-1-yl)ethan-1-one (3g)

Yield: 51%, 11.2 mg; ^{1}H NMR (500 MHz, $CDCl_3$) δ 8.24 (dd, *J* = 8.6, 4.7 Hz, 1H), 7.10–7.05 (m, 2H), 4.98 (dd, *J* = 7.9, 2.5 Hz, 1H), 4.25–4.20 (m, 1H), 4.02–3.95 (m, 1H), 2.23 (s, 3H); ^{13}C NMR (126 MHz, $CDCl_3$) δ 168.3, 160.3, 158.4, 139.3, 139.3, 129.6, 129.5, 118.8, 118.7, 117.6, 117.4, 112.2, 112.0, 59.6, 55.7, 24.1; ^{19}F NMR (471 MHz, $CDCl_3$) δ -117.74; HPLC: the ee value was determined by HPLC analysis (Chiralcel IG, *i*-PrOH/Hexane = 35/65, 1.0 mL/min, 264 nm), retention time: t_{minor} = 7.543 min, t_{major} = 8.513 min, ee = 90%; $[\alpha]_D^{25}$ = 57.25 (c = 0.32, $CHCl_3$); HRMS m/z $[M + H]^+$ calculated for $C_{10}H_{10}FN_4O$: 221.0833, found 221.0837.

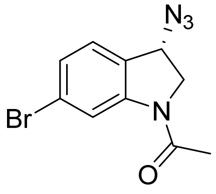

(*S*)-1-(3-Azido-6-bromoindolin-1-yl)ethan-1-one (3h)

Yield: 48%, 13.5 mg; ^{1}H NMR (500 MHz, $CDCl_3$) δ 8.46 (s, 1H), 7.28–7.23 (m, 2H), 5.01–4.93 (m, 1H), 4.23–4.17 (m, 1H), 3.99–3.93 (m, 1H), 2.24 (s, 3H); ^{13}C NMR (126 MHz, $CDCl_3$) δ 168.7, 144.1, 127.2, 127.0, 126.1, 124.9, 120.8, 59.4, 55.8, 24.2; HPLC: the ee value was determined by HPLC analysis (Chiralcel OD-H, *i*-PrOH/Hexane = 35/65, 1.0 mL/min, 265 nm), retention time: t_{minor} = 9.003 min, t_{major} = 6.870 min, ee = 97%; $[\alpha]_D^{25}$ = 73.16 (c = 0.39, $CHCl_3$); HRMS m/z $[M + H]^+$ calculated for $C_{10}H_{10}{}^{79}BrN_4O$: 281.0033, found 281.0035, calculated for $C_{10}H_{10}{}^{81}BrN_4O$: 283.0013, found 283.0019.

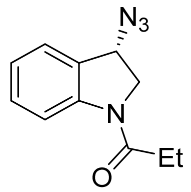

(*S*)-1-(3-Azidoindolin-1-yl)propan-1-one (3i)

Yield: 49%, 10.6 mg; ^{1}H NMR (500 MHz, $CDCl_3$) δ 8.30 (d, *J* = 8.3 Hz, 1H), 7.38 (t, *J* = 7.4 Hz, 2H), 7.13 (t, *J* = 7.5 Hz, 1H), 5.08–4.94 (m, 1H), 4.17–4.12 (m, 1H), 4.01–3.90 (m, 1H), 2.58–2.47 (m, 1H), 2.46–2.35 (m, 1H), 1.24 (t, *J* = 7.3 Hz, 3H); ^{13}C NMR (126 MHz, $CDCl_3$) δ 172.0, 143.3, 131.0, 127.8, 125.0, 124.1, 117.7, 60.0, 54.6, 29.4, 8.8; HPLC: the ee value was determined by HPLC analysis (Chiralcel IG, *i*-PrOH/Hexane = 20/80, 1.0 mL/min, 253 nm), retention time: t_{minor} = 9.480 min, t_{major} = 12.687 min, ee = 92%; $[\alpha]_D^{25}$ = 18.73 (c = 0.31, $CHCl_3$); HRMS m/z $[M + H]^+$ calculated for $C_{11}H_{13}N_4O$: 217.1084, found 217.1088.

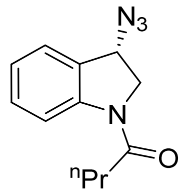

(*S*)-1-(3-Azidoindolin-1-yl)butan-1-one (3j)

Yield: 48%, 11.1 mg; ^{1}H NMR (500 MHz, $CDCl_3$) δ 8.31 (d, *J* = 8.0 Hz, 1H), 7.38 (t, *J* = 7.6 Hz, 2H), 7.13 (t, *J* = 7.5 Hz, 1H), 5.07–4.92 (m, 1H), 4.18–4.13 (m, 1H), 4.03–3.91 (m, 1H), 2.51–2.41 (m, 1H), 2.42–2.31 (m, 1H), 1.81–1.75 (m, 2H), 1.03 (t, *J* = 7.4

Hz, 3H); ^{13}C NMR (126 MHz, $CDCl_3$) δ 171.3, 143.3, 131.0, 127.8, 125.0, 124.1, 117.8, 60.0, 54.8, 38.1, 18.1, 14.1; HPLC: the ee value was determined by HPLC analysis (Chiralcel AD-H, *i*-PrOH/Hexane = 20/80, 1.0 mL/min, 253 nm), retention time: t_{minor} = 6.770 min, t_{major} = 5.870 min, ee = 94%; $[\alpha]_D^{25}$ = 19.20 (c = 0.23, $CHCl_3$); HRMS m/z $[M + H]^+$ calculated for $C_{12}H_{15}N_4O$: 231.1240, found 231.1248.

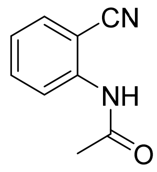

N-(2-cyanophenyl)acetamide (4a)

Yield: 42%, 6.7 mg; ^{1}H NMR (500 MHz, $CDCl_3$) δ 8.37 (d, *J* = 8.4 Hz, 1H), 7.74 (s, 1H), 7.60–7.55 (m, 2H), 7.19–7.14 (m, 1H), 2.26 (s, 3H); ^{13}C NMR (126 MHz, $CDCl_3$) δ 168.9, 140.7, 134.4, 132.5, 124.3, 121.6, 116.6, 102.1, 24.9; HRMS m/z $[M + H]^+$ calculated for $C_9H_9N_2O$: 161.0709, found 161.0715

Absolute Configuration Determination

Absolute configuration determination of THQ-based organic azides.

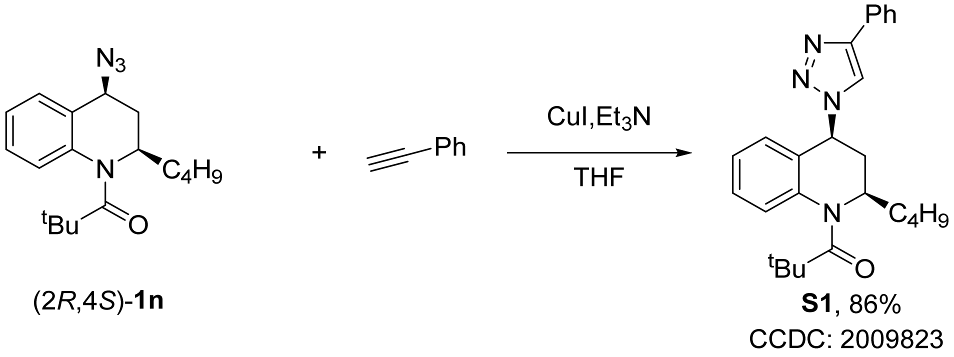

To a mixture of phenylacetylene (0.12 mmol, 1.2 equiv), (2*R*,4*S*)-**1n** (0.1 mmol, 1.0 equiv) and Et_3N (0.2 mmol, 2.0 equiv) in THF (1.0 mL) was added CuI (0.005 mmol, 0.05 equiv). Then the reaction mixture was stirred at room temperature until (2*R*,4*S*)-**1n** was consumed monitored by TLC. The reaction was quenched with saturated $NaHCO_3$ (5 mL), then the mixture was extracted with ethyl acetate (3 x 10 mL). The organic phases were combined and dried over anhydrous $MgSO_4$. The solvent was removed in vacuo and the residue was purified by silica chromatography to afford **9**. Yield: 86%, 35.8 mg. ^{1}H NMR (500 MHz, $CDCl_3$) δ 7.90 (d, J = 7.9 Hz, 3H), 7.46 (t, J = 7.5 Hz, 2H), 7.39–7.27 (m, 3H), 7.19 (t, J = 7.4 Hz, 1H), 6.49 (d, J = 7.6 Hz, 1H), 5.65–5.57 (m, 1H), 4.93–4.84 (m, 1H), 3.00–2.91 (m, 1H), 2.11–2.00 (m, 1H), 1.66–1.57 (m, 1H), 1.33–1.26 (m, 5H), 1.17 (s, 9H), 0.87 (t, J = 6.8 Hz, 3H); ^{13}C NMR (126 MHz, $CDCl_3$, number of scans: 512) δ 178.7, 137.3, 135.1, 130.6, 129.1, 128.6, 128.1, 127.5, 127.3, 126.0, 123.2, 57.3, 52.3, 41.8, 38.5, 36.1, 29.7, 28.0, 22.7, 14.2; HRMS m/z $[M + H]^+$ calculated for $C_{26}H_{33}N_4O$: 417.2649, found 417.2657. The absolute stereochemistry of **S1** was determined by the X-ray diffraction. A suitable crystal was selected and analyzed on a Bruker APEX-II CCD diffractometer. The flack parameter is 0.10(16). Further information is contained in the CCDC file 2009823.

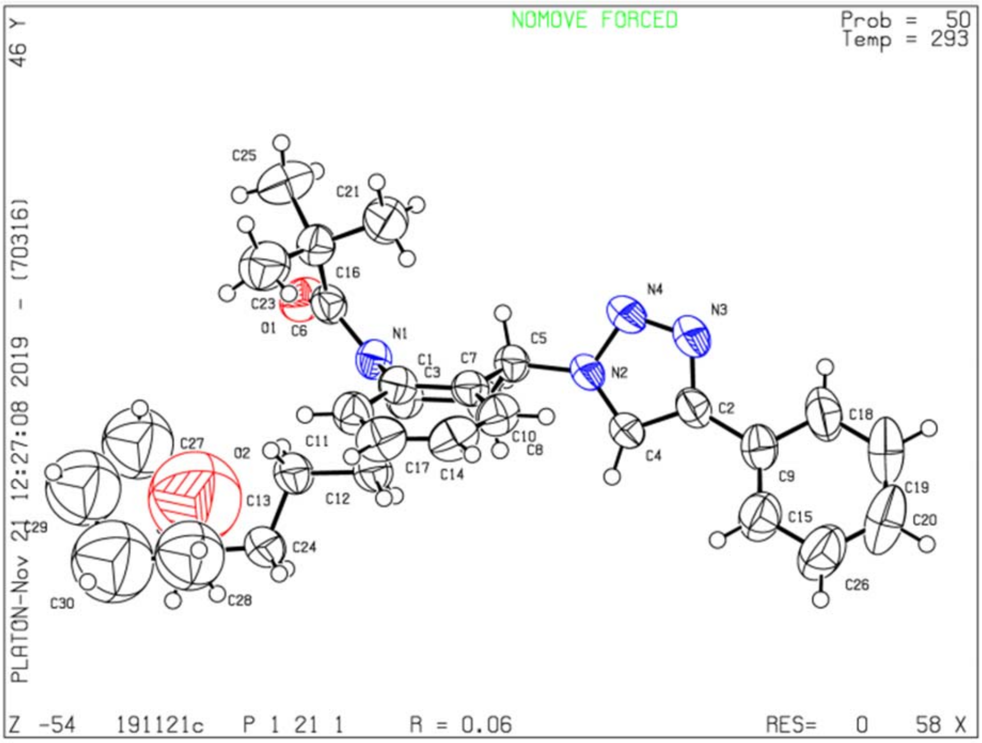

Supplementary Figure 1. Single Crystal XRD structure of **S1**.

Absolute configuration determination of indoline-based organic azides.

The absolute configuration of indoline-based azides (*S*)-**3a** was confirmed by employing CD spectroscopy measurements and theoretical calculations.

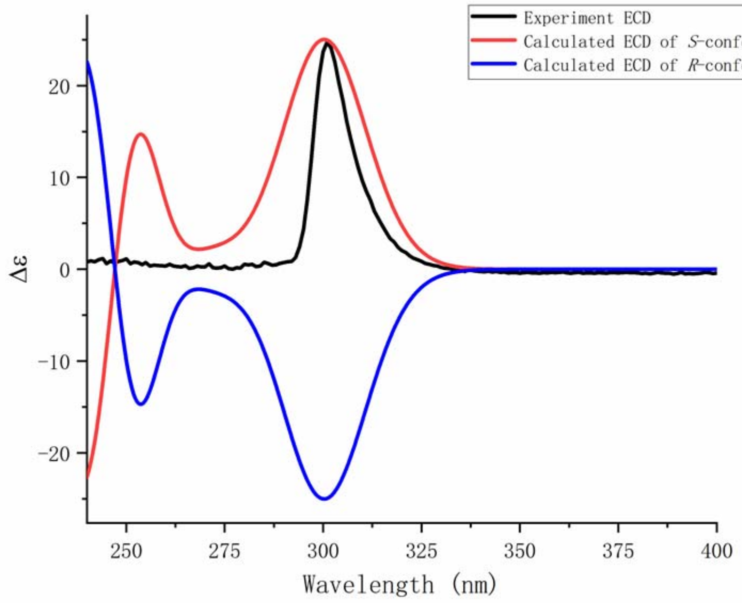

Supplementary Figure 2. CD spectroscopy.

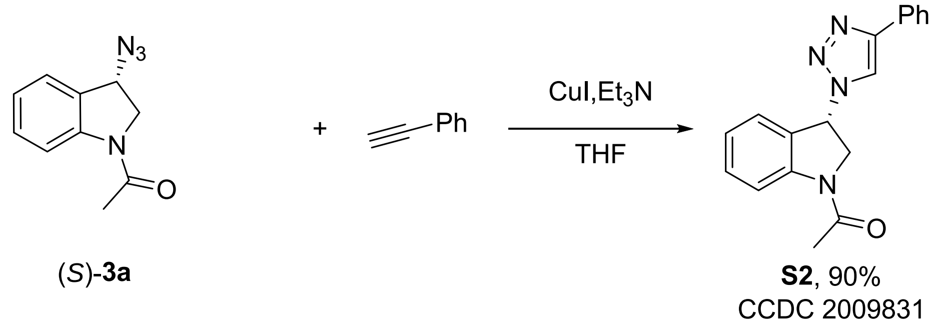

To a mixture of phenylacetylene (0.12 mmol, 1.2 equiv), (S)-**3a** (0.1 mmol, 1.0 equiv) and Et_3N (0.2 mmol, 2.0 equiv) in THF (1.0 mL) was added CuI (0.005 mmol, 0.05 equiv). Then the reaction mixture was stirred at room temperature until (S)-**3a** was consumed monitored by TLC. The reaction was quenched with saturated $NaHCO_3$ (5 mL), then the mixture was extracted with ethyl acetate (3 x 10 mL). The organic phases were combined and dried over anhydrous $MgSO_4$. The solvent was removed in vacuo and the residue was purified by silica chromatography to afford **S2**. Yield: 90%, 27.4 mg. ^{1}H NMR (500 MHz, $CDCl_3$) δ 8.38 (d, *J* = 8.2 Hz, 1H), 7.76–7.72 (m, 2H), 7.46 (t, *J* = 7.8 Hz, 1H), 7.40–7.36 (m, 3H), 7.34–7.30 (m, 2H), 7.16 (t, *J* = 7.4 Hz, 1H), 6.46–6.35 (m, 1H), 4.59–4.54 (m, 1H), 4.29–4.21 (m, 1H), 2.23 (s, 3H); ^{13}C NMR (126 MHz, $CDCl_3$) δ 168.8, 148.6, 143.8, 131.7, 130.3, 129.0, 128.6, 126.5, 125.9, 125.8, 124.9, 118.1, 117.8, 59.9, 56.3, 24.4; HRMS m/z $[M + H]^+$ calculated for $C_{18}H_{17}N_4O$: 305.1397, found 305.1391. The absolute stereochemistry of **S2** was determined by the X-ray diffraction. A suitable crystal was selected and analyzed on a Bruker APEX-II CCD diffractometer. The flack parameter is 0.3(2). Further information is contained in the CCDC file 2009831.

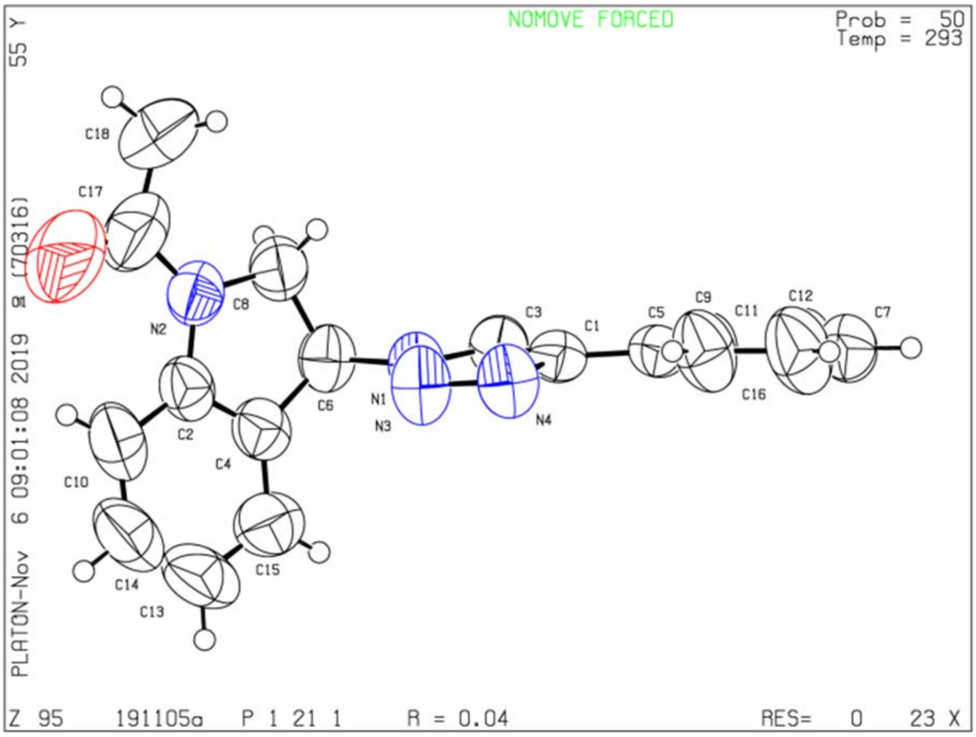

Supplementary Figure 3. Single Crystal XRD structure of **S2**.

Synthetic Applications

Copper-catalyzed azide–alkyne cycloaddition (AAC) of vitamin E.[6]

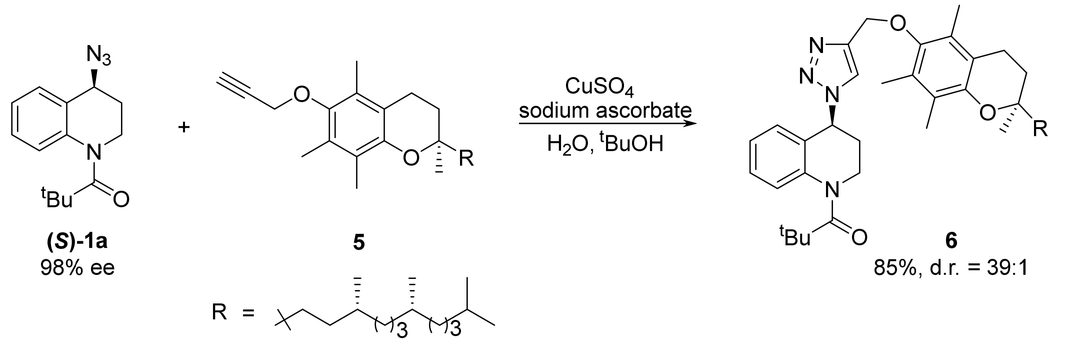

To a mixture of **5** (0.12 mmol, 1.2 equiv), ***(S)*-1a** (0.1 mmol, 1.0 equiv) and sodium ascorbate (0.2 mmol, 2.0 equiv) in $^tBuOH/H_2O$ (2.0 mL, v/v = 1:1) was added $CuSO_4{\bullet}5H_2O$ (0.005 mmol, 0.05 equiv). Then the reaction mixture was stirred at room temperature until ***(S)*-1a** was consumed monitored by TLC. The reaction was quenched with saturated $NaHCO_3$ (5 mL), then the mixture was extracted with ethyl acetate (3 x 10 mL). The organic phases were combined and dried over anhydrous $MgSO_4$. The solvent was removed in vacuo and the residue was purified by silica chromatography to afford **6**. Yield: 85%, 61.8 mg. ^1H NMR (500 MHz, $CDCl_3$) δ 7.58 (t, *J* = 7.3 Hz, 1H), 7.45 (s, 1H), 7.32 (t, *J* = 7.6 Hz, 1H), 7.12 (t, *J* = 7.3 Hz, 1H), 7.01 (d, *J* = 7.5 Hz, 1H), 6.06–5.91 (m, 1H), 4.83 (s, 2H), 4.11–4.01 (m, 1H), 3.94–3.82 (m, 1H), 2.67–2.52 (m, 3H), 2.50–2.42 (m, 1H), 2.15 (s, 3H), 2.13 (s, 3H), 2.06 (s, 3H), 1.85–1.71 (m, 2H), 1.60–1.38 (m, 6H), 1.37 (s, 9H), 1.33–1.23 (m, 7H), 1.23 (s, 3H), 1.21–1.00 (m, 8H), 0.88–0.82 (m, 12H); ^{13}C NMR (126 MHz, $CDCl_3$, number of scans: 1024) δ 178.6, 148.3, 147.8, 140.6, 128.9, 128.8, 128.1, 126.6, 126.1, 125.9, 125.6, 123.1, 117.8, 75.1, 66.3, 56.7, 43.1, 40.4, 40.3, 39.6, 37.7, 37.65, 37.6, 37.5, 33.0, 32.9, 32.5, 31.4, 28.9, 28.2, 25.0, 24.6, 24.1, 22.9, 22.8, 21.2, 20.9, 20.0, 19.9, 13.1, 12.3, 12.0; HPLC: the dr value was determined by HPLC analysis (Chiralcel OD-H, *i*-PrOH/Hexane = 20/80, 1.0 mL/min, 223 nm), retention time: t_{minor} = 9.043 min, t_{major} = 6.797 min, d.r. = 39:1%; $[\alpha]_D^{25}$ = –74.52 (c = 2.15, $CHCl_3$); HRMS m/z $[M + H]^+$ calculated for $C_{46}H_{71}N_4O_3$: 727.5521, found 727.5539.

Copper-catalyzed azide–alkyne cycloaddition (AAC) of estrone derivative.[7]

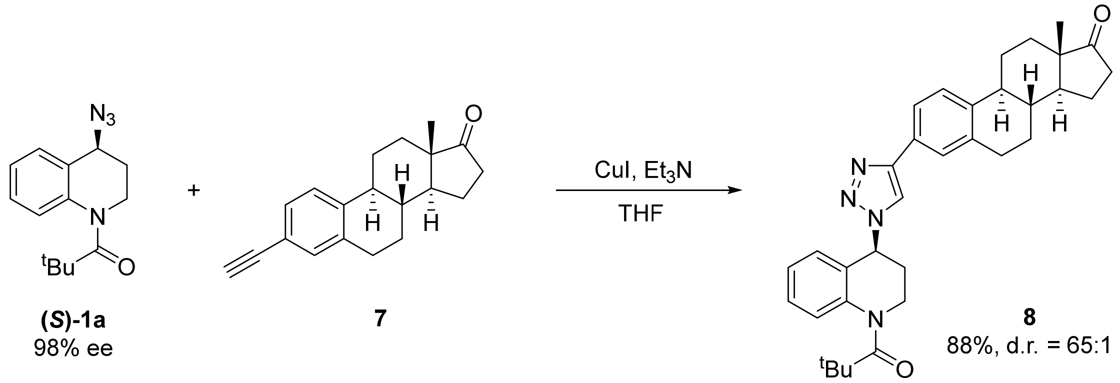

(*S*)-1a 98% ee **7**

To a mixture of **7** (0.12 mmol, 1.2 equiv), **(*S*)-1a** (0.1 mmol, 1.0 equiv) and Et_3N (0.2 mmol, 2.0 equiv) in THF (1.0 mL) was added CuI (0.005 mmol, 0.05 equiv). Then the reaction mixture was stirred at room temperature until **(*S*)-1a** was consumed monitored by TLC. The reaction was quenched with saturated $NaHCO_3$ (5 mL), then the mixture was extracted with ethyl acetate (3 x 10 mL). The organic phases were combined and dried over anhydrous $MgSO_4$. The solvent was removed in vacuo and the residue was purified by silica chromatography to afford **8**. Yield: 88%, 47.2 mg. ^{1}H NMR (500 MHz, $CDCl_3$) δ 7.64–7.46 (m, 4H), 7.36–7.30 (m, 2H), 7.12 (t, *J* = 7.1 Hz, 1H), 7.04 (d, *J* = 7.6 Hz, 1H), 6.03–5.97 (m, 1H), 4.06–3.99 (m, 1H), 3.98–3.91 (m, 1H), 2.99–2.91 (m, 2H), 2.65–2.58 (m, 1H), 2.54–2.41 (m, 3H), 2.35–2.29 (m, 1H), 2.19–2.10 (m, 1H), 2.09–2.01 (m, 2H), 1.99–1.95 (m, 1H), 1.65–1.45 (m, 6H), 1.36 (s, 9H), 0.91 (s, 3H); ^{13}C NMR (126 MHz, $CDCl_3$, number of scans: 1024) δ 221.0, 178.6, 140.6, 140.2, 137.2, 128.9, 128.8, 127.9, 126.6, 126.4, 126.11, 126.1, 126.0, 125.6, 123.3, 118.8, 56.9, 50.6, 48.1, 44.6, 43.3, 40.3, 38.2, 36.0, 32.6, 31.7, 29.5, 28.8, 26.6, 25.8, 21.7, 14.0; HPLC: the dr was determined by HPLC analysis (Chiralcel AD-H, *i*-PrOH/Hexane = 20/80, 1.0 mL/min, 211 nm), retention time: t_{minor} = 26.433 min, t_{major} = 23.963 min, d.r. = 65:1; $[\alpha]_D^{25}$ = –81.56 (c = 1.74, $CHCl_3$); HRMS m/z $[M + H]^+$ calculated for $C_{34}H_{41}N_4O_2$: 537.3224, found 537.3236.

Oxidative kinetic resolution of acyclic organic azide

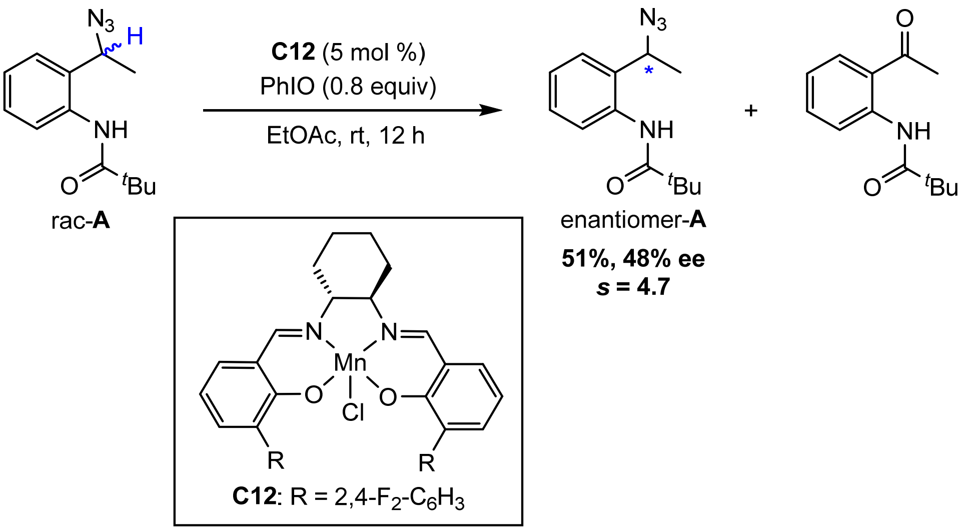

To a solution of rac-**A** (0.1 mmol, 1.0 equiv) in ethyl acetate (1.0 mL) was added **C12** (0.005 mmol, 0.05 equiv) at r.t. Then PhIO (0.08 mmol, 0.8 equiv) was added as 8 portions in 30-min intervals over 3.5 h. After 12 h, the solvent was removed under vacuum and the residue was purified by flash chromatography on silica gel using ethyl acetate/petroleum ether as eluent to give the product enantiomer-**A** (51% yield, 12.5mg). ^{1}H NMR (500 MHz, $CDCl_3$) δ 8.28 (s, 1H), 8.03 (d, J = 8.1 Hz, 1H), 7.37 – 7.31 (m, 1H), 7.23 (dd, J = 7.7, 1.3 Hz, 1H), 7.12 (td, J = 7.6, 1.1 Hz, 1H), 4.60 (q, J = 6.9 Hz, 1H), 1.63 (d, J = 6.9 Hz, 3H), 1.34 (s, 9H); ^{13}C NMR (126 MHz, $CDCl_3$) δ 176.9, 136.4, 130.2, 129.3, 127.3, 124.8, 124.3, 60.1, 39.9, 27.9, 19.0; HPLC: the ee value was determined by HPLC analysis (Chiral NY(2) 5u, *i*-PrOH/Hexane = 20/80, 1.0 mL/min, 211 nm), retention time: t_{major} = 5.537 min, t_{minor} = 6.790 min, ee = 48%; HRMS m/z $[M + H]^+$ calculated for $C_{13}H_{19}N_4O$: 247.1553, found 247.1558.

Control experiments

Correlation of the enantiomeric excess of C7 and 3a

The EtOAc solutions of **C7** and ent-**C7** (0.005 M, respectively) were prepared and mixed to regulate each complex solution (10% ee, 20% ee, 40% ee, 60% ee, 80% ee and 100% ee, 0.005 M, respectively) in an appropriate manner. To the solutions, **3a** (0.1 mmol, 20.2 mg) was added. Then PhIO (0.03 mmol, 0.3 eq) was added as 3 portions in 30-min intervals over 1.0 h (20% conversion of **3a**). After that, the solvent was removed under vacuum and the residue was purified by flash chromatography on silica gel using ethyl acetate/petroleum ether as eluent. The ee values of **3a** were determined by HPLC analysis on chiral phase column (Chiralpak OD, *i*-PrOH/Hexane = 35/65, 1.0 mL/min, 253 nm).

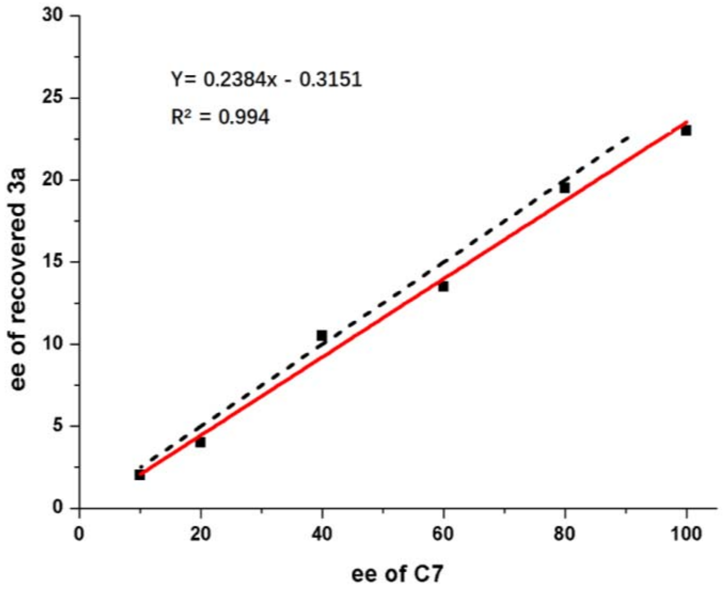

Supplementary figure 4. Plot of enantiomeric excess of recovered **3a** versus the enantiomeric excess of **C7** at 20% conversion. The dotted line symbolized the linear correlation.

Stoichiometric Mn(salen) C12 mediated control experiment

To a solution of rac-**1a** (0.0.5 mmol, 1.0 equiv) in ethyl acetate (0.5 mL) was added **C12** (0.05 mmol, 0.5 equiv) at r.t. No reaction was observed in the absence of PhIO.

Kinetic isotope effect experiments

Preparation of [D]-**1a**

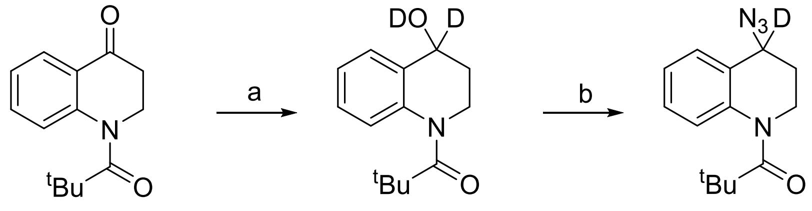

S-8 **S-9** [D]-**1a**

a) To a solution of **S-8** (3.0 mmol, 1.0 equiv) in dry MeOH (10 mL) was added $NaBD_4$ (6.0 mmol, 2.0 equiv) in batches at 0 °C. The mixture was stirred for another 30 min before it was quenched with saturated $NaHCO_3$ solution. It was diluted with CH_2Cl_2 and washed with brine. The organic layer was separated, dried over $MgSO_4$, and concentrated in vacuo. The residue was purified by flash chromatography to give **S-9**.

b) To a solution of **S9** (3 mmol, 1.0 equiv) in dry toluene (50 mL) was added DBU (4.5 mmol, 1.5 equiv) and DPPA (4.5 mmol, 1.5 equiv) dropwise at 0 °C. Then the reaction mixture was allowed to warm up to room temperature and stirred until **S9** was consumed monitored by TLC. The reaction was quenched with saturated NH_4Cl (10 mL) at 0 °C and extracted with ethyl acetate (3 x 20 mL). The organic phases were combined and dried over $MgSO_4$. The solvent was removed under vacuum and the residue was purified by silica chromatography to afford [D]-**1a**. ^{1}H NMR (500 MHz, $CDCl_3$) δ 7.51 (dd, J = 8.2, 1.0 Hz, 1H), 7.34 (dd, J = 7.7, 1.5 Hz, 1H), 7.29–7.25 (m, 1H), 7.17 (td, J = 7.5, 1.2 Hz, 1H), 4.10–4.04 (m, 1H), 3.70–3.63 (m, 1H), 2.22–2.15 (m, 1H), 2.14–2.07 (m, 1H), 1.34 (s, 9H); ^{13}C NMR (126 MHz, $CDCl_3$) δ 178.8, 139.9, 128.6, 128.3, 127.5, 126.1, 125.1, 57.0, 56.7, 56.6, 56.4, 42.5, 40.6, 30.5, 29.1; HRMS m/z $[M + H]^+$ calculated for $C_{14}H_{18}DN_4O$: 260.1616, found 260.1622.

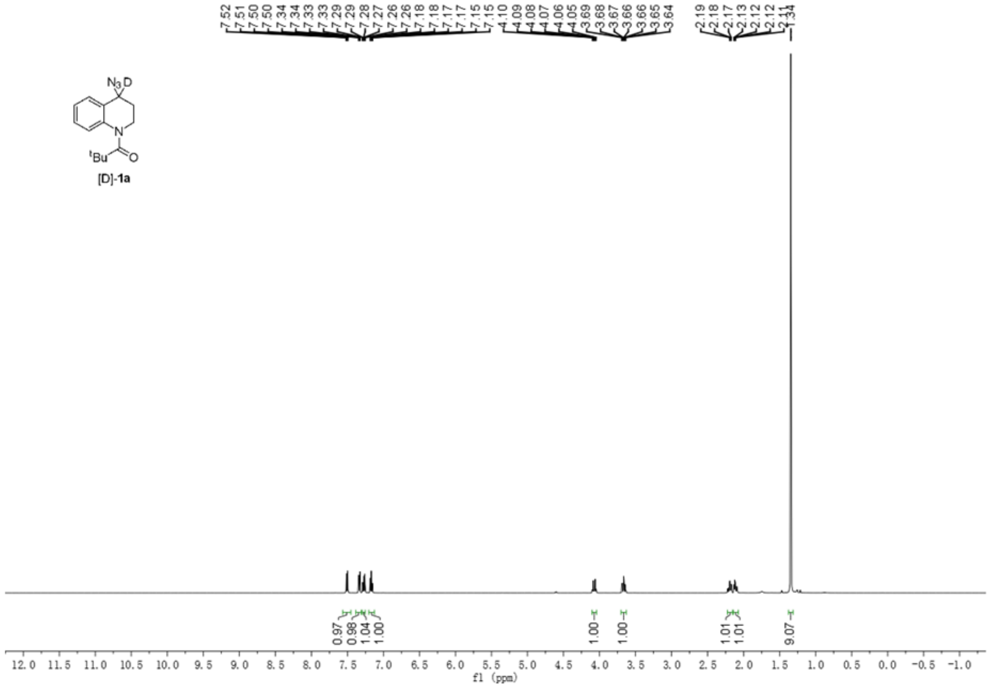

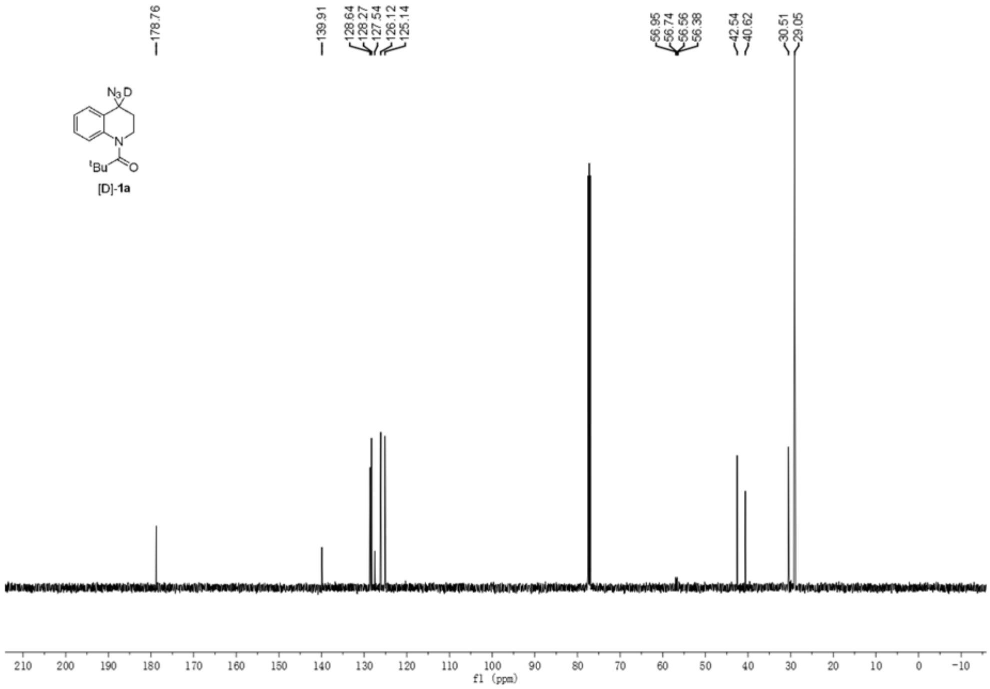

Supplementary figure 5. ^{1}H & ^{13}C NMR spectra of [D]-**1a**

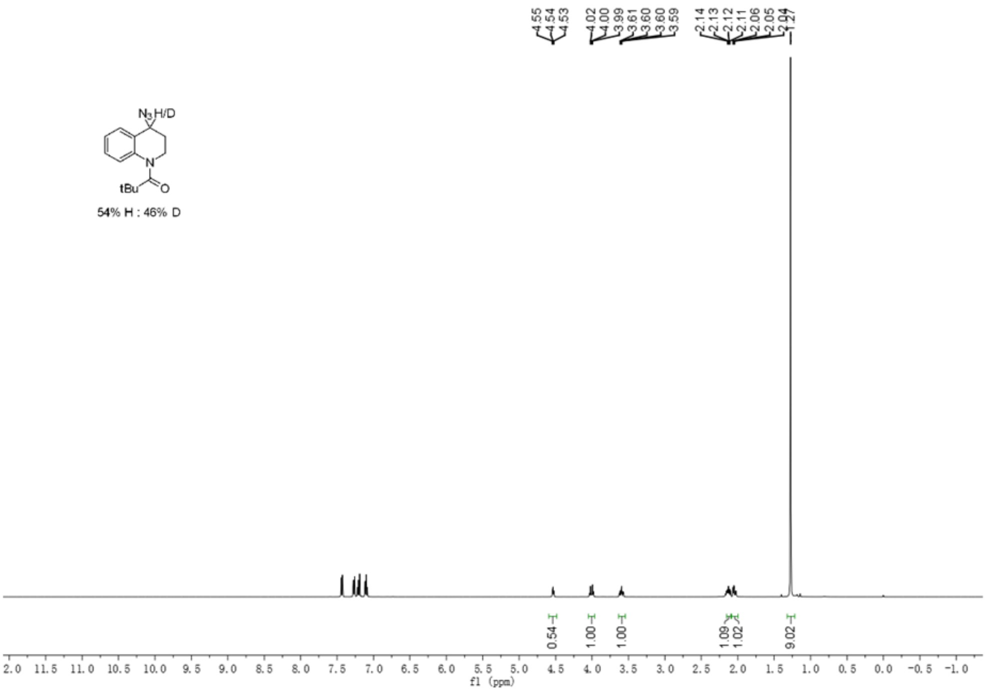

Supplementary figure 6. ^{1}H NMR of the mixture before oxidation

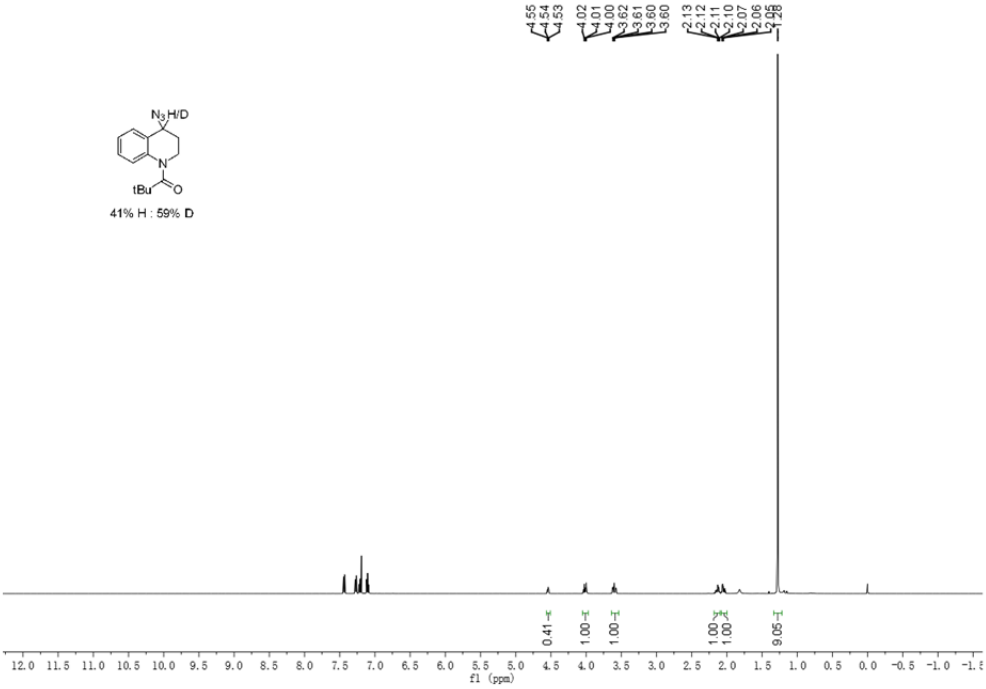

Supplementary figure 7. ^{1}H NMR of the mixture after oxidation

Intermolecular competition KIE experiment

[H]-**1a** (0.1 mmol) and [D]-**1a** (0.1 mmol) were added to a flask containing 2.0 mL CH_2Cl_2 and it was stirred for 10 min before the solvent was removed. The initial ratio of the well-mixed [H]-**1a** and [D]-**1a** was determined by ^{1}H NMR which shows [H]-**1a** / [D]-**1a** = 54% H: 46% D.

The well-mixed [H]-**1a** and [D]-**1a** mixture (52.0 mg, 0.2 mmol) was dissolved in EtOAc (2.0 mL), and **C12** (0.010 mmol) was added. Then PhIO (0.12 mmol) was added portionwise. After 3 h, the mixture was passed through a short pad of silica gel to afford the remaining [H]-**1a** and [D]-**1a** (30.8 mg, conversion 41.0%). The ratio of remaining [H]-**1a** and [D]-**1a** was analyzed by ^{1}H NMR which shows [H]-**1a** / [D]-**1a** = 41% H: 59% D.

The KIE was calculated as follows:

$KIE = K_H / K_D = [(C_{H0}-C_{Ht})/t] / [(C_{D0}-C_{Dt})/t] = [(M_{H0}-M_{Ht})/v] / [(M_{D0}-M_{Dt})/v] = (M_{H0}-M_{Ht}) / (M_{D0}-M_{Dt}) = (52*0.54-30.8*0.41) / (52*0.46-30.8*0.59) = 2.7$

The *N*-acyl substituent effect for THQ-based azides

To a solution of rac-**9** (0.1 mmol, 1.0 equiv) in ethyl acetate (1.0 mL) was added **C12** (0.005 mmol, 0.05 equiv) at r.t. Then PhIO (0.08 mmol, 0.8 equiv) was added as 8 portions in 30-min intervals over 3.5 h. After 24 h, the solvent was removed under vacuum and the residue was purified by flash chromatography on silica gel using ethyl acetate/petroleum ether as eluent.

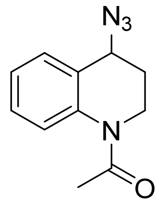

1-(4-Azido-3,4-dihydroquinolin-1(2H)-yl)ethan-1-one (9a)

No reaction. ^{1}H NMR (500 MHz, $CDCl_3$) δ7.39 – 7.29 (m, 3H), 7.24 – 7.19 (m, 1H), 4.59 (t, *J* = 5.1 Hz, 1H), 4.01 – 3.91 (m, 1H), 3.83 – 3.73 (m, 1H), 2.26 (s, 3H), 2.20 – 2.09 (m, 2H); ^{13}C NMR (126 MHz, $CDCl_3$) δ 170.3, 138.6, 128.9, 128.3, 125.5, 124.9, 57.5, 40.5, 30.2, 23.6; HRMS m/z $[M + H]^+$ calculated for $C_{11}H_{13}N_4O$: 217.1084, found 217.1081.

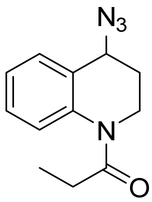

1-(4-Azido-3,4-dihydroquinolin-1(2H)-yl)propan-1-one (9b)

Yield: 85%, 19.6 mg. ^{1}H NMR (500 MHz, $CDCl_3$) δ 7.42 (s, 1H), 7.37–7.29 (m, 2H), 7.23–7.17 (m, 1H), 4.58 (t, *J* = 5.1 Hz, 1H), 3.99–3.91 (m, 1H), 3.82–3.74 (m, 1H), 2.53 (tt, *J* = 7.5, 3.9 Hz, 2H), 2.22–2.07 (m, 2H), 1.17 (t, *J* = 7.4 Hz, 3H); ^{13}C NMR (126 MHz, $CDCl_3$) δ 173.8, 138.5, 128.8, 128.3, 125.4, 124.9, 57.5, 40.5, 30.3, 28.3, 10.1; HRMS m/z $[M + H]^+$ calculated for $C_{12}H_{15}N_4O$: 231.1240, found 231.1244.

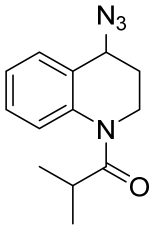

1-(4-Azido-3,4-dihydroquinolin-1(2H)-yl)-2-methylpropan-1-one (9c)

Yield: 65%, 15.9 mg. ^{1}H NMR (500 MHz, $CDCl_3$) δ7.39–7.35 (m, 1H), 7.35–7.27 (m, 2H), 7.25–7.19 (m, 1H), 4.59 (t, *J* = 5.3 Hz, 1H), 3.93 (dt, *J* = 12.3, 6.1 Hz, 1H), 3.85–3.75 (m, 1H), 3.12 (dt, *J* = 13.3, 6.7 Hz, 1H), 2.23–2.06 (m, 2H), 1.14 (dd, *J* = 6.6, 4.2 Hz, 6H); ^{13}C NMR (126 MHz, $CDCl_3$) δ 177.7, 138.7, 130.0, 128.8, 128.3, 125.6, 124.8, 57.6, 40.4, 31.4, 30.5, 20.2, 20.0; HRMS m/z $[M + H]^+$ calculated for $C_{13}H_{17}N_4O$: 245.1397, found 245.1399.

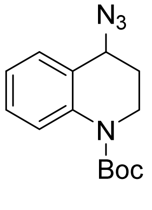

***Tert*-Butyl 4-azido-3,4-dihydroquinoline-1(2H)-carboxylate (9d)**

No reaction. ^{1}H NMR (500 MHz, $CDCl_3$) δ 7.85 (d, *J* = 8.2 Hz, 1H), 7.31–7.26 (m, 2H), 7.10–7.06 (m, 1H), 4.59 (t, *J* = 4.4 Hz, 1H), 4.06 (dt, *J* = 13.1, 4.8 Hz, 1H), 3.59 (ddd, *J* = 13.2, 8.9, 5.5 Hz, 1H), 2.09 (dt, *J* = 9.4, 4.6 Hz, 2H), 1.53 (s, 9H); ^{13}C NMR (126

MHz, $CDCl_3$) δ 153.7, 138.3, 128.9, 128.8, 125.8, 124.1, 123.2, 81.6, 57.7, 40.95, 29.2, 28.5; HRMS m/z $[M + H]^+$ calculated for $C_{13}H_{19}N_4O_2$: 275.1503, found 2275.1502.

Deuterated control experiment of indoline-based azide

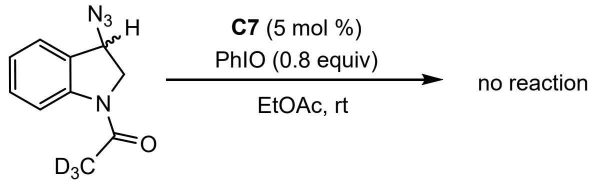

[D]-3a

To a solution of [D]-**3a** (0.1 mmol, 1.0 equiv) in ethyl acetate (1.0 mL) was added **C7** (0.005 mmol, 0.05 equiv) at r.t. Then PhIO (0.08 mmol, 0.8 equiv) was added as 8 portions in 30-min intervals over 3.5 h. No oxidative conversion was observed after 24 h.

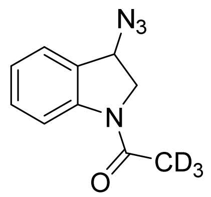

1-(3-Azidoindolin-1-yl)ethan-1-one-2,2,2-d3 ([D]-3a)

^1H NMR (500 MHz, $CDCl_3$) δ 8.27 (d, J = 8.4 Hz, 1H), 7.42–7.36 (m, 2H), 7.14 (td, J = 7.5, 0.9 Hz, 1H), 5.01 (dd, J = 7.8, 2.1 Hz, 1H), 4.17 (dd, J = 11.6, 8.0 Hz, 1H), 3.95 (dd, J = 11.6, 2.5 Hz, 1H); ^{13}C NMR (126 MHz, $CDCl_3$) δ 168.6, 143.1, 131.0, 127.9, 125.1, 124.2, 117.8, 60.0, 55.5, 23.9; HRMS m/z $[M + H]^+$ calculated for $C_{10}H_8D_3N_4O$: 206.1116, found 206.1122.

Computational Details

All DFT calculations were performed with Gaussian 09 program package.[8] Geometry optimizations were carried out at (U)M06[9]/BSI level in the gas phase [BSI denotes the basis set combination of LanL2DZ[10] for Mn atom, 6-31G(d,p) for the H atom of manganese-hydroxyl group and 6-31G(d) for other atoms]. The harmonic vibrational frequency was calculated at the same theoretical level for all of the stationary points to confirm them as the transition structures, and to obtain the thermochemical corrections and Gibbs free energies. The single-point energies of all optimized transition states were refined at the (U)M06/def2-TZVP[11] level of theory with the SMD model[12] in ethylethanoate solvent. Independent Gradient Model (IGM) analysis[13] was implemented by the Multiwfn (version 3.7) program.[14] CYLview[15] and the VMD[16] visualization software were used to show the 3D optimized transition states.

Supplementary table 1. The relative Gibbs free energies of possible isomers of the catalyst at different spin states. All energies are given in kcal/mol.

**Cat**	^{oss}**Cat-1**	^{css}**Cat-1**	^{3}**Cat-1**	^{5}**Cat-1**	^{oss}**Cat-2**	^{css}**Cat-2**	^{3}**Cat-2**	^{5}**Cat-2**
**$\Delta\Delta G^{\neq}$**	25.3	26.8	0.0	5.1	25.7	26.4	2.2	8.4
**Cat**	^{oss}**Cat-3**	^{css}**Cat-3**	^{3}**Cat-3**	^{5}**Cat-3**	^{oss}**Cat-4**	^{css}**Cat-4**	^{3}**Cat-4**	^{5}**Cat-4**
**$\Delta\Delta G^{\neq}$**	28.1	28.8	3.1	7.7	28.3	28.8	4.4	9.1

OSS represents the open shell singlet state, and *CSS* represents the close shell singlet state.

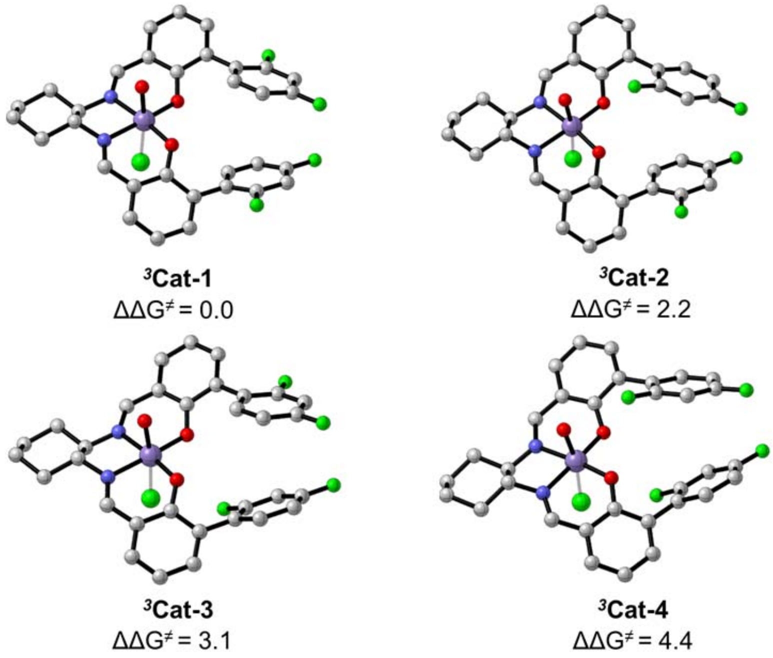

Supplementary figure 8. The relative Gibbs free energies of the optimized possible isomers of the catalyst at triplet spin state**.** All energies are given in kcal/mol.

Four conformers, based on the rotation of fluorine-substituted phenyl groups, for the catalyst were calculated. The conformer **Cat-1** is at least 2.2 kcal/mol more stable than other three conformers. Therefore, the following calculations in this work were performed on the basis of conformer **Cat-1**.

Supplementary table 2. The relative Gibbs free energies of the hydrogen atom transfer transition states at different spin states. All energies are given in kcal/mol.

TSs	${}^{oss}\mathbf{TS}_R$	${}^{css}\mathbf{TS}_R$	${}^{3}\mathbf{TS}_R$	${}^{5}\mathbf{TS}_R$	${}^{oss}\mathbf{TS}_S$	${}^{css}\mathbf{TS}_S$	${}^{3}\mathbf{TS}_S$	${}^{5}\mathbf{TS}_S$
**ΔΔG≠**	45.0	44.9	0.0	8.1	47.8	47.3	1.8	7.6

OSS represents the open shell singlet state, and *CSS* represents the close shell singlet state.

Supplementary table 3. Zero-point correction (*ZPE*), thermal correction to enthalpy (*TCH*), thermal correction to Gibbs free energy (*TCG*), energies (*E*), enthalpies (*H*), Gibbs free energies (*G*) (in Hartree) and the Imaginary Frequencies (IF, cm^{-1}) of the Optimized Structures calculated at the (U)M06/def2-TZVP-SMD(ethylethanoate)//(U)M06/BSI level of theory.

Structures	*ZPE* (1)	*TCH* (2)	*TCG* (3)	*E* (4)	*H* (5)=(2)+(4)	*G* (6)=(3)+(4)	*IF*
^{oss}Cat-1	0.436999	0.537469	0.436999	-3579.144078	-3578.606609	-3578.707079	
^{oss}Cat-2	0.500994	0.536804	0.437393	-3579.143841	-3578.607037	-3578.706448	
^{oss}Cat-3	0.501335	0.536979	0.438110	-3579.140635	-3578.603656	-3578.702525	
^{oss}Cat-4	0.501123	0.536672	0.438521	-3579.140764	-3578.604092	-3578.702243	
^{css}Cat-1	0.502568	0.538043	0.439376	-3579.144035	-3578.605992	-3578.704659	
^{css}Cat-2	0.501861	0.537398	0.439100	-3579.144410	-3578.607012	-3578.70531	
^{css}Cat-3	0.501601	0.537178	0.438403	-3579.139919	-3578.602741	-3578.701516	
^{css}Cat-4	0.501597	0.537088	0.438833	-3579.140348	-3578.60326	-3578.701515	
^{3}Cat-1	0.498680	0.535271	0.432331	-3579.179683	-3578.644412	-3578.747352	
^{3}Cat-2	0.499607	0.535574	0.435126	-3579.178954	-3578.64338	-3578.743828	
^{3}Cat-3	0.499316	0.535505	0.434260	-3579.176688	-3578.641183	-3578.742428	
^{3}Cat-4	0.499494	0.535421	0.434991	-3579.175298	-3578.639877	-3578.740307	
^{5}Cat-1	0.498607	0.535135	0.431962	-3579.171085	-3578.63595	-3578.739123	
^{5}Cat-2	0.499990	0.535827	0.435559	-3579.169523	-3578.633696	-3578.733964	
^{5}Cat-3	0.499079	0.535406	0.433083	-3579.168192	-3578.632786	-3578.735109	
^{5}Cat-4	0.499135	0.535247	0.433622	-3579.166524	-3578.631277	-3578.732902	
^{oss}TS$_R$	0.808283	0.861603	0.727746	-4417.374867	-4416.513264	-4416.647121	1648.07*i*
^{css}TS$_R$	0.808362	0.861705	0.727332	-4417.374659	-4416.512954	-4416.647327	931.99*i*
^{3}TS$_R$	0.806043	0.860282	0.722277	-4417.441146	-4416.580864	-4416.718869	861.49*i*
^{5}TS$_R$	0.808536	0.862062	0.725913	-4417.431832	-4416.56977	-4416.705919	639.04*i*
^{oss}TS$_S$	0.806494	0.860462	0.724218	-4417.366936	-4416.506474	-4416.642718	1476.36*i*
^{css}TS$_S$	0.807631	0.861248	0.725806	-4417.369201	-4416.507953	-4416.643395	1238.58*i*
^{3}TS$_S$	0.806854	0.860872	0.723112	-4417.439182	-4416.57831	-4416.71607	599.11*i*
^{5}TS$_S$	0.807973	0.861876	0.723594	-4417.430432	-4416.568556	-4416.706838	345.23*i*

NMR Spectra

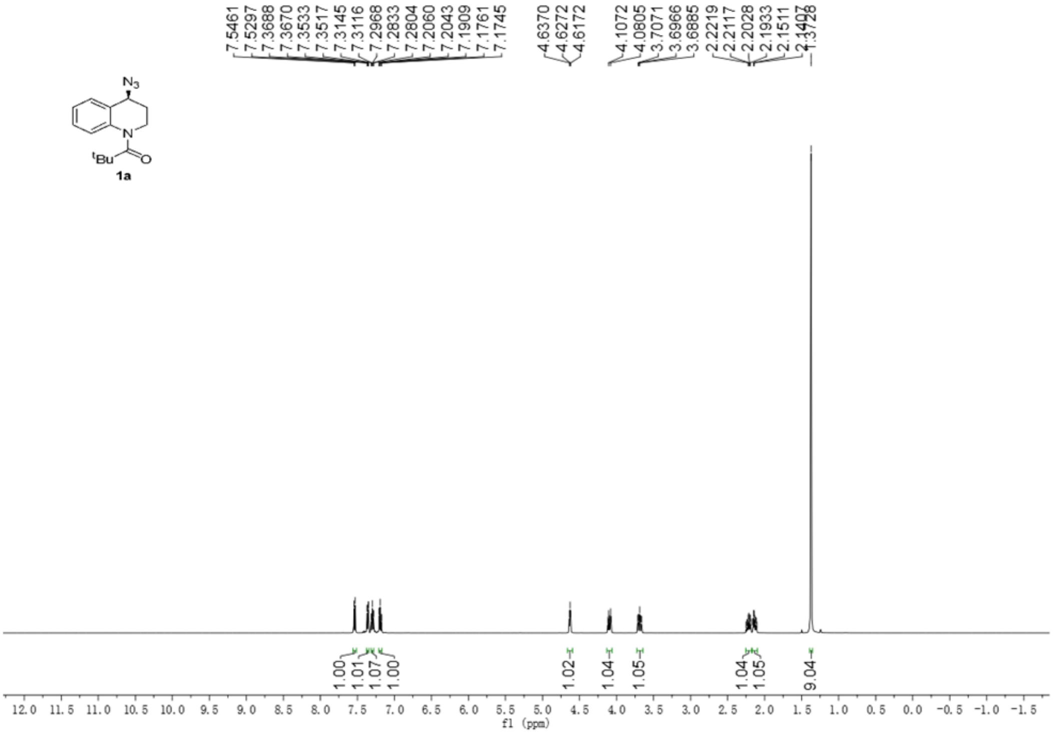

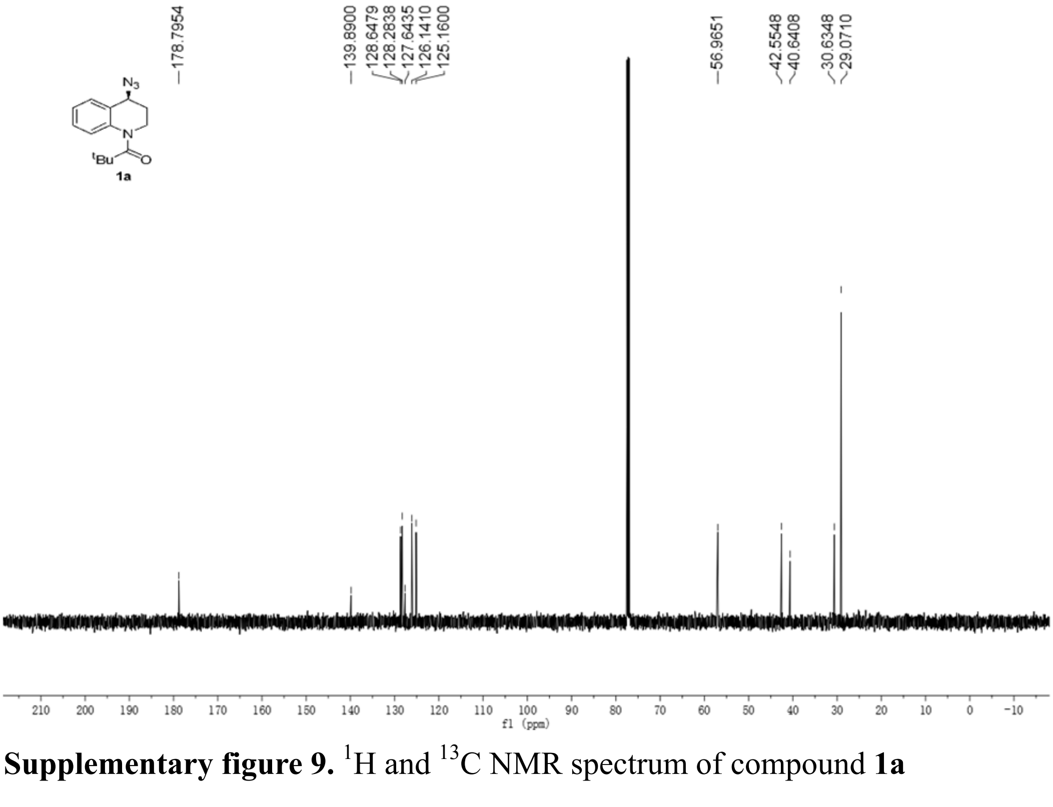

Supplementary figure 9. ^{1}H and ^{13}C NMR spectrum of compound **1a**

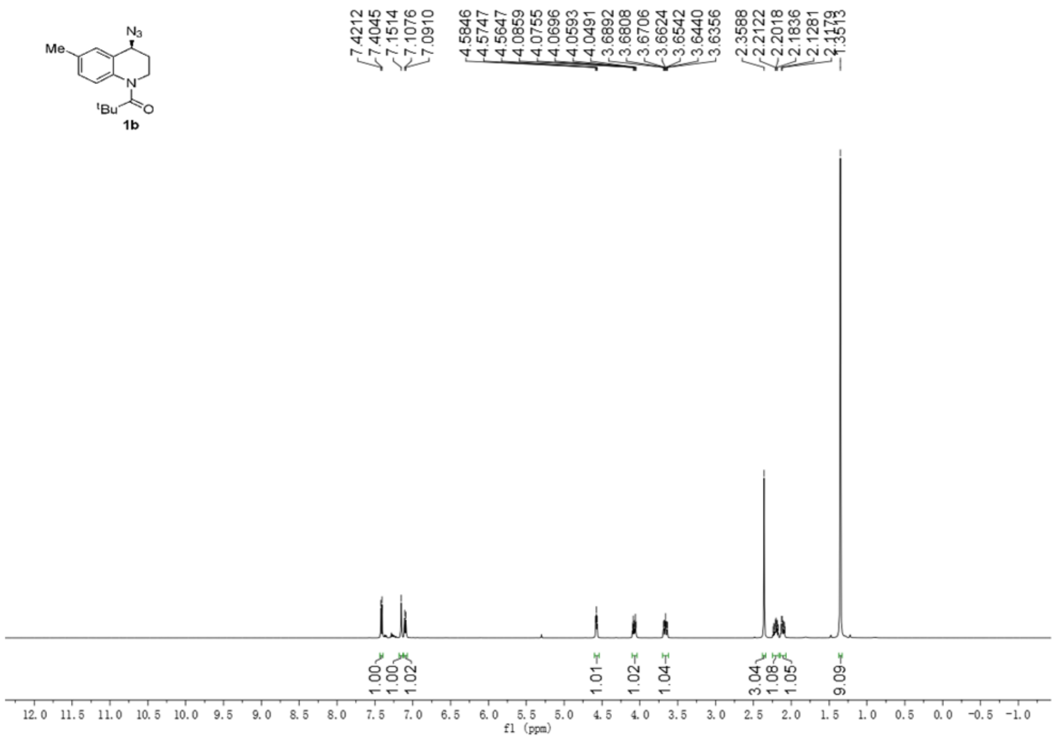

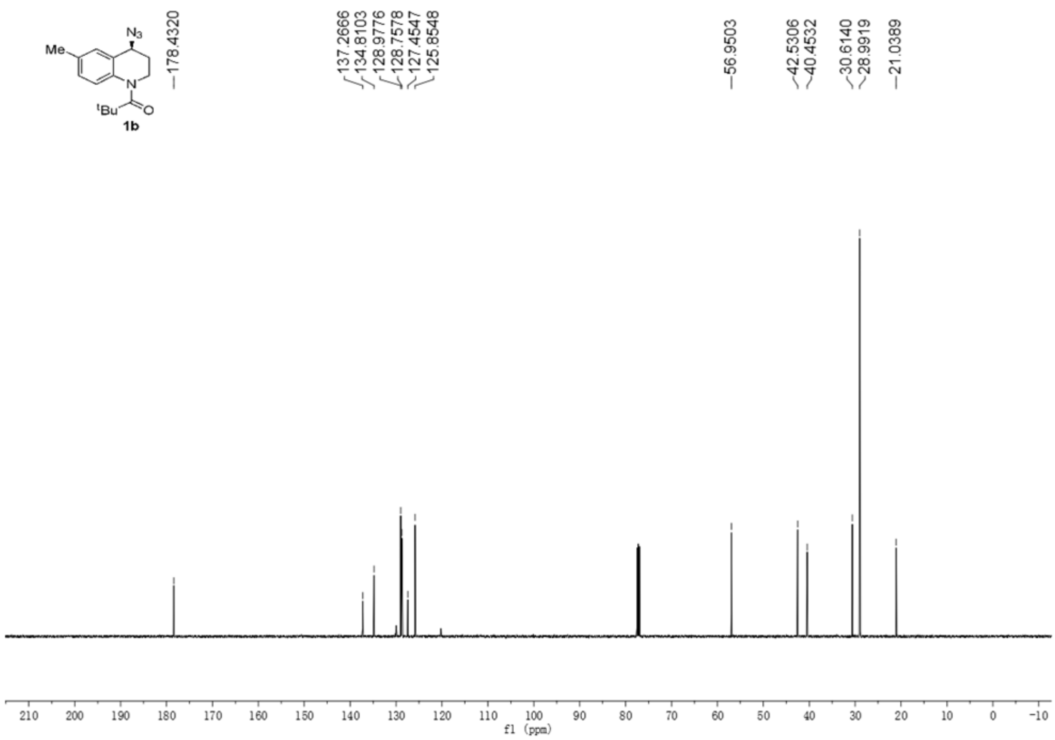

Supplementary figure 10. ^{1}H and ^{13}C NMR spectrum of compound **1b**

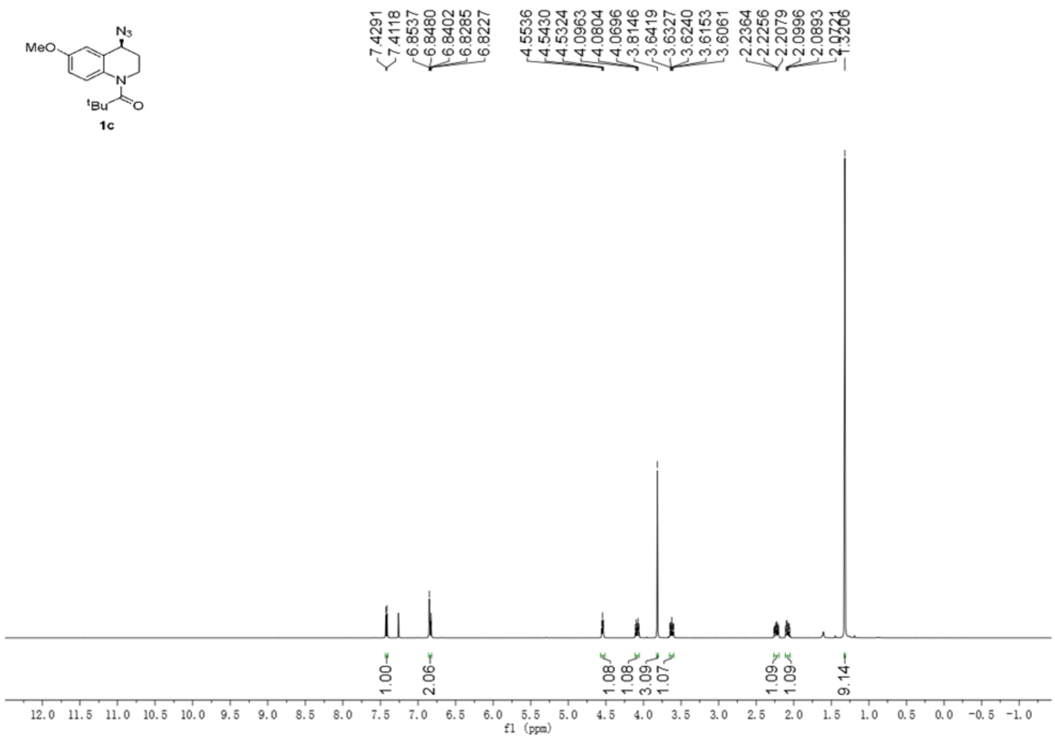

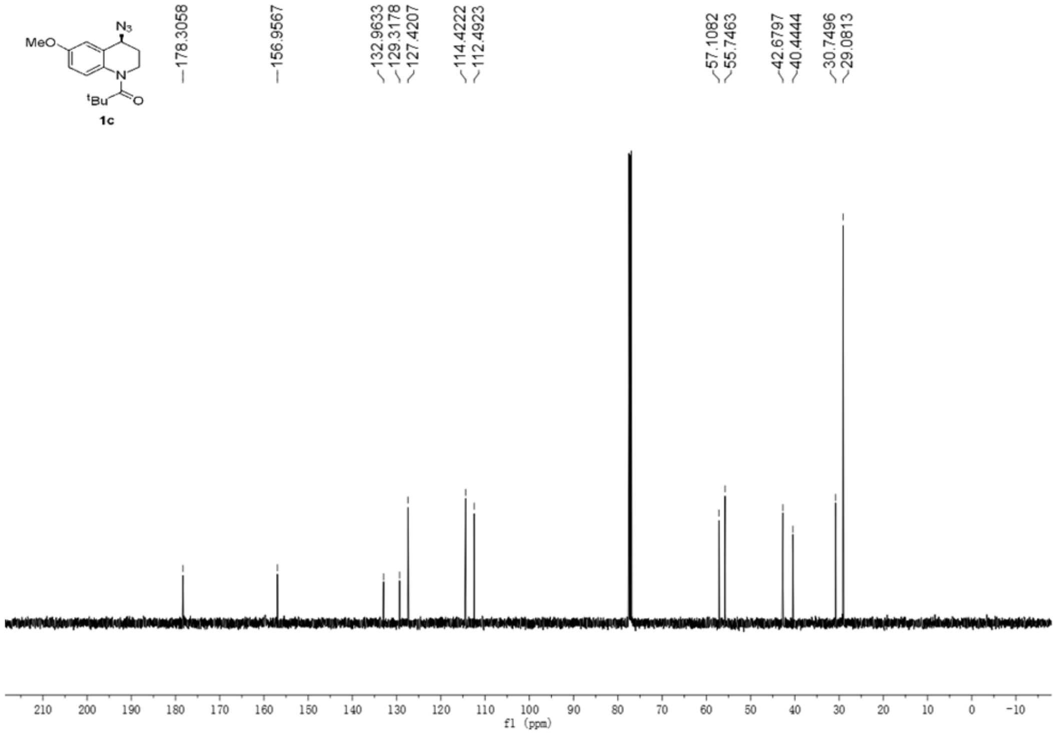

Supplementary figure 11. ^{1}H and ^{13}C NMR spectrum of compound **1c**

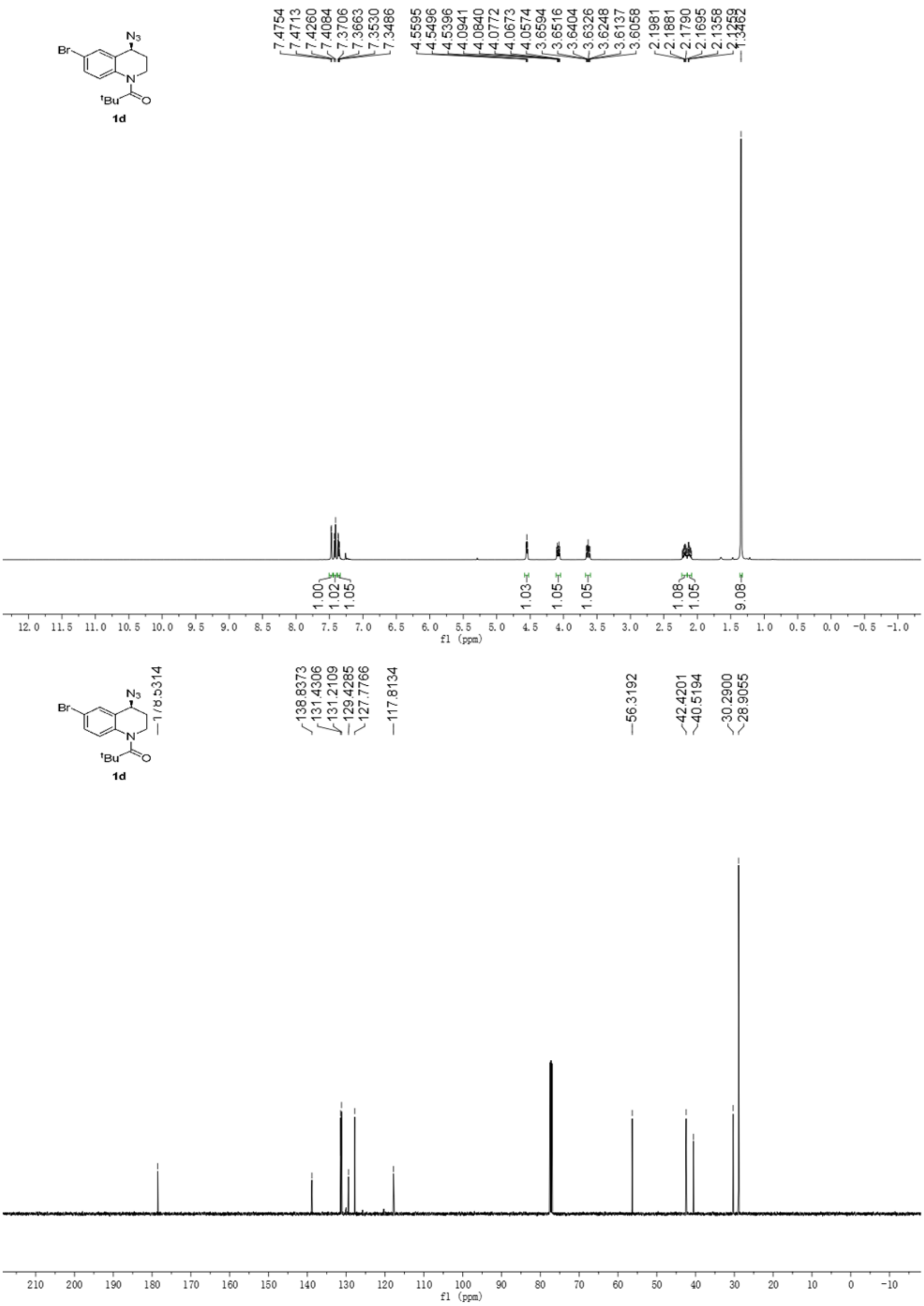

Supplementary figure 12. ^{1}H and ^{13}C NMR spectrum of compound **1d**

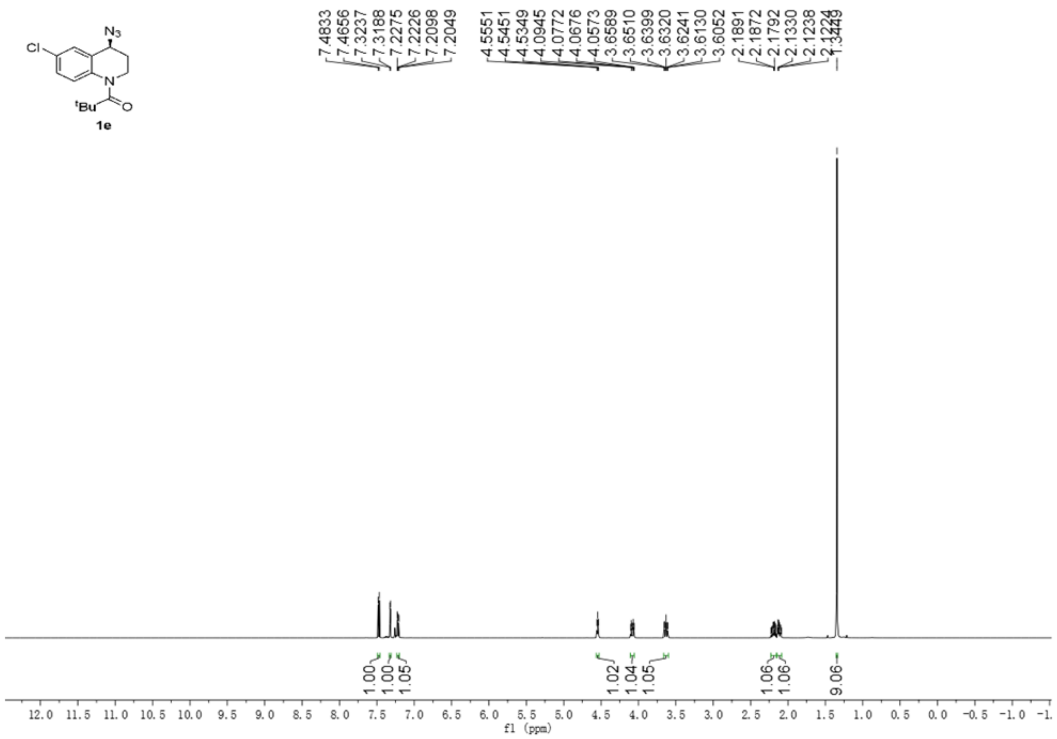

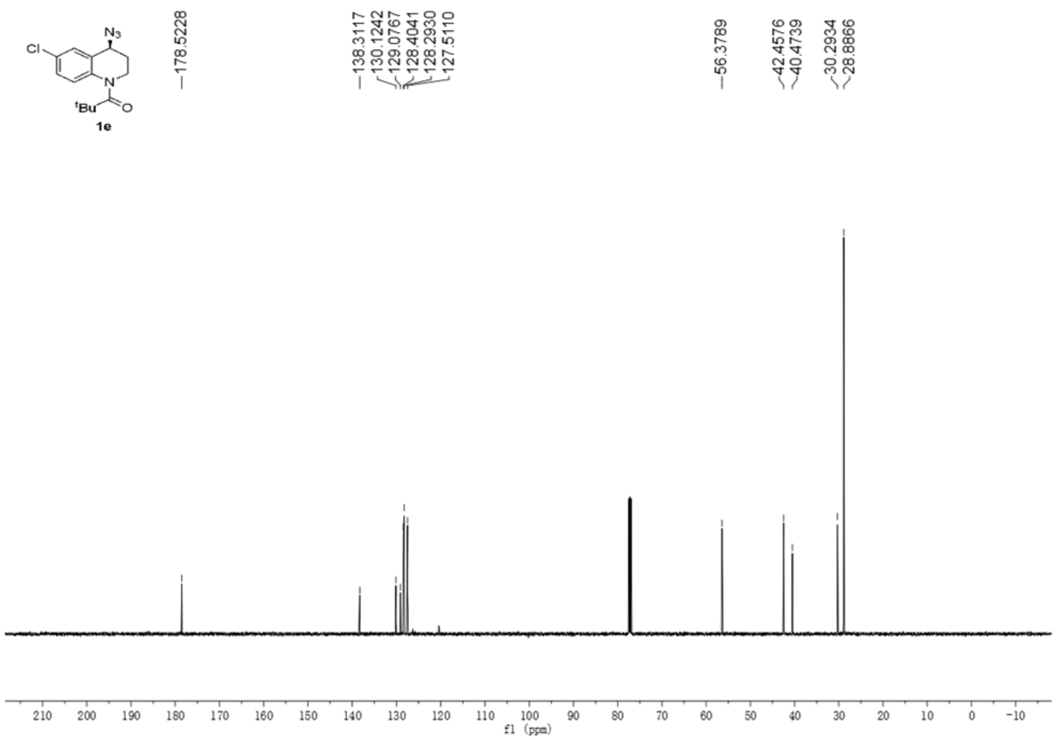

Supplementary figure 13. ^{1}H and ^{13}C NMR spectrum of compound **1e**

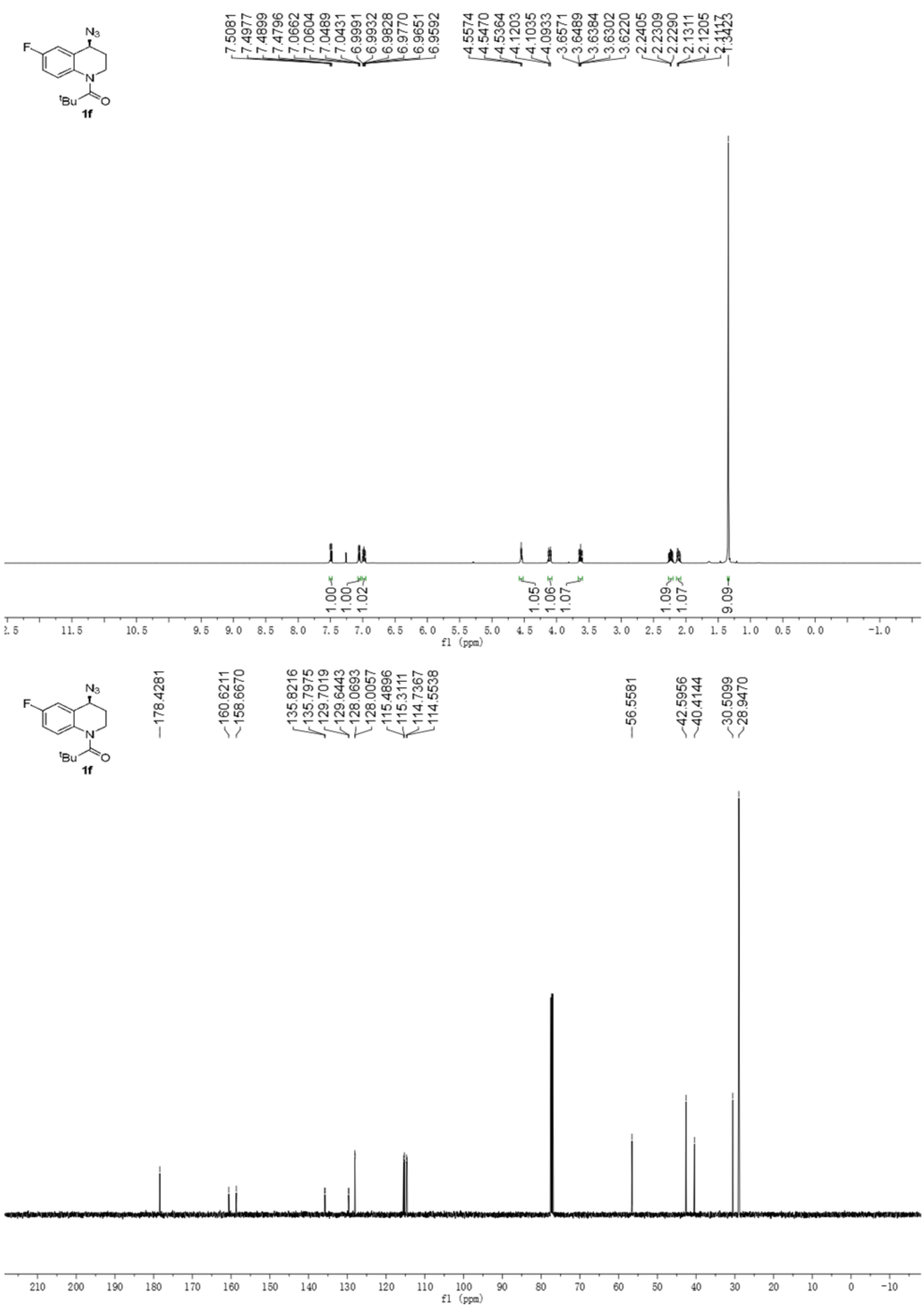

Supplementary figure 14. ^{1}H and ^{13}C NMR spectrum of compound **1f**

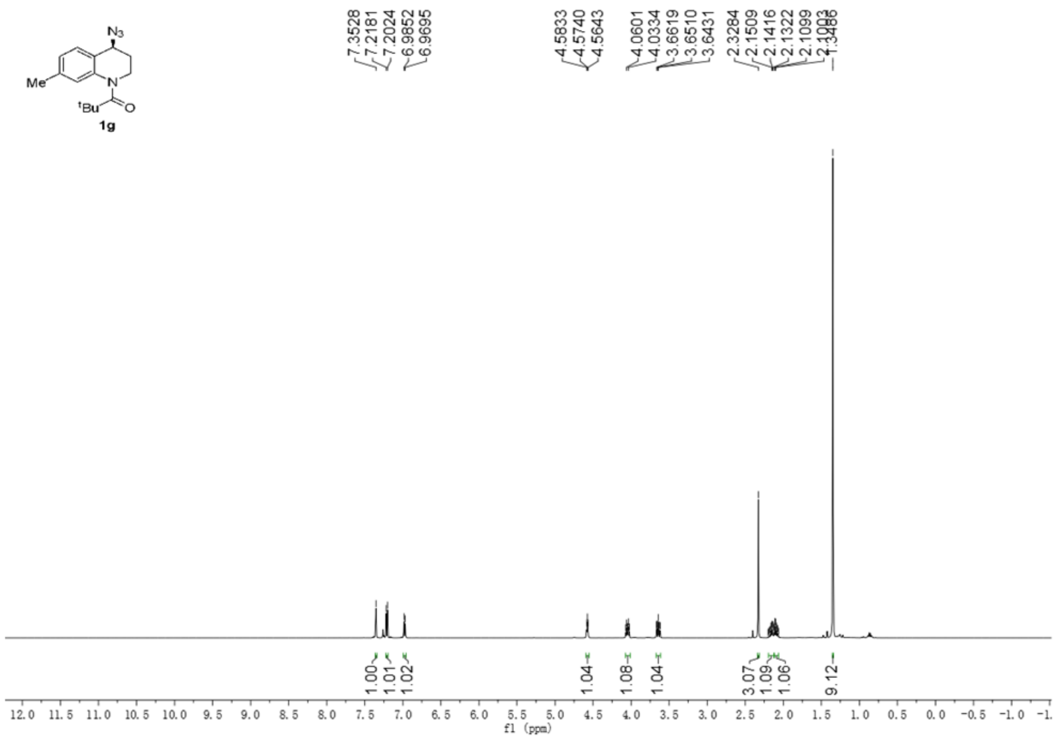

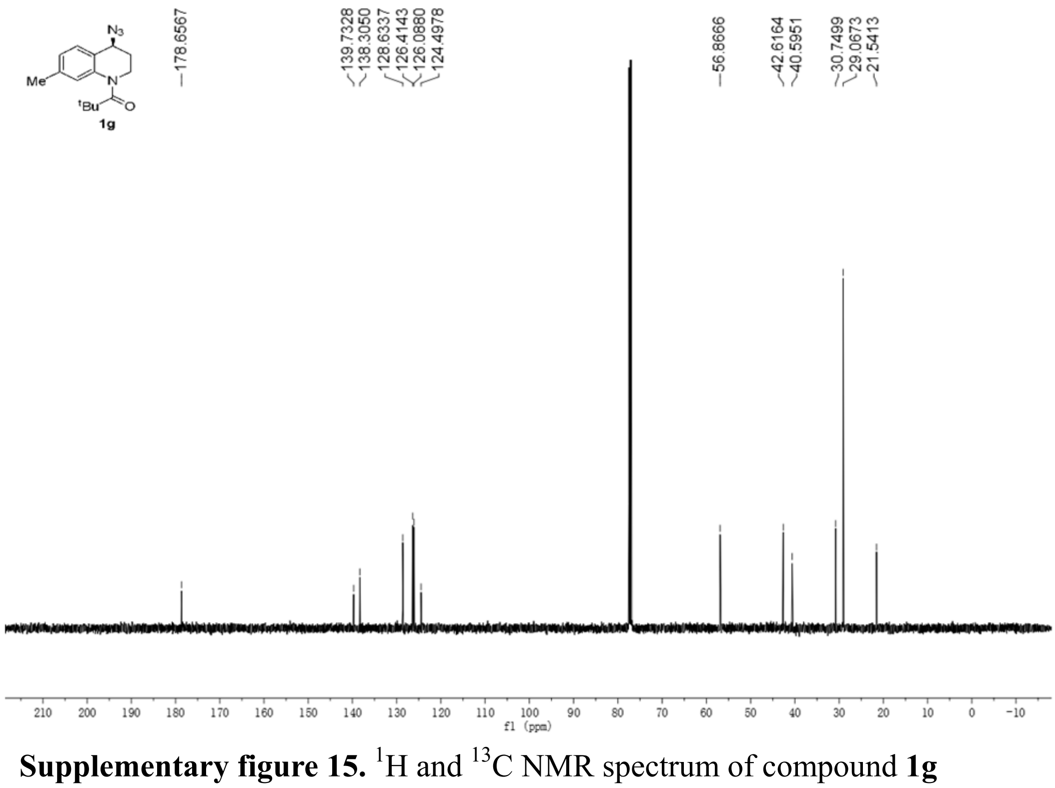

Supplementary figure 15. ^{1}H and ^{13}C NMR spectrum of compound **1g**

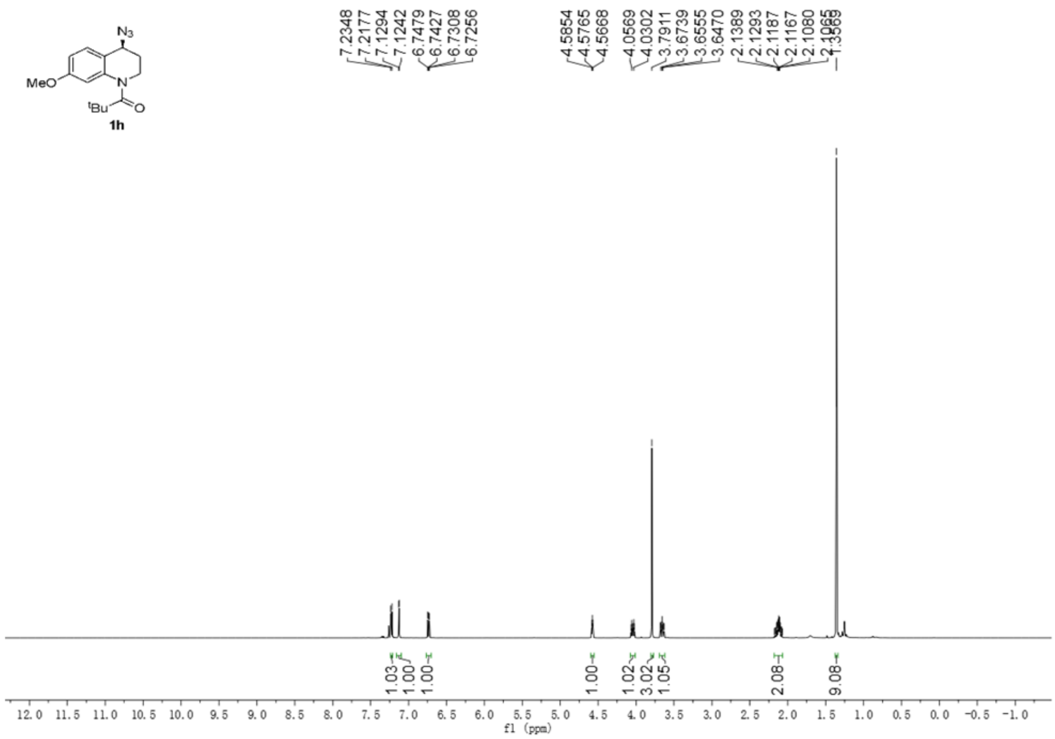

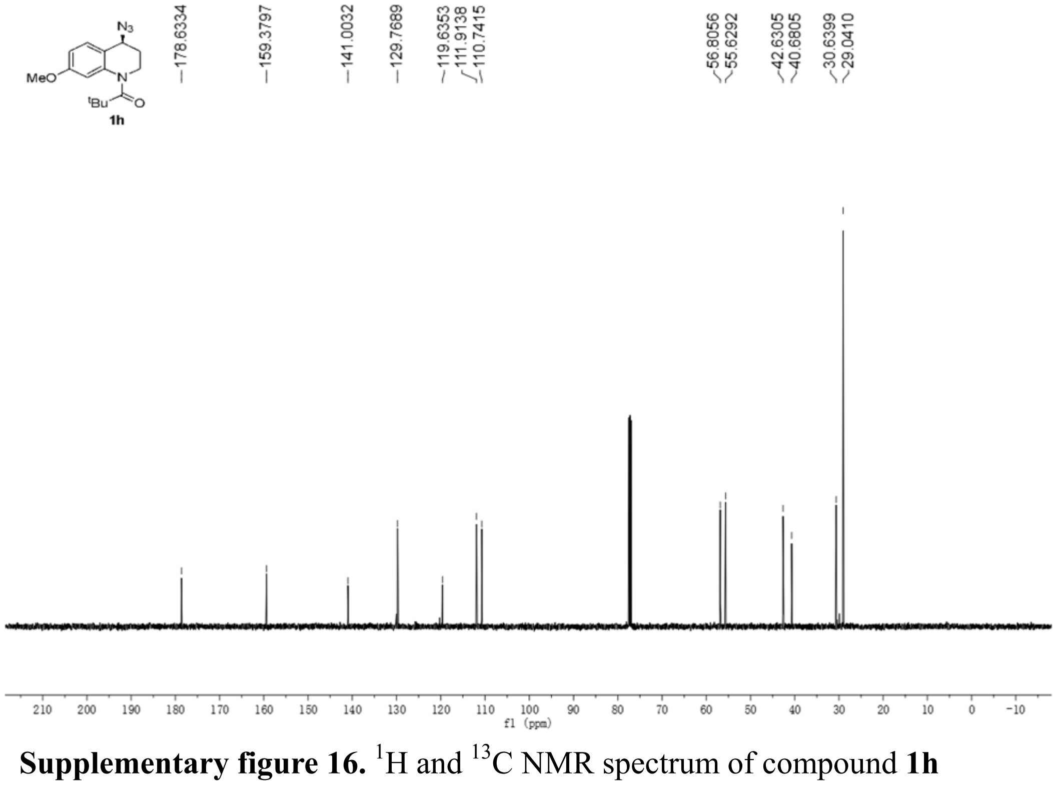

Supplementary figure 16. ^{1}H and ^{13}C NMR spectrum of compound **1h**

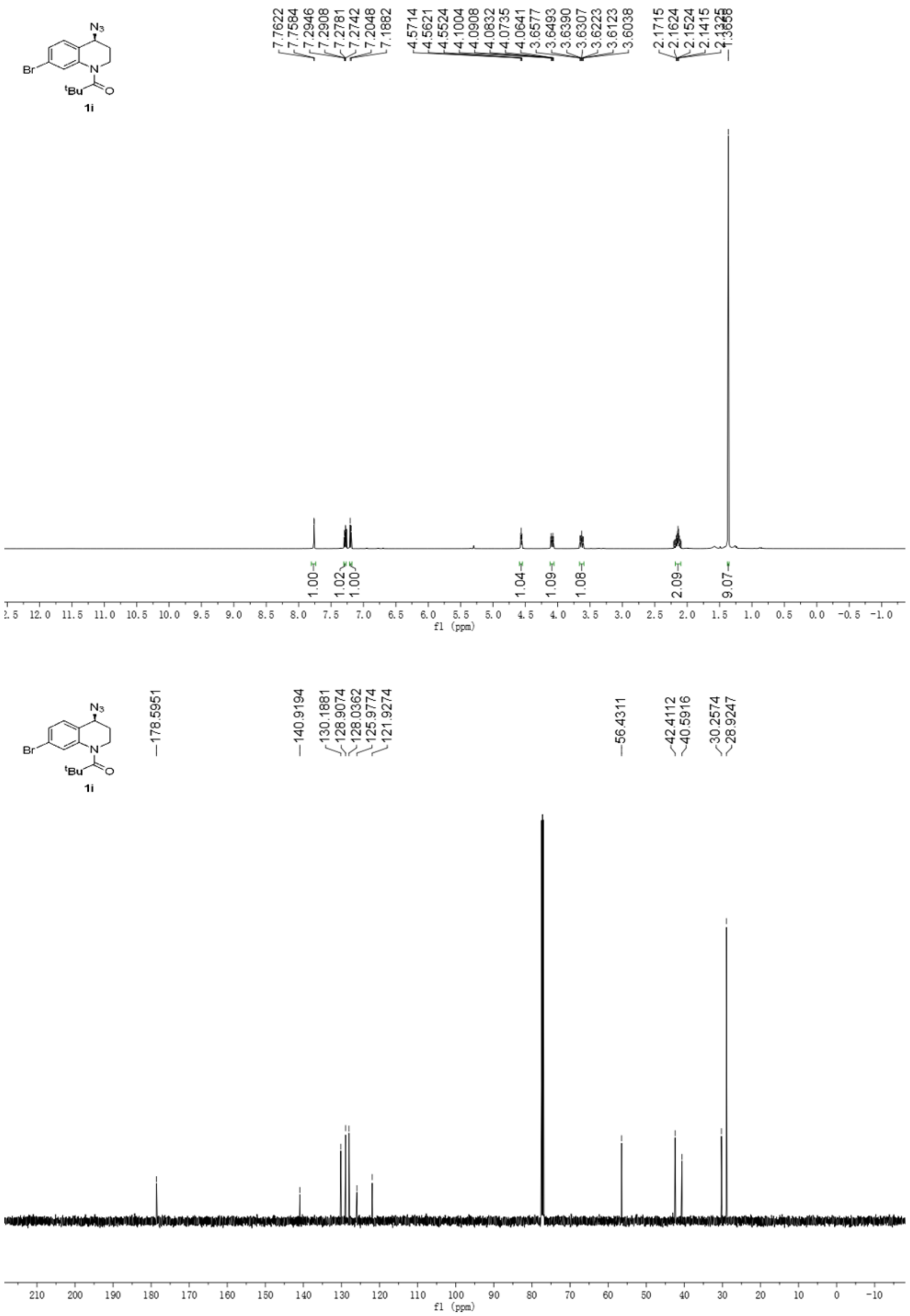

Supplementary figure 17. ^{1}H and ^{13}C NMR spectrum of compound **1i**

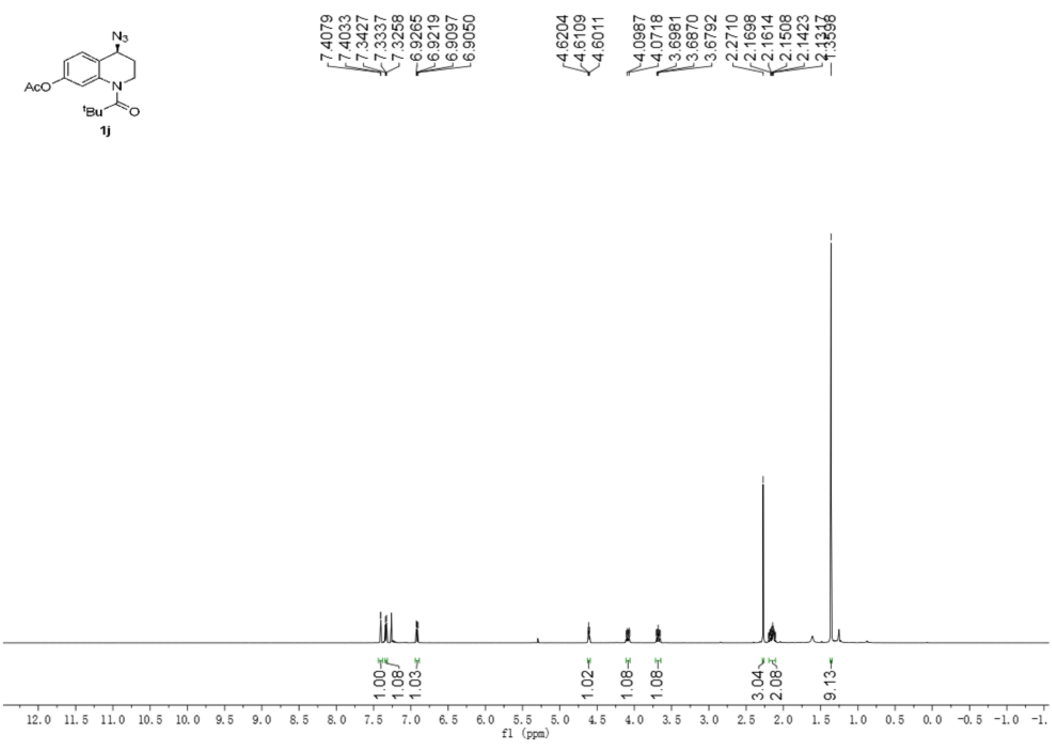

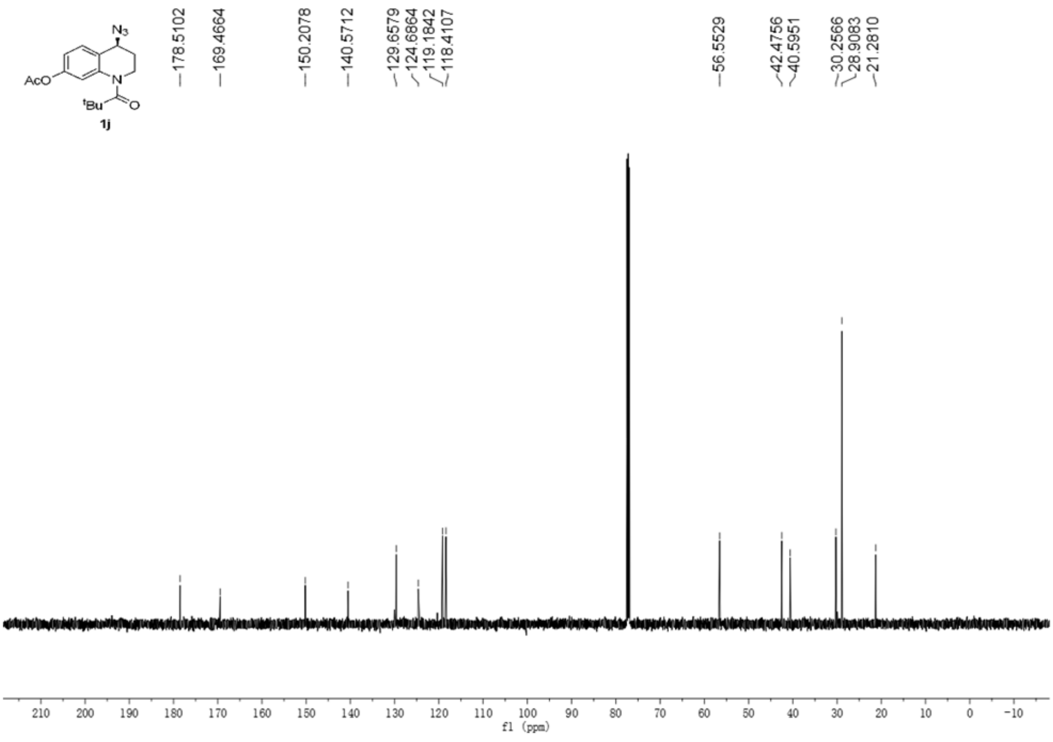

Supplementary figure 18. ^{1}H and ^{13}C NMR spectrum of compound **1j**

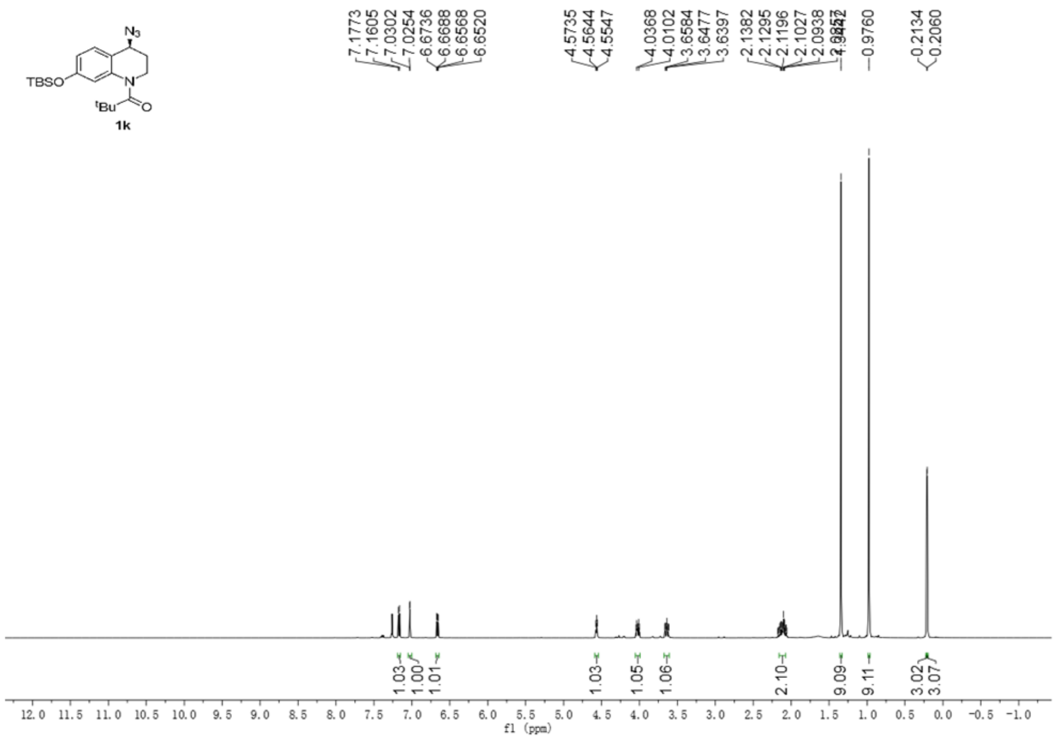

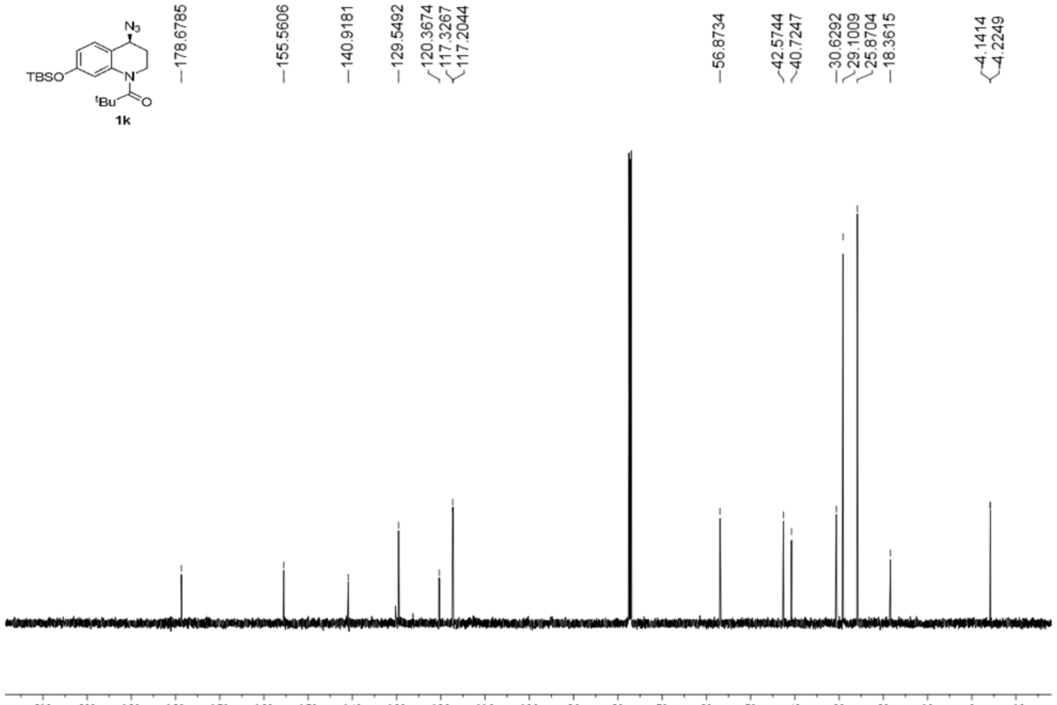

Supplementary figure 19. ^{1}H and ^{13}C NMR spectrum of compound **1k**

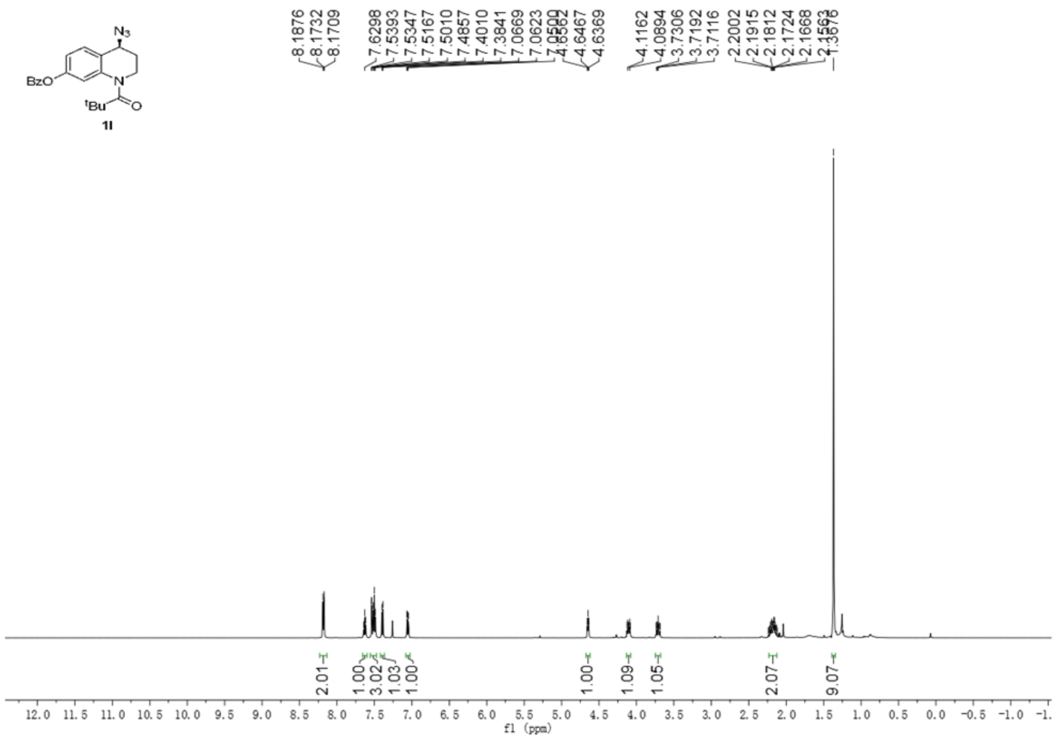

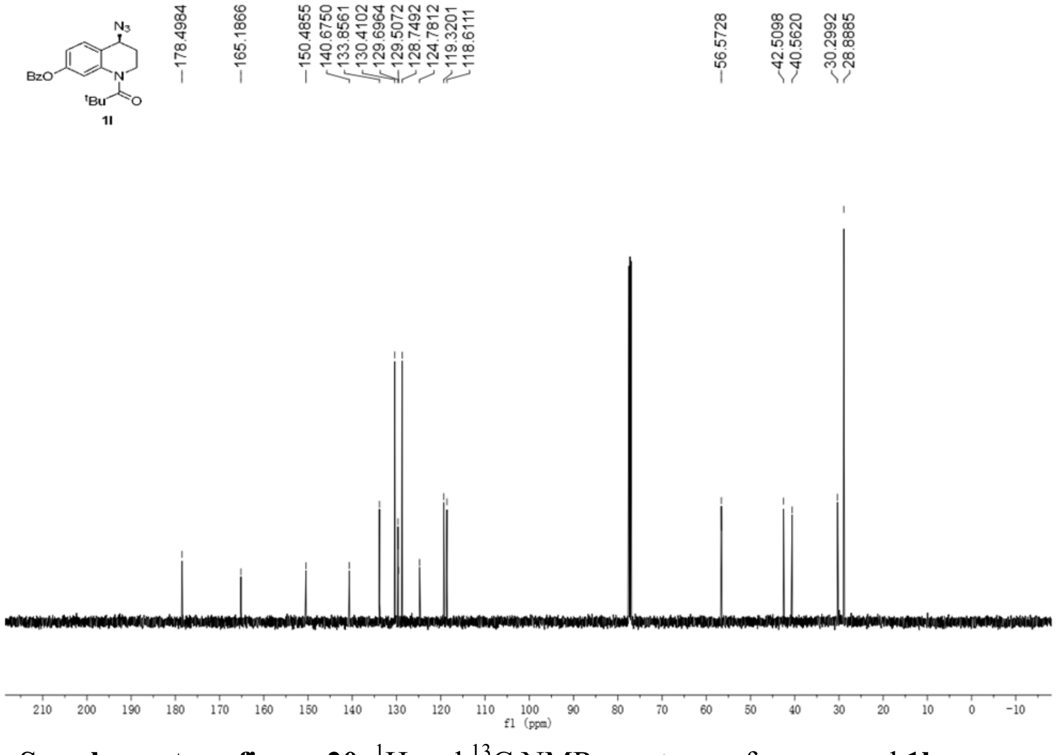

Supplementary figure 20. ^{1}H and ^{13}C NMR spectrum of compound **1l**

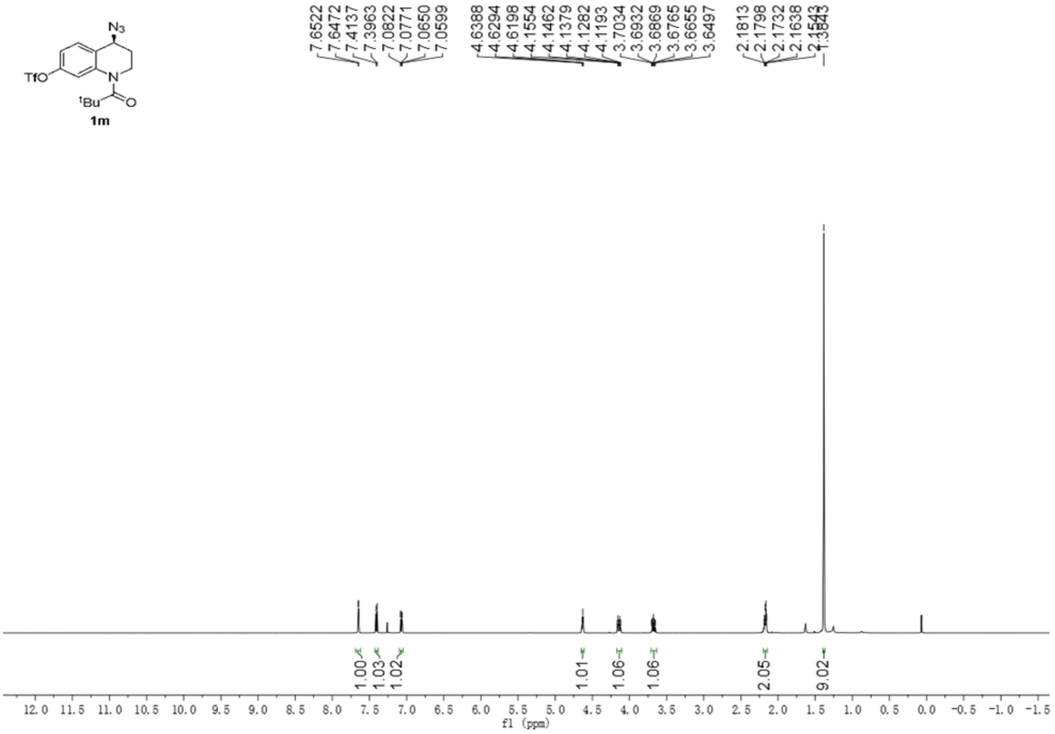

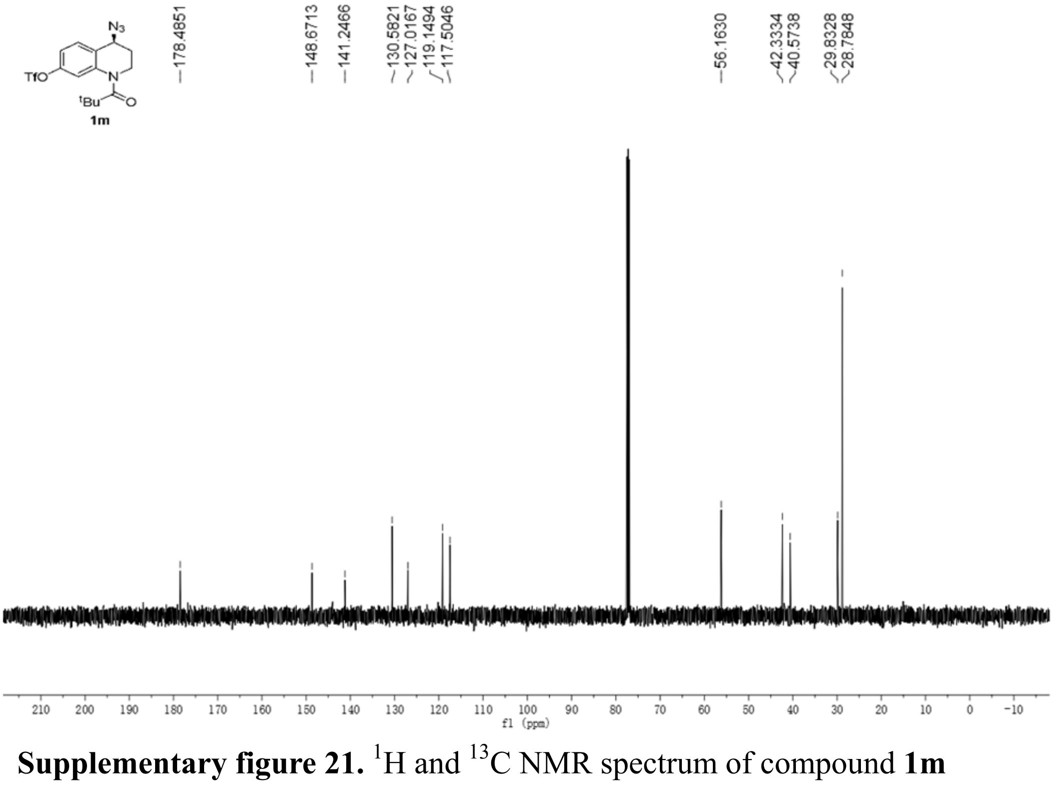

Supplementary figure 21. ^{1}H and ^{13}C NMR spectrum of compound **1m**

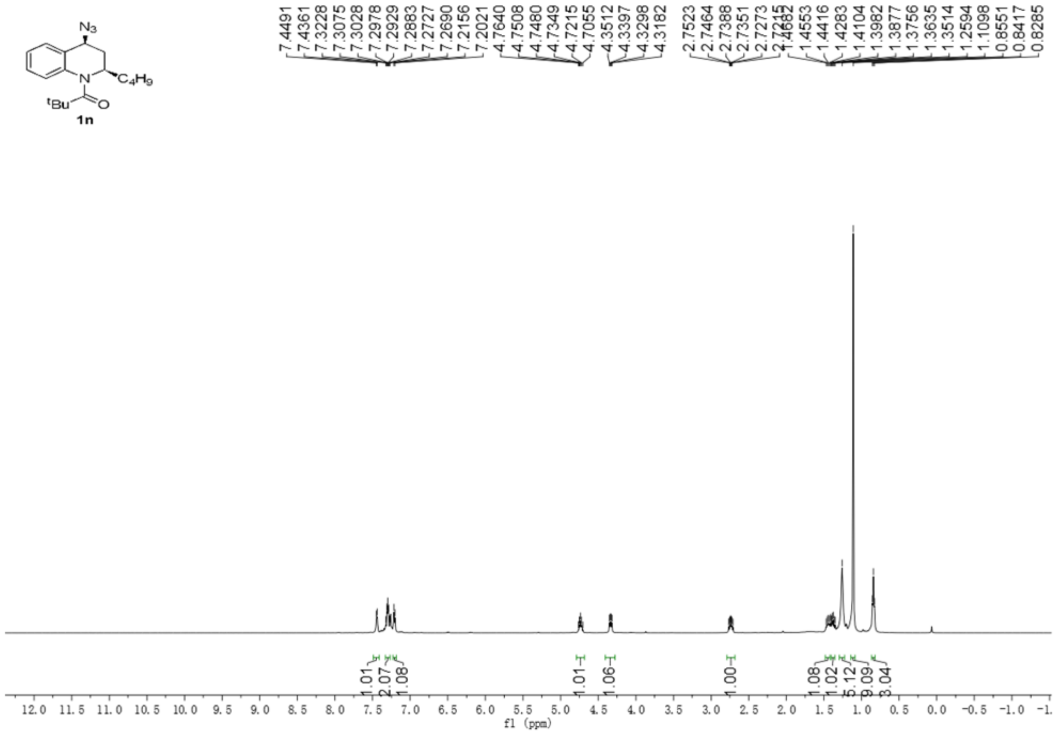

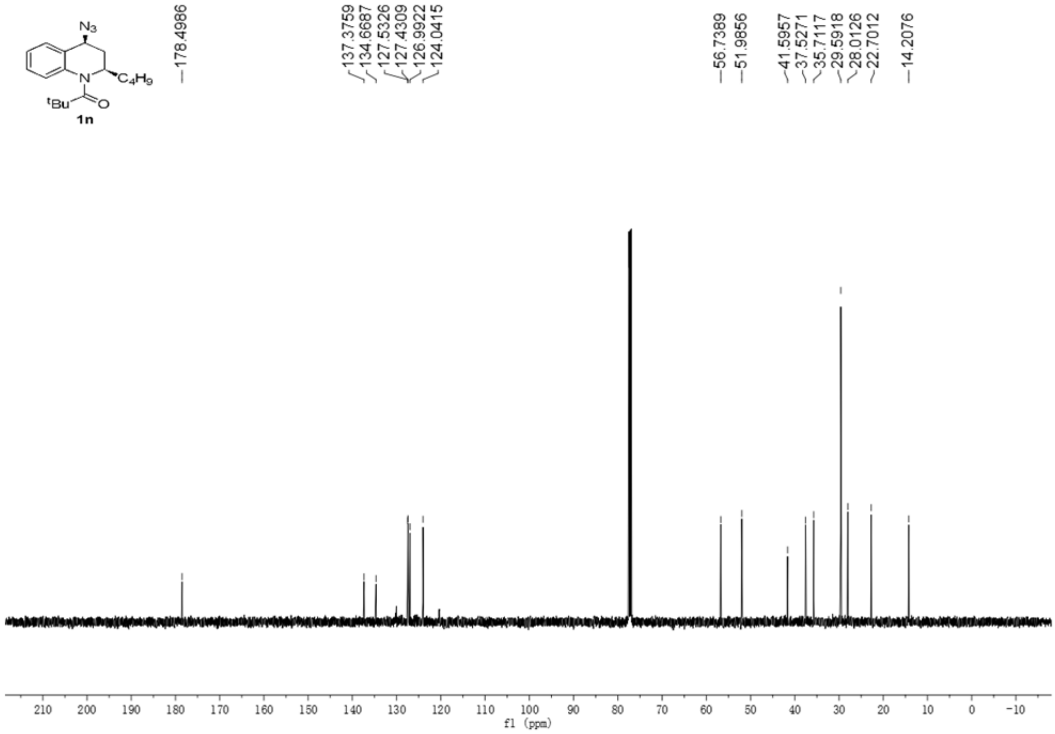

Supplementary figure 22. ^{1}H and ^{13}C NMR spectrum of compound **1n**

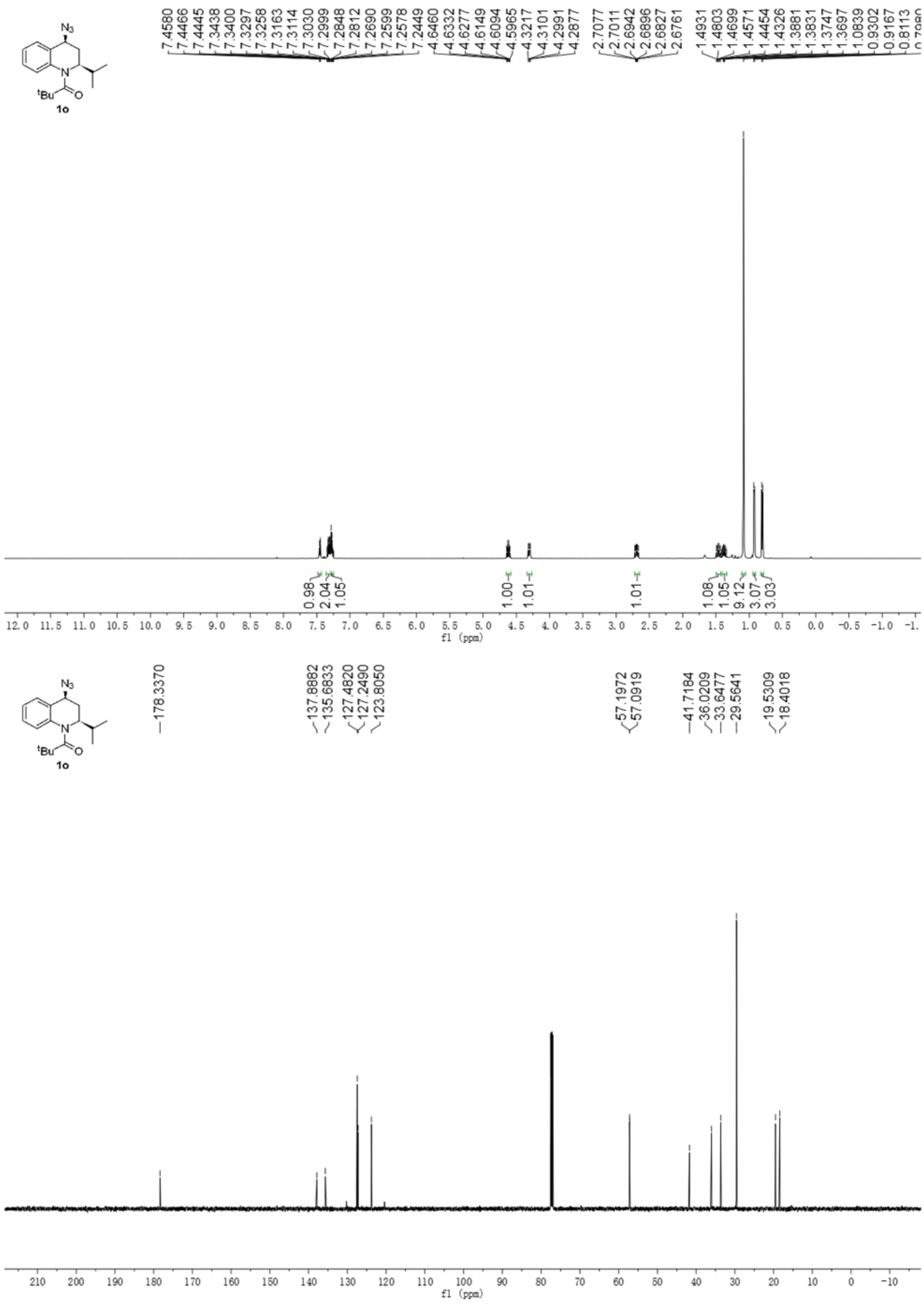

Supplementary figure 23. ^{1}H and ^{13}C NMR spectrum of compound **1o**

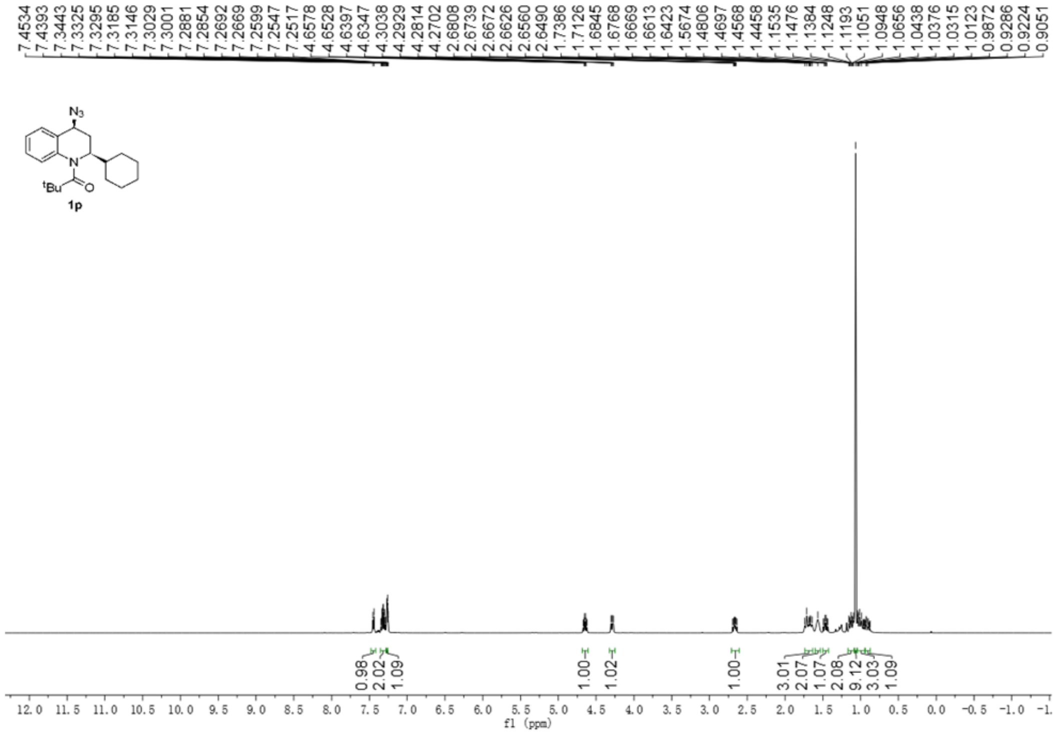

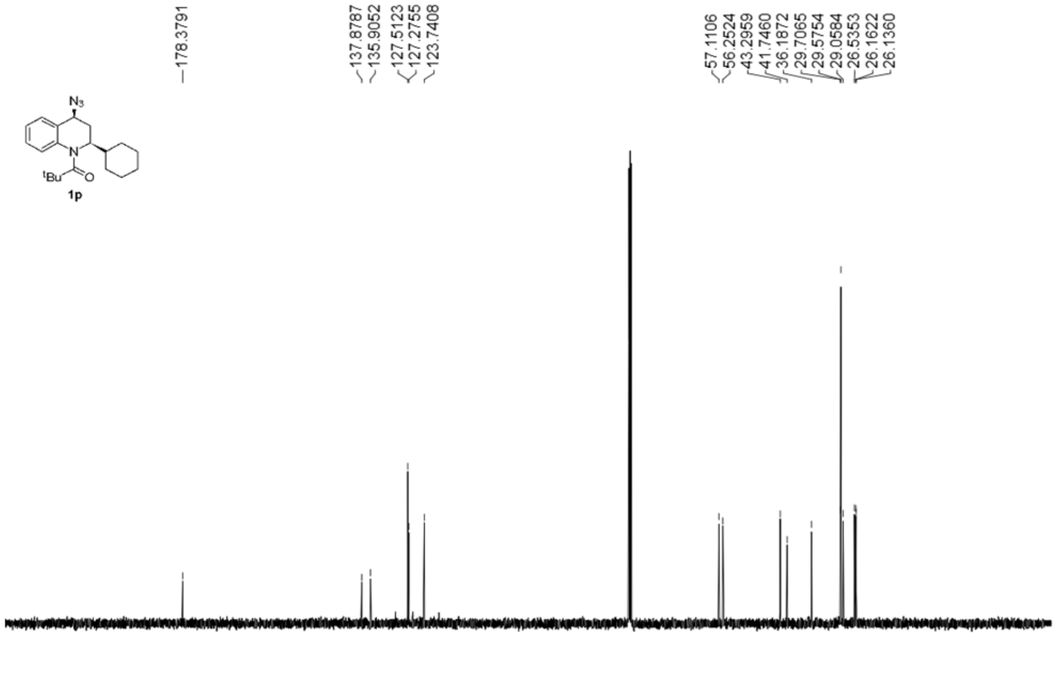

Supplementary figure 24. ^{1}H and ^{13}C NMR spectrum of compound **1p**

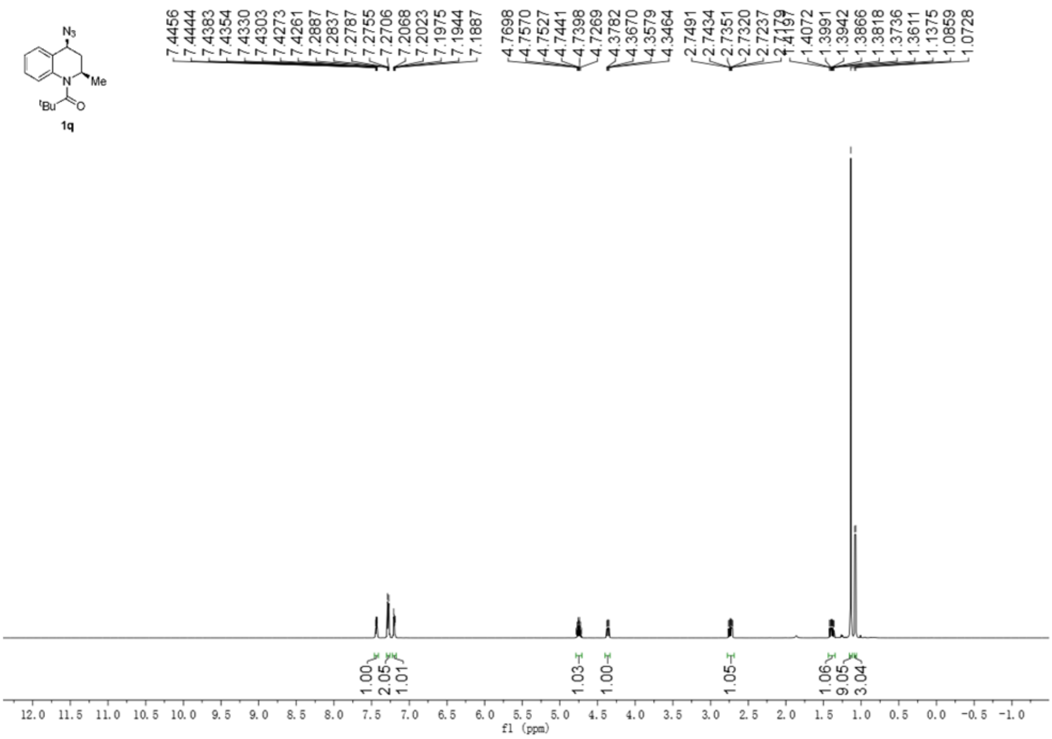

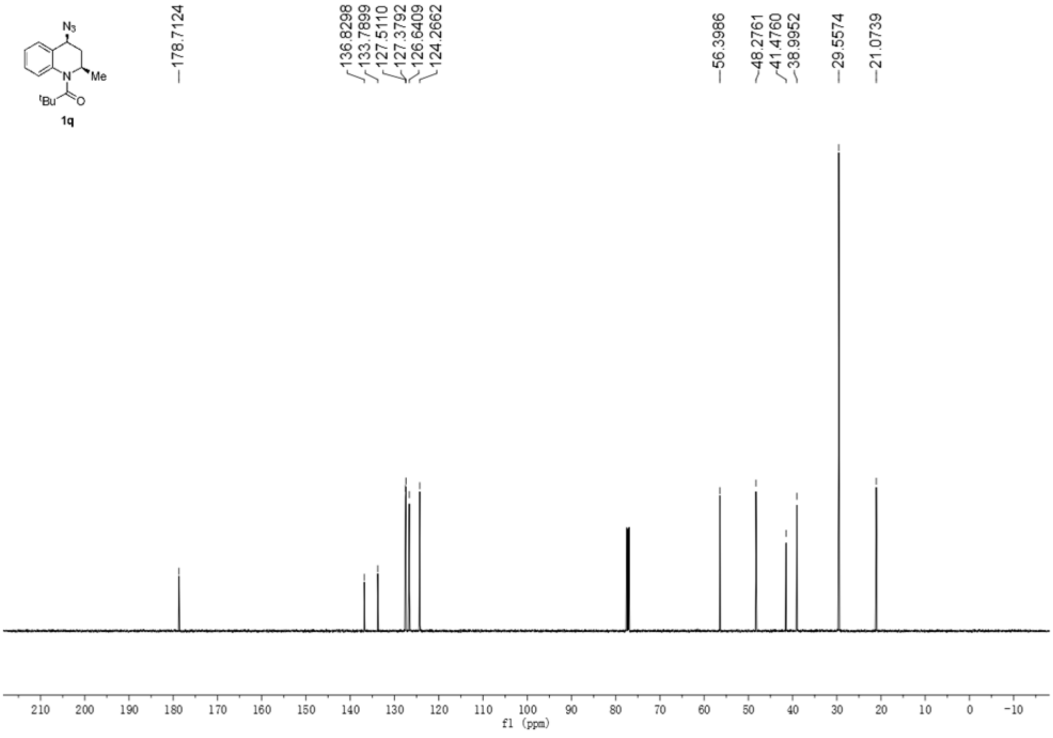

Supplementary figure 25. ^{1}H and ^{13}C NMR spectrum of compound **1q**

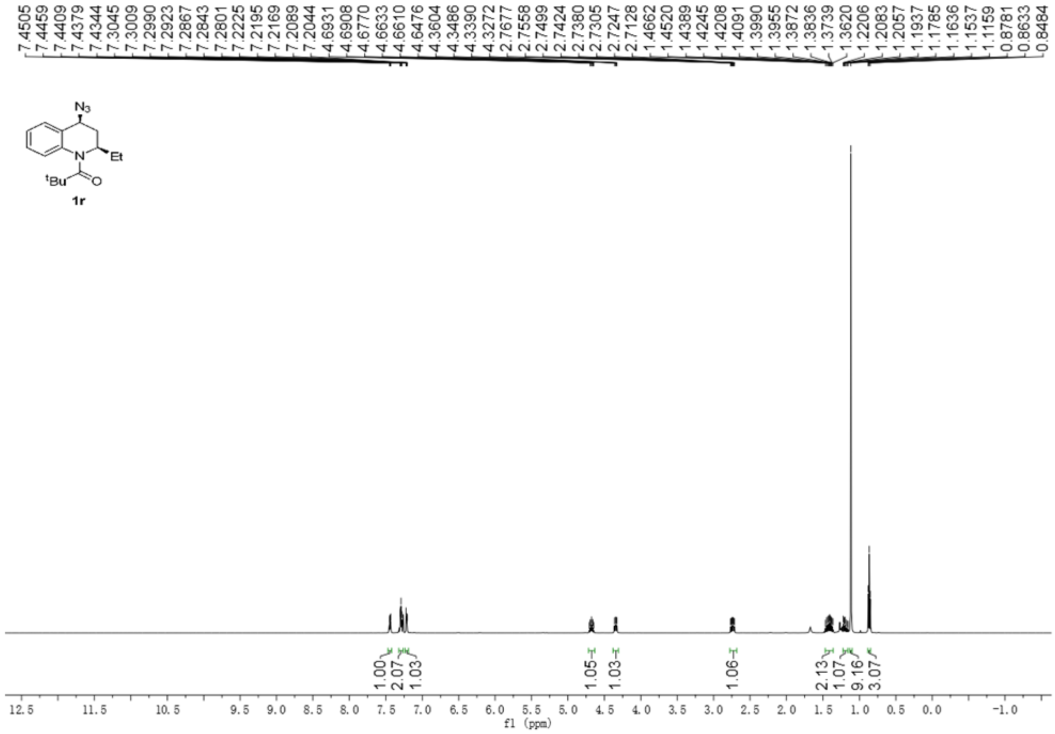

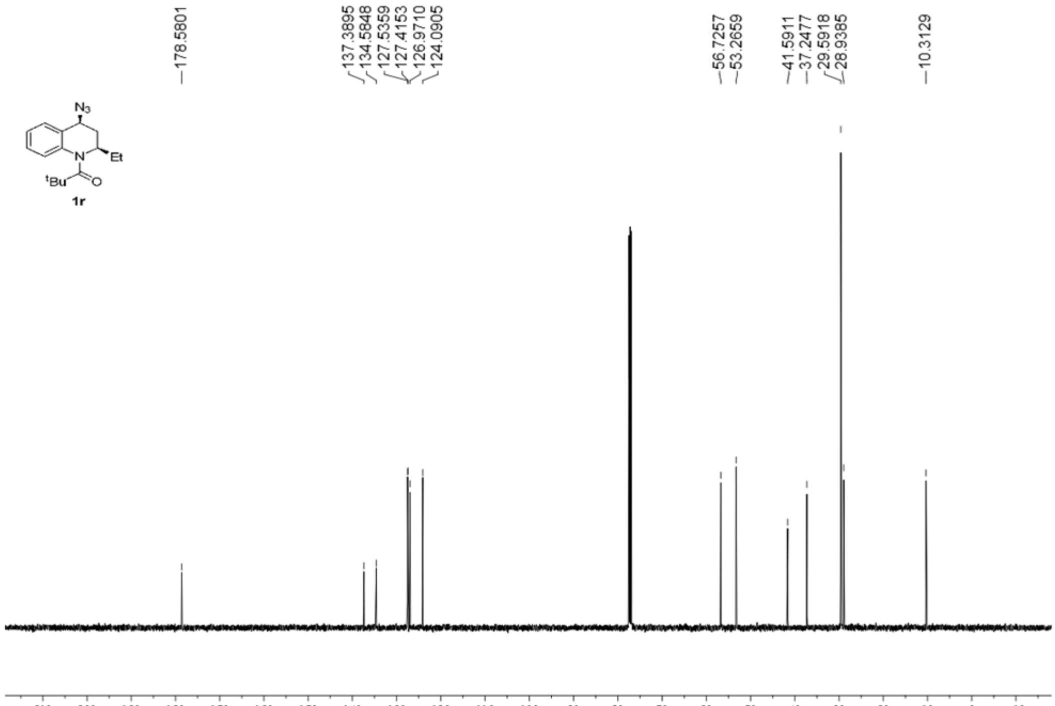

Supplementary figure 26. ^{1}H and ^{13}C NMR spectrum of compound **1r**

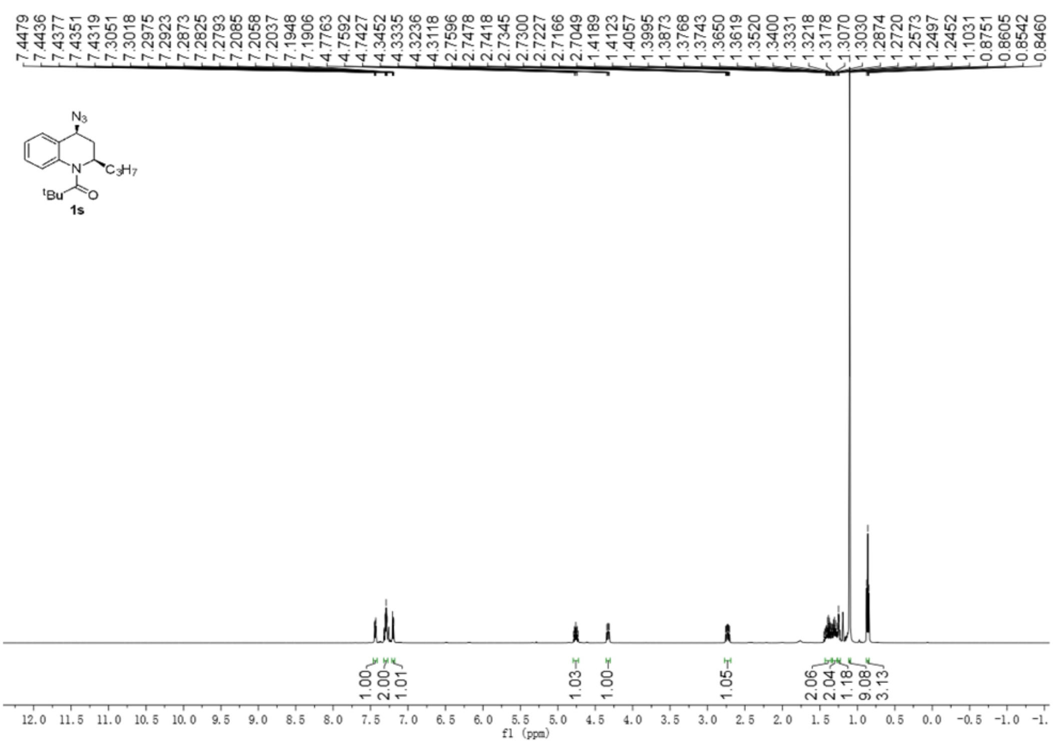

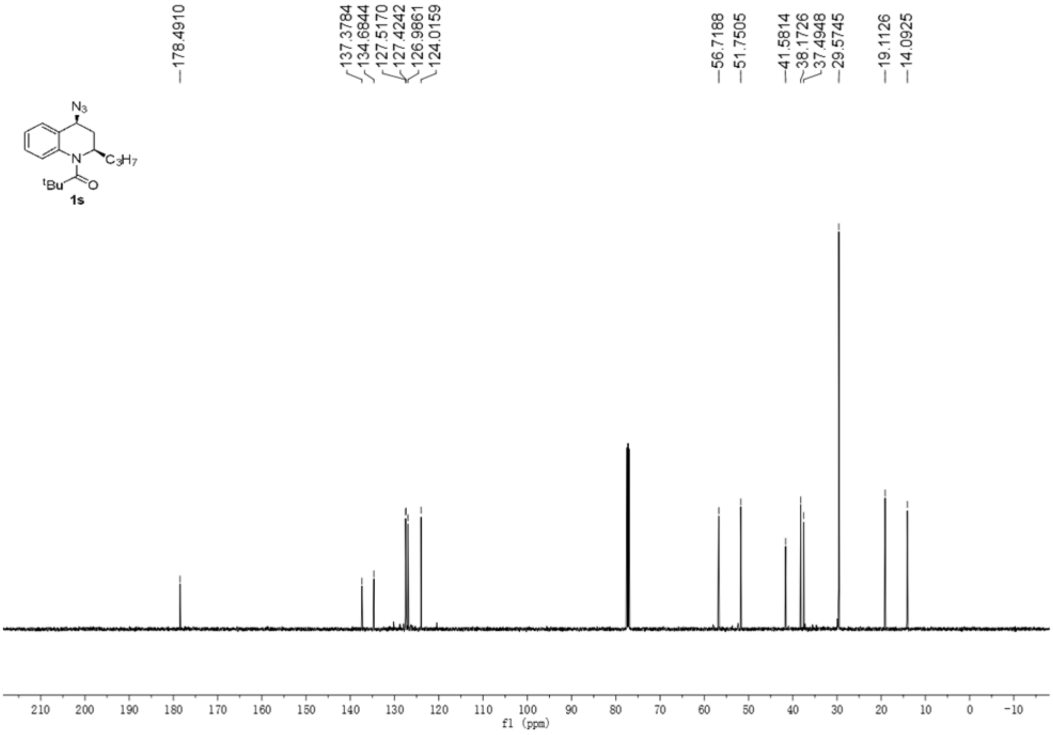

Supplementary figure 27. ^{1}H and ^{13}C NMR spectrum of compound **1s**

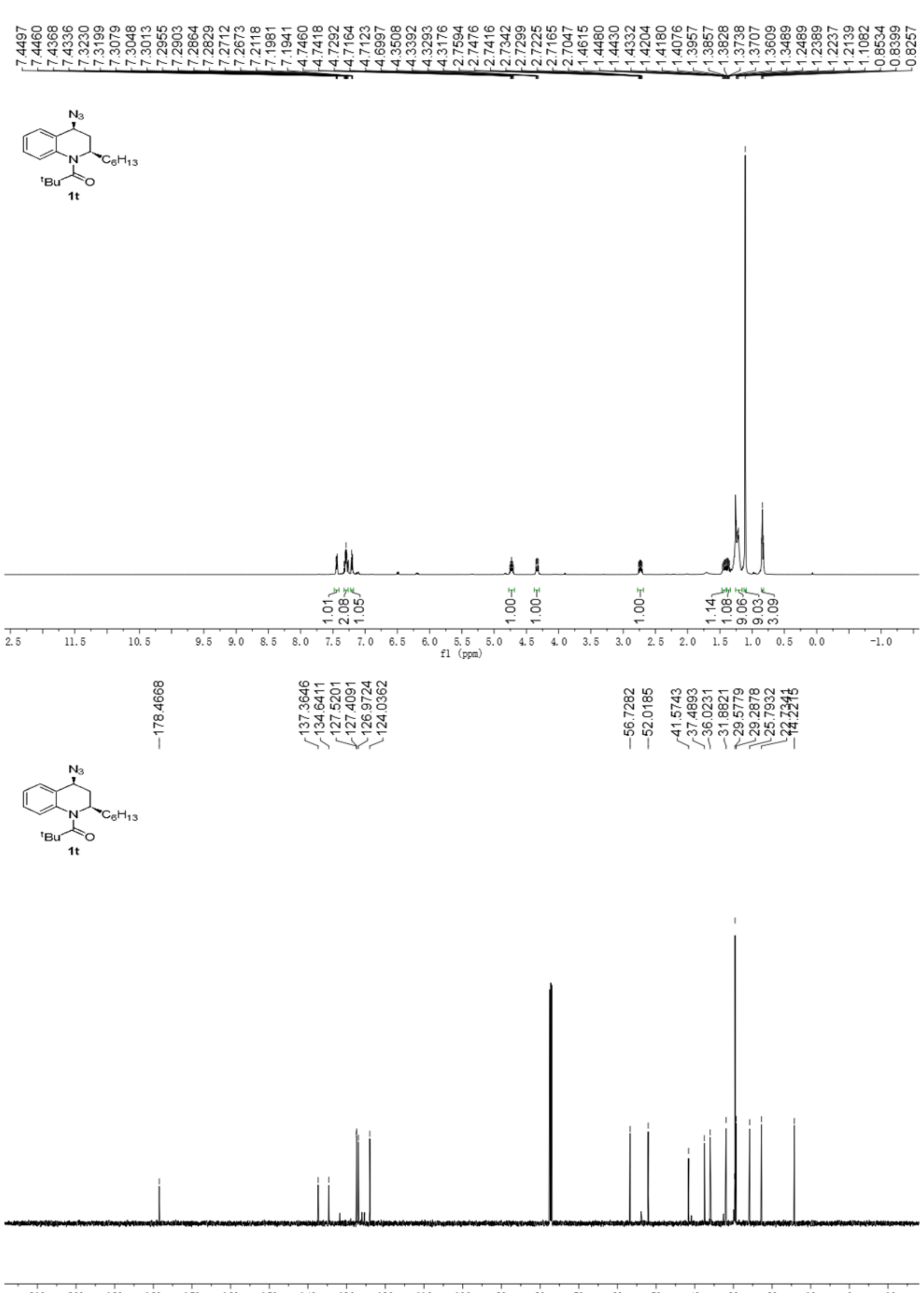

Supplementary figure 28. ^{1}H and ^{13}C NMR spectrum of compound **1t**

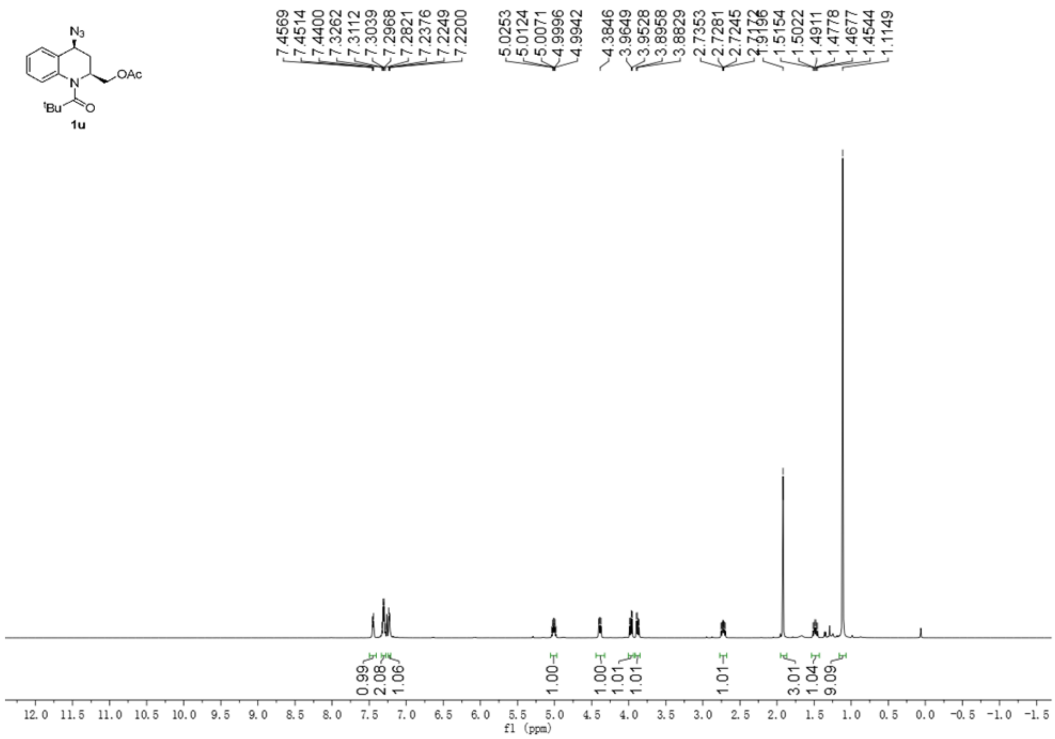

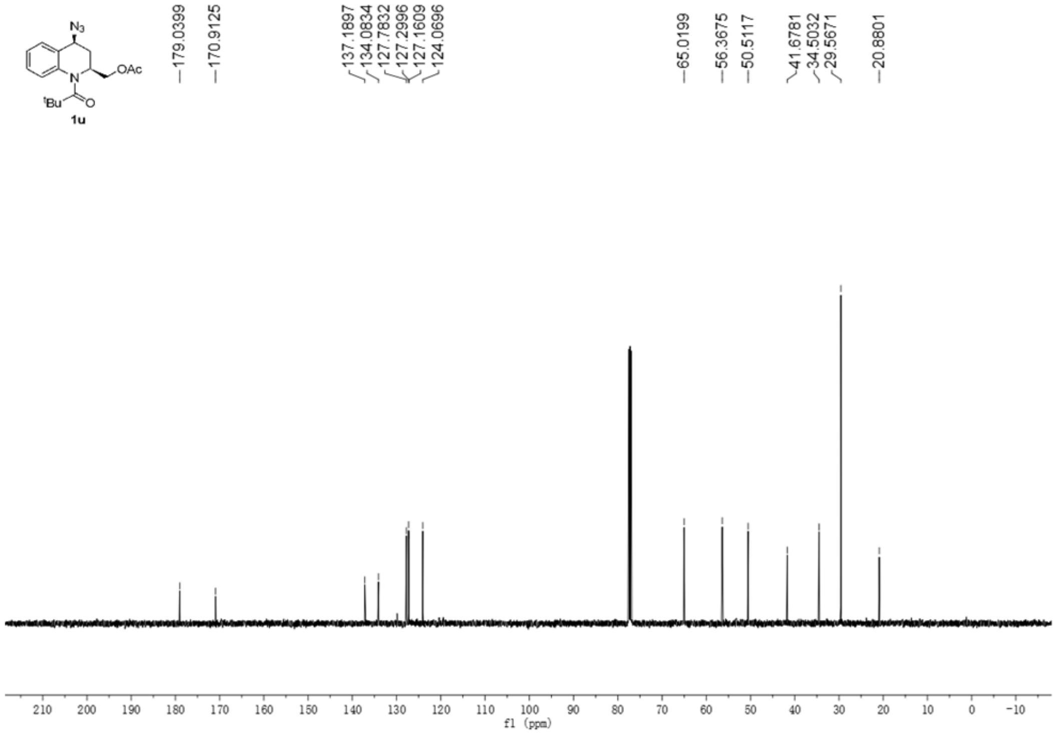

Supplementary figure 29. ^{1}H and ^{13}C NMR spectrum of compound **1u**

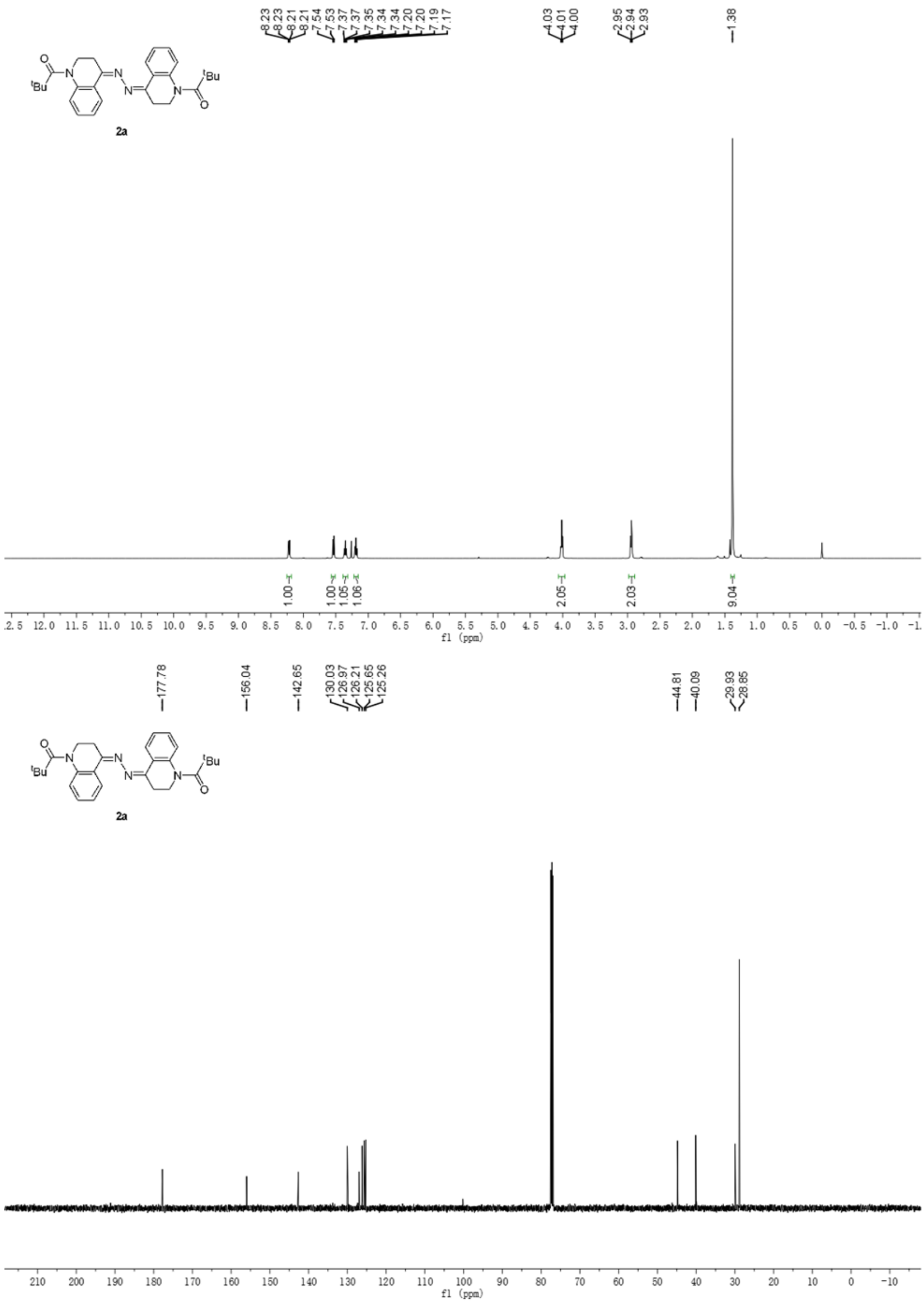

Supplementary figure 30. ^{1}H and ^{13}C NMR spectrum of compound **2a**

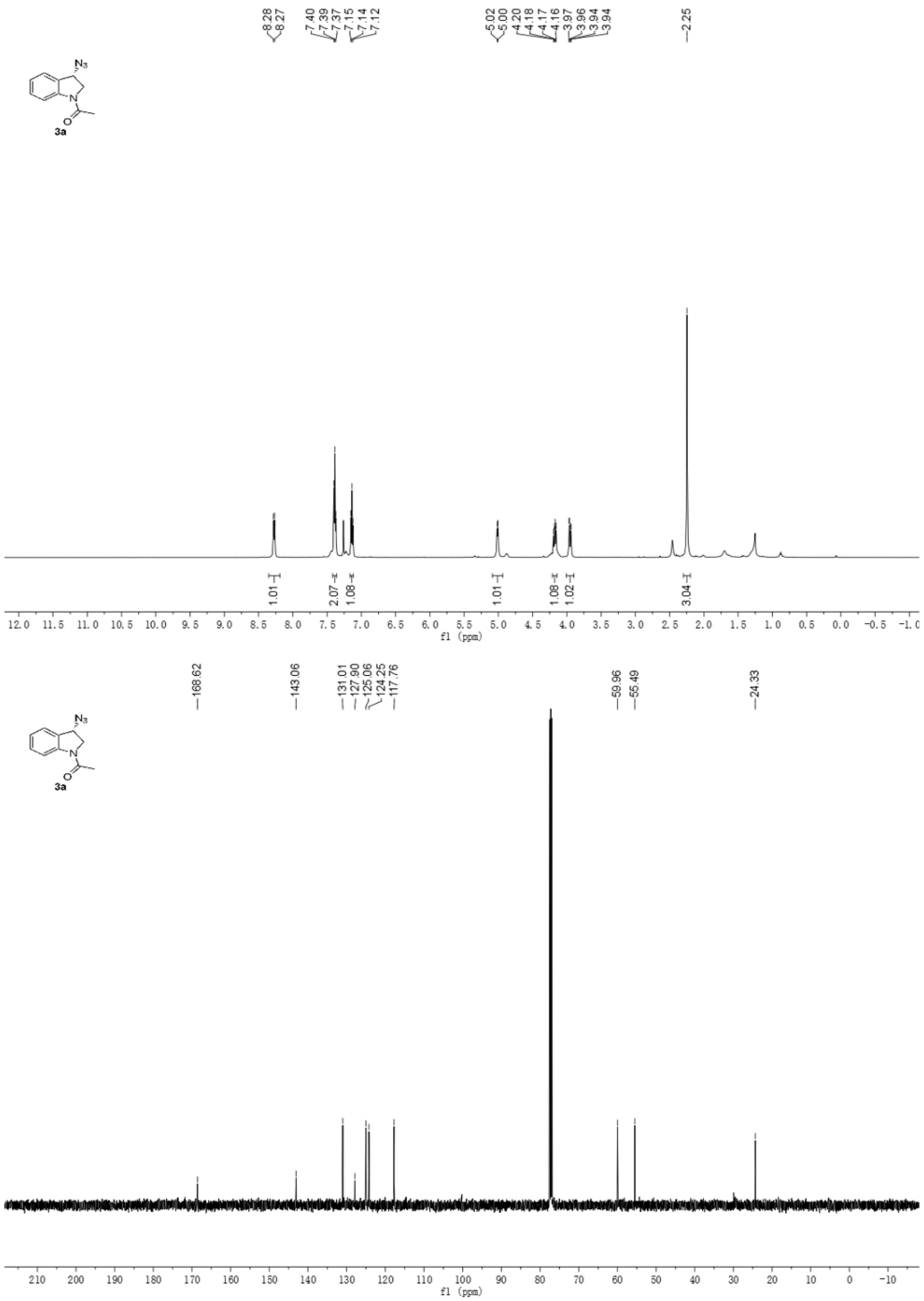

Supplementary figure 31. ^{1}H and ^{13}C NMR spectrum of compound **3a**

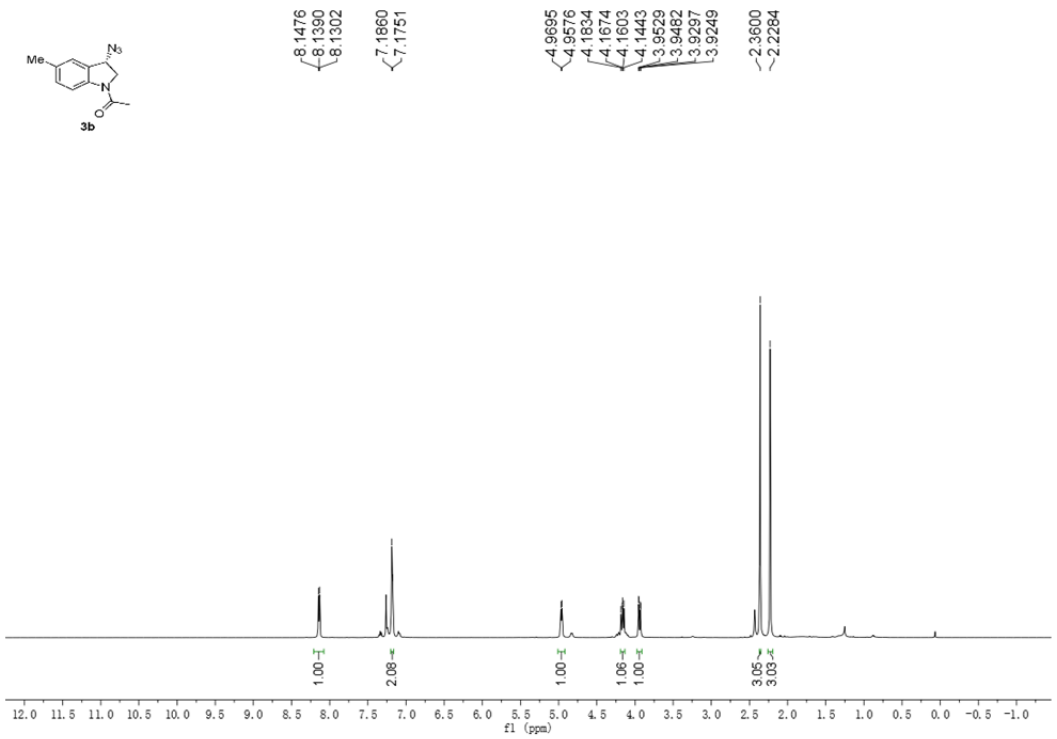

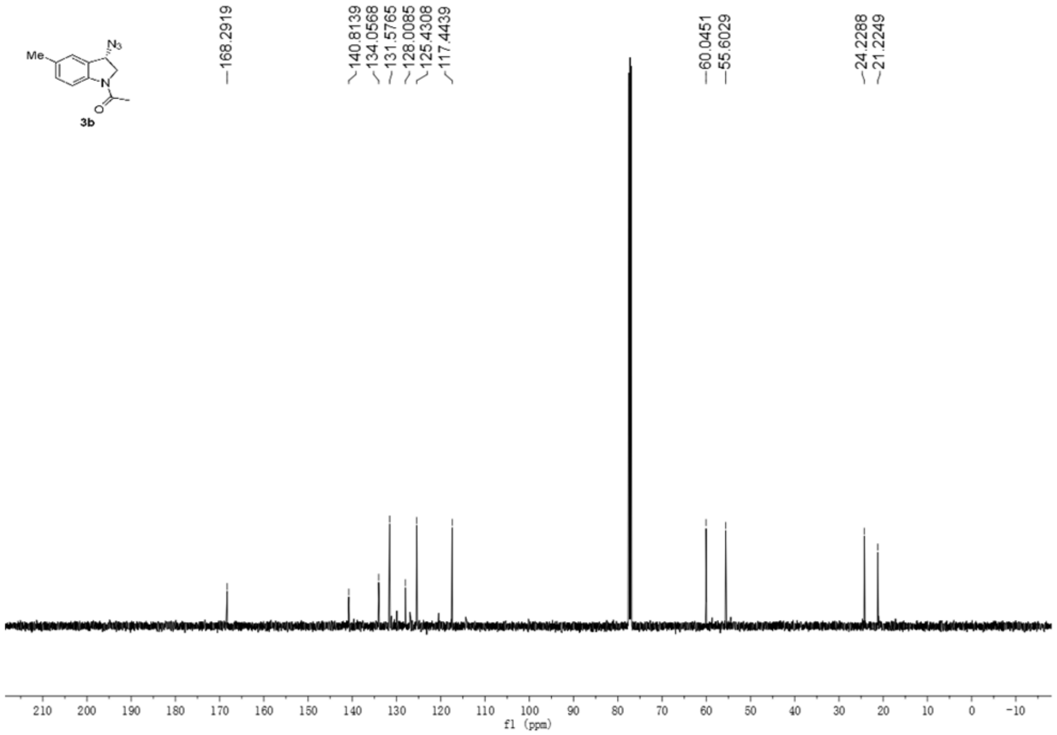

Supplementary figure 32. ^{1}H and ^{13}C NMR spectrum of compound **3b**

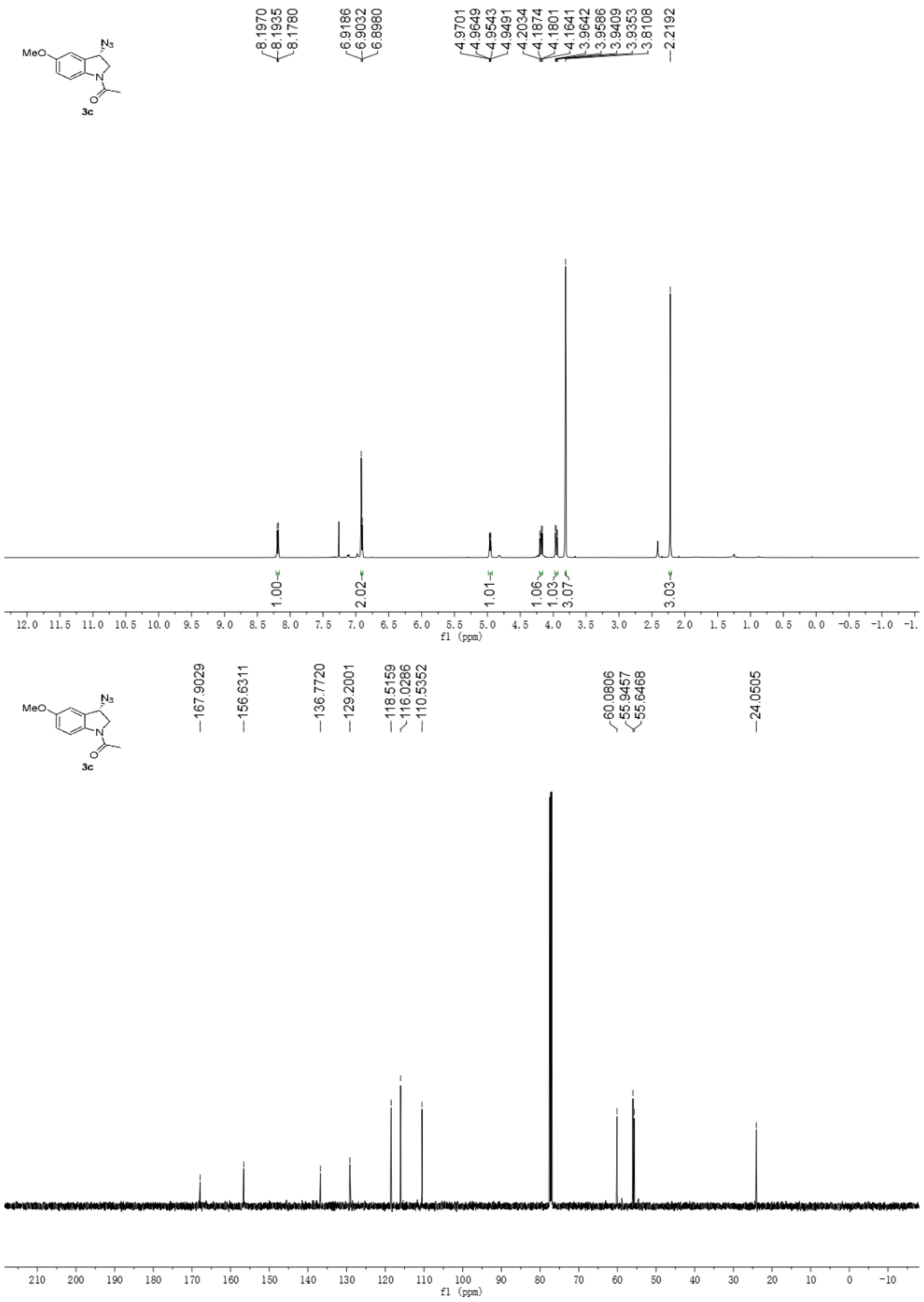

Supplementary figure 33. ^{1}H and ^{13}C NMR spectrum of compound **3c**

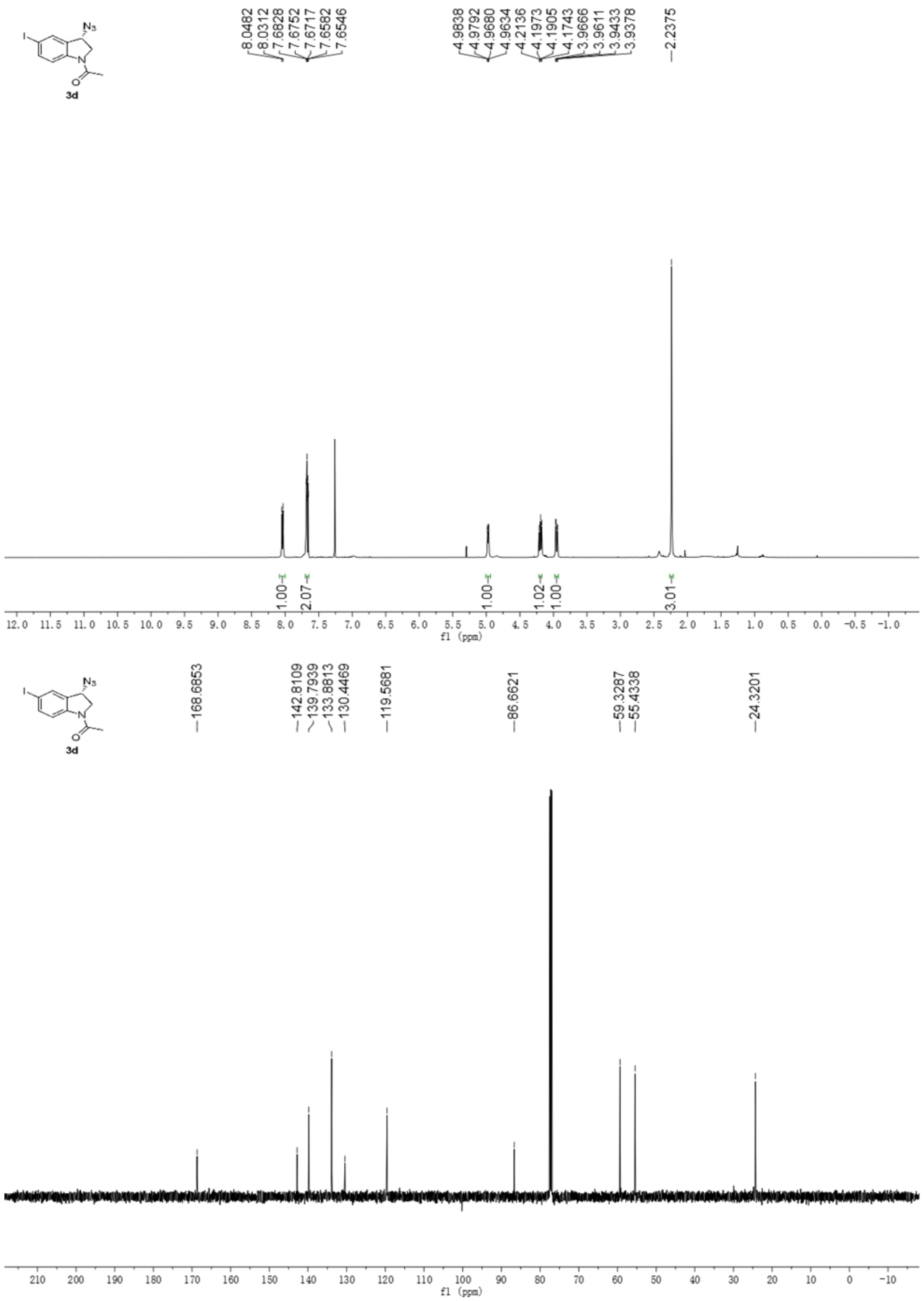

Supplementary figure 34. ^{1}H and ^{13}C NMR spectrum of compound **3d**

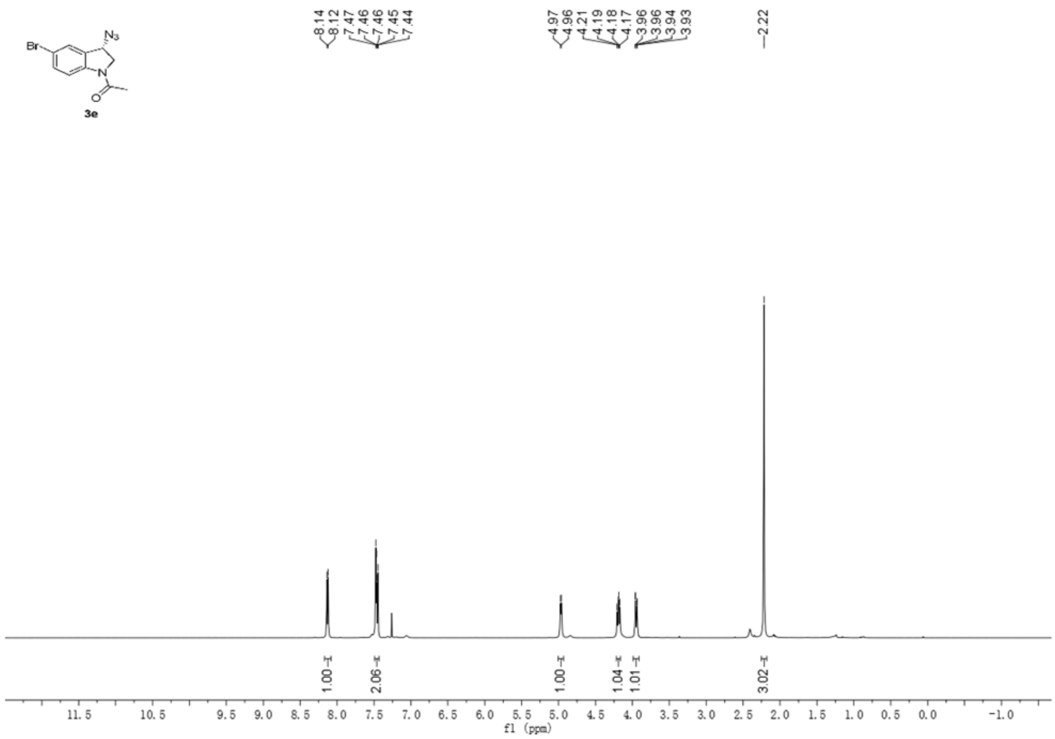

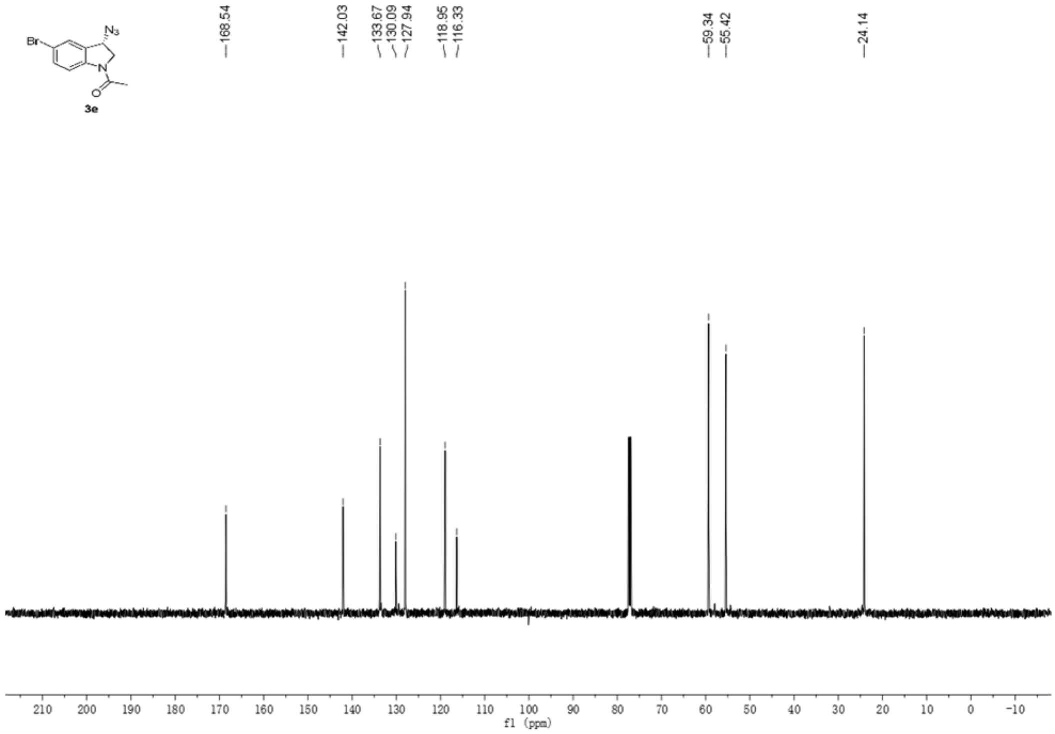

Supplementary figure 35. ^{1}H and ^{13}C NMR spectrum of compound **3e**

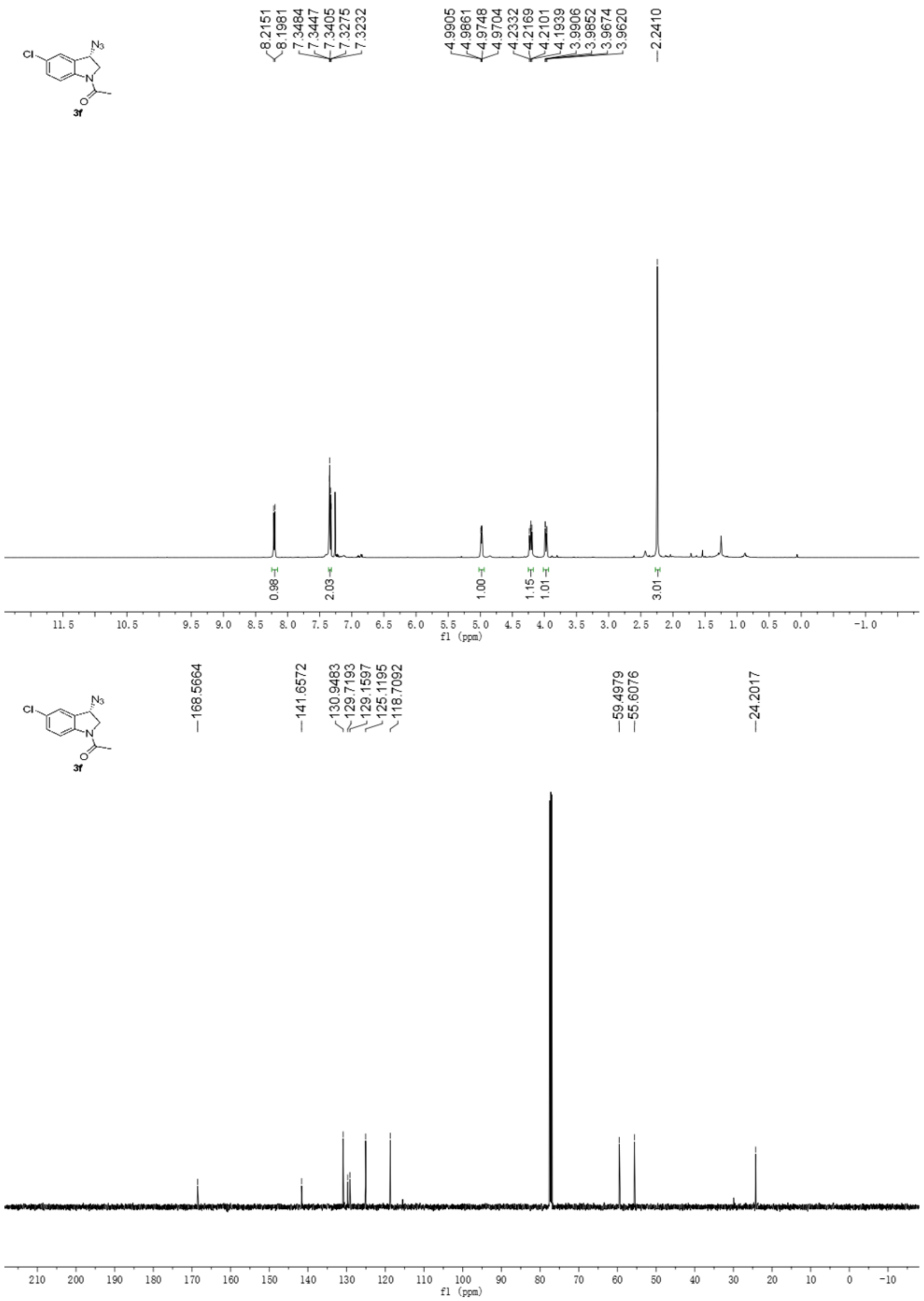

Supplementary figure 36. ^{1}H and ^{13}C NMR spectrum of compound **3f**

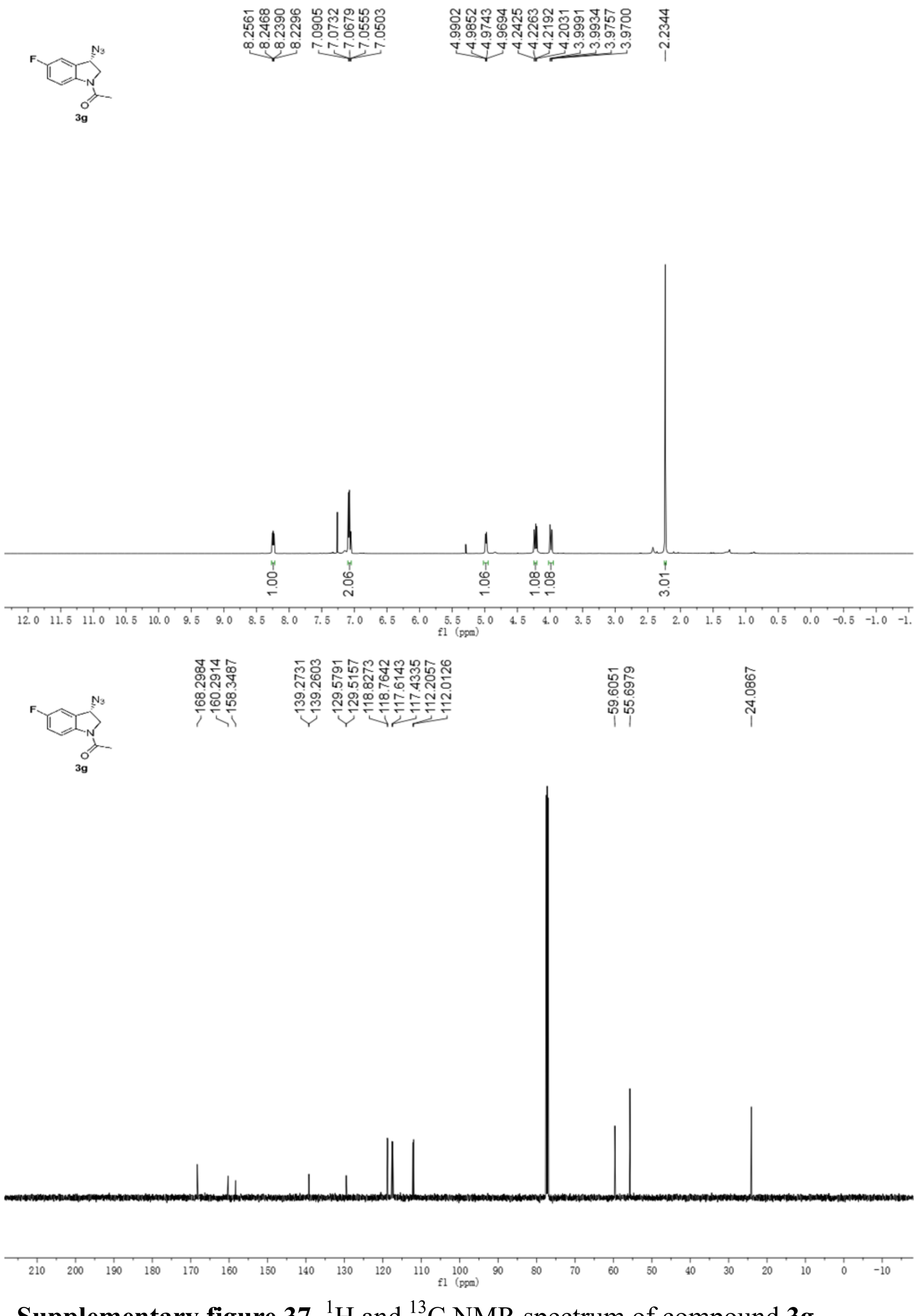

Supplementary figure 37. ^{1}H and ^{13}C NMR spectrum of compound **3g**

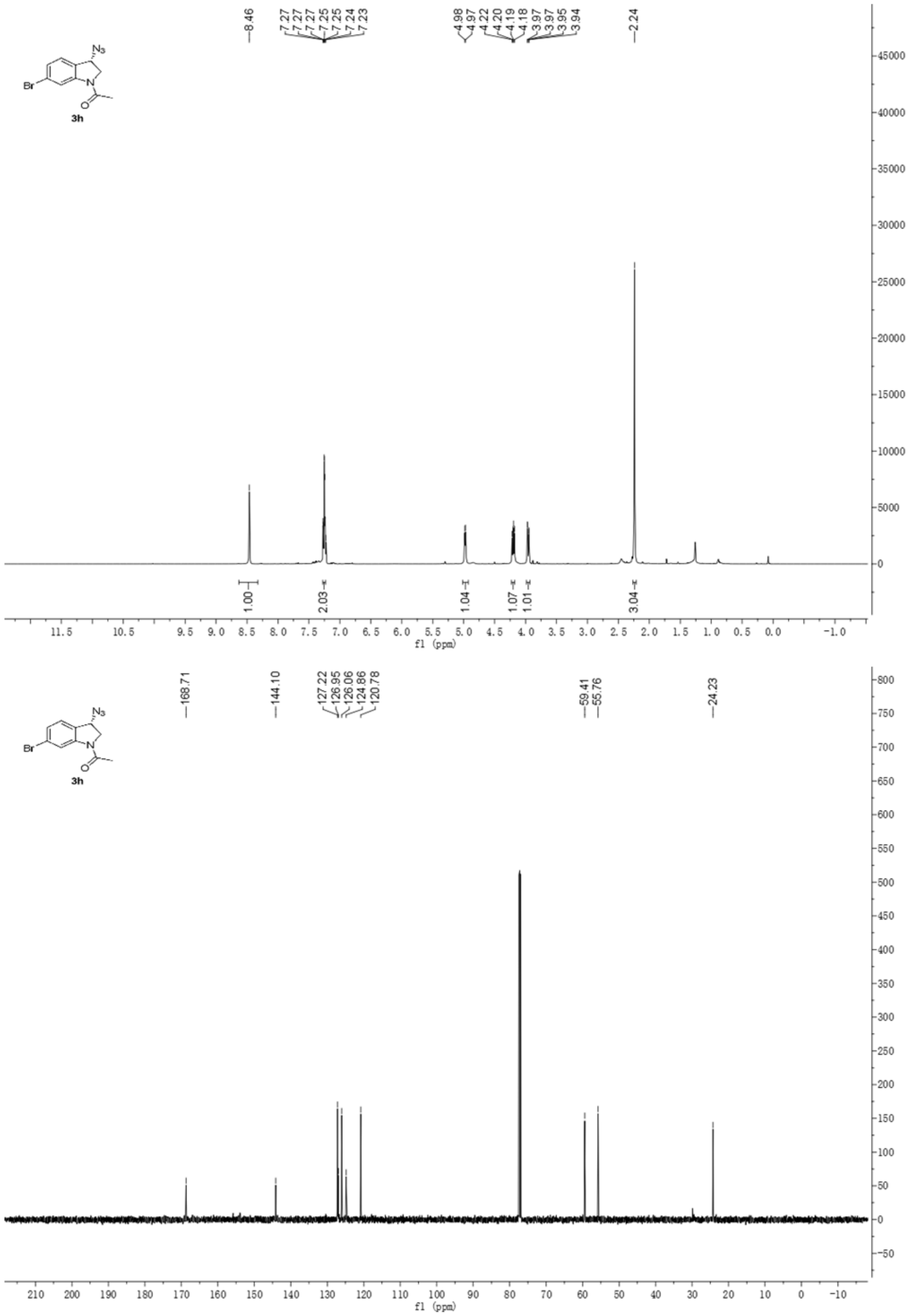

Supplementary figure 38. ^{1}H and ^{13}C NMR spectrum of compound **3h**

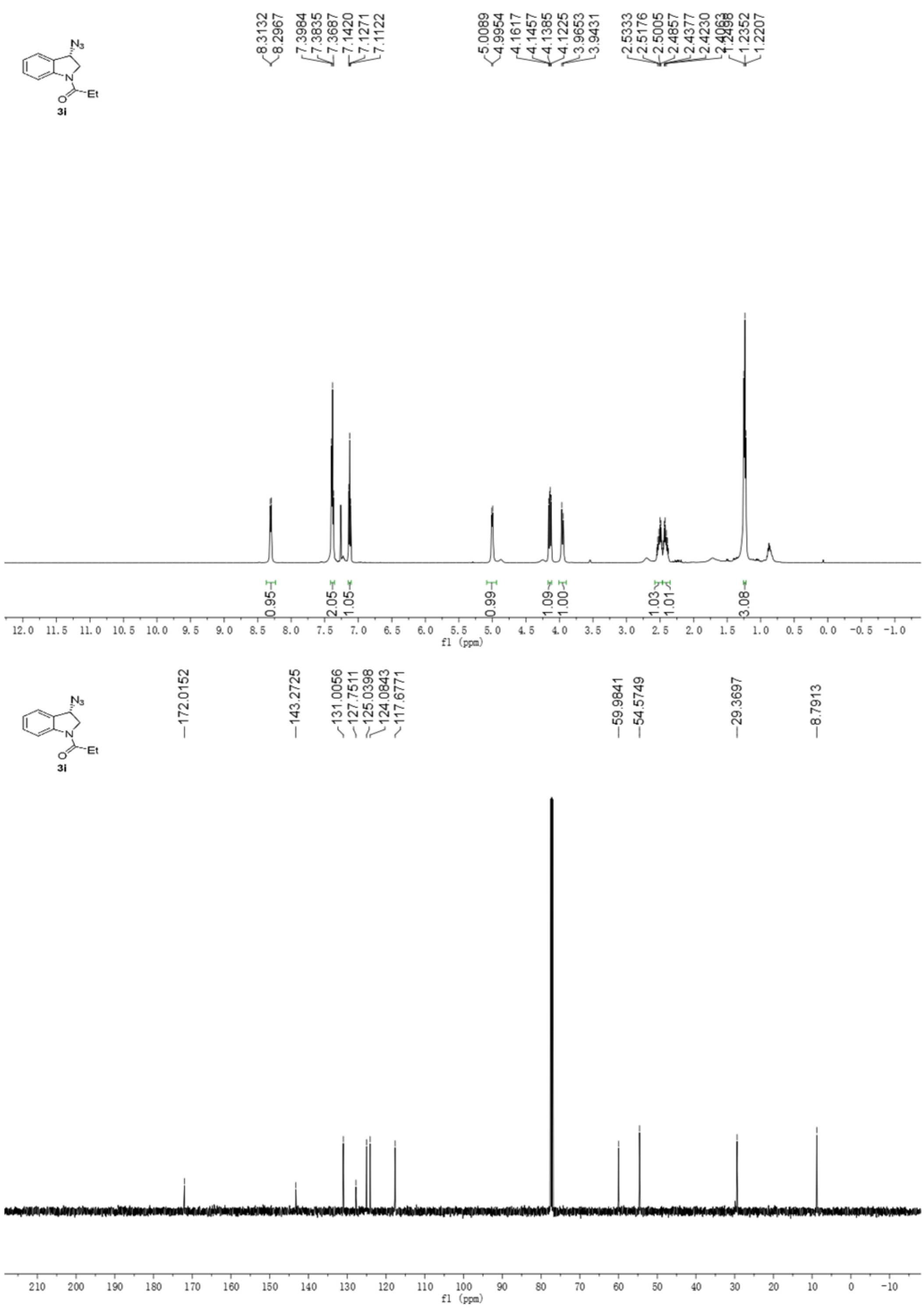

Supplementary figure 39. ^{1}H and ^{13}C NMR spectrum of compound **3i**

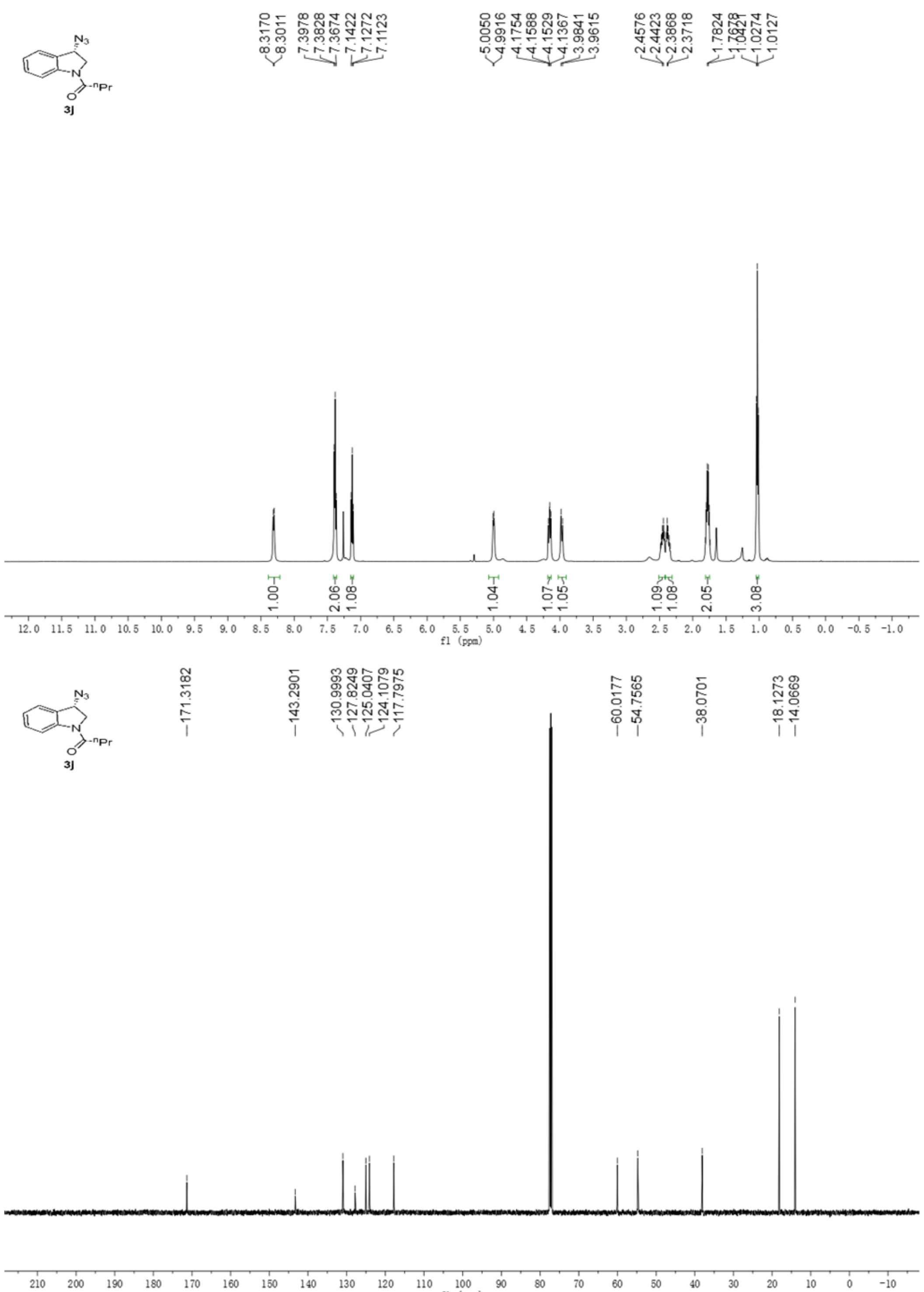

Supplementary figure 40. ^{1}H and ^{13}C NMR spectrum of compound **3j**

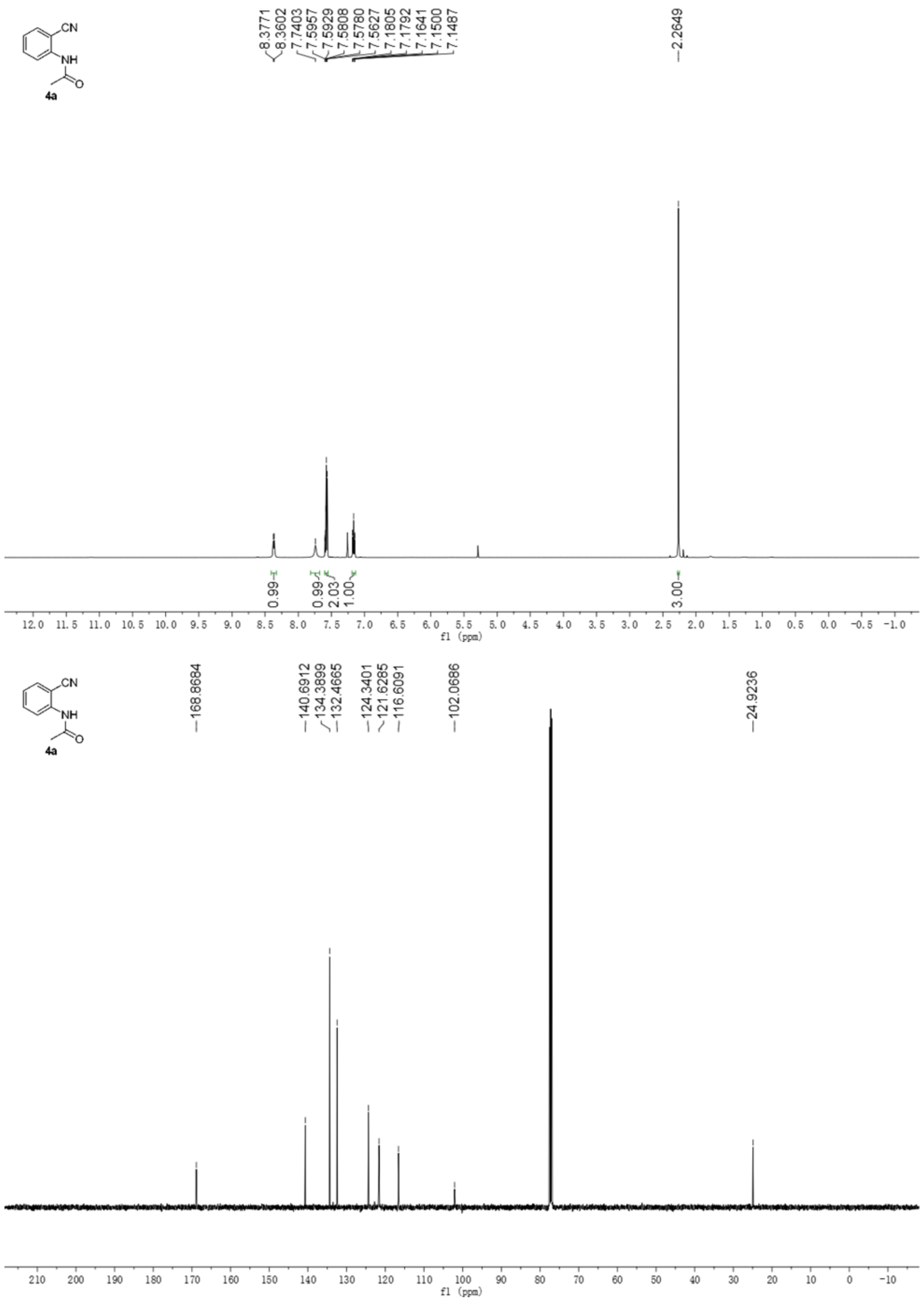

Supplementary figure 41. ^{1}H and ^{13}C NMR spectrum of compound **4a**

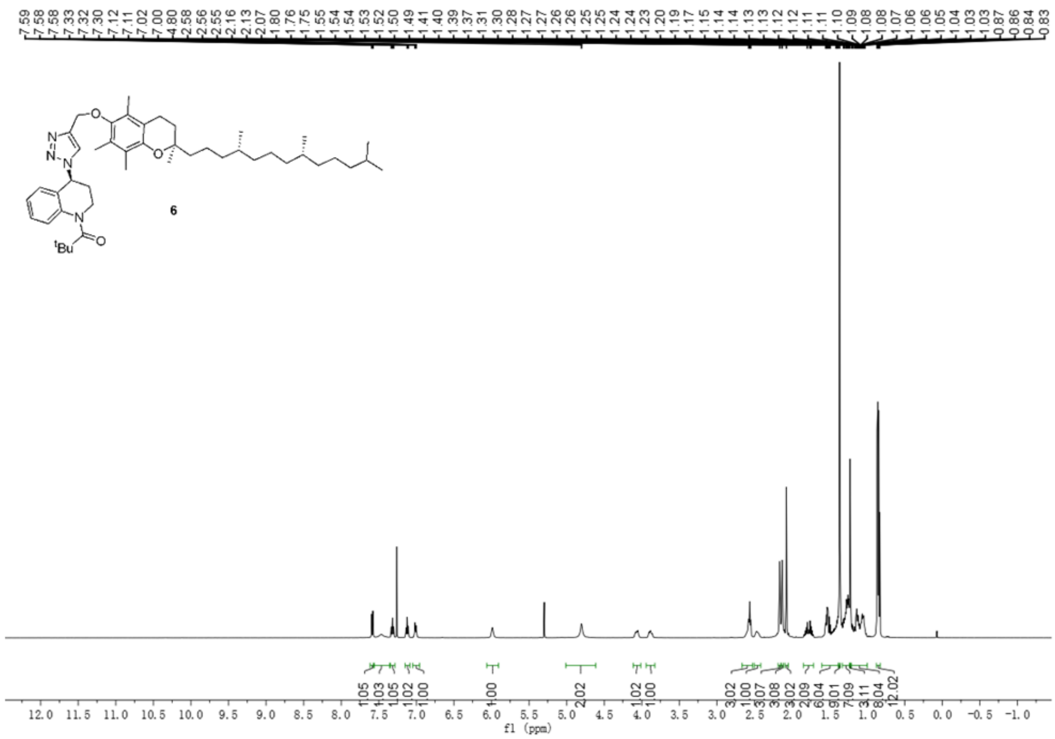

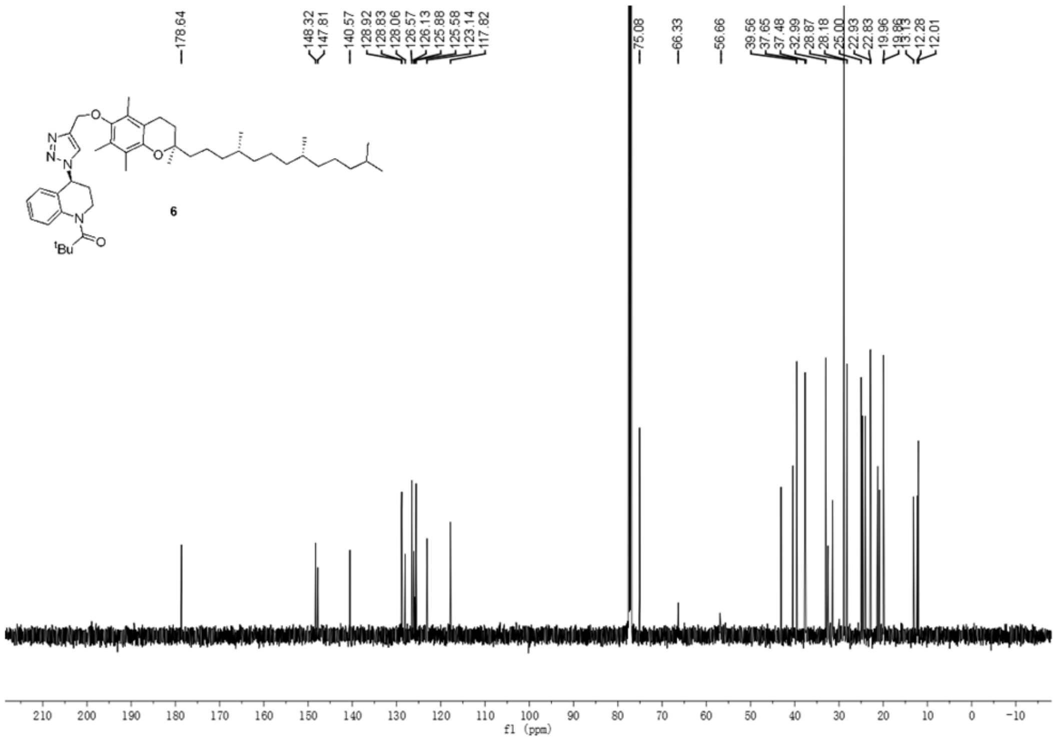

Supplementary figure 42. ^{1}H and ^{13}C NMR spectrum of compound **6**

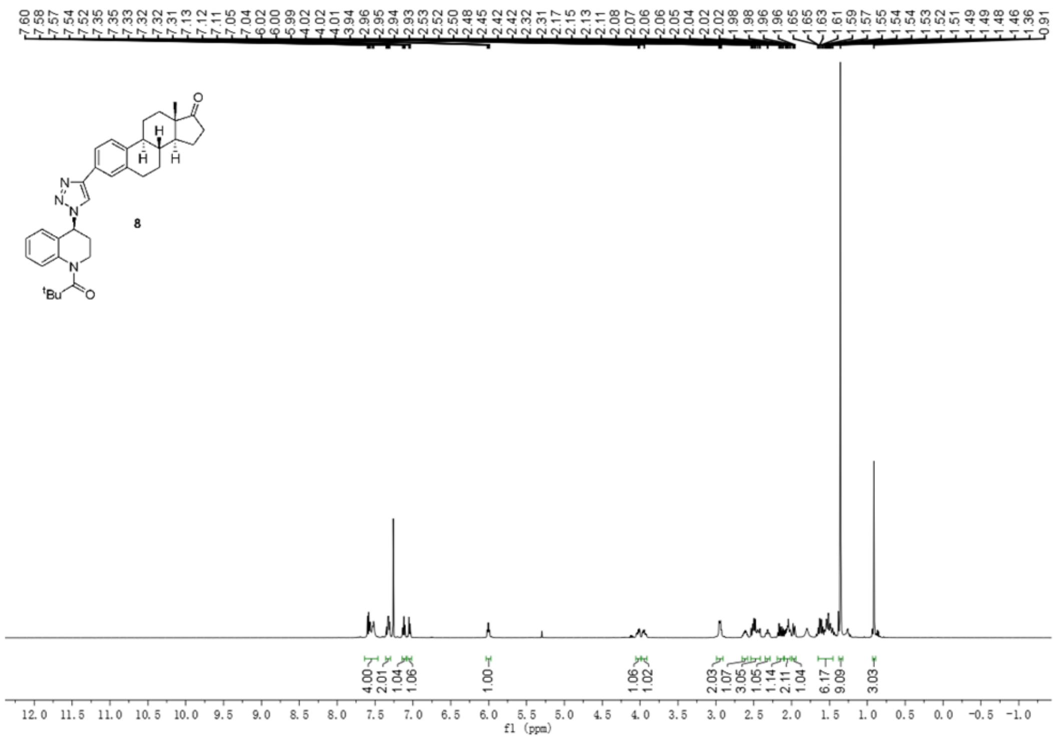

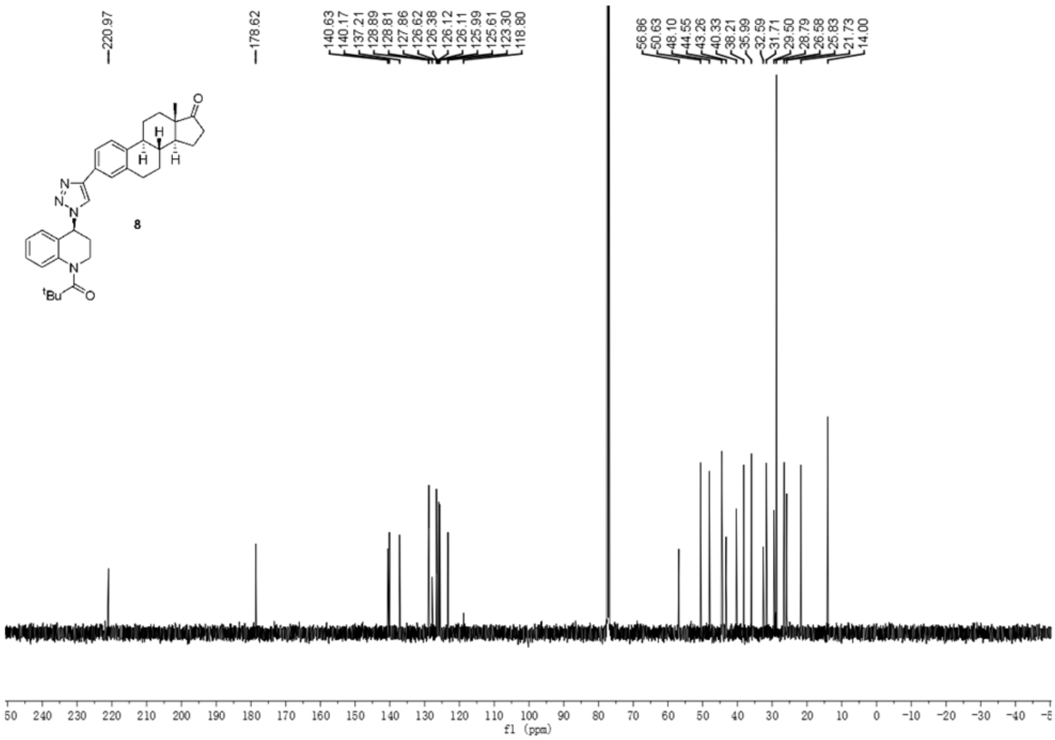

Supplementary figure 43. ^{1}H and ^{13}C NMR spectrum of compound **8**

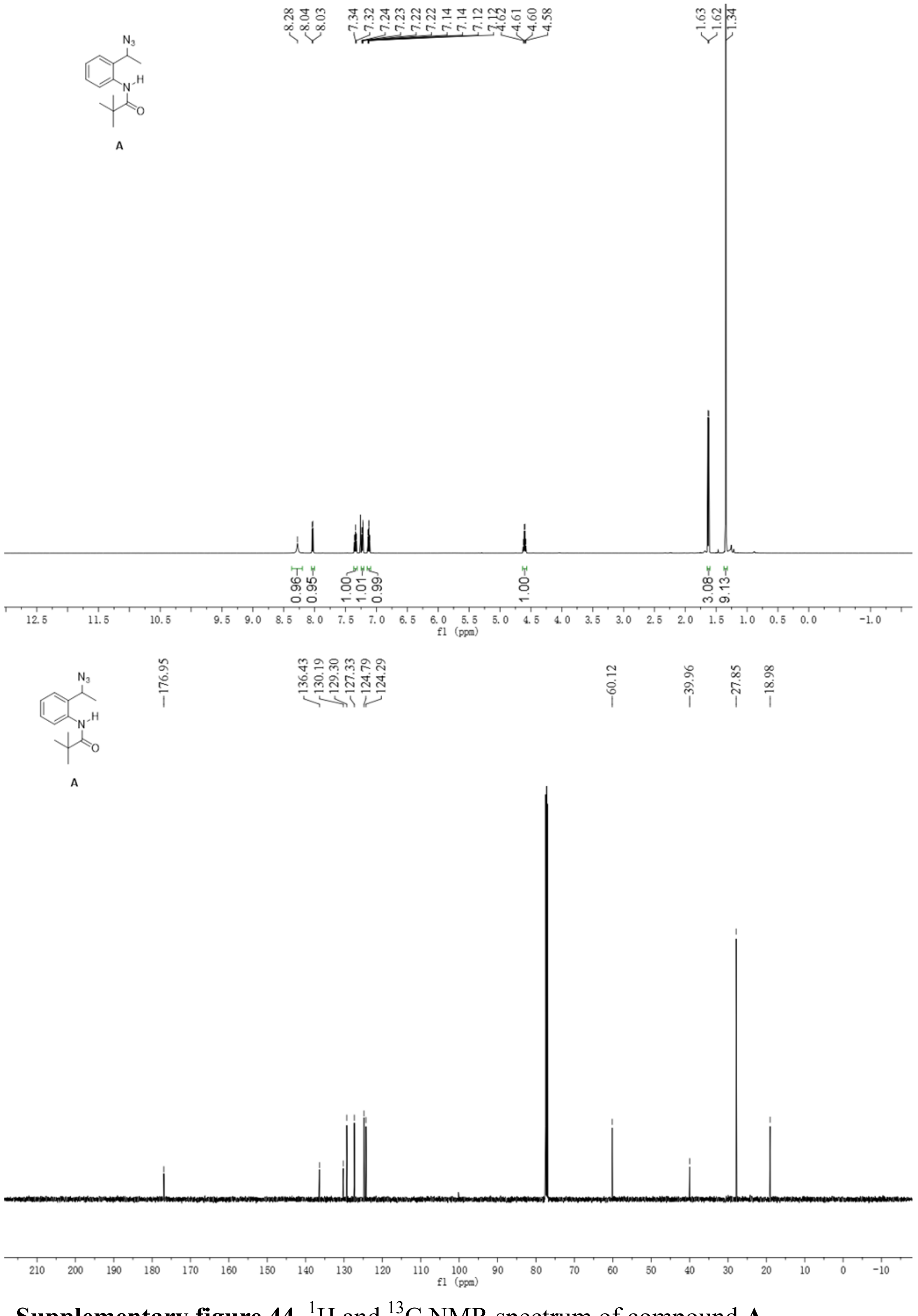

Supplementary figure 44. ^{1}H and ^{13}C NMR spectrum of compound **A**

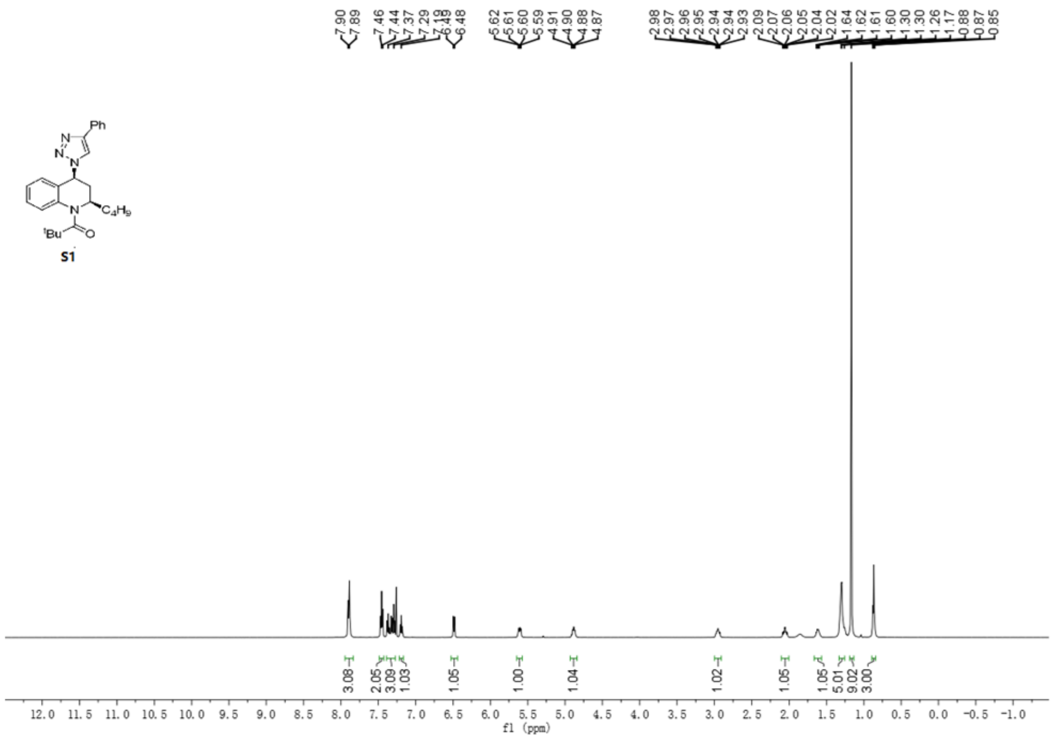

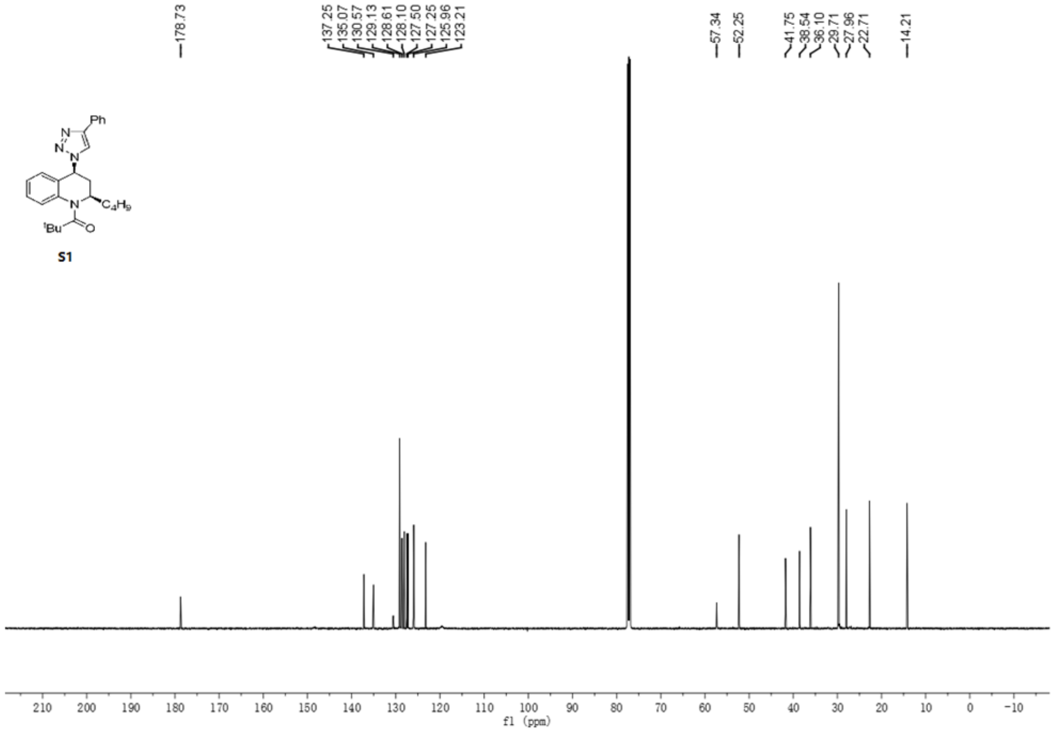

Supplementary figure 45. ^{1}H and ^{13}C NMR spectrum of compound **S1**

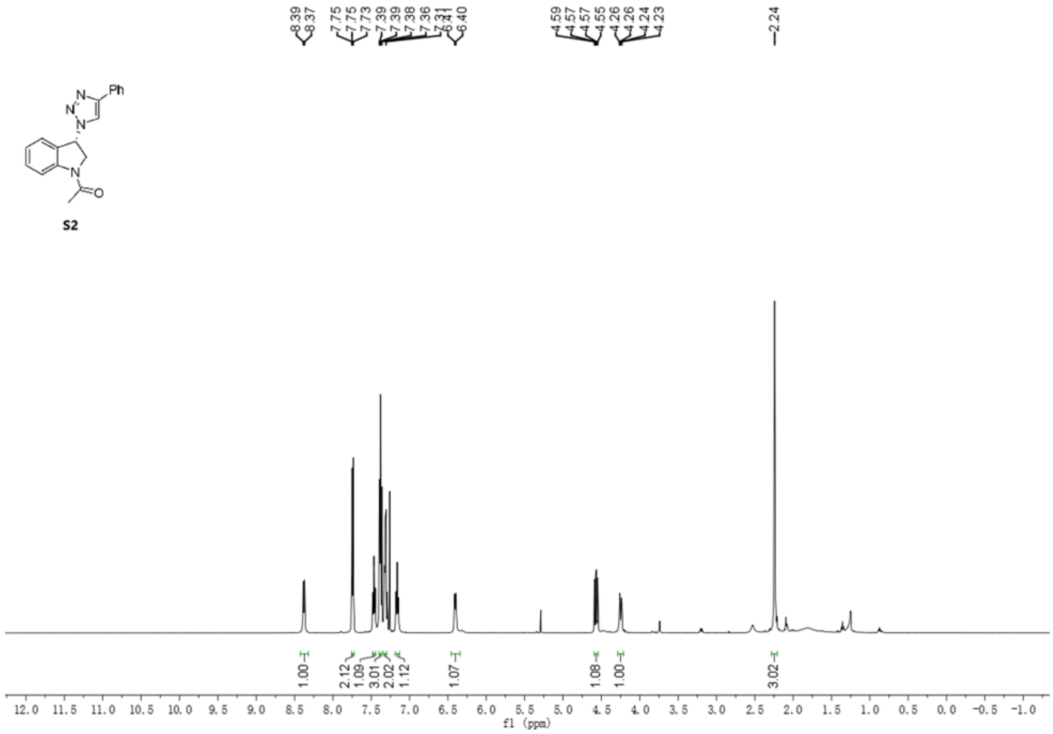

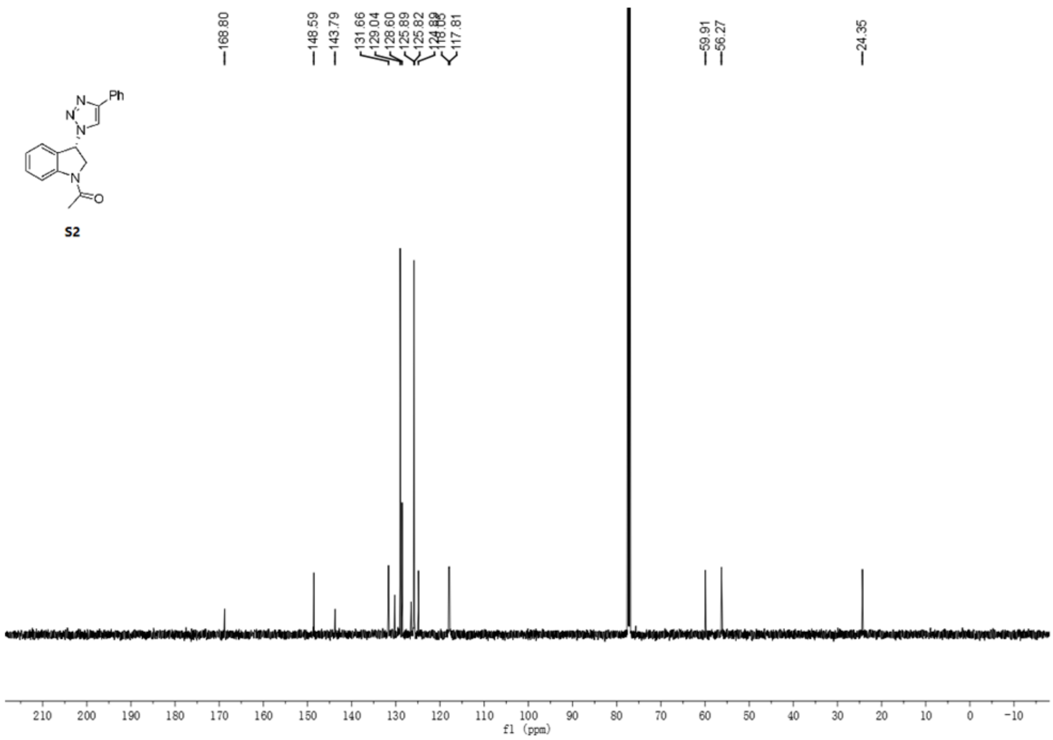

Supplementary figure 46. ^{1}H and ^{13}C NMR spectrum of compound **S2**

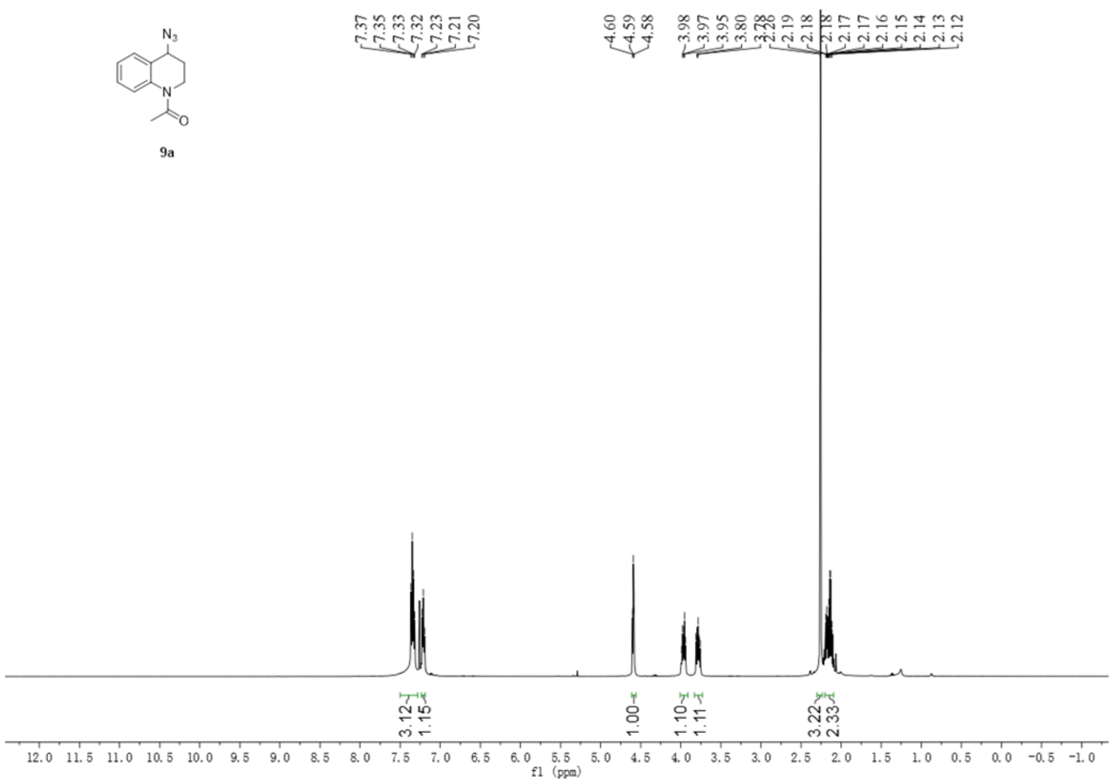

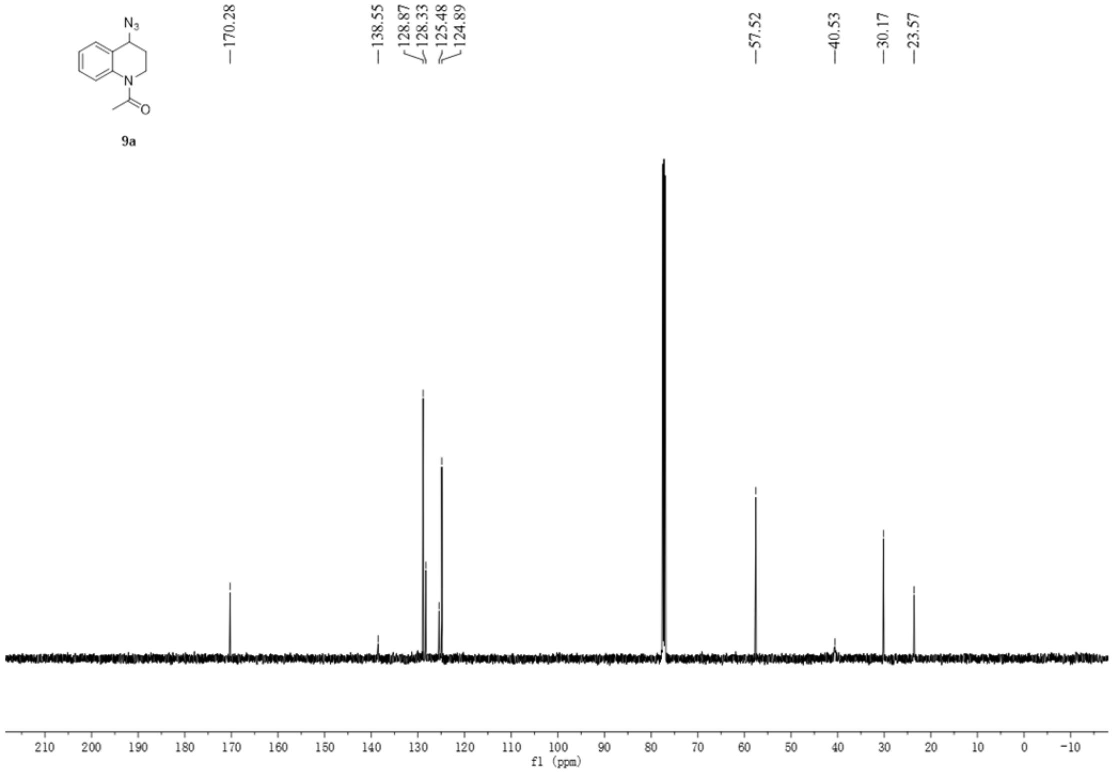

Supplementary figure 47. ^{1}H and ^{13}C NMR spectrum of compound **9a**

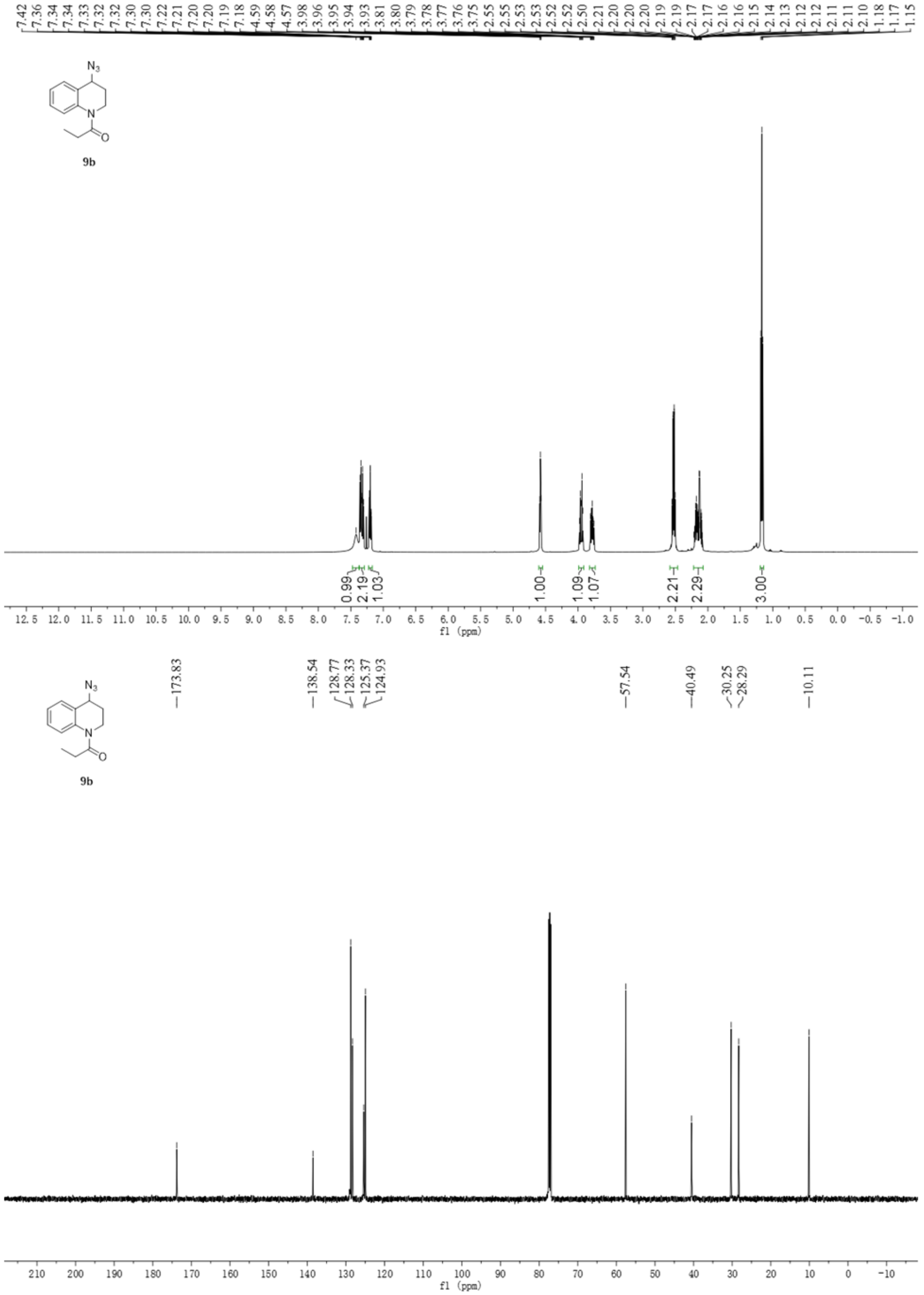

Supplementary figure 48. ^{1}H and ^{13}C NMR spectrum of compound **9b**

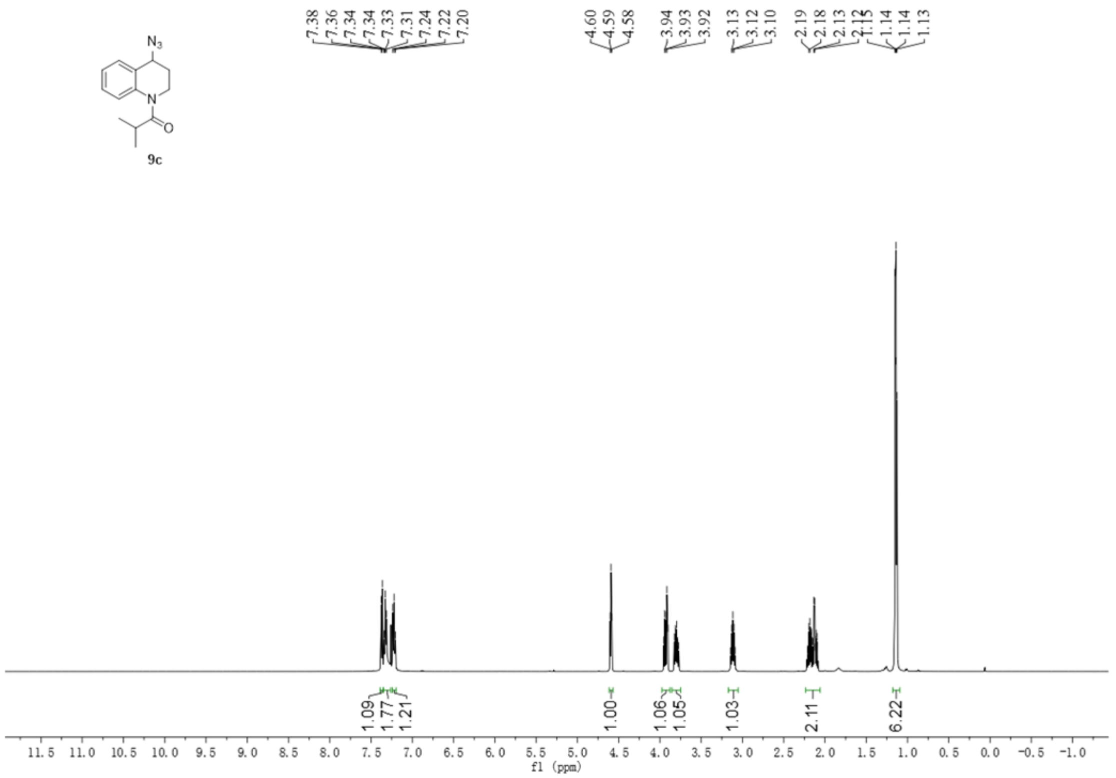

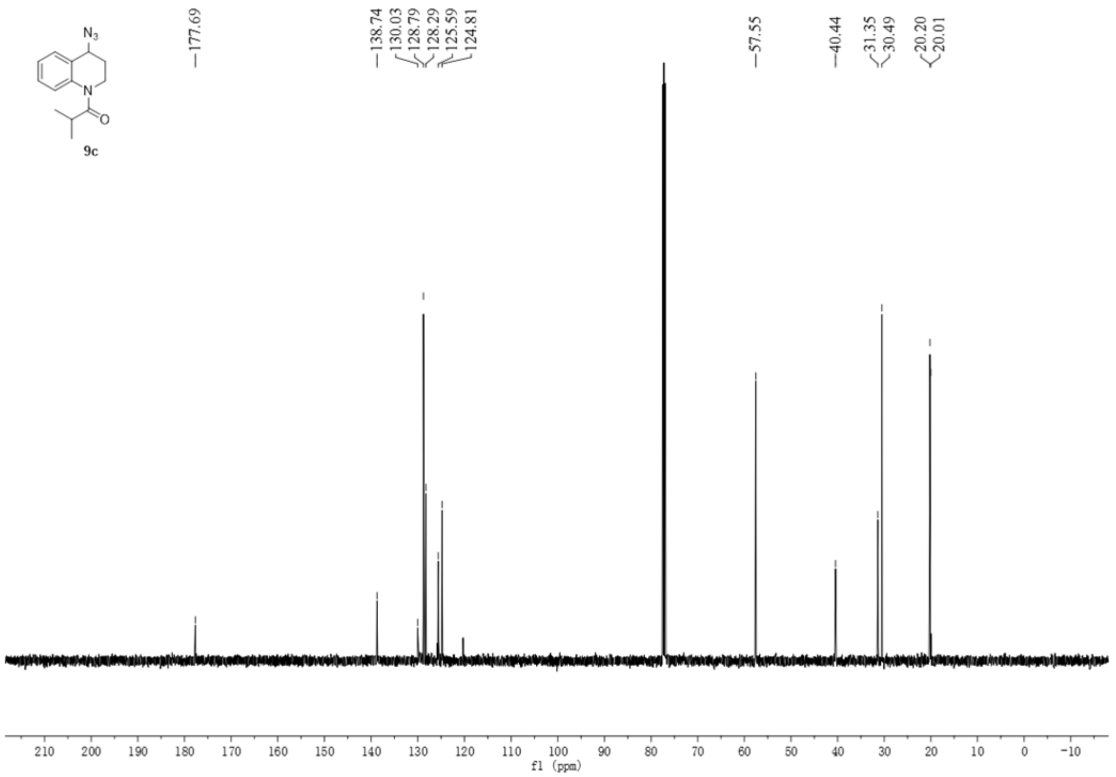

Supplementary figure 49. ^{1}H and ^{13}C NMR spectrum of compound **9c**

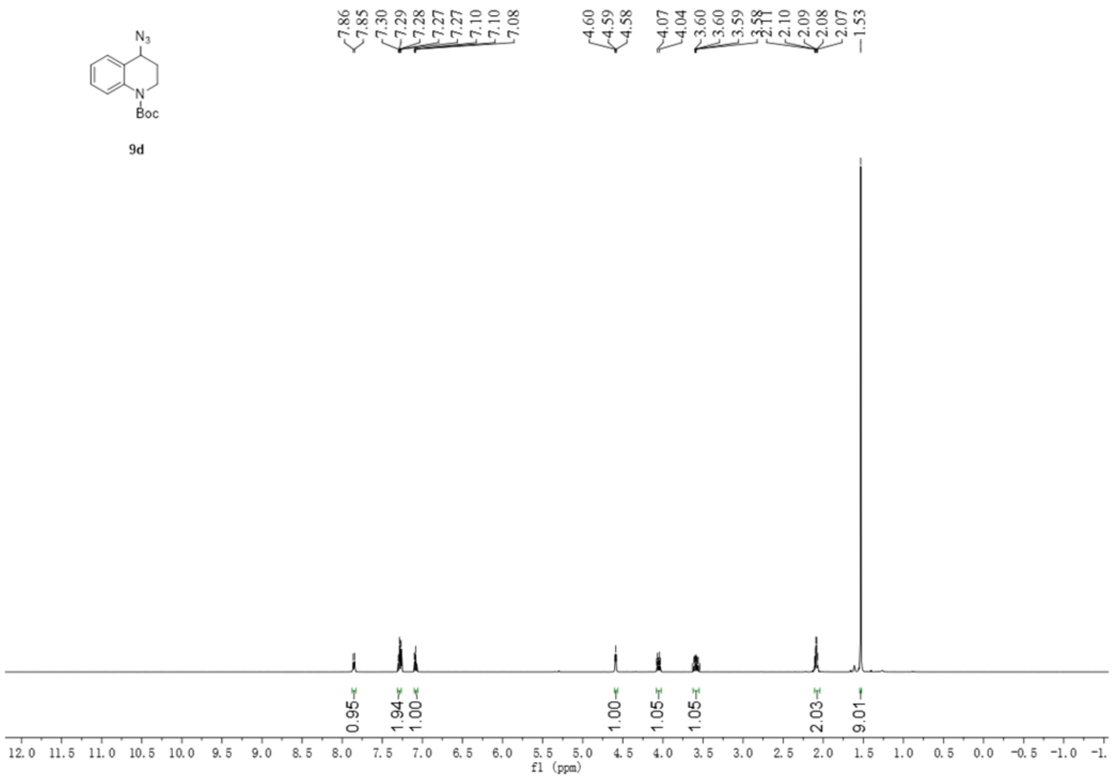

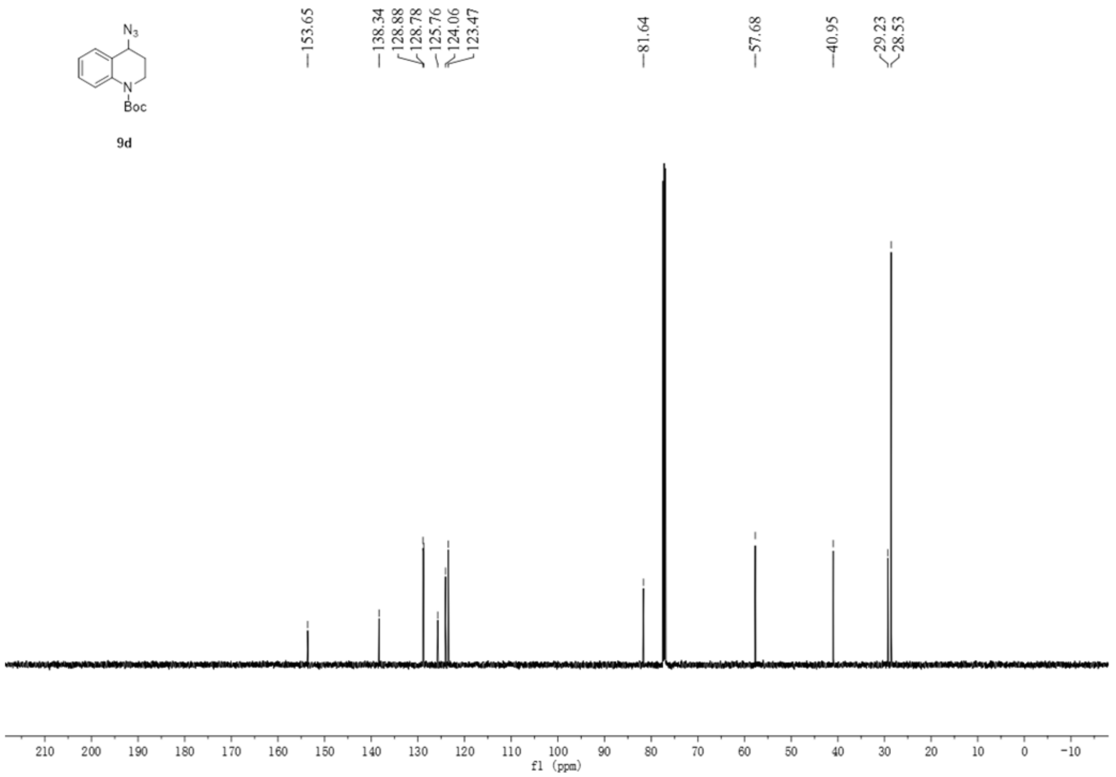

Supplementary figure 50. ^{1}H and ^{13}C NMR spectrum of compound **9d**

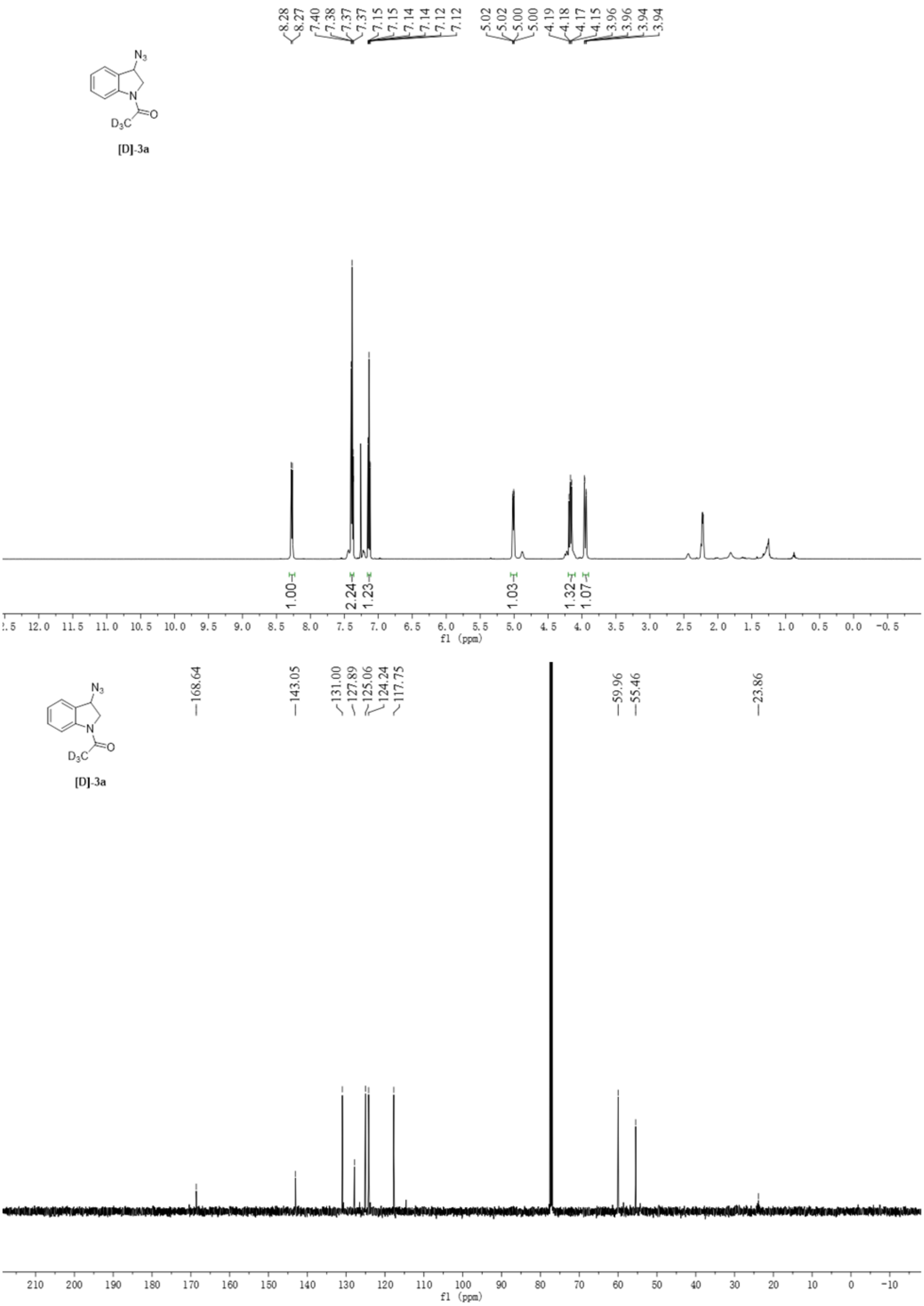

Supplementary figure 51. ^{1}H and ^{13}C NMR spectrum of compound [D]-**3a**

HPLC Traces

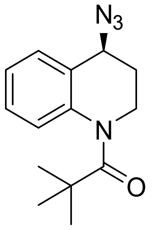

(*S*)-1-(4-Azido-3,4-dihydroquinolin-1(2*H*)-yl)-2,2-dimethylpropan-1-one (1a)

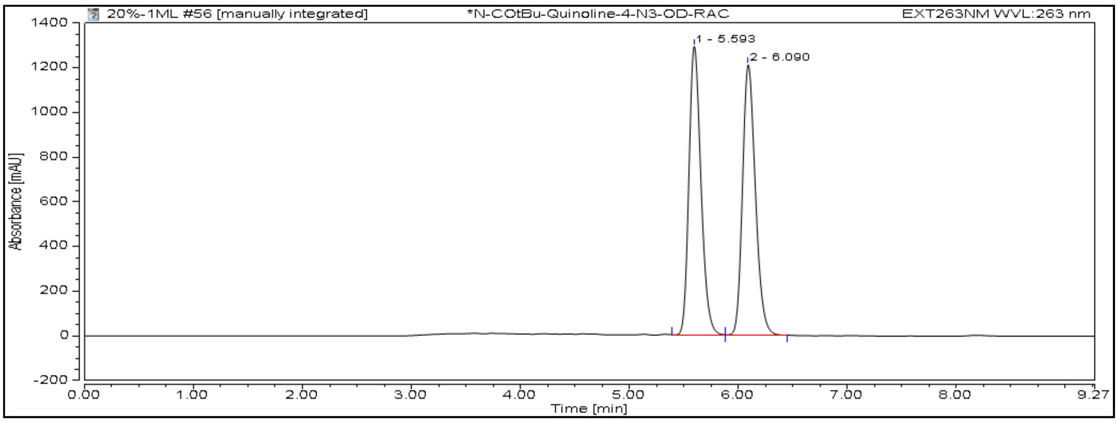

Integration Results					
No.	Peak Name	Retention Time<br>min	Area<br>mAU*min	Relative Area<br>%	Amount<br>n.a.
1		5.593	164.579	49.86	n.a.
2		6.090	165.506	50.14	n.a.
**Total:**			**330.085**	**100.00**	

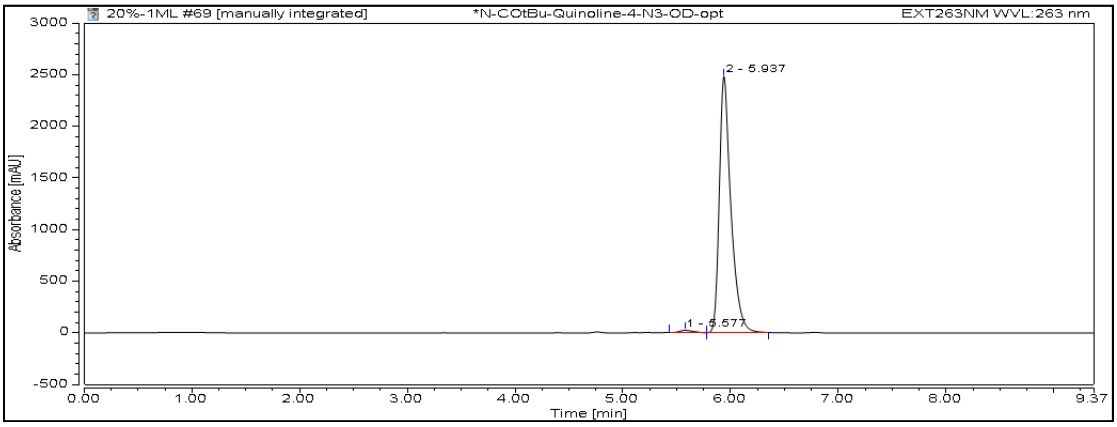

Integration Results					
No.	Peak Name	Retention Time<br>min	Area<br>mAU*min	Relative Area<br>%	Amount<br>n.a.
1		5.577	2.990	0.96	n.a.
2		5.937	308.154	99.04	n.a.
**Total:**			**311.144**	**100.00**	

Supplementary figure 52. HPLC chromatogram for **1a**

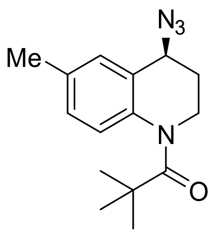

(*S*)-1-(4-Azido-6-methyl-3,4-dihydroquinolin-1(2*H*)-yl)-2,2-dimethylpropan-1-one (1b)

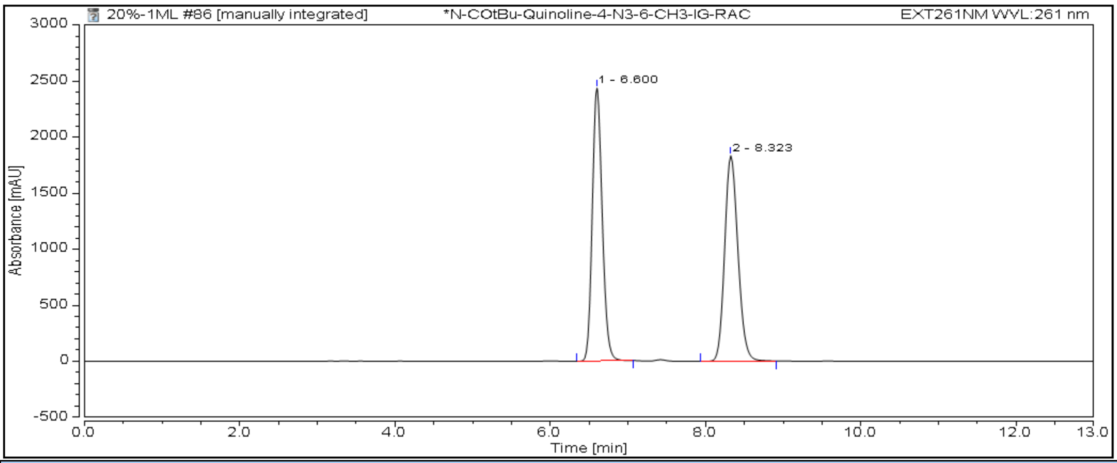

Integration Results

No.	Peak Name	Retention Time min	Area mAU*min	Relative Area %	Amount n.a.
1		6.600	360.163	49.80	n.a.
2		8.323	363.084	50.20	n.a.
**Total:**			**723.247**	**100.00**	

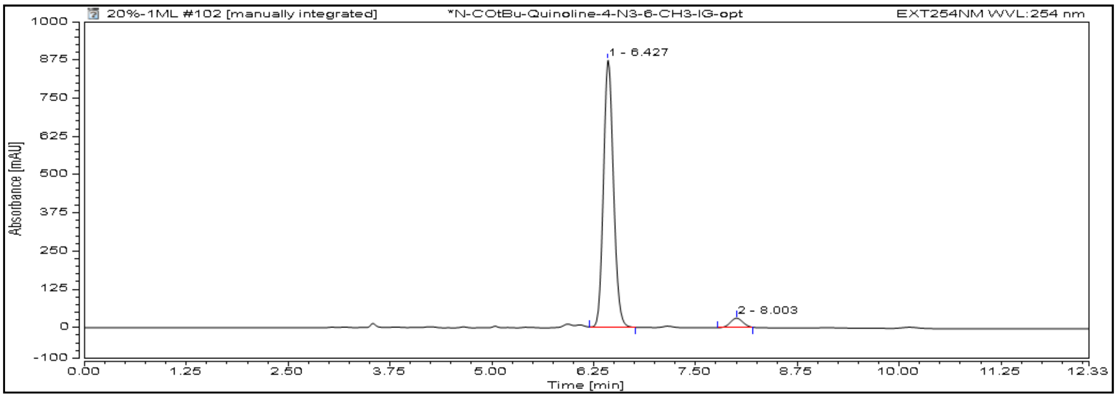

Integration Results

No.	Peak Name	Retention Time min	Area mAU*min	Relative Area %	Amount n.a.
1		6.427	124.290	95.81	n.a.
2		8.003	5.442	4.19	n.a.
**Total:**			**129.732**	**100.00**	

Supplementary figure 53. HPLC chromatogram for **1b**

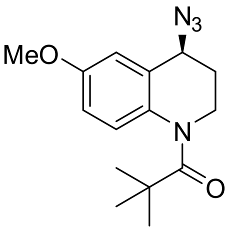

(*S*)-1-(4-Azido-6-methoxy-3,4-dihydroquinolin-1(2*H*)-yl)-2,2-dimethylpropan-1-one (1c)

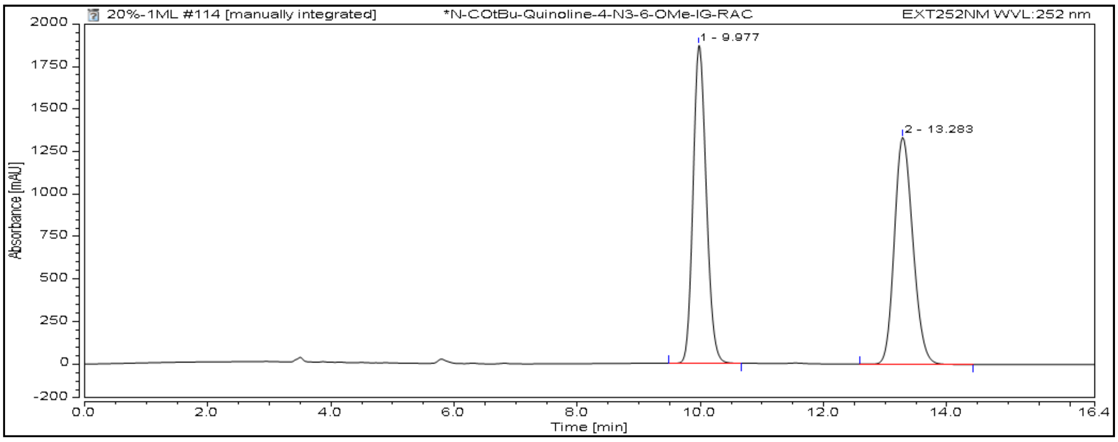

Integration Results					
No.	Peak Name	Retention Time min	Area mAU*min	Relative Area %	Amount n.a.
1		9.977	464.098	49.95	n.a.
2		13.283	465.083	50.05	n.a.
**Total:**			**929.181**	**100.00**	

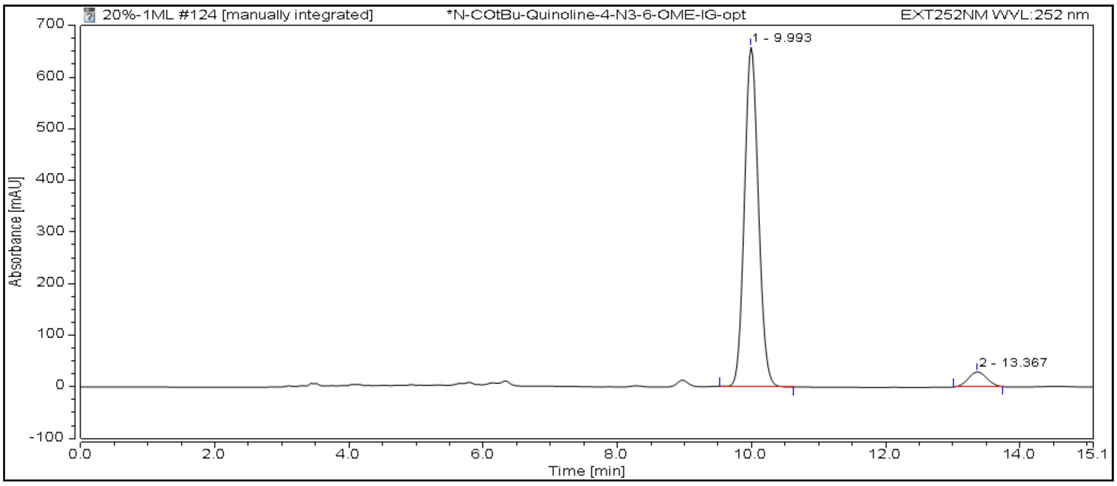

Integration Results					
No.	Peak Name	Retention Time min	Area mAU*min	Relative Area %	Amount n.a.
1		9.993	159.070	94.87	n.a.
2		13.367	8.598	5.13	n.a.
**Total:**			**167.668**	**100.00**	

Supplementary figure 54. HPLC chromatogram for **1c**

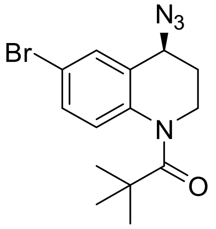

(*S*)-1-(4-Azido-6-bromo-3,4-dihydroquinolin-1(2*H*)-yl)-2,2-dimethylpropan-1-one (1d)

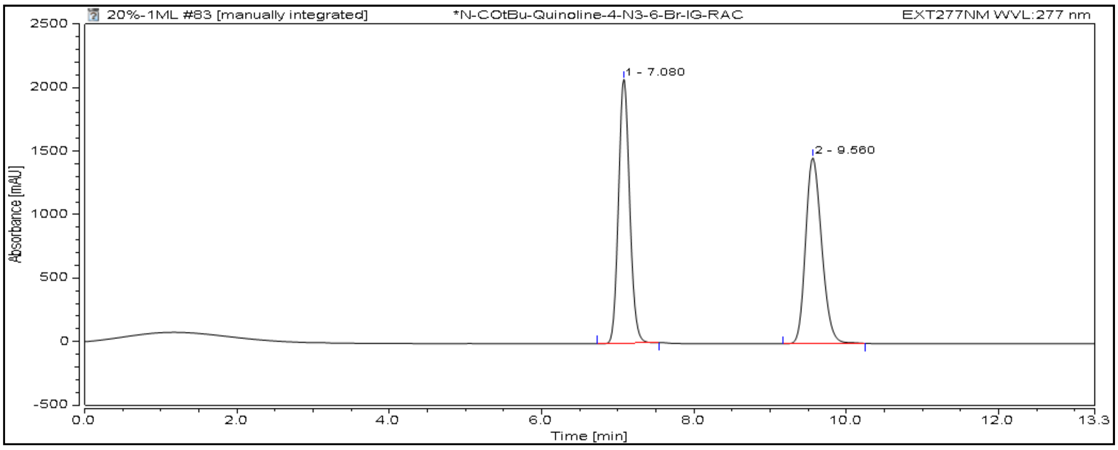

Integration Results					
No.	Peak Name	Retention Time min	Area mAU*min	Relative Area %	Amount n.a.
1		7.080	351.441	49.59	n.a.
2		9.560	357.237	50.41	n.a.
**Total:**			**708.678**	**100.00**	

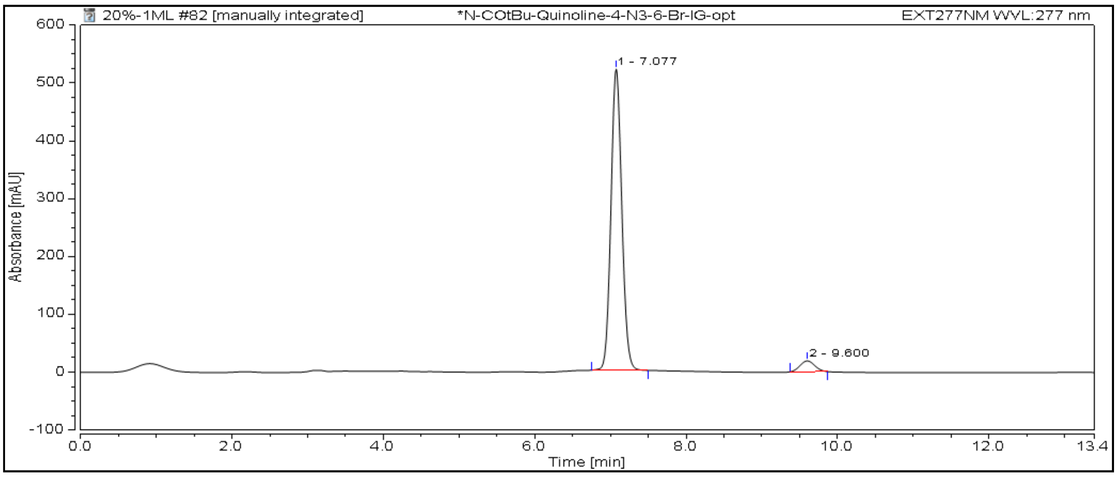

Integration Results					
No.	Peak Name	Retention Time min	Area mAU*min	Relative Area %	Amount n.a.
1		7.077	86.273	95.32	n.a.
2		9.600	4.235	4.68	n.a.
**Total:**			**90.509**	**100.00**	

Supplementary figure 55. HPLC chromatogram for **1d**

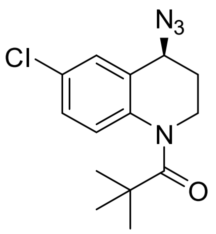

(*S*)-1-(4-Azido-6-chloro-3,4-dihydroquinolin-1(2*H*)-yl)-2,2-dimethylpropan-1-one (1e)

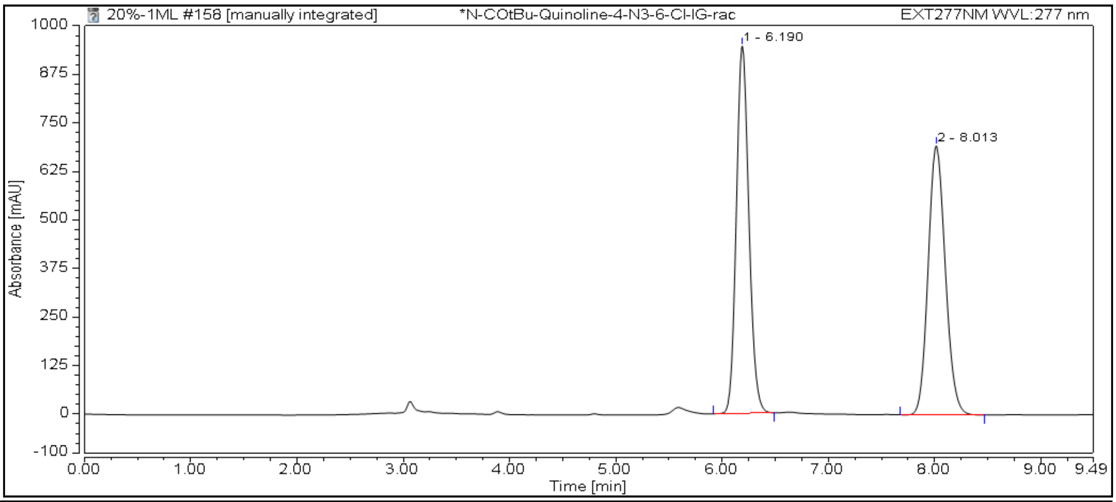

Integration Results					
No.	Peak Name	Retention Time<br>min	Area<br>mAU*min	Relative Area<br>%	Amount<br>n.a.
1		6.190	129.534	49.94	n.a.
2		8.013	129.842	50.06	n.a.
**Total:**			**259.375**	**100.00**	

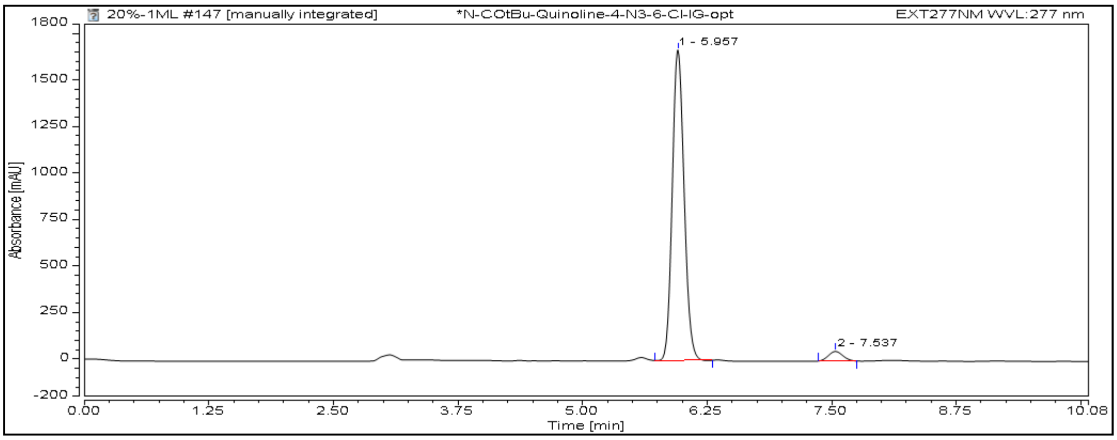

Integration Results					
No.	Peak Name	Retention Time<br>min	Area<br>mAU*min	Relative Area<br>%	Amount<br>n.a.
1		5.957	229.788	96.75	n.a.
2		7.537	7.713	3.25	n.a.
**Total:**			**237.501**	**100.00**	

Supplementary figure 56. HPLC chromatogram for **1e**

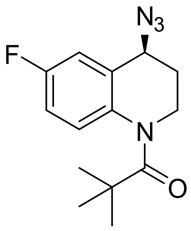

(*S*)-1-(4-Azido-6-fluoro-3,4-dihydroquinolin-1(2*H*)-yl)-2,2-dimethylpropan-1-one (1f)

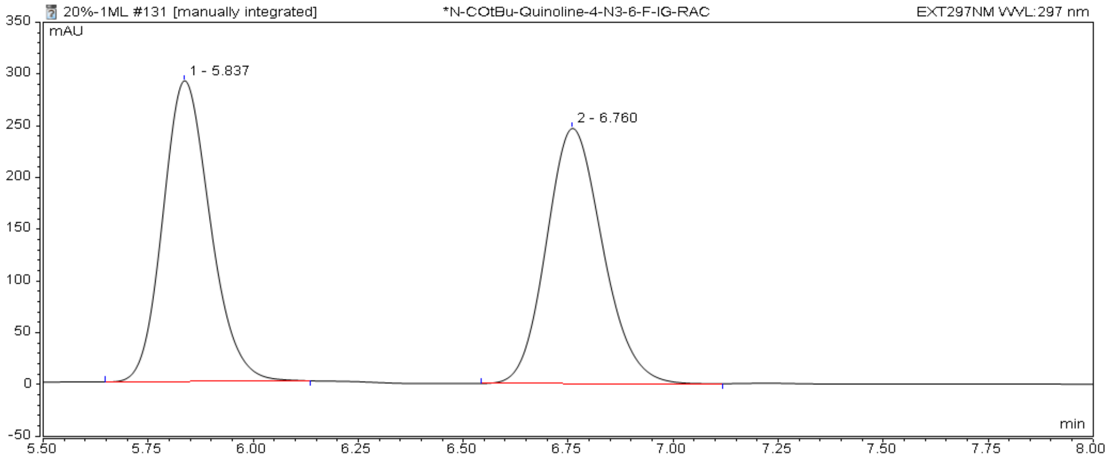

Integration Results					
No.	Peak Name	Retention Time min	Area mAU*min	Relative Area %	Amount n.a.
1		5.837	37.984	49.80	n.a.
2		6.760	38.289	50.20	n.a.
**Total:**			**76.274**	**100.00**	

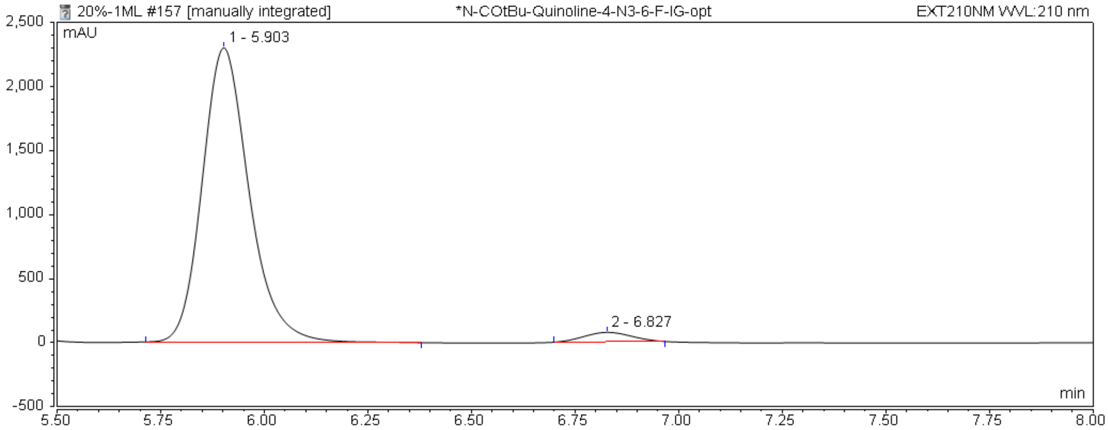

Integration Results					
No.	Peak Name	Retention Time min	Area mAU*min	Relative Area %	Amount n.a.
1		5.903	301.317	96.86	n.a.
2		6.827	9.775	3.14	n.a.
**Total:**			**311.092**	**100.00**	

Supplementary figure 57. HPLC chromatogram for **1f**

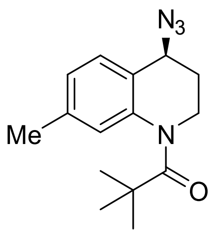

(*S*)-1-(4-Azido-7-methyl-3,4-dihydroquinolin-1(2*H*)-yl)-2,2-dimethylpropan-1-one (1g)

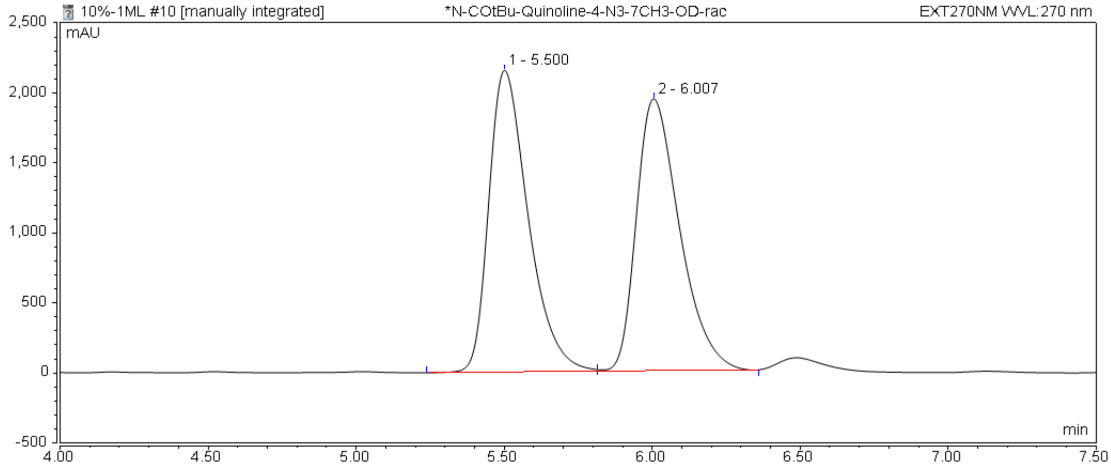

Integration Results					
No.	Peak Name	Retention Time<br>min	Area<br>mAU*min	Relative Area<br>%	Amount<br>n.a.
1		5.500	332.133	50.31	n.a.
2		6.007	327.998	49.69	n.a.
**Total:**			**660.131**	**100.00**	

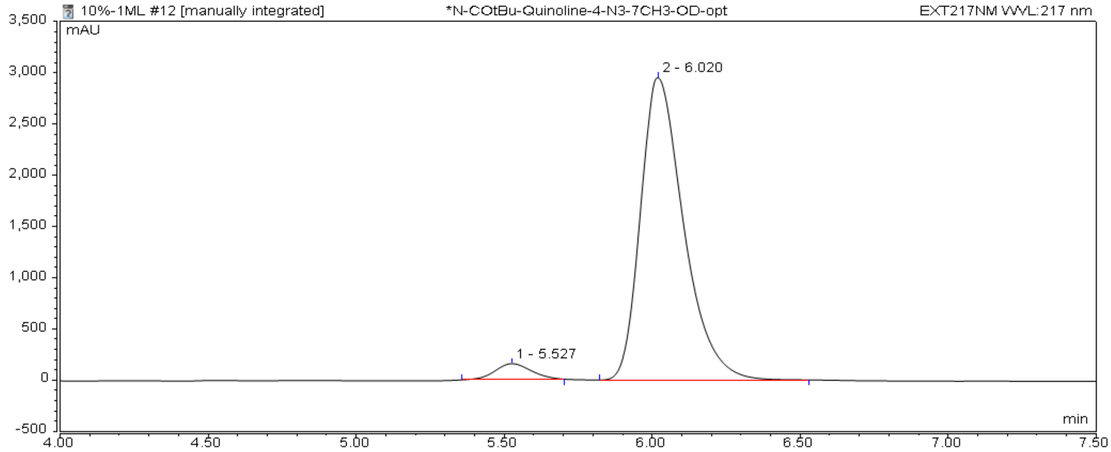

Integration Results					
No.	Peak Name	Retention Time<br>min	Area<br>mAU*min	Relative Area<br>%	Amount<br>n.a.
1		5.527	22.024	4.23	n.a.
2		6.020	498.839	95.77	n.a.
**Total:**			**520.863**	**100.00**	

Supplementary figure 58. HPLC chromatogram for **1g**

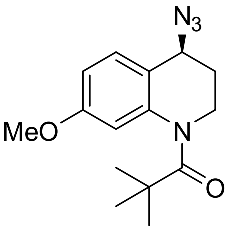

(*S*)-1-(4-Azido-7-methoxy-3,4-dihydroquinolin-1(2*H*)-yl)-2,2-dimethylpropan-1-one (1h)

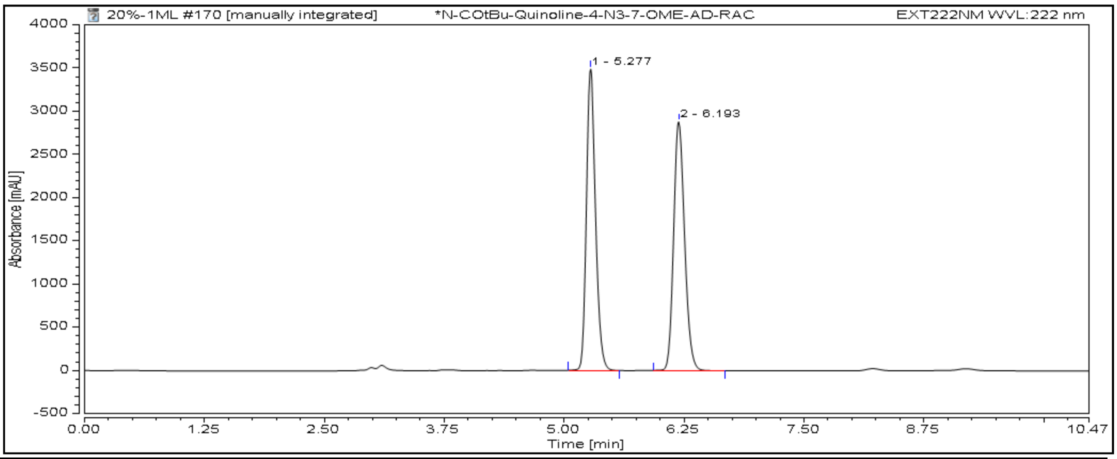

Integration Results

No.	Peak Name	Retention Time min	Area mAU*min	Relative Area %	Amount n.a.
1		5.277	382.554	50.14	n.a.
2		6.193	380.441	49.86	n.a.
**Total:**			**762.995**	**100.00**	

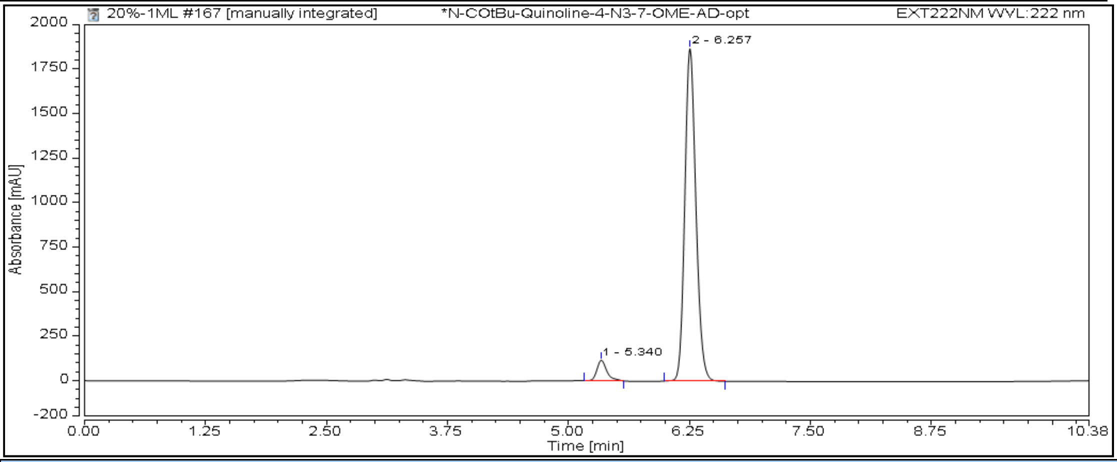

Integration Results

No.	Peak Name	Retention Time min	Area mAU*min	Relative Area %	Amount n.a.
1		5.340	11.663	4.50	n.a.
2		6.257	247.399	95.50	n.a.
**Total:**			**259.062**	**100.00**	

Supplementary figure 59. HPLC chromatogram for **1h**

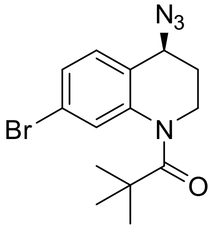

(*S*)-1-(4-Azido-7-bromo-3,4-dihydroquinolin-1(2*H*)-yl)-2,2-dimethylpropan-1-one (1i)

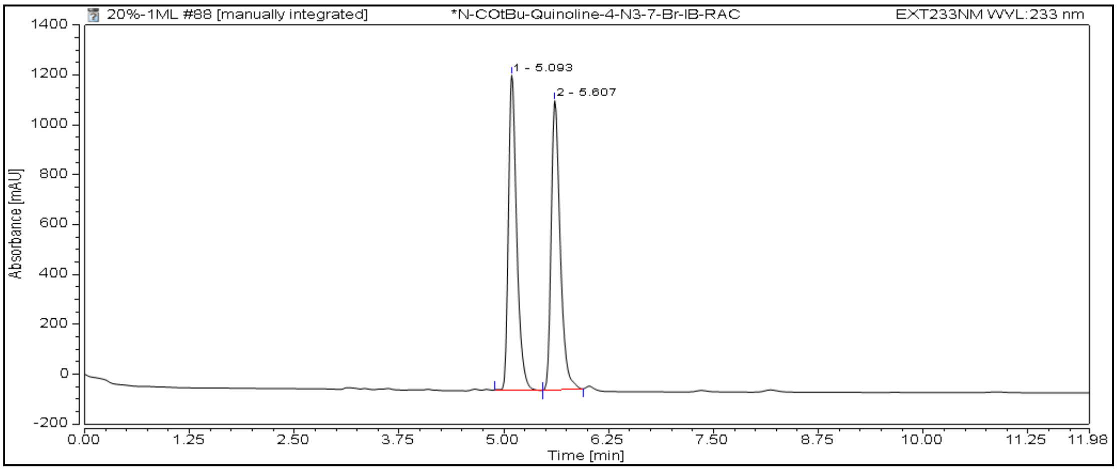

Integration Results					
No.	Peak Name	Retention Time<br>min	Area<br>mAU*min	Relative Area<br>%	Amount<br>n.a.
1		5.093	144.523	50.02	n.a.
2		5.607	144.413	49.98	n.a.
**Total:**			**288.937**	**100.00**	

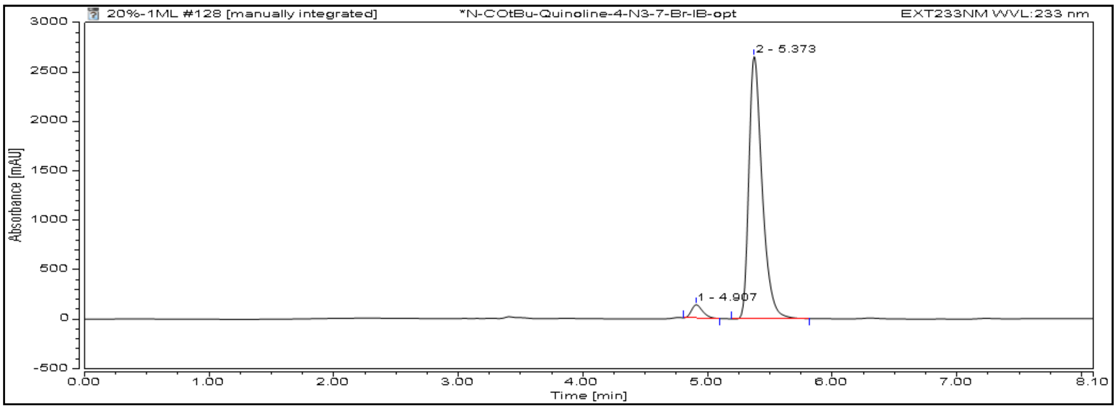

Integration Results					
No.	Peak Name	Retention<br>Time<br>min	Area<br>mAU*min	Relative<br>Area<br>%	Amount<br>n.a.
1		4.907	13.889	4.08	n.a.
2		5.373	326.517	95.92	n.a.
**Total:**			**340.407**	**100.00**	

Supplementary figure 60. HPLC chromatogram for **1i**

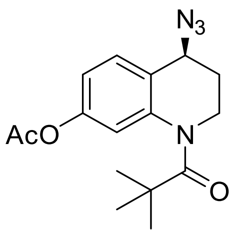

(*S*)-4-Azido-1-pivaloyl-1,2,3,4-tetrahydroquinolin-7-yl acetate (1j)

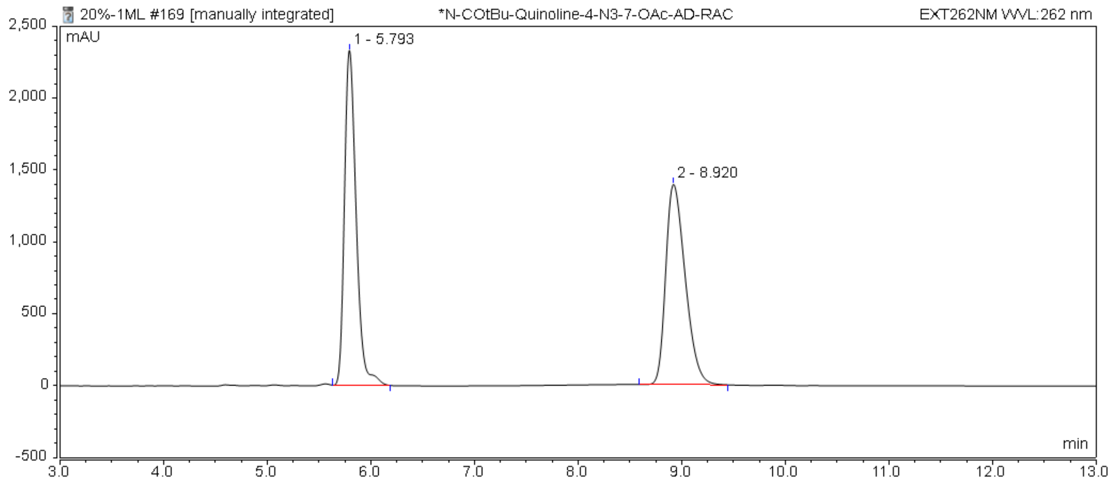

Integration Results					
No.	Peak Name	Retention Time<br>min	Area<br>mAU*min	Relative Area<br>%	Amount<br>n.a.
1		5.793	304.160	50.02	n.a.
2		8.920	303.877	49.98	n.a.
**Total:**			**608.037**	**100.00**	

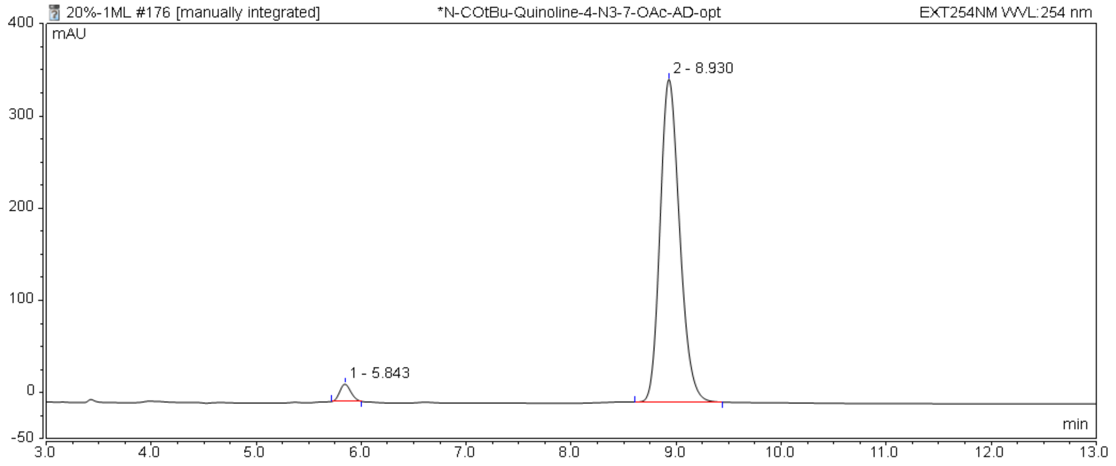

Integration Results					
No.	Peak Name	Retention Time<br>min	Area<br>mAU*min	Relative Area<br>%	Amount<br>n.a.
1		5.843	2.310	2.97	n.a.
2		8.930	75.456	97.03	n.a.
**Total:**			**77.767**	**100.00**	

Supplementary figure 61. HPLC chromatogram for **1j**

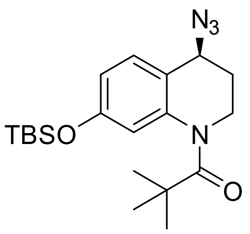

(*S*)-1-(4-Azido-7-((tert-butyldimethylsilyl)oxy)-3,4-dihydroquinolin-1(2*H*)-yl)-2,2 -dimethylpropan-1-one (1k)

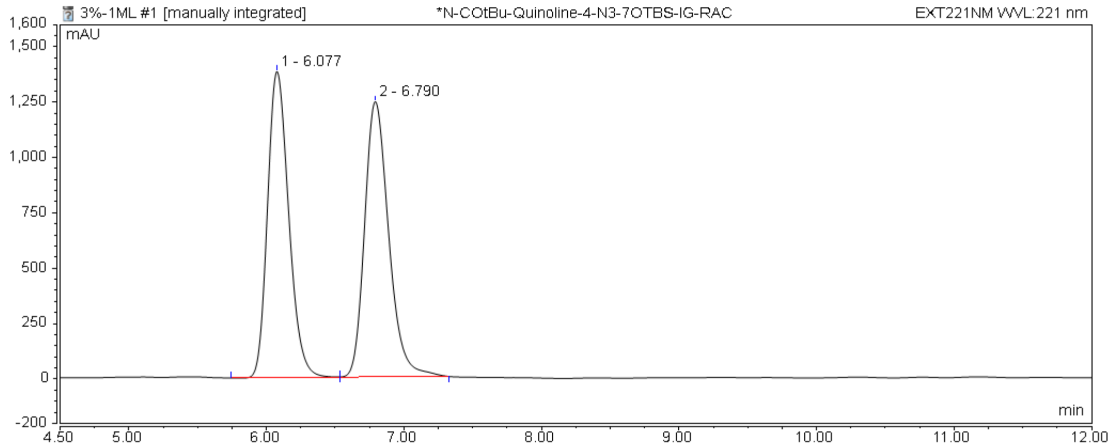

**Integration Results**					
No.	Peak Name	Retention Time<br>min	Area<br>mAU*min	Relative Area<br>%	Amount<br>n.a.
1		6.077	246.465	49.44	n.a.
2		6.790	252.025	50.56	n.a.
**Total:**			**498.489**	**100.00**	

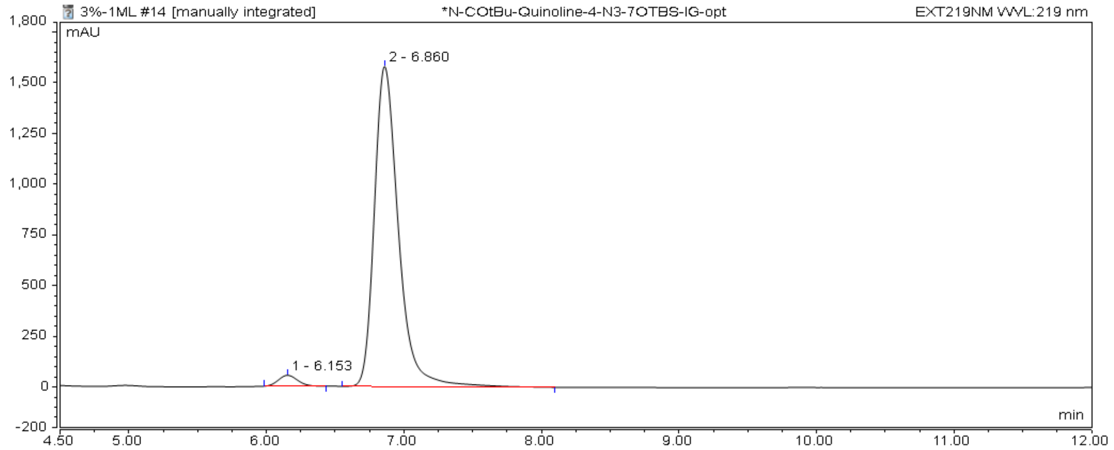

**Integration Results**					
No.	Peak Name	Retention Time<br>min	Area<br>mAU*min	Relative Area<br>%	Amount<br>n.a.
1		6.153	8.555	2.60	n.a.
2		6.860	320.397	97.40	n.a.
**Total:**			**328.952**	**100.00**	

Supplementary figure 62. HPLC chromatogram for **1k**

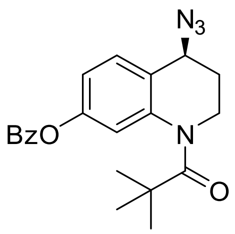

(*S*)-4-Azido-1-pivaloyl-1,2,3,4-tetrahydroquinolin-7-yl benzoate (1l)

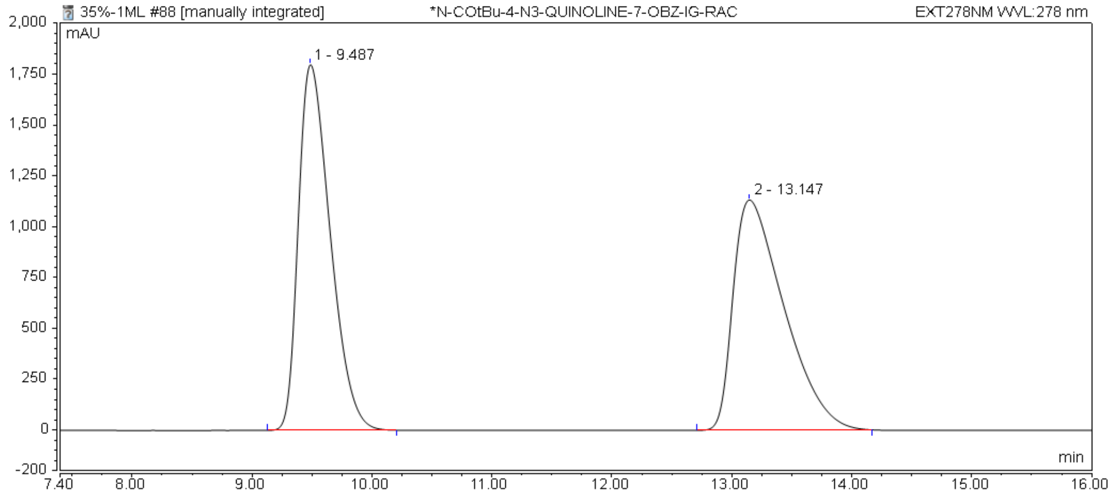

Integration Results

No.	Peak Name	Retention Time min	Area mAU*min	Relative Area %	Amount n.a.
1		9.487	553.019	49.84	n.a.
2		13.147	556.500	50.16	n.a.
**Total:**			**1109.519**	**100.00**	

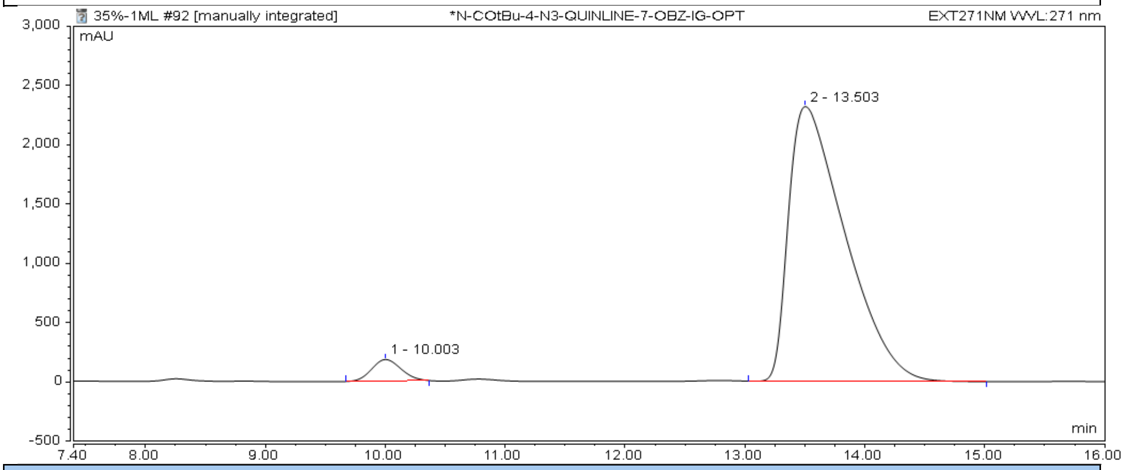

Integration Results

No.	Peak Name	Retention Time min	Area mAU*min	Relative Area %	Amount n.a.
1		10.003	51.276	3.89	n.a.
2		13.503	1266.166	96.11	n.a.
**Total:**			**1317.442**	**100.00**	

Supplementary figure 63. HPLC chromatogram for **1l**

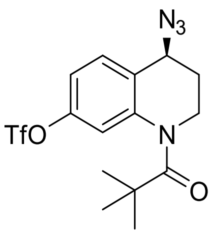

(*S*)-4-Azido-1-pivaloyl-1,2,3,4-tetrahydroquinolin-7-yl trifluoromethanesulfonate (1m)

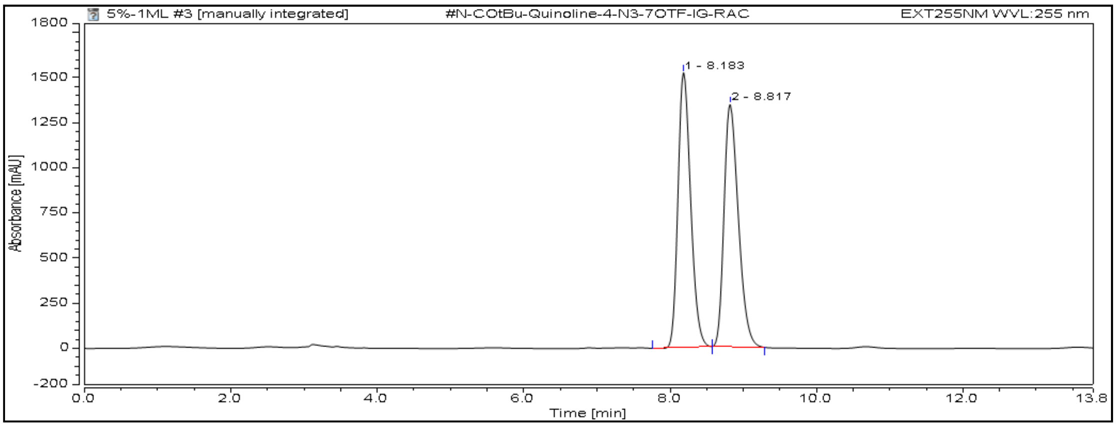

Integration Results					
No.	Peak Name	Retention Time min	Area mAU*min	Relative Area %	Amount n.a.
1		8.183	304.557	50.04	n.a.
2		8.817	304.039	49.96	n.a.
**Total:**			**608.596**	**100.00**	

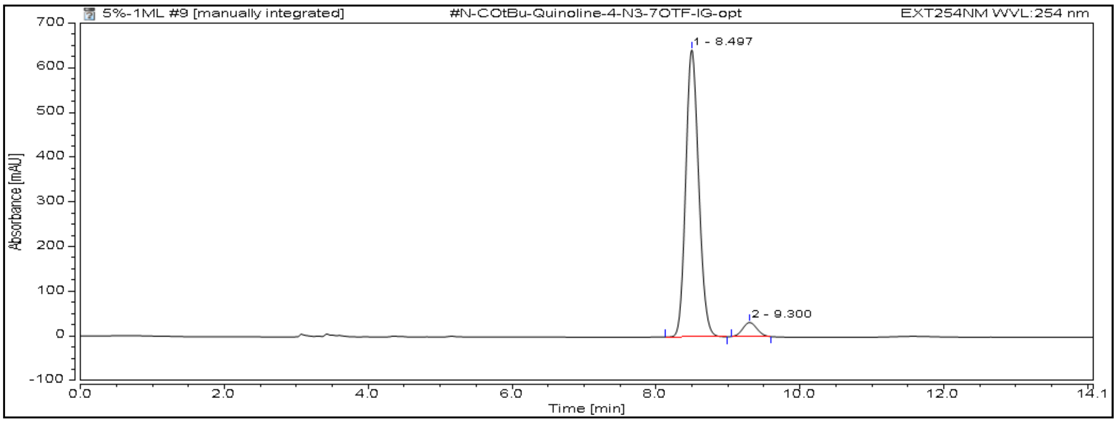

Integration Results					
No.	Peak Name	Retention Time min	Area mAU*min	Relative Area %	Amount n.a.
1		8.497	134.187	94.89	n.a.
2		9.300	7.225	5.11	n.a.
**Total:**			**141.412**	**100.00**	

Supplementary figure 64. HPLC chromatogram for **1m**

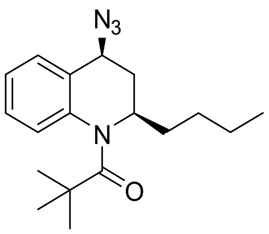

1-((2*R*,4*S*)-4-Azido-2-butyl-3,4-dihydroquinolin-1(2*H*)-yl)-2,2-dimethylpropan-1-one (1n)

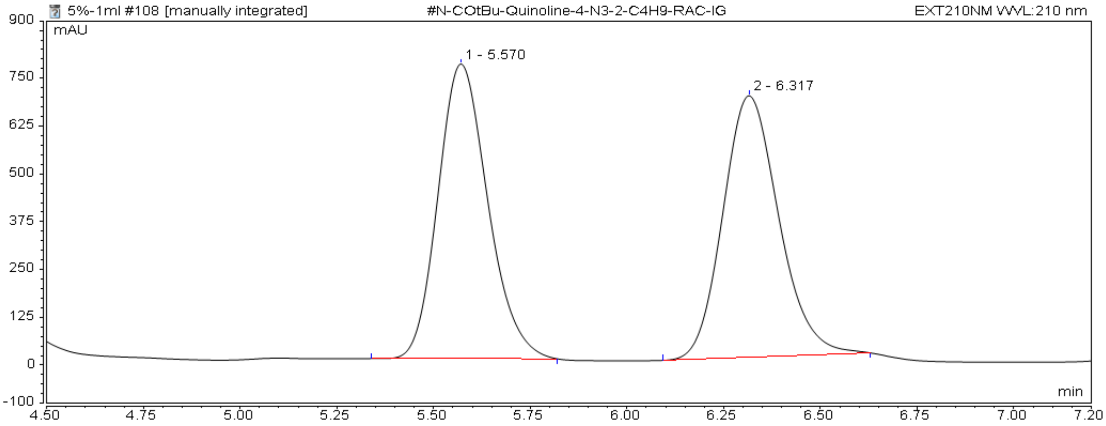

Integration Results					
No.	Peak Name	Retention Time min	Area mAU*min	Relative Area %	Amount n.a.
1		5.570	114.960	50.08	n.a.
2		6.317	114.584	49.92	n.a.
**Total:**			**229.544**	**100.00**	

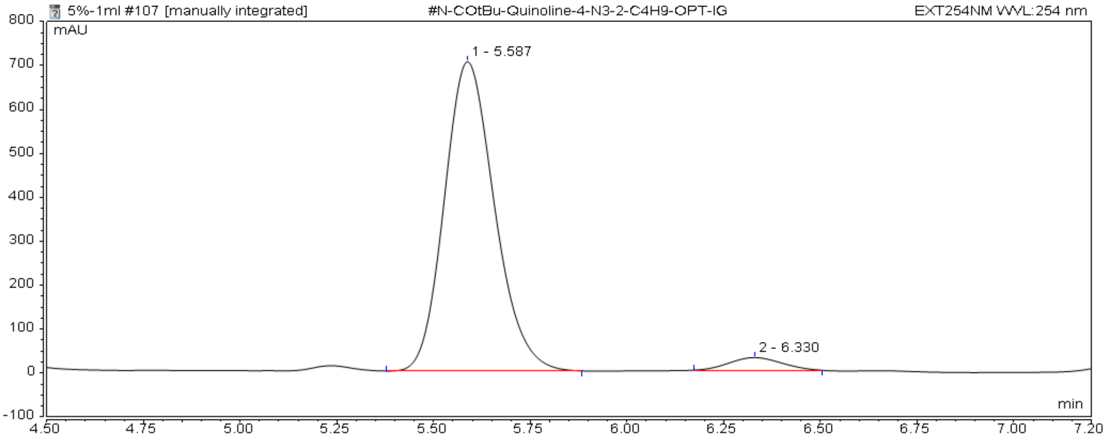

Integration Results					
No.	Peak Name	Retention Time min	Area mAU*min	Relative Area %	Amount n.a.
1		5.587	105.760	95.92	n.a.
2		6.330	4.502	4.08	n.a.
**Total:**			**110.262**	**100.00**	

Supplementary figure 65. HPLC chromatogram for **1n**

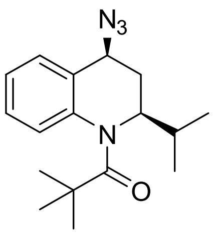

1-((2*S*,4*S*)-4-Azido-2-isopropyl-3,4-dihydroquinolin-1(2*H*)-yl)-2,2-dimethylpropan-1-one (1o)

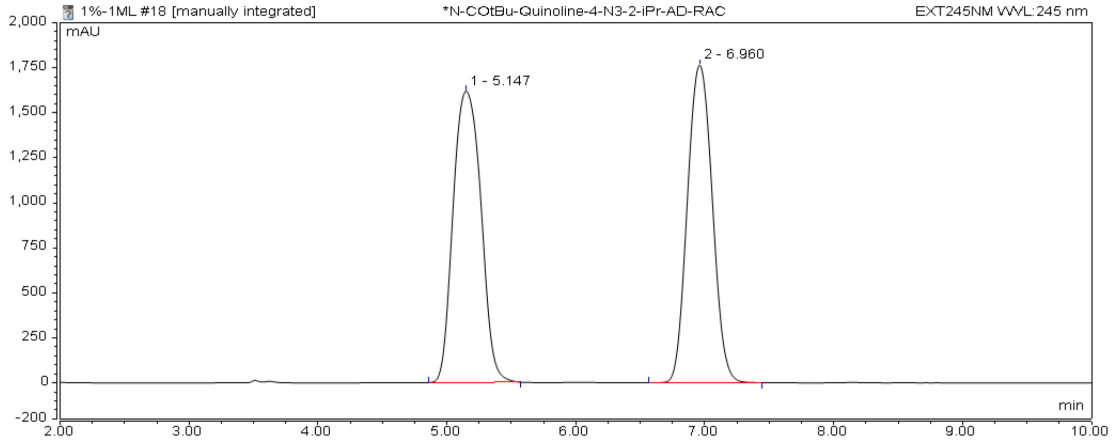

Integration Results					
No.	Peak Name	Retention Time min	Area mAU*min	Relative Area %	Amount n.a.
1		5.147	404.607	50.59	n.a.
2		6.960	395.228	49.41	n.a.
**Total:**			**799.834**	**100.00**	

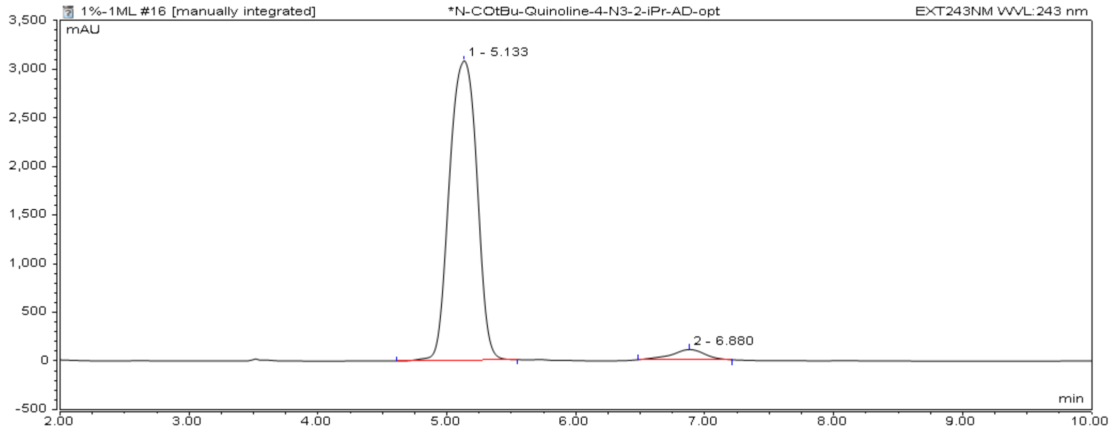

Integration Results					
No.	Peak Name	Retention Time min	Area mAU*min	Relative Area %	Amount n.a.
1		5.133	762.036	95.99	n.a.
2		6.880	31.835	4.01	n.a.
**Total:**			**793.870**	**100.00**	

Supplementary figure 66. HPLC chromatogram for **1o**

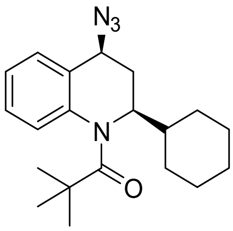

1-((2*S*,4*S*)-4-Azido-2-cyclohexyl-3,4-dihydroquinolin-1(2*H*)-yl)-2,2-dimethylprop an-1-one (1p)

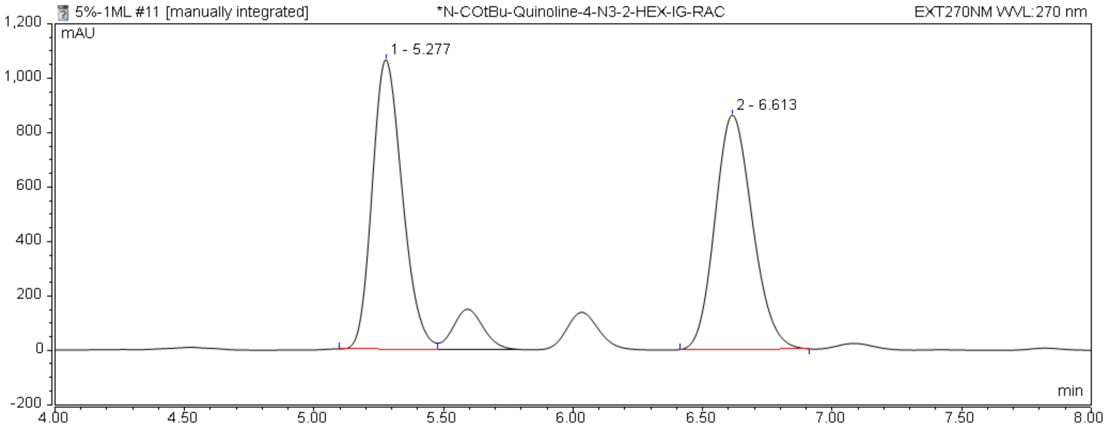

Integration Results					
No.	Peak Name	Retention Time min	Area mAU*min	Relative Area %	Amount n.a.
1		5.277	143.014	49.80	n.a.
2		6.613	144.188	50.20	n.a.
**Total:**			**287.201**	**100.00**	

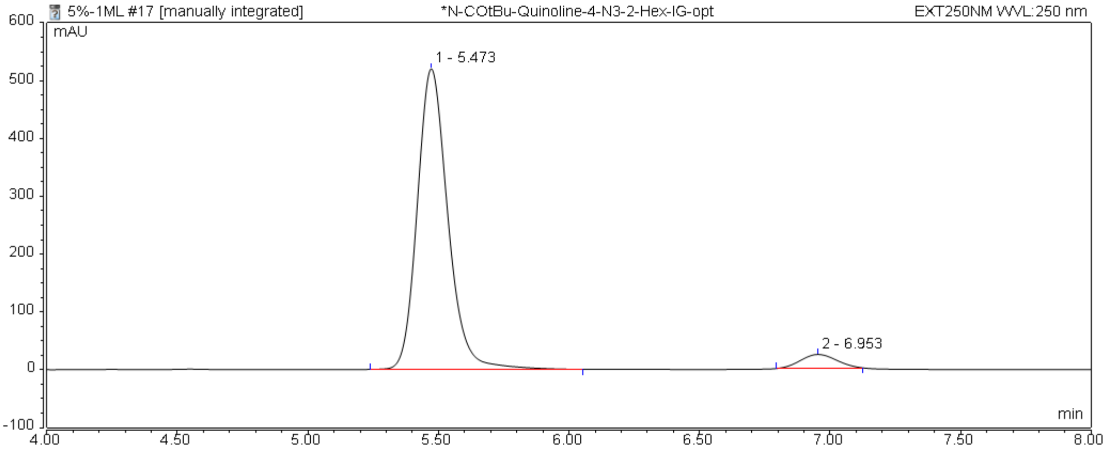

Integration Results					
No.	Peak Name	Retention Time min	Area mAU*min	Relative Area %	Amount n.a.
1		5.473	70.447	94.92	n.a.
2		6.953	3.767	5.08	n.a.
**Total:**			**74.214**	**100.00**	

Supplementary figure 67. HPLC chromatogram for **1p**

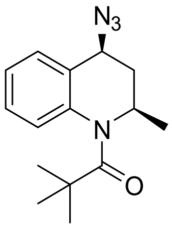

1-((2*R*,4*S*)-4-Azido-2-methyl-3,4-dihydroquinolin-1(2*H*)-yl)-2,2-dimethylpropan-1-one (1q)

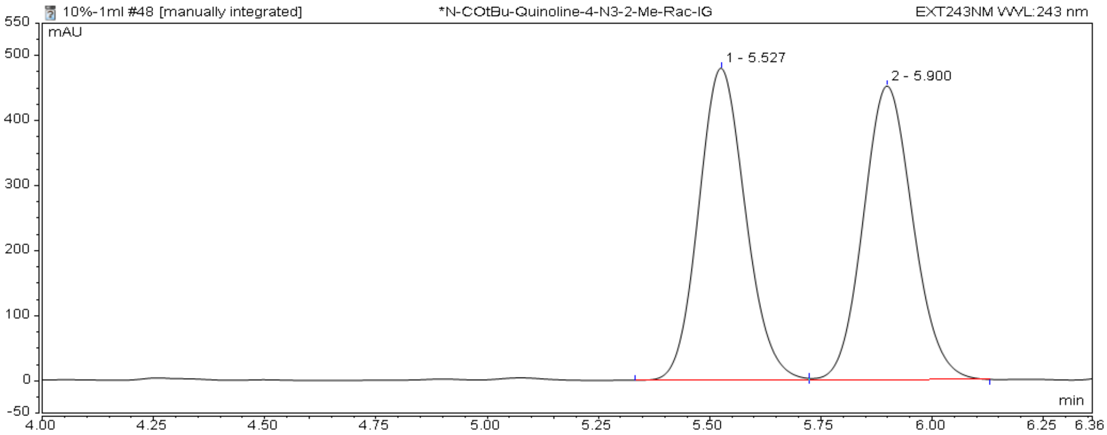

**Integration Results**					
No.	Peak Name	Retention Time min	Area mAU*min	Relative Area %	Amount n.a.
1		5.527	58.419	50.04	n.a.
2		5.900	58.326	49.96	n.a.
**Total:**			**116.745**	**100.00**	

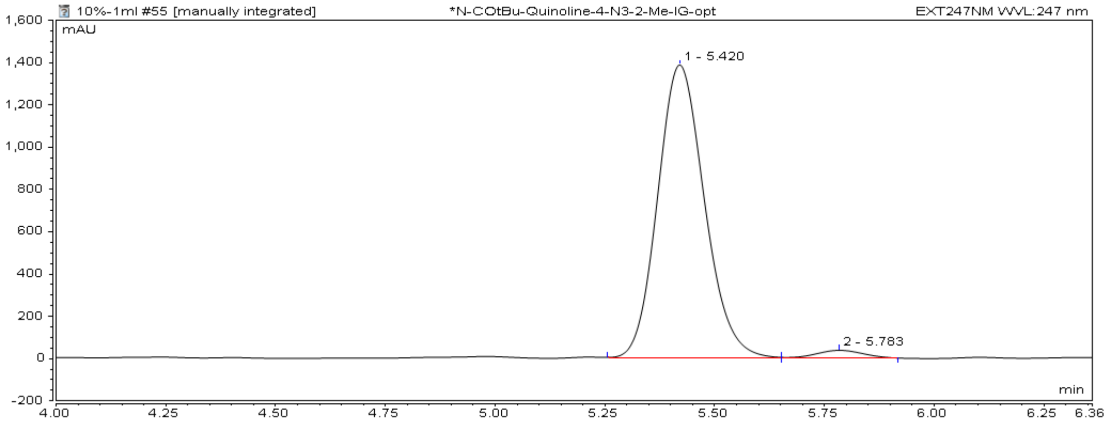

**Integration Results**					
No.	Peak Name	Retention Time min	Area mAU*min	Relative Area %	Amount n.a.
1		5.420	170.184	97.54	n.a.
2		5.783	4.296	2.46	n.a.
**Total:**			**174.480**	**100.00**	

Supplementary figure 68. HPLC chromatogram for **1q**

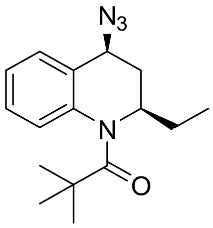

1-((2*R*,4*S*)-4-Azido-2-ethyl-3,4-dihydroquinolin-1(2*H*)-yl)-2,2-dimethylpropan-1-one (1r)

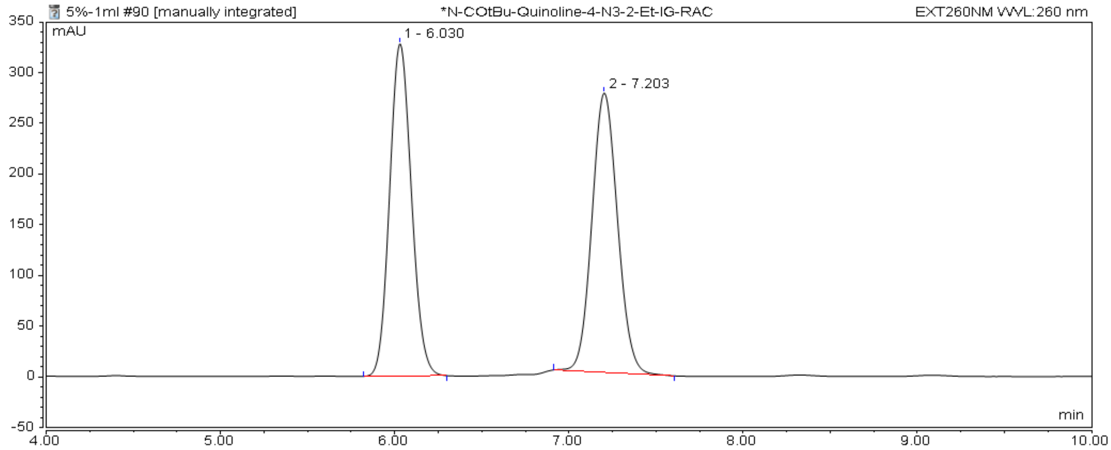

**Integration Results**					
No.	Peak Name	Retention Time<br>min	Area<br>mAU*min	Relative Area<br>%	Amount<br>n.a.
1<br>2		6.030<br>7.203	47.593<br>46.919	50.36<br>49.64	n.a.<br>n.a.
**Total:**			**94.512**	**100.00**	

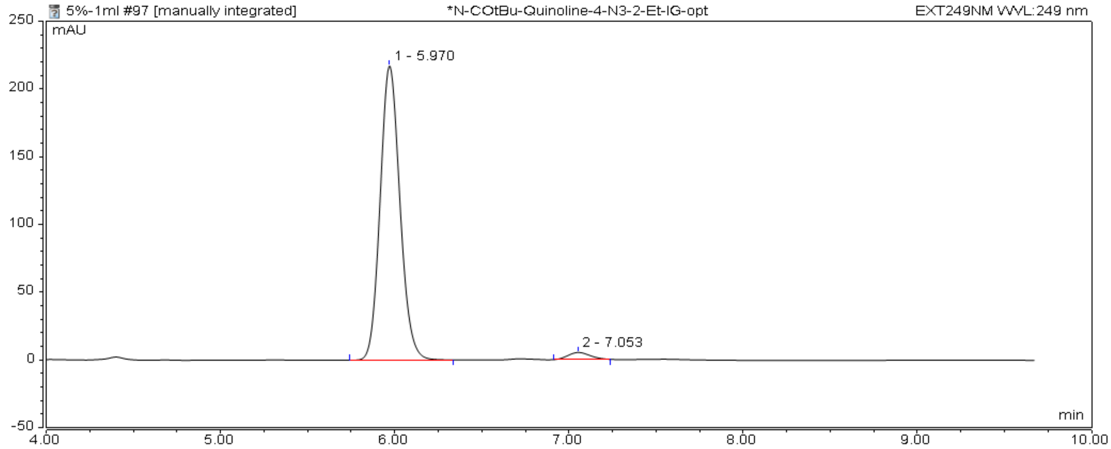

**Integration Results**					
No.	Peak Name	Retention Time<br>min	Area<br>mAU*min	Relative Area<br>%	Amount<br>n.a.
1<br>2		5.970<br>7.053	28.606<br>0.762	97.41<br>2.59	n.a.<br>n.a.
**Total:**			**29.368**	**100.00**	

Supplementary figure 69. HPLC chromatogram for **1r**

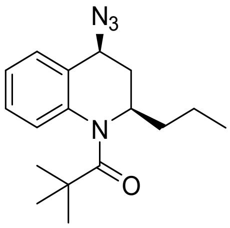

1-((2*R*,4*S*)-4-Azido-2-propyl-3,4-dihydroquinolin-1(2*H*)-yl)-2,2-dimethylpropan-1-one (1s)

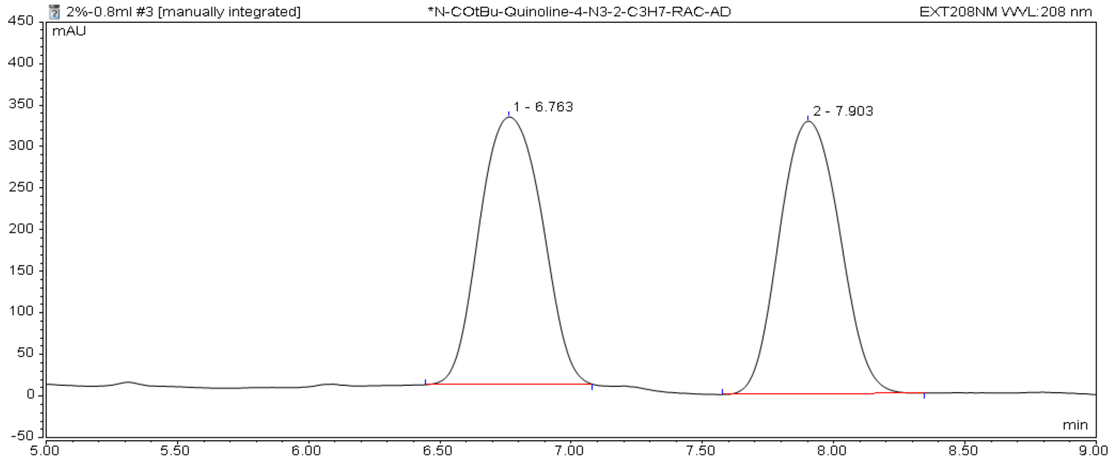

Integration Results					
No.	Peak Name	Retention Time min	Area mAU*min	Relative Area %	Amount n.a.
1		6.763	88.659	50.38	n.a.
2		7.903	87.318	49.62	n.a.
**Total:**			**175.977**	**100.00**	

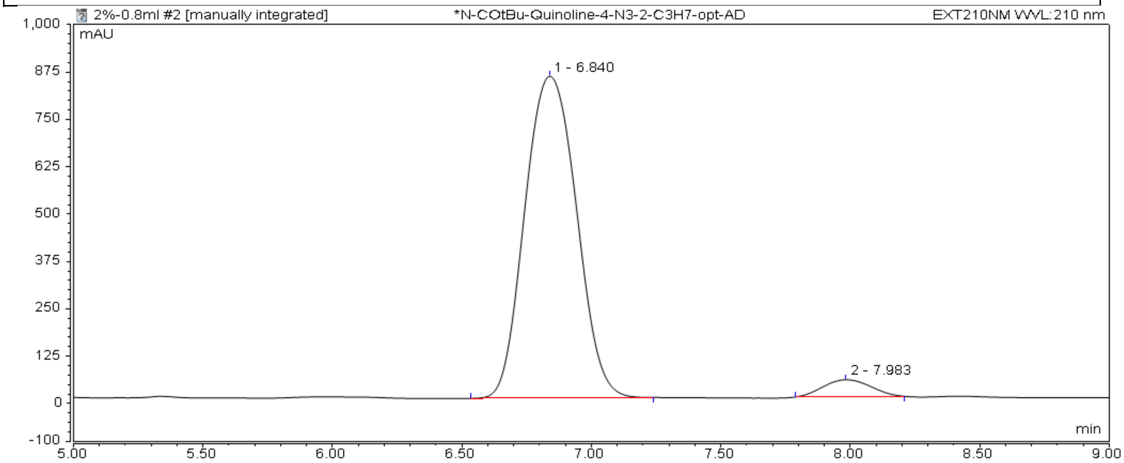

Integration Results					
No.	Peak Name	Retention Time min	Area mAU*min	Relative Area %	Amount n.a.
1		6.840	196.300	95.42	n.a.
2		7.983	9.424	4.58	n.a.
**Total:**			**205.724**	**100.00**	

Supplementary figure 70. HPLC chromatogram for **1s**

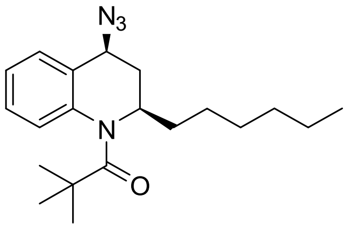

1-((2*R*,4*S*)-4-Azido-2-hexyl-3,4-dihydroquinolin-1(2*H*)-yl)-2,2-dimethylpropan-1-one (1t)

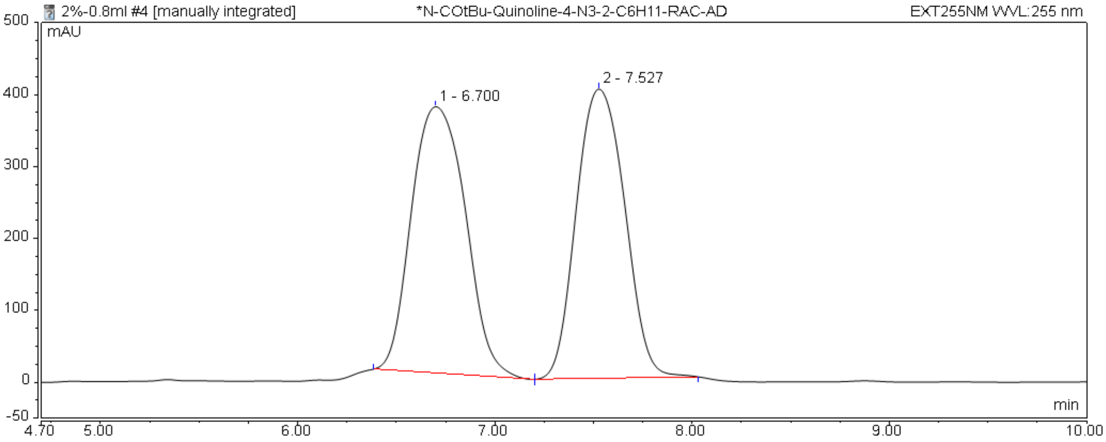

Integration Results					
No.	Peak Name	Retention Time<br>min	Area<br>mAU*min	Relative Area<br>%	Amount<br>n.a.
1		6.700	117.854	50.68	n.a.
2		7.527	114.698	49.32	n.a.
**Total:**			**232.552**	**100.00**	

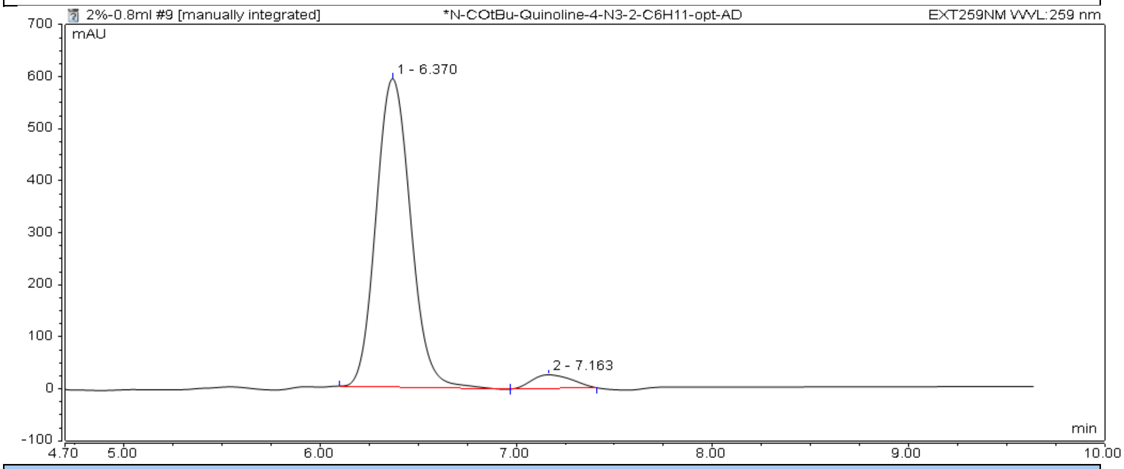

Integration Results					
No.	Peak Name	Retention Time<br>min	Area<br>mAU*min	Relative Area<br>%	Amount<br>n.a.
1		6.370	121.186	95.10	n.a.
2		7.163	6.246	4.90	n.a.
**Total:**			**127.432**	**100.00**	

Supplementary figure 71. HPLC chromatogram for **1t**

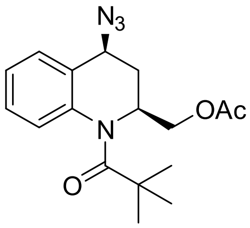

((2*S*,4*S*)-4-Azido-1-pivaloyl-1,2,3,4-tetrahydroquinolin-2-yl)methyl acetate (1u)

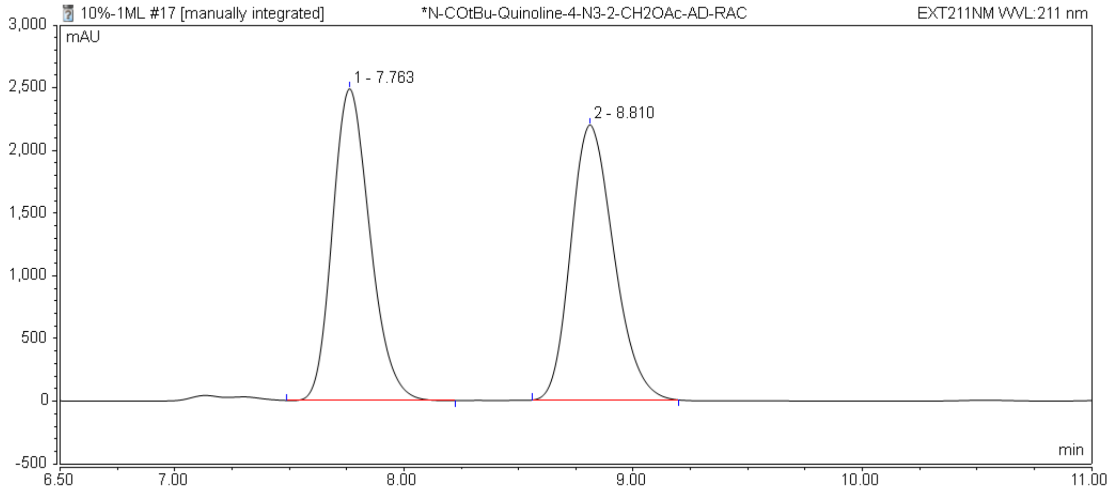

Integration Results					
No.	Peak Name	Retention Time<br>min	Area<br>mAU*min	Relative Area<br>%	Amount<br>n.a.
1		7.763	470.509	49.65	n.a.
2		8.810	477.230	50.35	n.a.
**Total:**			**947.739**	**100.00**	

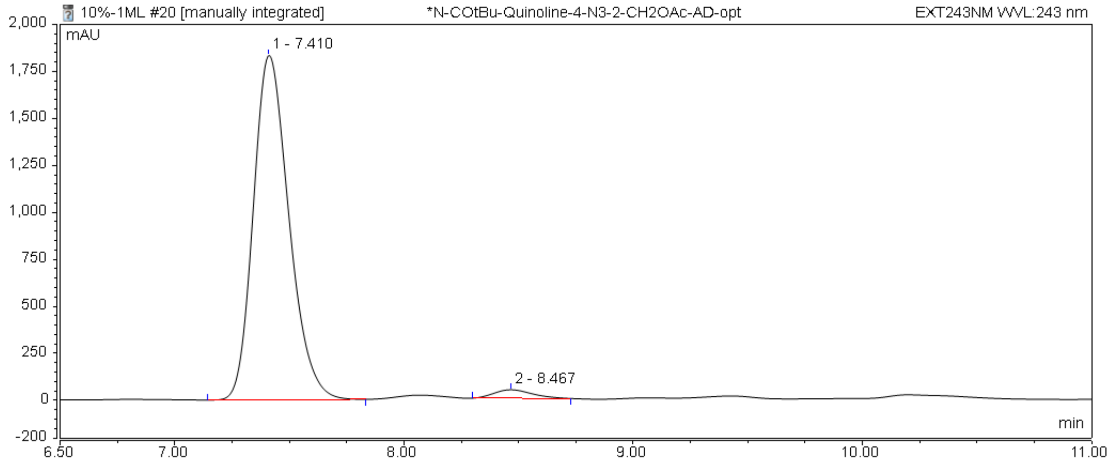

Integration Results					
No.	Peak Name	Retention Time<br>min	Area<br>mAU*min	Relative Area<br>%	Amount<br>n.a.
1		7.410	329.457	97.41	n.a.
2		8.467	8.771	2.59	n.a.
**Total:**			**338.227**	**100.00**	

Supplementary figure 72. HPLC chromatogram for **1u**

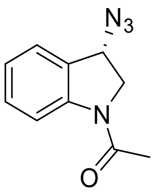

(*S*)-1-(3-Azidoindolin-1-yl)ethan-1-one (3a)

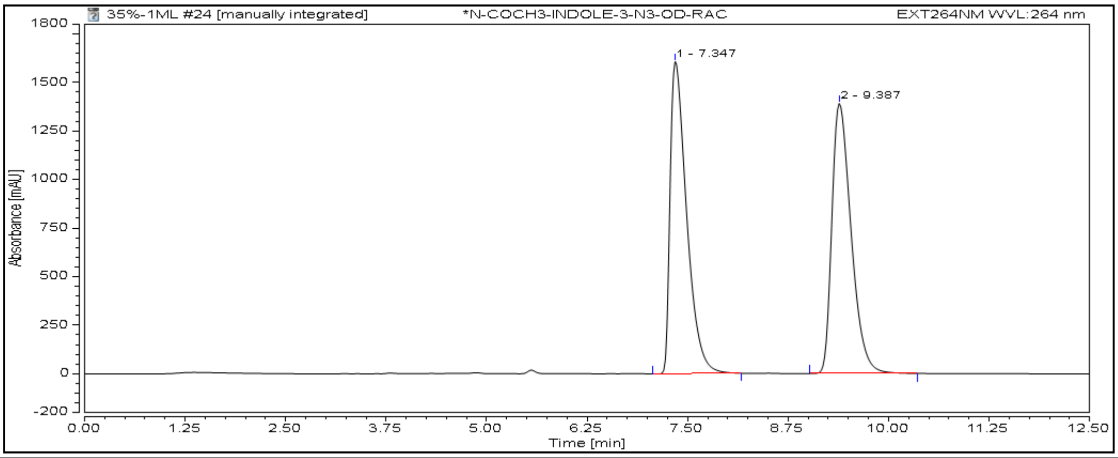

Integration Results					
No.	Peak Name	Retention Time<br>min	Area<br>mAU*min	Relative Area<br>%	Amount<br>n.a.
1		7.347	382.570	49.82	n.a.
2		9.387	385.396	50.18	n.a.
**Total:**			**767.966**	**100.00**	

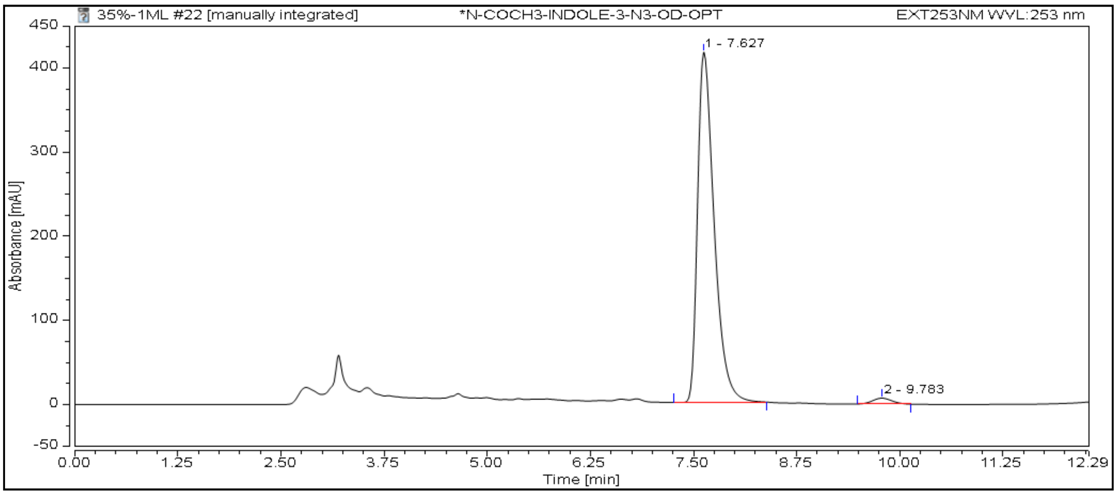

Integration Results					
No.	Peak Name	Retention Time<br>min	Area<br>mAU*min	Relative Area<br>%	Amount<br>n.a.
1		7.627	96.795	98.06	n.a.
2		9.783	1.918	1.94	n.a.
**Total:**			**98.713**	**100.00**	

Supplementary figure 73. HPLC chromatogram for **3a**

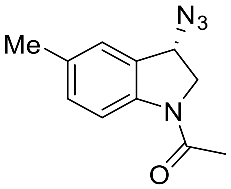

(*S*)-1-(3-Azido-5-methylindolin-1-yl)ethan-1-one (3b)

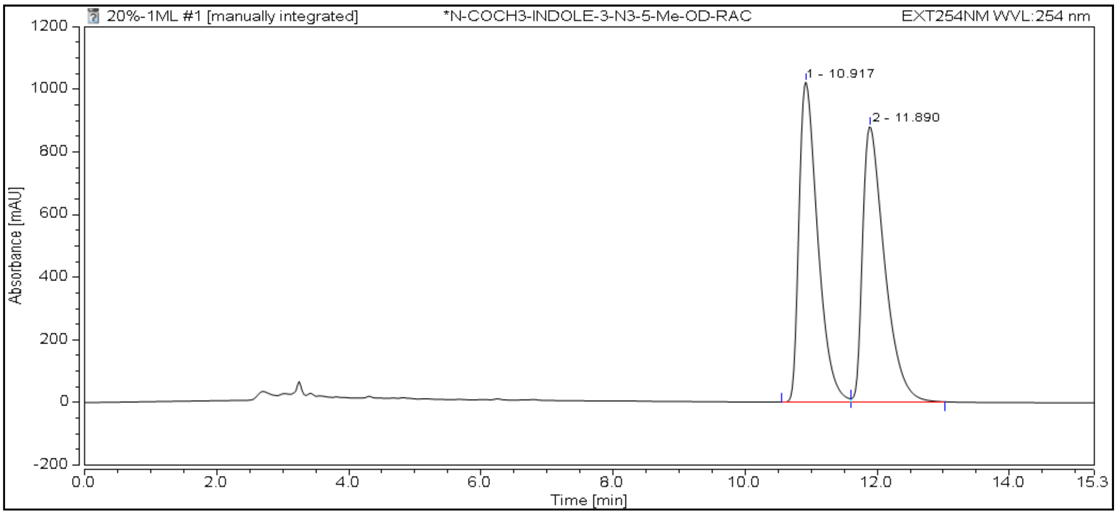

Integration Results					
No.	Peak Name	Retention Time<br>min	Area<br>mAU*min	Relative Area<br>%	Amount<br>n.a.
1		10.917	335.839	49.83	n.a.
2		11.890	338.170	50.17	n.a.
**Total:**			**674.009**	**100.00**	

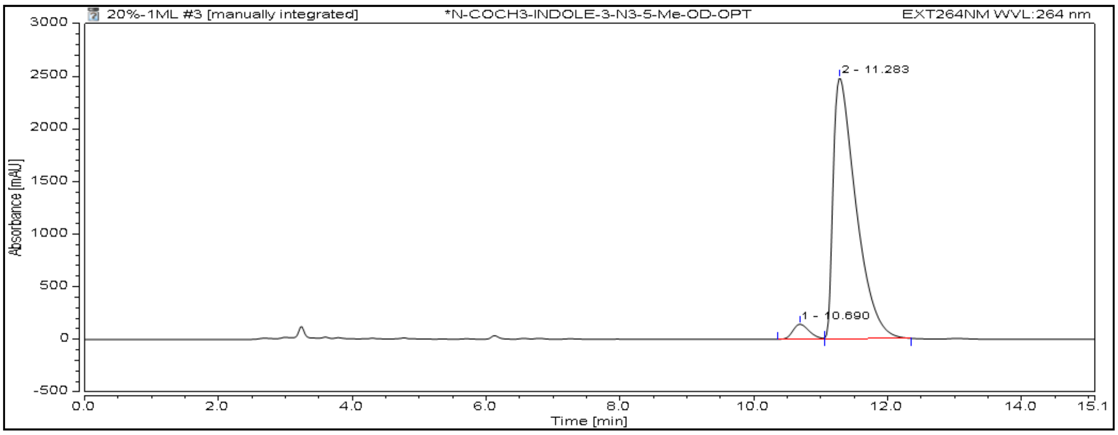

Integration Results					
No.	Peak Name	Retention Time<br>min	Area<br>mAU*min	Relative Area<br>%	Amount<br>n.a.
1		10.690	39.904	3.91	n.a.
2		11.283	979.466	96.09	n.a.
**Total:**			**1019.370**	**100.00**	

Supplementary figure 74. HPLC chromatogram for **3b**

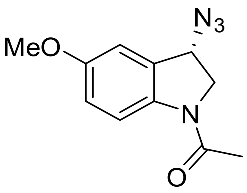

(*S*)-1-(3-Azido-5-methoxyindolin-1-yl)ethan-1-one (3c)

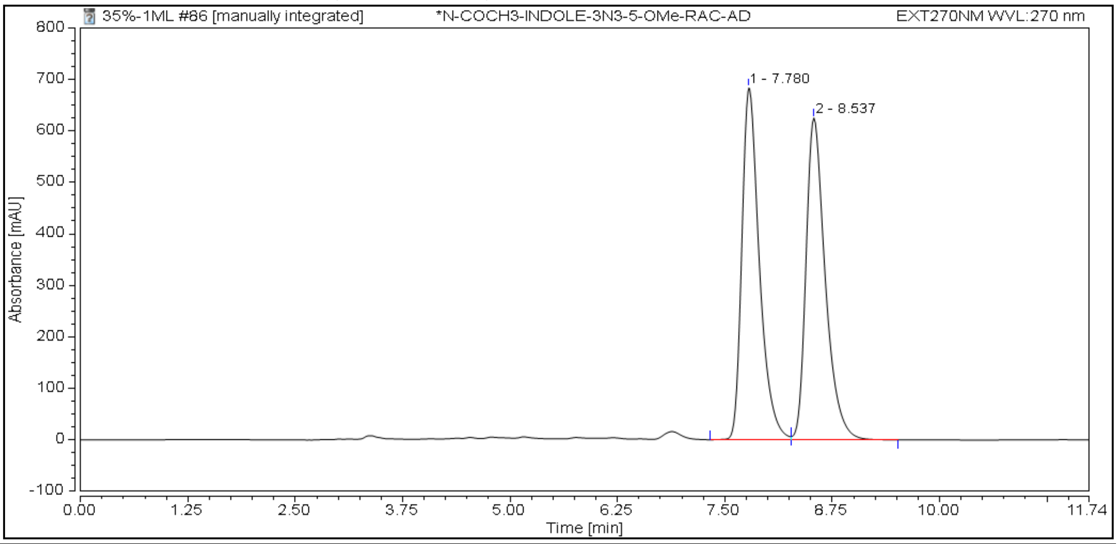

Integration Results

No.	Peak Name	Retention Time min	Area mAU*min	Relative Area %	Amount n.a.
1		7.780	161.556	49.82	n.a.
2		8.537	162.746	50.18	n.a.
**Total:**			**324.302**	**100.00**	

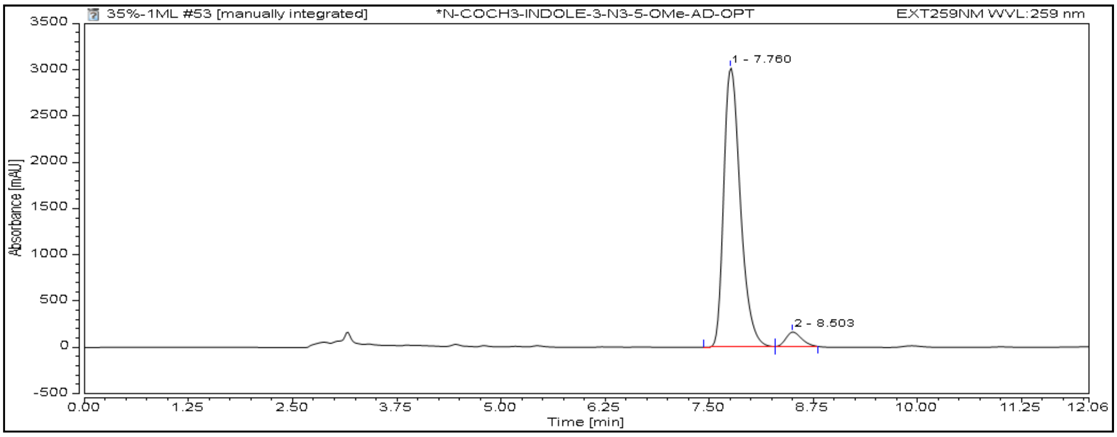

Integration Results

No.	Peak Name	Retention Time min	Area mAU*min	Relative Area %	Amount n.a.
1		7.760	699.945	95.32	n.a.
2		8.503	34.397	4.68	n.a.
**Total:**			**734.342**	**100.00**	

Supplementary figure 75. HPLC chromatogram for **3c**

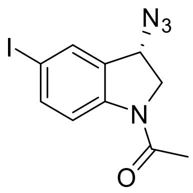

(*S*)-1-(3-Azido-5-iodoindolin-1-yl)ethan-1-one (3d)

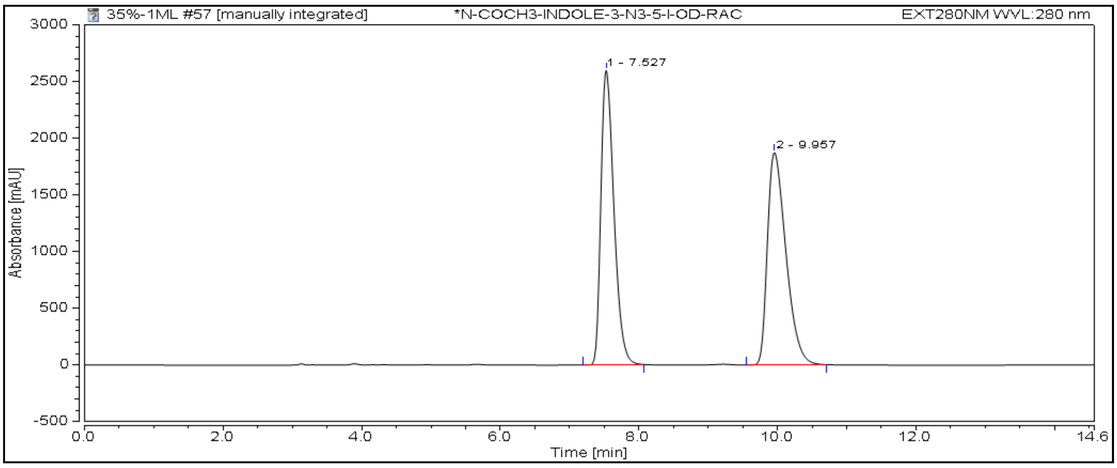

Integration Results					
No.	Peak Name	Retention Time<br>min	Area<br>mAU*min	Relative Area<br>%	Amount<br>n.a.
1		7.527	564.194	49.37	n.a.
2		9.957	578.624	50.63	n.a.
**Total:**			**1142.818**	**100.00**	

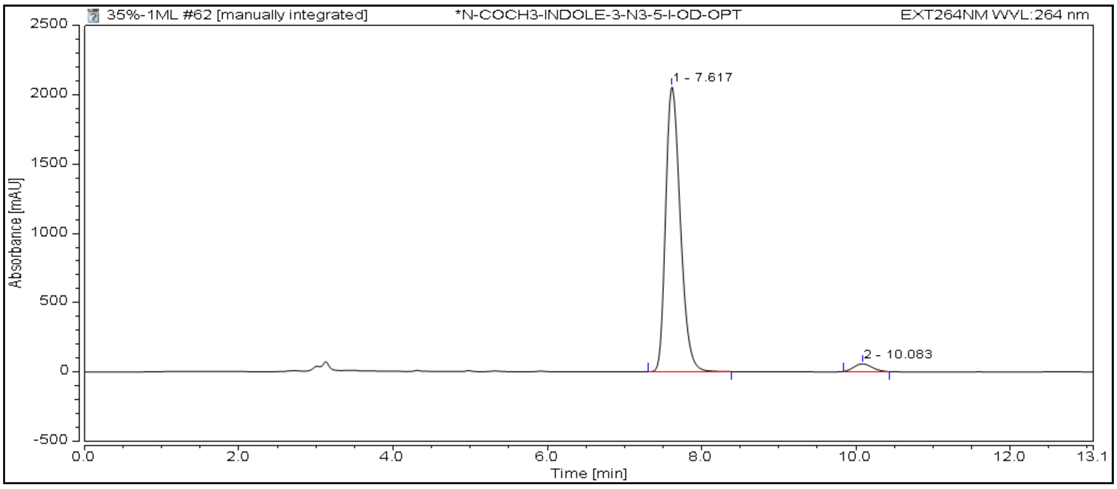

Integration Results					
No.	Peak Name	Retention Time<br>min	Area<br>mAU*min	Relative Area<br>%	Amount<br>n.a.
1		7.617	452.902	96.82	n.a.
2		10.083	14.893	3.18	n.a.
**Total:**			**467.796**	**100.00**	

Supplementary figure 76. HPLC chromatogram for **3d**

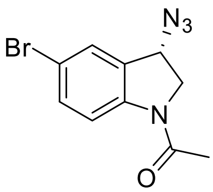

(*S*)-1-(3-Azido-5-bromoindolin-1-yl)ethan-1-one (3e)

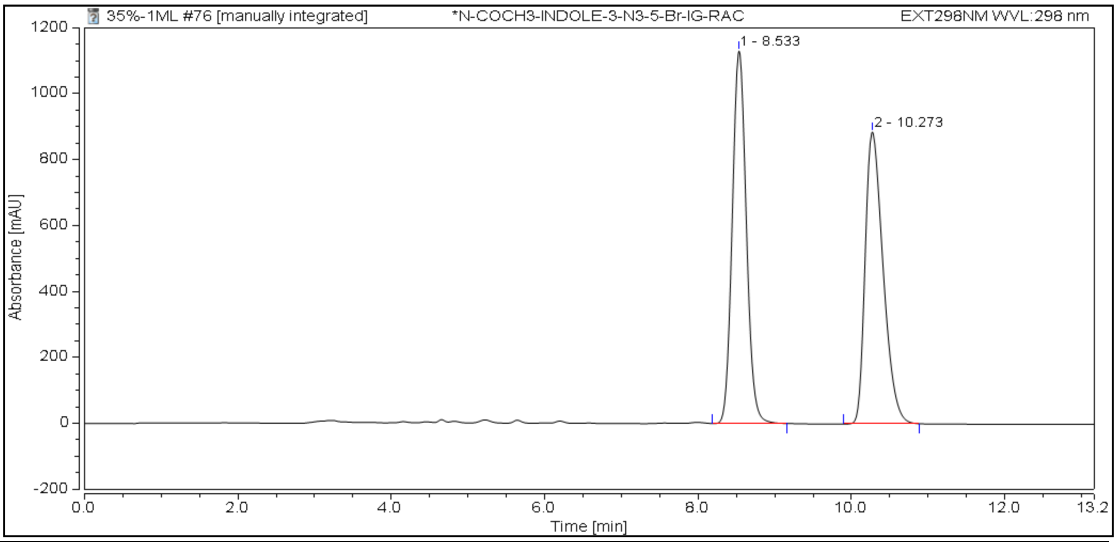

Integration Results					
No.	Peak Name	Retention Time<br>min	Area<br>mAU*min	Relative Area<br>%	Amount<br>n.a.
1		8.533	239.680	49.96	n.a.
2		10.273	240.038	50.04	n.a.
**Total:**			**479.718**	**100.00**	

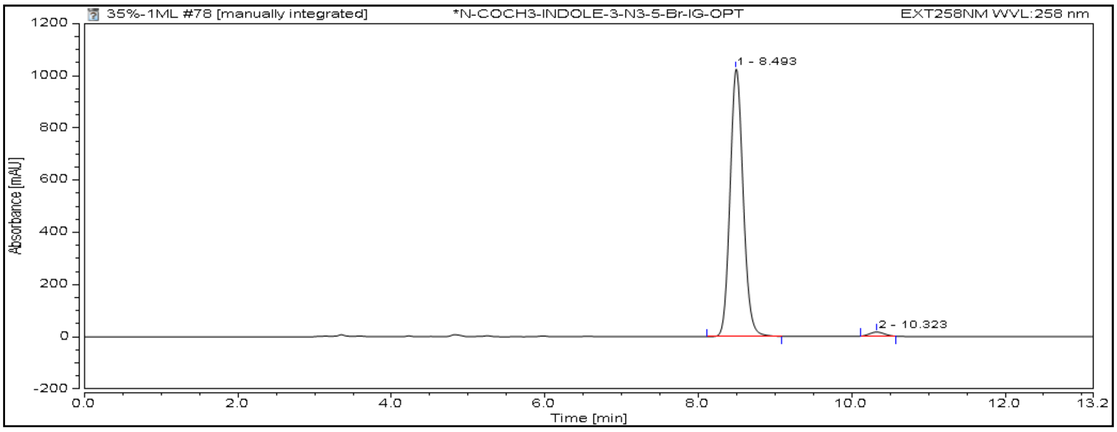

Integration Results					
No.	Peak Name	Retention Time<br>min	Area<br>mAU*min	Relative Area<br>%	Amount<br>n.a.
1		8.493	202.747	98.28	n.a.
2		10.323	3.542	1.72	n.a.
**Total:**			**206.289**	**100.00**	

Supplementary figure 77. HPLC chromatogram for **3e**

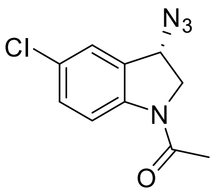

(*S*)-1-(3-Azido-5-chloroindolin-1-yl)ethan-1-one (3f)

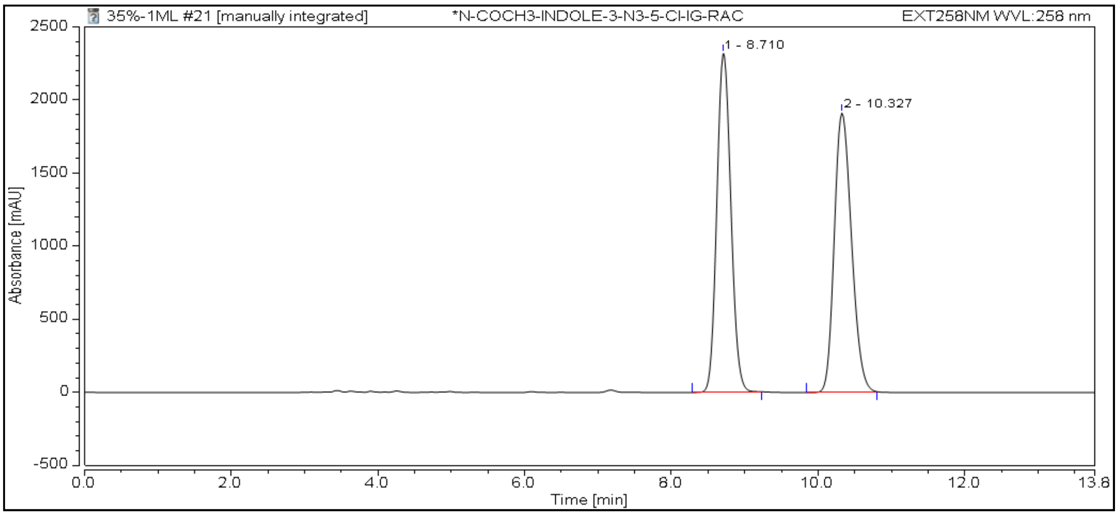

Integration Results					
No.	Peak Name	Retention Time<br>min	Area<br>mAU*min	Relative Area<br>%	Amount<br>n.a.
1		8.710	514.823	49.76	n.a.
2		10.327	519.784	50.24	n.a.
**Total:**			**1034.607**	**100.00**	

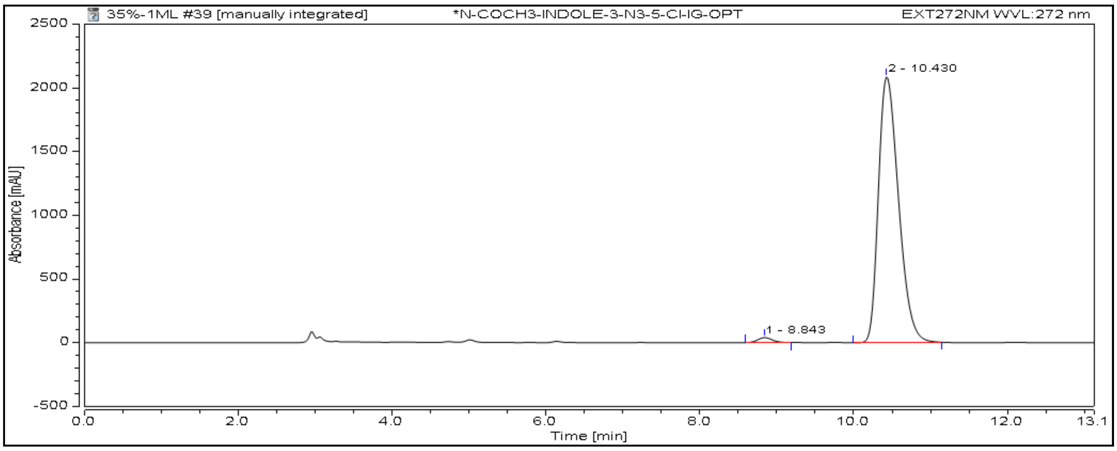

Integration Results					
No.	Peak Name	Retention Time<br>min	Area<br>mAU*min	Relative Area<br>%	Amount<br>n.a.
1		8.843	8.301	1.30	n.a.
2		10.430	630.246	98.70	n.a.
**Total:**			**638.546**	**100.00**	

Supplementary figure 78. HPLC chromatogram for **3f**

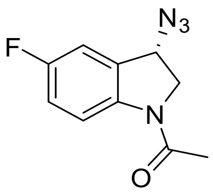

(*S*)-1-(3-Azido-5-fluoroindolin-1-yl)ethan-1-one (3g)

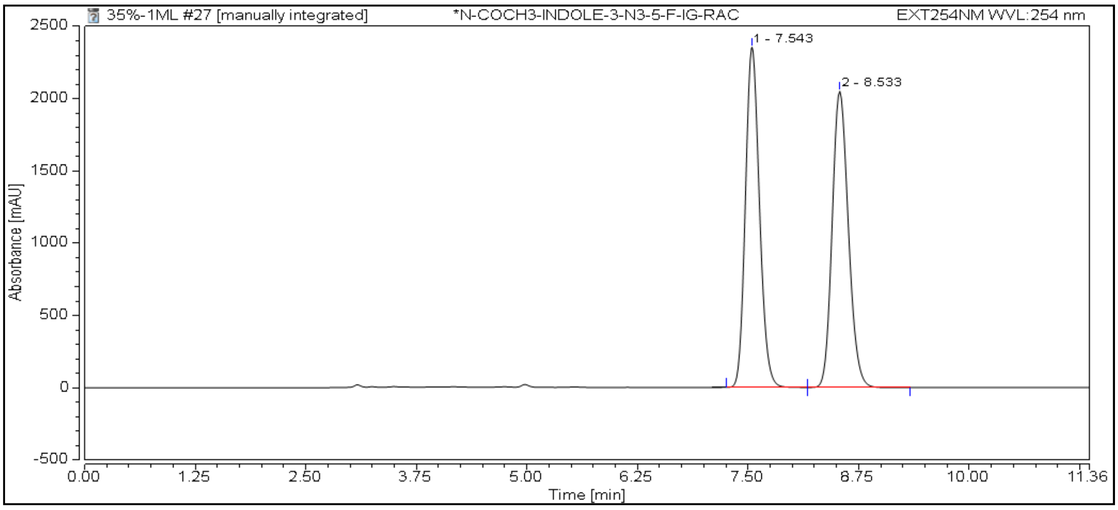

Integration Results					
No.	Peak Name	Retention Time<br>min	Area<br>mAU*min	Relative Area<br>%	Amount<br>n.a.
1		7.543	434.554	49.90	n.a.
2		8.533	436.250	50.10	n.a.
**Total:**			**870.804**	**100.00**	

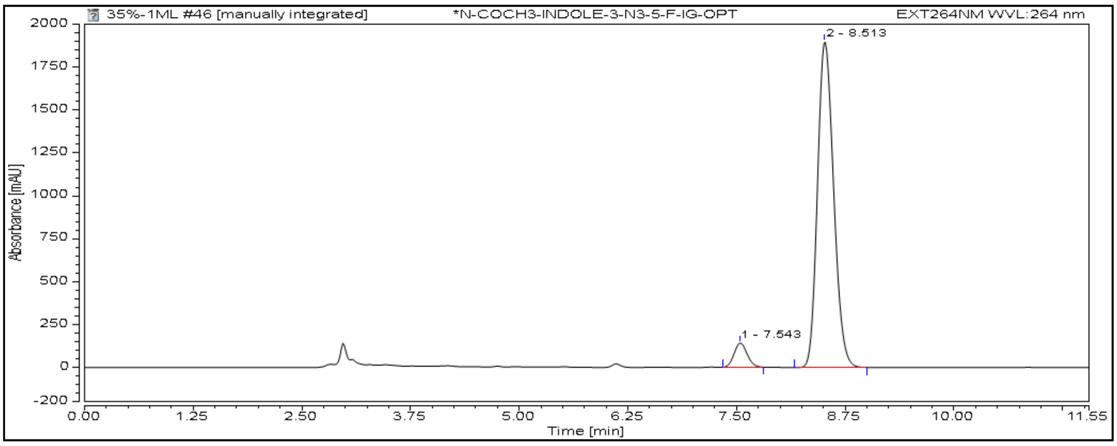

Integration Results					
No.	Peak Name	Retention Time<br>min	Area<br>mAU*min	Relative Area<br>%	Amount<br>n.a.
1		7.543	22.361	5.19	n.a.
2		8.513	408.643	94.81	n.a.
**Total:**			**431.004**	**100.00**	

Supplementary figure 79. HPLC chromatogram for **3g**

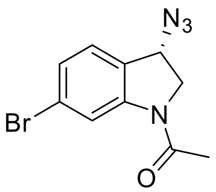

(*S*)-1-(3-Azido-6-bromoindolin-1-yl)ethan-1-one (3h)

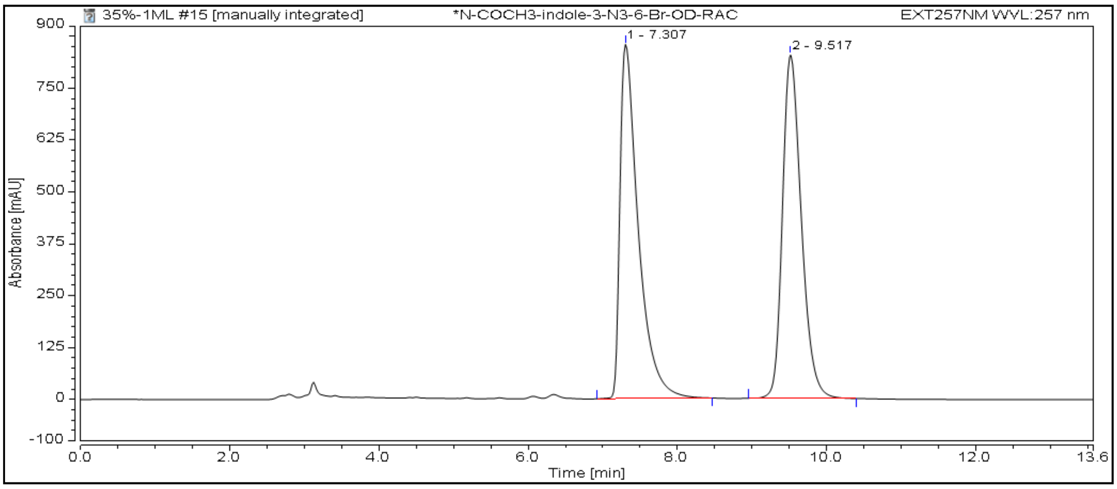

Integration Results					
No.	Peak Name	Retention Time<br>min	Area<br>mAU*min	Relative Area<br>%	Amount<br>n.a.
1		7.307	242.589	50.05	n.a.
2		9.517	242.130	49.95	n.a.
**Total:**			**484.719**	**100.00**	

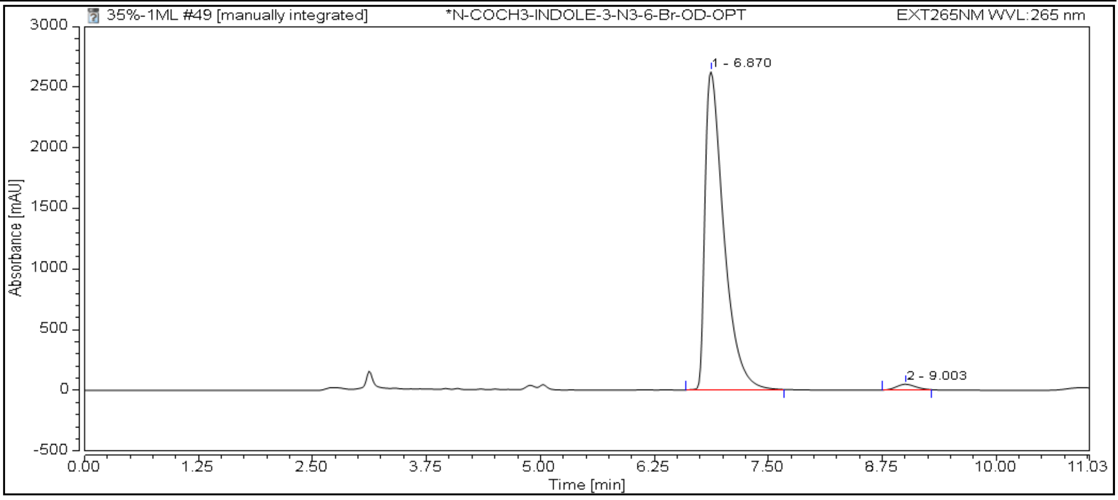

Integration Results					
No.	Peak Name	Retention Time<br>min	Area<br>mAU*min	Relative Area<br>%	Amount<br>n.a.
1		6.870	626.644	98.27	n.a.
2		9.003	11.057	1.73	n.a.
**Total:**			**637.701**	**100.00**	

Supplementary figure 80. HPLC chromatogram for **3h**

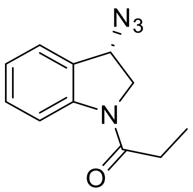

(*S*)-1-(3-Azidoindolin-1-yl)propan-1-one (3i)

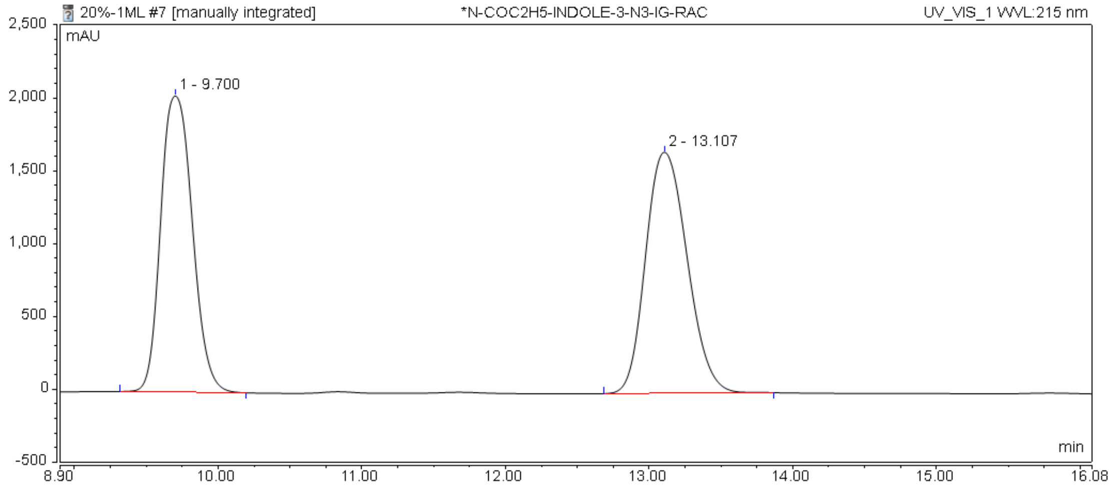

Integration Results					
No.	Peak Name	Retention Time<br>min	Area<br>mAU*min	Relative Area<br>%	Amount<br>n.a.
1		9.700	519.546	48.37	n.a.
2		13.107	554.628	51.63	n.a.
**Total:**			**1074.174**	**100.00**	

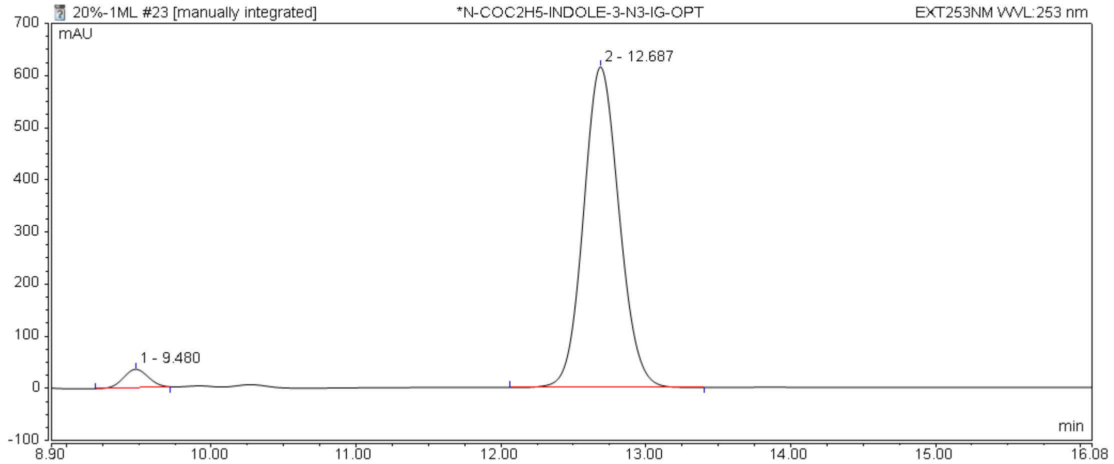

Integration Results					
No.	Peak Name	Retention Time<br>min	Area<br>mAU*min	Relative Area<br>%	Amount<br>n.a.
1		9.480	6.971	3.83	n.a.
2		12.687	175.155	96.17	n.a.
**Total:**			**182.126**	**100.00**	

Supplementary figure 81. HPLC chromatogram for **3i**

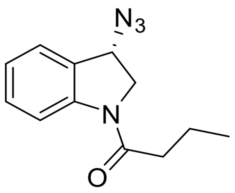

(*S*)-1-(3-Azidoindolin-1-yl)butan-1-one (3j)

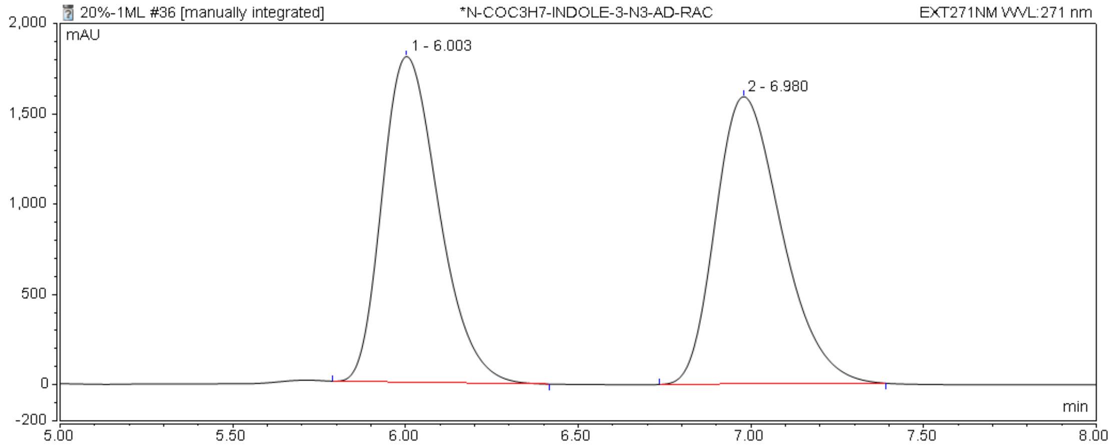

Integration Results					
No.	Peak Name	Retention Time min	Area mAU*min	Relative Area %	Amount n.a.
1		6.003	343.544	49.23	n.a.
2		6.980	354.257	50.77	n.a.
**Total:**			**697.801**	**100.00**	

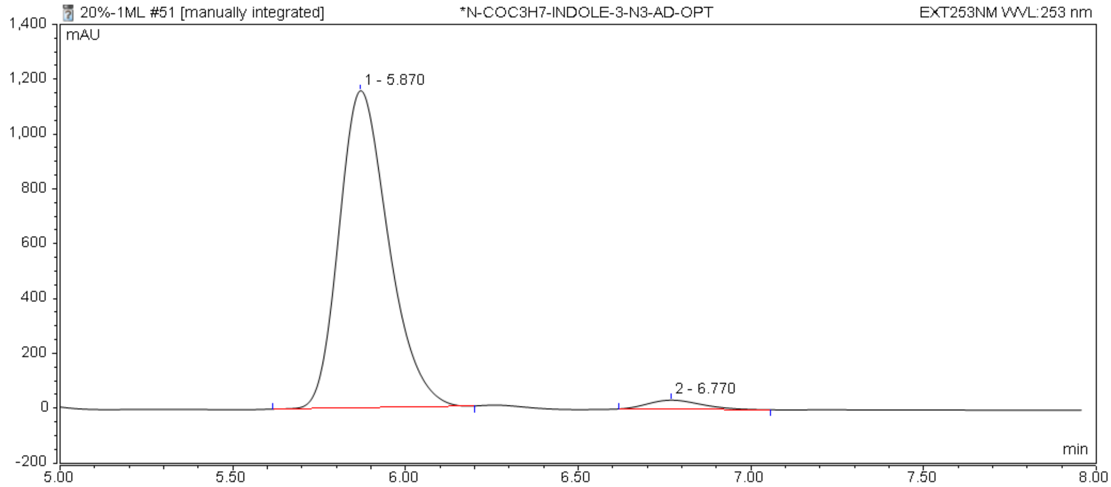

Integration Results					
No.	Peak Name	Retention Time min	Area mAU*min	Relative Area %	Amount n.a.
1		5.870	191.517	96.97	n.a.
2		6.770	5.980	3.03	n.a.
**Total:**			**197.496**	**100.00**	

Supplementary figure 82. HPLC chromatogram for **3j**

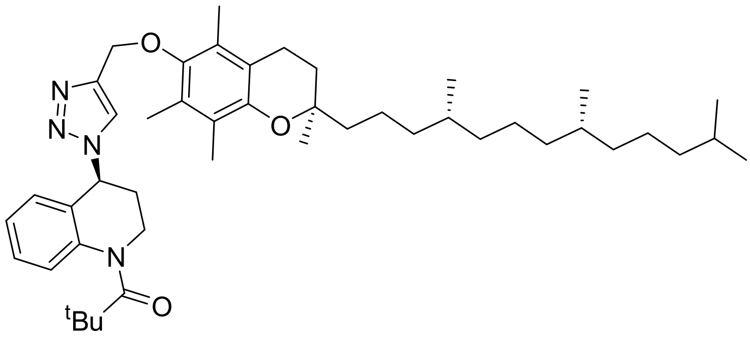

2,2-dimethyl-1-((*S*)-4-(4-((((R)-2,5,7,8-tetramethyl-2-((4*R*,8*R*)-4,8,12-trimethyltridecyl)chroman-6-yl)oxy)methyl)-1*H*-1,2,3-triazol-1-yl)-3,4-dihydroquinolin-1(2*H*)-yl)propan-1-one (6)

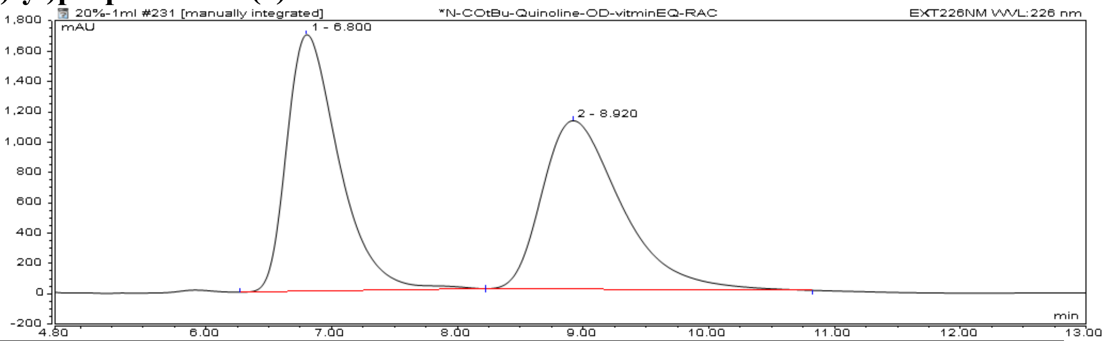

Integration Results					
No.	Peak Name	Retention Time<br>min	Area<br>mAU*min	Relative Area<br>%	Amount<br>n.a.
1		6.800	813.952	50.13	n.a.
2		8.920	809.866	49.87	n.a.
**Total:**			**1623.818**	**100.00**	

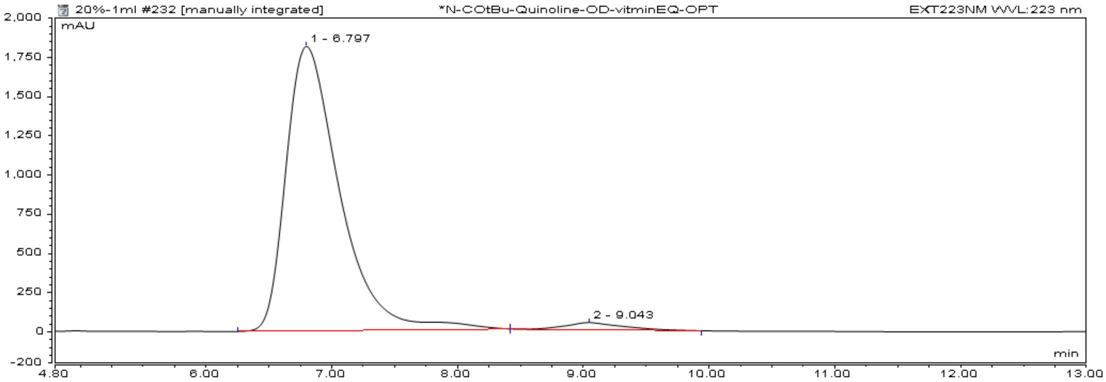

Integration Results					
No.	Peak Name	Retention Time<br>min	Area<br>mAU*min	Relative Area<br>%	Amount<br>n.a.
1		6.797	899.049	97.36	n.a.
2		9.043	24.373	2.64	n.a.
**Total:**			**923.422**	**100.00**	

Supplementary figure 83. HPLC chromatogram for **6**

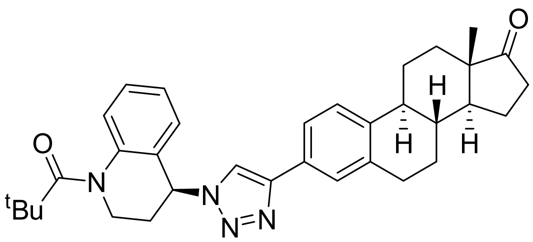

(8*R*,9*S*,13*S*,14*S*)-13-Methyl-3-(1-((*S*)-1-pivaloyl-1,2,3,4-tetrahydroquinolin-4-yl)-1*H*-1,2,3-triazol-4-yl)-6,7,8,9,11,12,13,14,15,16-decahydro-17*H*-cyclopenta[a]phenanthren-17-one (8)

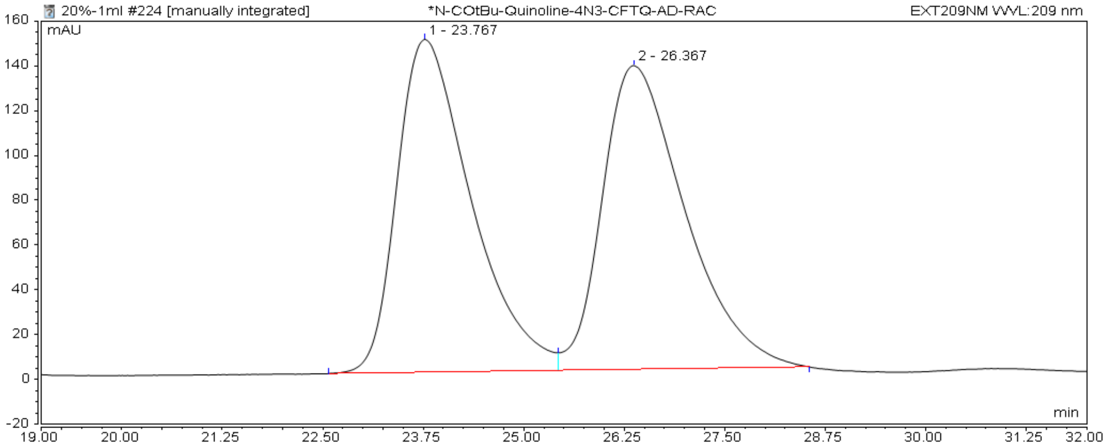

Integration Results					
No.	Peak Name	Retention Time min	Area mAU*min	Relative Area %	Amount n.a.
1		23.767	158.706	49.57	n.a.
2		26.367	161.458	50.43	n.a.
**Total:**			**320.164**	**100.00**	

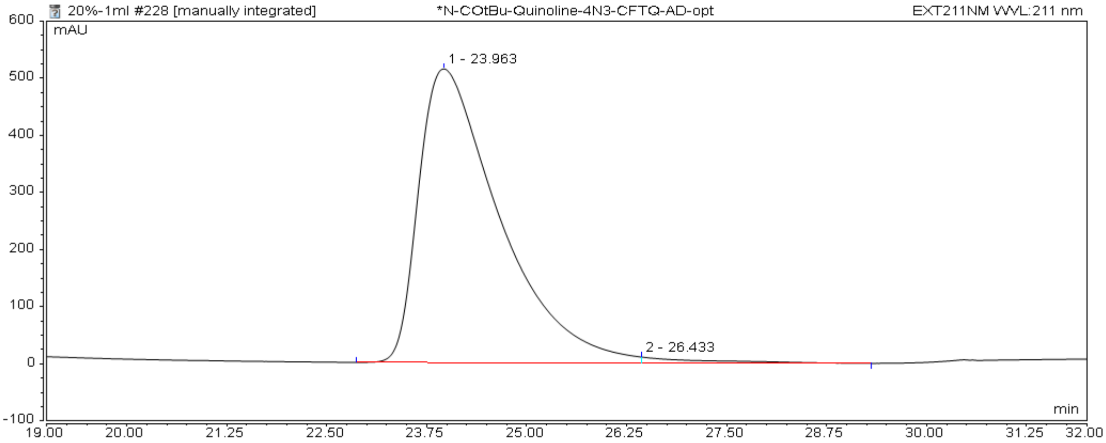

Integration Results					
No.	Peak Name	Retention Time min	Area mAU*min	Relative Area %	Amount n.a.
1		23.963	601.554	98.57	n.a.
2		26.433	8.706	1.43	n.a.
**Total:**			**610.261**	**100.00**	

Supplementary figure 84. HPLC chromatogram for **8**

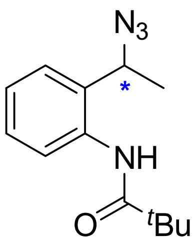

***N*-(2-(1-Azidoethyl)phenyl)pivalamide (A)**

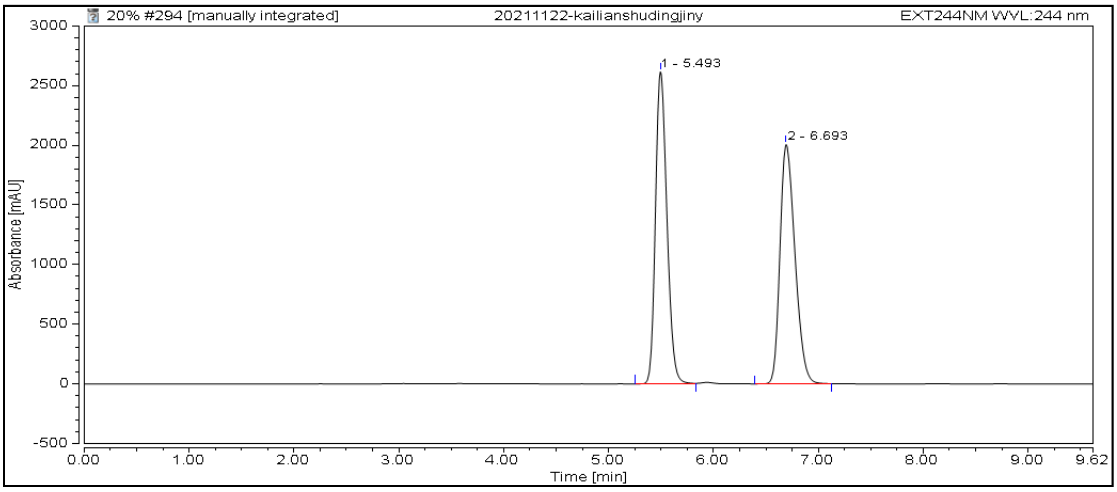

Integration Results

No.	Peak Name	Retention Time min	Area mAU*min	Relative Area %	Amount n.a.
1		5.493	329.218	49.82	n.a.
2		6.693	331.600	50.18	n.a.
**Total:**			**660.818**	**100.00**	

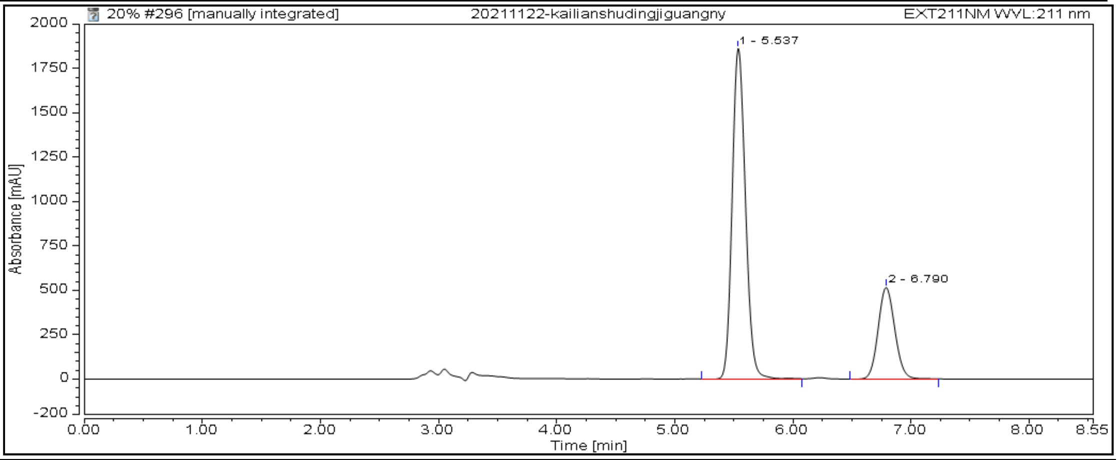

Integration Results

No.	Peak Name	Retention Time min	Area mAU*min	Relative Area %	Amount n.a.
1		5.537	242.260	74.19	n.a.
2		6.790	84.268	25.81	n.a.
**Total:**			**326.528**	**100.00**	

Supplementary figure 85. HPLC chromatogram for **A**

Supplementary References

[1] E. N. Jacobsen, W. Zhang, A. R. Muci, J. R. Ecker, L. Deng. *J. Am. Chem. Soc.* **1991**, 113, 7063.

[2] T. Hamada, R. Irie, J. Mihara, K. Hamachi, T. Katsuki. *Tetrahedron*, **1998**, 54, 10017.

[3] Y. Chen, X. Zhou, D. Zheng, B. Cui, W. Han. *Tetrahedron*, **2015**, 71(29), 4738.

[4] J. C. Rodríguez-Domínguez, A. Balbuzano-Deus, M. A. López-López, G. *J.* Kirsch. *Heterocyclic Chem.* **2007**, *44*, 273.

[5] S. Matsumoto, D. Samata, M. Akazome, K. Ogura. *Tetrahedron Lett.* **2009**, *50*, 111.

[6] I. Rintaro, N. Kazutaka, Y. Takanori, W. Takeshi. *Bioorg. Med. Chem.* **2014**, 22, 1394.

[7] J. R. Clark, A. Reyes, Z. P. Vang, L. Z. Li, K. T. Behlow, S. E. Sloane. *Org. Lett.* **2020**, 22, 9139.

[8] Gaussian 09 (Revision B.01), M. J. Frisch, G. W. Trucks, H. B. Schlegel, G. E. Scuseria, M. A. Robb, J. R. Cheeseman, G. Scalmani, V. Barone, B. Mennucci, G. A. Petersson, H. Nakatsuji, M. Caricato, X. Li, H. P. Hratchian, A. F. Izmaylov, J. Bloino, G. Zheng, J. L. Sonnenberg, M. Hada, M. Ehara, K. Toyota, R. Fukuda, J. Hasegawa, M. Ishida, T. Nakajima, Y. Honda, O. Kitao, H. Nakai, T. Vreven, J. A. Montgomery, Jr., J. E. Peralta, F. Ogliaro, M. Bearpark, J. J. Heyd, E. Brothers, K. N. Kudin, V. N. Staroverov, R. Kobayashi, J. Normand, K. Raghavachari, A. Rendell, J. C. Burant, S. S. Iyen-gar, J. Tomasi, M. Cossi, N. Rega, N. J. Millam, M. Klene, J. E. Knox, J. B. Cross, V. Bakken, C. Adamo, J. Jaramillo, R. Gomperts, R. E. Stratmann, O. Yazyev, A. J. Austin, R. Cammi, C. Pomelli, J. W. Ochterski, R. L. Martin, K. Morokuma, V. G. Zakrzewski, G. A. Voth, P. Salvador, J. J. Dannenberg, S. Dapprich, A. D. Daniels, O. Farkas, J. B. Foresman, J. V. Ortiz, J. Cioslowski, D. J. Fox, Gaussian, Inc., Wallingford, CT, **2010**.

[9] Y. Zhao, D. G. Truhlar, *Acc. Chem. Res.* **2008**, *41*, 157.

[10] P. J. Hay, W. R. Wadt, *J. Chem. Phys.* **1985**, *82*, 270.

[11] F. Weigend, R. Ahlrichs, *Phys. Chem. Chem. Phys.* **2005**, *7*, 3297.

[12] D. Andrae, U. Häussermann, M. Dolg, H. Stoll, H. Preuss, *Theor. Chim. Acta* **1990**, *77*, 123.

[13] C. Lefebvre, G. Rubez, H. Khartabil, J.-C. Boisson, J. Contreras-García, E. Hénon, *Phys. Chem. Chem. Phys.* **2017**, *19*, 17928.

[14] T. Lu, F. Chen, *J. Comput. Chem.* **2012**, *33*, 580.

[15] C. Y. Legault, *CYLview*, 1.0b, Université Sherbrooke, Sherbrooke, Quebec, Canada, **2009**, http://www.cylview.org.

[16] W. Humphrey, A. Dalke, K. Schulten, *J. Mol. Graphics* **1996**, *14*, 33.
