# Supplementary material for: Kinetic resolution of cyclic benzylic azides enabled by site- and enantioselective C(sp3)–H oxidation
Source: Nat Commun. 2022 Mar 25;13:1621. doi: 10.1038/s41467-022-29319-z (PMC8956603; doi:10.1038/s41467-022-29319-z)
Supplement: Supplementary file 3 — Description of Additional Supplementary Files [file 41467_2022_29319_MOESM3_ESM.pdf]

Description of Additional Supplementary files

File name: Supplementary Data 1

Description:1n derivative.cif Absolute configuration determination of 1n .

File name: Supplementary Data 2

Description:3a derivative.cif Absolute configuration determinatio n of 3a.

File name: Supplementary Data 3

Description: Cartesian coordinates.
