# Supplementary material for: Kinetic resolution of cyclic benzylic azides enabled by site- and enantioselective C(sp3)–H oxidation
Source: Nat Commun. 2022 Mar 25;13:1621. doi: 10.1038/s41467-022-29319-z (PMC8956603; doi:10.1038/s41467-022-29319-z)
Supplement: Supplementary file 6 — Supplementary Dataset 3 [file 41467_2022_29319_MOESM6_ESM.pdf]

Cartesian coordinates of the transition states

^{oss}Cat-1

C	5.47921200	-1.29951000	-0.27659300
C	6.71570300	-0.41079300	-0.37920300
C	6.61959100	0.79060400	0.55191400
C	5.35588400	1.60528200	0.28585700
C	4.13202700	0.71160600	0.40691000
C	4.22743900	-0.48367100	-0.55466800
N	2.83778000	1.35481600	0.15933200
N	2.95640100	-1.20056400	-0.46397700
C	2.61058000	2.61360700	0.27780000
C	2.82038200	-2.47645800	-0.46733800
C	1.34161600	3.25818800	0.10166700
C	1.57536400	-3.18040600	-0.33309700
C	1.30872500	4.66445800	0.12120400
C	0.12295000	5.34476500	-0.04363400
C	-1.06047200	4.62175500	-0.23547700
C	-1.08963600	3.23342700	-0.24484900
C	0.13170800	2.52133900	-0.04901700
C	0.32756200	-2.50183200	-0.22918300
C	-0.83534400	-3.24985500	0.10513000
C	-0.73935100	-4.63519500	0.18596800
C	0.47153900	-5.30806400	0.00557500
C	1.62017600	-4.57918300	-0.22742100
O	0.06575500	1.22378000	0.00085500
O	0.17209000	-1.22438800	-0.44443800
C	-2.33858000	2.48431800	-0.49367900
C	-2.09754000	-2.54681900	0.40711200
C	-2.37538400	1.36370500	-1.33950200
C	-3.54966900	0.67327600	-1.59610200
C	-4.71866600	1.09873400	-0.98463400
C	-4.74538700	2.19445700	-0.13961300
C	-3.55266100	2.86216500	0.08605200
C	-3.32963400	-2.99511700	-0.07024200
C	-4.53308900	-2.38138500	0.23946700
C	-4.49106400	-1.27576200	1.07143700
C	-3.29963200	-0.78220000	1.58067100
C	-2.11501400	-1.41553000	1.23871800
Mn	1.45473300	0.07491300	-0.38942900
Cl	1.57940000	-0.45042100	2.00732400
O	1.68674900	0.43970200	-1.88477700
H	5.56430000	-2.13212800	-0.98896500
H	5.40496100	-1.73715700	0.73355000
H	5.29871900	2.43660700	1.00186200

H	5.39106000	2.04578000	-0.72546400
H	4.07232000	0.30141600	1.42897000
H	4.27905100	-0.07726000	-1.58162000
H	3.44711700	3.27569100	0.53302300
H	3.71761300	-3.10503600	-0.52885800
H	2.24430800	5.20753500	0.26028700
H	0.10157700	6.43179000	-0.04021400
H	-1.99109400	5.16313700	-0.39207000
H	-1.63646000	-5.20389000	0.42188100
H	0.51098600	-6.39172400	0.08558000
H	2.58434600	-5.08090000	-0.31855200
H	-3.56629700	-0.20569700	-2.23584500
H	-5.66199800	2.51002400	0.34986900
H	-5.47049100	-2.75264400	-0.16427800
H	-3.31076900	0.09690300	2.22024200
H	7.61625100	-0.99805200	-0.15669500
H	6.82087300	-0.05830400	-1.41865500
H	7.50515700	1.43090900	0.44614000
H	6.60792700	0.44282300	1.59776400
F	-5.84992100	0.42373400	-1.19578800
F	-5.63604200	-0.66111600	1.37787300
H	-1.17052600	-1.02947500	1.62192200
F	-3.37247600	-4.06578900	-0.87631000
F	-3.58810800	3.91210100	0.91766100
H	-1.45014700	1.02165100	-1.79754900

^{oss}Cat-2

C	5.32467500	-1.60051700	-0.30833900
C	6.61984500	-0.79744700	-0.39471300
C	6.61630800	0.37248500	0.57969900
C	5.41245200	1.28464500	0.35552100
C	4.12759500	0.47734200	0.45438500
C	4.13221300	-0.68908600	-0.54593500
N	2.88304300	1.22093200	0.23434800
N	2.81458100	-1.31706200	-0.46418400
C	2.74861100	2.48810900	0.39456400
C	2.58712800	-2.57925500	-0.50089800
C	1.52554400	3.22247900	0.24486900
C	1.29570100	-3.19451100	-0.37614500
C	1.58006300	4.62749300	0.31399500
C	0.44068100	5.39046900	0.18720000
C	-0.78712600	4.74966700	-0.02609800
C	-0.89778500	3.36957900	-0.08837800
C	0.27023500	2.57025600	0.08222900

C	0.10015400	-2.43192700	-0.24499500
C	-1.11083000	-3.10563700	0.07702900
C	-1.11141100	-4.49588300	0.12333300
C	0.04778300	-5.24790600	-0.08341100
C	1.24301400	-4.59569300	-0.30666300
O	0.10474500	1.28290800	0.10715800
O	0.03251900	-1.14123800	-0.42109100
C	-2.20297300	2.71865300	-0.31154200
C	-2.31838700	-2.32596100	0.41373400
C	-2.38140900	1.73328100	-1.28998100
C	-3.60003200	1.11813200	-1.51752300
C	-4.68302300	1.52107200	-0.75435700
C	-4.57568100	2.50324600	0.21724700
C	-3.33221800	3.08712400	0.42667900
C	-3.58507900	-2.68739800	-0.04647000
C	-4.74496400	-2.02328800	0.32055500
C	-4.62119900	-0.95546600	1.19241200
C	-3.39039200	-0.53855800	1.67540500
C	-2.25077600	-1.22024500	1.27641000
Mn	1.40624300	0.05848300	-0.34416600
Cl	1.51222700	-0.54470800	2.04240300
O	1.66390900	0.44630600	-1.82413700
H	5.34346700	-2.41277700	-1.04845700
H	5.22784300	-2.06480900	0.68794400
H	5.42214400	2.09085100	1.10166200
H	5.47276900	1.75779700	-0.63975300
H	4.04052100	0.03851100	1.46259300
H	4.20565700	-0.25363300	-1.55946300
H	3.63074300	3.08112800	0.66664500
H	3.43594700	-3.26937000	-0.58764400
H	2.54972100	5.10468500	0.46251700
H	0.49206700	6.47566100	0.23002000
H	-1.68820700	5.34679600	-0.16923200
H	-2.04433200	-5.00692700	0.35261900
H	0.01028800	-6.33326600	-0.03001900
H	2.16917100	-5.16109100	-0.41667900
H	-3.70145300	0.35101800	-2.28009400
H	-5.44916000	2.77461700	0.80432400
H	-5.71185200	-2.32967200	-0.06760500
H	-3.33756100	0.31625300	2.34499300
H	7.47908600	-1.45374100	-0.20425100
H	6.73921500	-0.41583000	-1.42229500
H	7.54485700	0.95120400	0.48829800
H	6.58732000	-0.01252200	1.61208200

H	-3.21767200	3.84150200	1.20476500
F	-1.36445200	1.39772800	-2.08005000
F	-5.86528400	0.93933800	-0.96551200
F	-5.72448100	-0.30296100	1.56759600
H	-1.27815800	-0.90348900	1.65103100
F	-3.70705100	-3.72492100	-0.88716800

^{oss}Cat-3

C	5.49160100	-1.10997800	-0.48644800
C	6.67058400	-0.16332200	-0.69723600
C	6.58563600	1.04645400	0.22387200
C	5.26449700	1.79255400	0.05142000
C	4.09855800	0.84149600	0.27584700
C	4.18544900	-0.35833400	-0.68087800
N	2.76067800	1.42464900	0.11947900
N	2.95860200	-1.13239000	-0.50825000
C	2.47245900	2.65646800	0.33812900
C	2.87981100	-2.41097700	-0.45578700
C	1.16273200	3.23778200	0.26186800
C	1.66783400	-3.16172000	-0.26906800
C	1.05504600	4.63507800	0.38078900
C	-0.16721000	5.26156300	0.27529400
C	-1.31056500	4.49443000	0.02386300
C	-1.26456900	3.11090300	-0.08687000
C	-0.01058000	2.45092000	0.07987500
C	0.38882300	-2.53944000	-0.18866200
C	-0.74704500	-3.34855800	0.09351800
C	-0.58787100	-4.72382900	0.20016500
C	0.66109900	-5.34025100	0.07516800
C	1.77504500	-4.55663100	-0.14290000
O	-0.01912500	1.15083500	0.06508900
O	0.17276800	-1.27175900	-0.39519300
C	-2.45835100	2.31692400	-0.44299400
C	-2.08408200	-2.73846900	0.24472600
C	-2.39382800	1.27687300	-1.38505100
C	-3.51856100	0.56875400	-1.77764800
C	-4.74202400	0.89881400	-1.21573300
C	-4.86580000	1.89914300	-0.26715200
C	-3.71795200	2.58595000	0.09664100
C	-3.18155900	-3.20294200	-0.48654600
C	-4.45958600	-2.68173600	-0.31474900
C	-4.63382300	-1.67298500	0.61911000
C	-3.58247400	-1.17258400	1.36868800
C	-2.32503600	-1.71866300	1.17309600

Mn	1.40788300	0.07997100	-0.36910300
Cl	1.69508000	-0.42016100	1.99120600
O	1.58391000	0.44936200	-1.87135700
H	5.56118100	-1.95051000	-1.19132500
H	5.51796900	-1.53252900	0.53249300
H	5.22331600	2.63021500	0.76093900
H	5.19465800	2.22126100	-0.96307800
H	4.13510400	0.44067400	1.30285000
H	4.15052400	0.03992600	-1.71137000
H	3.28394900	3.34774800	0.59756500
H	3.80153400	-3.00254900	-0.52509700
H	1.96226600	5.21707000	0.54740000
H	-0.24542000	6.34276000	0.36006100
H	-2.26885800	4.99448000	-0.10165400
H	-1.46889900	-5.32841000	0.41727300
H	0.75315400	-6.41897900	0.17598900
H	2.76345000	-5.01257000	-0.21327700
H	-3.45984100	-0.23928400	-2.50234900
H	-5.82468800	2.13580300	0.18472000
H	-5.31083100	-3.03636300	-0.88991800
H	7.61479000	-0.70080500	-0.53889900
H	6.67646100	0.17591700	-1.74637700
H	7.42586100	1.72903500	0.04067400
H	6.67396500	0.71349000	1.27089000
F	-5.83225900	0.22874900	-1.59238500
F	-3.84552800	3.55057500	1.01743800
H	-1.43145800	1.02556000	-1.82490400
H	-3.01820900	-3.99038200	-1.22213500
F	-1.33433600	-1.28538600	1.93691300
F	-5.85149300	-1.15925000	0.80932700
H	-3.73578700	-0.38575100	2.10195500

^{oss}Cat-4

C	5.35122100	-1.39488100	-0.45150000
C	6.59590500	-0.52987100	-0.63292100
C	6.58768100	0.66528200	0.31004900
C	5.32374000	1.50608400	0.14331600
C	4.09346200	0.63387500	0.34312100
C	4.10278200	-0.54890500	-0.63844100
N	2.79910600	1.30998700	0.19700800
N	2.82416000	-1.24219300	-0.49361400
C	2.59382900	2.55551900	0.43179400
C	2.65314300	-2.51285500	-0.50350600
C	1.32238900	3.21894200	0.36383800

C	1.38886200	-3.18313800	-0.35869100
C	1.29366100	4.61917200	0.50361600
C	0.10901300	5.31857600	0.42588800
C	-1.07950700	4.61954400	0.17478400
C	-1.10743200	3.24037000	0.04148200
C	0.10418900	2.50416600	0.18729100
C	0.15837700	-2.47591900	-0.23589400
C	-1.03183900	-3.21626900	0.01065400
C	-0.97488100	-4.60275300	0.02868000
C	0.22542900	-5.30026500	-0.14409900
C	1.39359400	-4.58763400	-0.31652900
O	0.00604900	1.21045100	0.16625400
O	0.02977600	-1.18750200	-0.36886700
C	-2.36253100	2.52678100	-0.26549600
C	-2.31576100	-2.52042700	0.23567400
C	-2.44083100	1.59461100	-1.30760000
C	-3.61800100	0.95496000	-1.65307100
C	-4.76317200	1.27256200	-0.94279400
C	-4.75149000	2.17920900	0.10482100
C	-3.54727500	2.79468000	0.42839500
C	-3.46529900	-2.84999500	-0.48858000
C	-4.69874200	-2.26257300	-0.22587700
C	-4.77359300	-1.32714100	0.79335600
C	-3.66445000	-0.94906700	1.52997900
C	-2.45502300	-1.55919200	1.24349500
Mn	1.35909200	0.06723400	-0.31929600
Cl	1.63800700	-0.52100400	2.02943200
O	1.56568200	0.45629600	-1.80668500
H	5.36842700	-2.22319700	-1.17375300
H	5.33633400	-1.83923500	0.55833000
H	5.33824500	2.33066500	0.86879500
H	5.29241000	1.95704800	-0.86340700
H	4.09843800	0.21104300	1.36154800
H	4.10448400	-0.12646400	-1.66001200
H	3.44760800	3.18989300	0.70133400
H	3.52919800	-3.16678200	-0.59933700
H	2.23510900	5.14591000	0.66540000
H	0.09643100	6.40118800	0.52583600
H	-2.01333100	5.16973200	0.05397900
H	-1.89729600	-5.15313400	0.21601000
H	0.23825400	-6.38697900	-0.11150800
H	2.34646900	-5.10895800	-0.41755900
H	-3.64080400	0.24261700	-2.47256100
H	-5.66841100	2.37656800	0.65413700

H	-5.59116200	-2.50638900	-0.79642900
H	-3.73746700	-0.20895500	2.32186100
H	7.49818300	-1.13556800	-0.47651300
H	6.63616900	-0.17258100	-1.67531000
H	7.47550700	1.28977600	0.14471300
H	6.64473900	0.30818800	1.35128800
H	-3.38026500	-3.58609400	-1.28782100
H	-3.51184400	3.49861200	1.25937000
F	-1.41337900	-1.25387900	2.00091500
F	-1.36700500	1.34563400	-2.05402900
F	-5.90717700	0.67344500	-1.28081400
F	-5.94893200	-0.75852200	1.07535900

***css*Cat-1**

C	5.47794300	-1.35874300	-0.18635200
C	6.72705800	-0.49269700	-0.33256200
C	6.63412900	0.77168000	0.51159800
C	5.38024800	1.57726200	0.18106700
C	4.14758800	0.70291200	0.35448700
C	4.24124800	-0.54603600	-0.53225300
N	2.85733500	1.34614700	0.07171500
N	2.95834200	-1.23893100	-0.42575400
C	2.62078200	2.59360800	0.27133500
C	2.79825300	-2.51104200	-0.38323900
C	1.34911500	3.24070700	0.14214900
C	1.53722700	-3.18665400	-0.25744200
C	1.30176900	4.63974000	0.28659800
C	0.11025500	5.32135600	0.17810500
C	-1.06559900	4.60794300	-0.08570900
C	-1.07930900	3.22675300	-0.22276200
C	0.14818400	2.51057900	-0.08365600
C	0.29914300	-2.48324700	-0.20479300
C	-0.88136600	-3.20126900	0.13859600
C	-0.81073900	-4.58455400	0.26471800
C	0.39101600	-5.28415800	0.12806400
C	1.55608800	-4.58201400	-0.10318100
O	0.08663300	1.21466900	-0.16598100
O	0.16456100	-1.21344300	-0.46944900
C	-2.31624900	2.48406100	-0.53901600
C	-2.13400700	-2.47196000	0.42053300
C	-2.33309400	1.44716600	-1.48549300
C	-3.48778600	0.74094500	-1.78249800
C	-4.65688200	1.06801900	-1.11401000
C	-4.70470000	2.08416500	-0.17517800

C	-3.53045200	2.77098900	0.08871800
C	-3.37611600	-2.92032400	-0.03061900
C	-4.57298600	-2.30172800	0.29566400
C	-4.51435600	-1.18764400	1.11534500
C	-3.31122500	-0.68574300	1.58732100
C	-2.13400500	-1.32553500	1.23114400
Mn	1.48284300	0.06142500	-0.47440600
Cl	1.54725000	-0.34656600	1.96214600
O	1.77169100	0.30182500	-1.97354400
H	5.55949300	-2.23650300	-0.84262300
H	5.38632500	-1.73004300	0.84849300
H	5.33100000	2.45682800	0.83710900
H	5.42321500	1.94771300	-0.85740200
H	4.08421100	0.35378000	1.39920400
H	4.31711700	-0.20227500	-1.58057300
H	3.45211400	3.24161500	0.57533700
H	3.68518400	-3.15700900	-0.39588300
H	2.23101600	5.17696900	0.48019600
H	0.07851500	6.40319500	0.28193900
H	-2.00297200	5.14991000	-0.19411000
H	-1.72118100	-5.12995900	0.50462000
H	0.40958800	-6.36516600	0.24374800
H	2.51341700	-5.10212800	-0.15525700
H	-3.49140000	-0.06733200	-2.50894800
H	-5.62057400	2.31708300	0.36021100
H	-5.51829200	-2.68058500	-0.08170200
H	-3.30932200	0.19569200	2.22372500
H	7.61765200	-1.07352600	-0.05875700
H	6.84907600	-0.21517000	-1.39271100
H	7.52670600	1.39465300	0.36803700
H	6.61196100	0.49679000	1.57877900
F	-5.76850400	0.37639400	-1.37142700
F	-5.65427700	-0.58392900	1.46323400
H	-1.18244700	-0.93842300	1.59544400
F	-3.43736700	-4.00302900	-0.81951100
F	-3.57949400	3.74095000	1.01185600
H	-1.40782500	1.18427400	-1.99259700

^{css}Cat-2

C	5.32826000	-1.61569800	-0.27812100
C	6.62545700	-0.81843200	-0.38936400
C	6.62588200	0.38083800	0.54832300
C	5.42275900	1.28823200	0.30044900
C	4.13727900	0.48472900	0.42646700

C	4.13901200	-0.70686200	-0.54067600
N	2.89062200	1.22354000	0.19294200
N	2.81882100	-1.32628100	-0.44619300
C	2.74964800	2.48467100	0.39306000
C	2.58271100	-2.58684300	-0.46323700
C	1.52322600	3.21555900	0.26805000
C	1.28631800	-3.19079900	-0.34045600
C	1.56918100	4.61829900	0.38141200
C	0.42546600	5.37781100	0.27748800
C	-0.79790500	4.73597200	0.04220700
C	-0.89978000	3.35782800	-0.06275800
C	0.27316300	2.56054800	0.08414600
C	0.09246200	-2.42105300	-0.22607300
C	-1.12120100	-3.09094100	0.09833300
C	-1.12594400	-4.48032200	0.16124000
C	0.03186000	-5.23853600	-0.02971500
C	1.22916400	-4.59121800	-0.25329300
O	0.11088100	1.27237600	0.05677000
O	0.02442300	-1.13237800	-0.41449400
C	-2.20012300	2.70669800	-0.31071600
C	-2.33009800	-2.30861100	0.42381800
C	-2.36892500	1.74349700	-1.31274300
C	-3.58335200	1.12751100	-1.55981400
C	-4.67123100	1.50534700	-0.79089300
C	-4.57417100	2.46749800	0.20164800
C	-3.33507900	3.05323200	0.42972000
C	-3.59522100	-2.67522900	-0.03669000
C	-4.75713200	-2.01129200	0.32399600
C	-4.63720500	-0.93900200	1.19048800
C	-3.40827900	-0.51768900	1.67448000
C	-2.26621000	-1.19806800	1.28032000
Mn	1.41857200	0.05599700	-0.37828100
Cl	1.52007900	-0.50504800	2.02863100
O	1.67994500	0.36796000	-1.86537100
H	5.34380300	-2.44881500	-0.99475900
H	5.23070800	-2.05168300	0.73077600
H	5.43740300	2.11597600	1.02239900
H	5.48031500	1.73281900	-0.70800900
H	4.05507800	0.07335500	1.44668300
H	4.21648600	-0.29921500	-1.56566700
H	3.62841500	3.07350700	0.68429400
H	3.42809200	-3.28341000	-0.53001900
H	2.53610000	5.09609500	0.54490000
H	0.46985900	6.46143600	0.35420200

H	-1.70273800	5.33132200	-0.08358700
H	-2.06138400	-4.98582700	0.39251300
H	-0.00867300	-6.32304200	0.03699700
H	2.15487100	-5.15985000	-0.34995700
H	-3.67761400	0.38119700	-2.34345000
H	-5.45122600	2.71518000	0.79403500
H	-5.72245100	-2.32049900	-0.06585300
H	-3.35890000	0.34013900	2.34026100
H	7.48360300	-1.47024900	-0.17946900
H	6.74476400	-0.46907100	-1.42836000
H	7.55517300	0.95474500	0.43630600
H	6.59956800	0.02794200	1.59221000
H	-3.22791000	3.78997300	1.22552300
F	-1.34721300	1.43053100	-2.10667600
F	-5.84861600	0.91917600	-1.01653900
F	-5.74267100	-0.28459800	1.55720300
H	-1.29426500	-0.87599400	1.65247500
F	-3.71402800	-3.71740500	-0.87212300

***css*Cat-3**

C	5.51235300	-1.11784200	-0.39866300
C	6.69641000	-0.18188000	-0.63158100
C	6.59130400	1.07641800	0.22059400
C	5.27173600	1.80730900	-0.01555500
C	4.10645900	0.86359100	0.24319000
C	4.21112600	-0.37657900	-0.65595100
N	2.76618300	1.43471600	0.05353700
N	2.97909700	-1.14093200	-0.47875300
C	2.46607200	2.65707500	0.30910700
C	2.89229600	-2.41740800	-0.39972000
C	1.15198900	3.22748300	0.25553900
C	1.67313800	-3.15887900	-0.22528200
C	1.03275500	4.61834600	0.42908300
C	-0.19506500	5.23683000	0.35292100
C	-1.33292800	4.46842000	0.08145800
C	-1.27742100	3.08994500	-0.07864100
C	-0.01406600	2.43638100	0.04717200
C	0.39453500	-2.53059100	-0.17625600
C	-0.74687400	-3.33284500	0.10710200
C	-0.59425700	-4.70669600	0.23583000
C	0.65407300	-5.32946200	0.13690300
C	1.77339400	-4.55219100	-0.07458800
O	-0.01096200	1.13896200	-0.03799200
O	0.18065900	-1.26755900	-0.40745000

C	-2.47259200	2.30133100	-0.44351000
C	-2.08390300	-2.71899100	0.24383600
C	-2.41690600	1.28119100	-1.40812700
C	-3.54379700	0.57748900	-1.80231000
C	-4.76199400	0.89268200	-1.22138300
C	-4.87792000	1.87369600	-0.25194100
C	-3.72796200	2.55503300	0.11475700
C	-3.18020900	-3.19504000	-0.48180100
C	-4.46114500	-2.68022700	-0.31172900
C	-4.64028600	-1.66751700	0.61671400
C	-3.59013800	-1.15477800	1.35956000
C	-2.32888800	-1.69167800	1.16279800
Mn	1.43138800	0.07666700	-0.42679200
Cl	1.68366800	-0.35042000	1.96387100
O	1.63939000	0.35415800	-1.93468400
H	5.59590700	-1.99114700	-1.06083200
H	5.51607400	-1.49151800	0.63942700
H	5.21922100	2.68400500	0.64414600
H	5.21698100	2.17826000	-1.05326200
H	4.13684600	0.51016400	1.28786300
H	4.20069500	-0.02496700	-1.70419400
H	3.27091000	3.34630900	0.59374200
H	3.81295000	-3.01380000	-0.43349900
H	1.93559700	5.20123000	0.61505900
H	-0.28303400	6.31335200	0.47802700
H	-2.29558500	4.96513700	-0.01862000
H	-1.48005700	-5.30481000	0.45149900
H	0.74101800	-6.40689900	0.25465100
H	2.76146200	-5.01193500	-0.12123800
H	-3.48979900	-0.21752600	-2.54164300
H	-5.83201900	2.09696200	0.21668000
H	-5.31121800	-3.04418000	-0.88278300
H	7.63651400	-0.70968700	-0.42379600
H	6.72588300	0.10074400	-1.69702600
H	7.43360300	1.75009900	0.01524200
H	6.66130200	0.80124300	1.28559700
F	-5.85387000	0.22495800	-1.59710900
F	-3.85022800	3.49636800	1.06025200
H	-1.45886900	1.03871300	-1.86132700
H	-3.01389800	-3.98799200	-1.21074200
F	-1.33883700	-1.24200000	1.91801300
F	-5.86129900	-1.16155900	0.80791000
H	-3.74680300	-0.36587300	2.08978600

^{css}Cat-4

C	5.36220300	-1.39672700	-0.41658600
C	6.60677100	-0.53417400	-0.61153500
C	6.59233200	0.68288400	0.30281000
C	5.32738500	1.51742400	0.11180100
C	4.09853300	0.64644300	0.32723200
C	4.11466000	-0.55528900	-0.62838900
N	2.80147800	1.31612200	0.16434300
N	2.83489700	-1.24419300	-0.47921400
C	2.58957500	2.55532300	0.42756400
C	2.65974700	-2.51403800	-0.47587400
C	1.31587900	3.21296900	0.37287900
C	1.39211600	-3.17855400	-0.33655200
C	1.27978800	4.60981500	0.54397200
C	0.09192200	5.30437000	0.47856300
C	-1.09240000	4.60419700	0.21073000
C	-1.11354700	3.22797600	0.04899100
C	0.10247800	2.49526400	0.17809700
C	0.16105400	-2.46850400	-0.22656500
C	-1.02972800	-3.20882900	0.02114400
C	-0.97378500	-4.59498400	0.04863300
C	0.22691400	-5.29452800	-0.11315500
C	1.39573300	-4.58293700	-0.28324400
O	0.00904900	1.20232500	0.11798600
O	0.02931400	-1.18177200	-0.36863200
C	-2.36725400	2.51627000	-0.26751200
C	-2.31498300	-2.51354400	0.23956500
C	-2.44908300	1.59652500	-1.32050500
C	-3.62717400	0.95882100	-1.66686300
C	-4.76969300	1.26649100	-0.94808900
C	-4.75523900	2.16387300	0.10741700
C	-3.55022500	2.77624000	0.43283700
C	-3.46250600	-2.84853400	-0.48518600
C	-4.69842700	-2.26526500	-0.22476100
C	-4.77778500	-1.32899300	0.79321300
C	-3.67072100	-0.94591700	1.53053700
C	-2.45839900	-1.55080800	1.24529100
Mn	1.37102500	0.06749100	-0.35040800
Cl	1.63546800	-0.48951100	2.01618900
O	1.58400800	0.39569900	-1.84295100
H	5.38346800	-2.24074300	-1.12033000
H	5.34241400	-1.81905000	0.60254100
H	5.33847600	2.35895500	0.81750600
H	5.29845200	1.94512800	-0.90502500

H	4.10190800	0.24513400	1.35446200
H	4.12384700	-0.15421700	-1.65877200
H	3.43960500	3.18699700	0.71485100
H	3.53518800	-3.17123300	-0.55360800
H	2.21831200	5.13735900	0.71935900
H	0.07299900	6.38451700	0.60158600
H	-2.02901900	5.15190700	0.10097900
H	-1.89745900	-5.14336700	0.23566900
H	0.23929900	-6.38099100	-0.07296600
H	2.34920200	-5.10507900	-0.37427300
H	-3.65266400	0.25545000	-2.49393500
H	-5.67008500	2.35526100	0.66229500
H	-5.58938500	-2.51346100	-0.79570500
H	-3.74759700	-0.20616900	2.32238600
H	7.50918700	-1.13451700	-0.43630200
H	6.65179000	-0.20140600	-1.66183900
H	7.47973900	1.30490800	0.12615800
H	6.64579500	0.35069400	1.35245900
H	-3.37397100	-3.58622500	-1.28257800
H	-3.51211800	3.47056400	1.27171400
F	-1.41819200	-1.23941100	2.00231100
F	-1.37978500	1.35774700	-2.07727900
F	-5.91367600	0.66746100	-1.28593900
F	-5.95572600	-0.76458500	1.07340300

^{3}Cat-1

C	5.40578100	-1.33566100	-0.53400200
C	6.64395900	-0.45254100	-0.64983400
C	6.63660600	0.64966600	0.40067600
C	5.37477500	1.50105000	0.30012100
C	4.13794700	0.62579400	0.42099500
C	4.14324800	-0.49481700	-0.63638300
N	2.85644200	1.31681900	0.28041800
N	2.88325400	-1.21823000	-0.48172600
C	2.68137600	2.59249800	0.34566300
C	2.74430200	-2.49552800	-0.56082800
C	1.43614400	3.28278100	0.17346000
C	1.51685400	-3.21672300	-0.38063600
C	1.45414200	4.68540200	0.06333700
C	0.29029500	5.38679500	-0.16439600
C	-0.91368600	4.68769700	-0.31887800
C	-0.98594600	3.30449600	-0.21859800
C	0.19764100	2.58276800	0.09744100
C	0.27087600	-2.54490200	-0.25175600

C	-0.88986500	-3.28893000	0.08341000
C	-0.78901300	-4.67316400	0.16093300
C	0.42109300	-5.34611700	-0.04064600
C	1.56482800	-4.61876600	-0.29306800
O	0.10510500	1.31181800	0.36007800
O	0.14172400	-1.26455100	-0.47982500
C	-2.23648100	2.56271100	-0.47481300
C	-2.14720700	-2.57460500	0.38333600
C	-2.24228500	1.41869800	-1.28711800
C	-3.40981600	0.73025900	-1.57593700
C	-4.60003500	1.18390800	-1.02813600
C	-4.65452300	2.30252000	-0.21425300
C	-3.46691900	2.97023700	0.04072600
C	-3.38073400	-2.99610800	-0.11316700
C	-4.57444000	-2.35339800	0.17600000
C	-4.52109300	-1.24582600	1.00525800
C	-3.32801300	-0.78122600	1.53686300
C	-2.15386600	-1.44439500	1.21560300
Mn	1.37199300	0.04814000	-0.11477900
Cl	1.52280400	-0.65364200	2.12384400
O	1.46339000	0.63611000	-1.73831100
H	5.42193700	-2.10097700	-1.32245900
H	5.40696100	-1.86240100	0.43556800
H	5.38091900	2.26620700	1.08877700
H	5.35308700	2.02810000	-0.66950200
H	4.11707700	0.13889800	1.41166900
H	4.10376100	-0.00572100	-1.62650000
H	3.55638700	3.23075900	0.52118400
H	3.63326900	-3.11213900	-0.74564000
H	2.40852300	5.20693300	0.14687600
H	0.30546200	6.47021900	-0.25438700
H	-1.82551300	5.23888600	-0.54159700
H	-1.68292600	-5.24429300	0.40476000
H	0.46003900	-6.43014200	0.03394200
H	2.52661900	-5.11968300	-0.41040200
H	-3.40892400	-0.16321600	-2.19551500
H	-5.59098400	2.64043900	0.21956100
H	-5.51368800	-2.70224900	-0.24308600
H	-3.32869600	0.10035700	2.17320900
H	7.54899800	-1.06718900	-0.55840400
H	6.67410200	0.00030500	-1.65470700
H	7.52432000	1.28737900	0.29793500
H	6.68986800	0.19842900	1.40492700
F	-5.72953300	0.52179200	-1.28524700

F	-5.65617900	-0.60331000	1.29066500
H	-1.20807000	-1.08214000	1.61740000
F	-3.43281000	-4.06822900	-0.91635200
F	-3.52111500	4.05430800	0.82591400
H	-1.29856700	1.06602600	-1.69890800

^{3}Cat-2

C	5.28784000	-1.61498300	-0.41292400
C	6.58934600	-0.82050500	-0.46724100
C	6.61393300	0.27749000	0.58778800
C	5.42004300	1.21498000	0.43651100
C	4.12411100	0.42423700	0.50406800
C	4.09188600	-0.68784000	-0.56172200
N	2.89728700	1.19848500	0.32856800
N	2.77940700	-1.32341100	-0.46044000
C	2.80009500	2.48226500	0.38428500
C	2.55398100	-2.58397300	-0.58716000
C	1.60049300	3.24585900	0.19608300
C	1.27620600	-3.22463600	-0.45930000
C	1.70478900	4.64654300	0.10494700
C	0.58848500	5.42737300	-0.09895300
C	-0.66132100	4.80814300	-0.23557900
C	-0.81958900	3.43314200	-0.14703600
C	0.31941000	2.62513800	0.12035800
C	0.07690200	-2.47564100	-0.31696500
C	-1.13354900	-3.15090700	-0.01288900
C	-1.12799400	-4.54106800	0.00560300
C	0.03464400	-5.28650300	-0.21889800
C	1.22834300	-4.62921000	-0.42859100
O	0.13863900	1.36107000	0.35517200
O	0.03311800	-1.18309500	-0.49880400
C	-2.14652700	2.80360900	-0.30028800
C	-2.33674200	-2.36911000	0.33905000
C	-2.36155900	1.77025900	-1.21889700
C	-3.59709300	1.17607800	-1.40344300
C	-4.66036700	1.64585100	-0.65052200
C	-4.51507000	2.67066700	0.27105700
C	-3.25493100	3.23602800	0.43390800
C	-3.60702300	-2.70483800	-0.12905700
C	-4.75903200	-2.03226500	0.25020300
C	-4.62374500	-0.98513700	1.14618600
C	-3.38811700	-0.59518400	1.63889000
C	-2.25779600	-1.28380500	1.22601400
Mn	1.34250200	0.03429400	-0.11706200

Cl	1.42009700	-0.69802700	2.12816700
O	1.56909000	0.65270900	-1.70634300
H	5.28559300	-2.37480900	-1.20669400
H	5.20758900	-2.14629300	0.55081000
H	5.44487300	1.97764300	1.22727600
H	5.47608000	1.74233800	-0.53157900
H	4.03468100	-0.06733300	1.48845400
H	4.12784500	-0.19288500	-1.54941300
H	3.70951400	3.06496800	0.57851400
H	3.40182200	-3.25637000	-0.77040500
H	2.69132000	5.10472700	0.18759400
H	0.67502100	6.50819700	-0.17941400
H	-1.54301700	5.41617600	-0.44134100
H	-2.06079600	-5.05917200	0.22075700
H	-0.00214900	-6.37280800	-0.19104300
H	2.15534200	-5.18959000	-0.55610700
H	-3.72487500	0.36909100	-2.11966800
H	-5.37407600	2.99666600	0.85149500
H	-5.72973800	-2.32064200	-0.14225000
H	-3.32444200	0.23940200	2.33233400
H	7.44299000	-1.49886300	-0.33893300
H	6.69659400	-0.36870100	-1.46733200
H	7.54849200	0.85039000	0.52690400
H	6.59000700	-0.17850900	1.59100600
H	-3.11293200	4.02961800	1.16713600
F	-1.35753300	1.36440400	-1.99053700
F	-5.85878500	1.08614700	-0.82506100
F	-5.71783000	-0.33086300	1.54270000
H	-1.28292800	-0.98788200	1.61133400
F	-3.73929000	-3.72630800	-0.98729500

^{3}Cat-3

C	-5.39267600	1.14367900	-0.72955500
C	-6.57823600	0.20318800	-0.92220800
C	-6.58676600	-0.89567400	0.13199700
C	-5.28201900	-1.68635000	0.11892500
C	-4.09791800	-0.75314300	0.31495900
C	-4.08962100	0.36095100	-0.74908400
N	-2.77985300	-1.38504800	0.25972100
N	-2.87459600	1.14102800	-0.53058400
C	-2.55013400	-2.64756100	0.38349600
C	-2.78948100	2.42303300	-0.59229300
C	-1.26845400	-3.28326200	0.28970700
C	-1.59867200	3.19526900	-0.37604300

C	-1.21785900	-4.68762000	0.22010400
C	-0.01733400	-5.33744600	0.03179000
C	1.15074500	-4.58427200	-0.14278700
C	1.15343300	-3.19636400	-0.08507900
C	-0.06301500	-2.52731300	0.22220200
C	-0.32327300	2.58111500	-0.23593700
C	0.80931600	3.39571200	0.03065900
C	0.64444100	4.77304100	0.08342500
C	-0.60162800	5.38513900	-0.09809400
C	-1.70948300	4.59620000	-0.31922400
O	-0.03186400	-1.25175000	0.47362700
O	-0.13248800	1.30394500	-0.41193200
C	2.35262000	-2.39992900	-0.41385600
C	2.14139500	2.78201400	0.20961400
C	2.26071600	-1.29490700	-1.27434700
C	3.38152100	-0.58769700	-1.68028100
C	4.62375700	-0.98074800	-1.20560700
C	4.77224800	-2.04834400	-0.33640000
C	3.62814200	-2.73804000	0.03639300
C	3.24763800	3.21492800	-0.52810300
C	4.51375200	2.66659500	-0.35361300
C	4.66694600	1.65870000	0.58523900
C	3.60798700	1.19324000	1.34587700
C	2.36443500	1.76884800	1.14957300
Mn	-1.33196500	-0.05104600	-0.06277900
Cl	-1.68168100	0.65343900	2.13256500
O	-1.30896400	-0.65612100	-1.69283300
H	-5.39229400	1.90795300	-1.51925000
H	-5.48064600	1.66884600	0.23690500
H	-5.30515900	-2.44823200	0.91029500
H	-5.17070500	-2.21500000	-0.84362200
H	-4.16374800	-0.26202500	1.30138900
H	-3.96938600	-0.13128800	-1.73114500
H	-3.40265000	-3.32114400	0.53658200
H	-3.69631100	3.00240200	-0.80909400
H	-2.14759700	-5.25242200	0.29984400
H	0.02257900	-6.42256600	-0.02406500
H	2.08857700	-5.09586100	-0.35206500
H	1.52025600	5.38741500	0.29494200
H	-0.69320200	6.46711300	-0.04233600
H	-2.69442500	5.04882000	-0.44157300
H	3.30648100	0.26640000	-2.34872000
H	5.74796600	-2.34059700	0.04037100
H	5.37047000	2.99226100	-0.93766700

H	-7.51548300	0.77419300	-0.89298700
H	-6.51969100	-0.25230800	-1.92463200
H	-7.43430200	-1.57558400	-0.02529000
H	-6.72634300	-0.44524400	1.12832000
F	5.71063800	-0.31327000	-1.59820600
F	3.77330100	-3.78134200	0.86457100
H	1.27951700	-1.00090200	-1.64302900
H	3.09931000	3.99535000	-1.27434300
F	1.36564300	1.36258600	1.91952200
F	5.86972500	1.10950100	0.76663800
H	3.74323200	0.40447800	2.08075500

^{3}Cat-4

C	5.29903100	-1.41068600	-0.63019100
C	6.54684700	-0.54620300	-0.78567000
C	6.59615200	0.55116200	0.26899600
C	5.34412600	1.42108400	0.22262200
C	4.10161800	0.56095300	0.38752200
C	4.04904800	-0.54758100	-0.68168100
N	2.82604800	1.27059600	0.30978100
N	2.77968500	-1.24853700	-0.50177800
C	2.66426400	2.54376100	0.42527600
C	2.60921700	-2.51954800	-0.59606400
C	1.41872500	3.24851100	0.32524500
C	1.36325700	-3.21419200	-0.42769600
C	1.44563200	4.65509400	0.28333000
C	0.28467500	5.38176000	0.13360400
C	-0.93050200	4.69987200	-0.01822400
C	-1.01103200	3.31611600	0.01912800
C	0.16999800	2.56354100	0.26330700
C	0.12985200	-2.51823600	-0.29273300
C	-1.05951100	-3.26072800	-0.06603100
C	-0.99135400	-4.64694300	-0.05810800
C	0.21271600	-5.33811500	-0.24014500
C	1.37655600	-4.62041700	-0.41272600
O	0.05588100	1.28918600	0.47832900
O	0.02613600	-1.22798100	-0.43202700
C	-2.29354200	2.62284800	-0.21503900
C	-2.34818100	-2.56943900	0.14712900
C	-2.40838200	1.64419300	-1.20887200
C	-3.60951300	1.03725300	-1.52909300
C	-4.74119600	1.43495800	-0.83673000
C	-4.69185800	2.38571800	0.17023100
C	-3.46381900	2.96954300	0.46609100

C	-3.49201100	-2.91054600	-0.58132600
C	-4.73082500	-2.32848500	-0.33137600
C	-4.81915000	-1.38420300	0.67928700
C	-3.71574100	-0.99301000	1.41773700
C	-2.50100900	-1.59744800	1.14295400
Mn	1.30529300	0.03568100	-0.06196000
Cl	1.59430100	-0.70941800	2.14307100
O	1.41990900	0.68839600	-1.65619200
H	5.27317600	-2.17058600	-1.42367200
H	5.32558600	-1.94439000	0.33517500
H	5.39324000	2.18048500	1.01543900
H	5.29031800	1.95598500	-0.74146800
H	4.11631100	0.06274700	1.37219100
H	3.98795500	-0.04606100	-1.66502000
H	3.55081700	3.16833000	0.59293000
H	3.47945300	-3.15511800	-0.80484300
H	2.40843300	5.16255000	0.35852300
H	0.31135100	6.46793900	0.09402300
H	-1.84473400	5.26482500	-0.20383700
H	-1.91065400	-5.20516100	0.12206500
H	0.22862100	-6.42501900	-0.21888400
H	2.33107200	-5.13528200	-0.53009600
H	-3.66300900	0.28468300	-2.31068000
H	-5.60120000	2.65319700	0.70187500
H	-5.61865900	-2.59251300	-0.89990500
H	-3.79732000	-0.24607400	2.20242900
H	7.44486700	-1.17554500	-0.73077300
H	6.54678800	-0.08872500	-1.78893700
H	7.48909400	1.17544600	0.13371600
H	6.68170000	0.09413000	1.26840300
H	-3.39847900	-3.65410900	-1.37267100
H	-3.40047600	3.71828600	1.25530700
F	-1.46539900	-1.27116300	1.90125900
F	-1.33896700	1.31992700	-1.93021300
F	-5.91073300	0.88008800	-1.16084700
F	-6.00043100	-0.83070400	0.96025200

^{5}Cat-1

C	5.39814000	-1.41429700	-0.42890900
C	6.64645300	-0.55242100	-0.59145100
C	6.64897000	0.60888500	0.39202900
C	5.39836800	1.46821700	0.23459100
C	4.15072400	0.61696100	0.40788000
C	4.14433200	-0.56636900	-0.57555300

N	2.87370000	1.30885100	0.23214600
N	2.87578700	-1.26799500	-0.38346300
C	2.72442400	2.59236200	0.22316900
C	2.71883500	-2.53676200	-0.50664800
C	1.48864800	3.29215300	0.06143100
C	1.47070100	-3.23937400	-0.37071700
C	1.51707500	4.68994900	-0.10964400
C	0.35524200	5.39286300	-0.33282600
C	-0.86023800	4.69839400	-0.42468800
C	-0.94305200	3.32381000	-0.26043300
C	0.24230000	2.60097100	0.05250800
C	0.22942800	-2.54828900	-0.23568900
C	-0.96414500	-3.29108200	0.00946200
C	-0.89672000	-4.67864200	-0.01209700
C	0.30776400	-5.35586900	-0.22499500
C	1.48040300	-4.63934800	-0.38546500
O	0.14734600	1.34382000	0.35690900
O	0.13348400	-1.26157800	-0.34280600
C	-2.20360000	2.58320100	-0.46809100
C	-2.20011600	-2.56558600	0.34856500
C	-2.23503800	1.43041300	-1.26762100
C	-3.41764300	0.75763400	-1.53386100
C	-4.59297300	1.23115400	-0.97068300
C	-4.61856900	2.35123400	-0.15792800
C	-3.41819100	3.00654000	0.07056000
C	-3.44493700	-2.93598700	-0.16499800
C	-4.62198900	-2.28210500	0.15842100
C	-4.54181500	-1.21956600	1.04382500
C	-3.33888300	-0.81169600	1.60030600
C	-2.18000700	-1.48266900	1.24411200
Mn	1.39957800	0.03551200	-0.02882800
Cl	1.55272100	-0.59538000	2.22991500
O	1.44875600	0.43200000	-1.74475300
H	5.40866700	-2.22052100	-1.17541100
H	5.39142200	-1.88911700	0.56703400
H	5.41247200	2.28174000	0.97303400
H	5.38595200	1.93303400	-0.76660600
H	4.12520800	0.19302100	1.42754400
H	4.09734800	-0.13582100	-1.59247000
H	3.61715000	3.22168200	0.32244000
H	3.59298700	-3.16547100	-0.71836900
H	2.47904800	5.20326800	-0.07712500
H	0.37694600	6.47117000	-0.47067000
H	-1.76925800	5.25115100	-0.65632700

H	-1.80707200	-5.24752600	0.16418900
H	0.32435700	-6.44311700	-0.23069500
H	2.43098600	-5.15830700	-0.51246500
H	-3.44028200	-0.13337500	-2.15664300
H	-5.54317100	2.70388400	0.28948900
H	-5.56913200	-2.58418000	-0.27874400
H	-3.32036700	0.03049200	2.28718000
H	7.54422500	-1.17074900	-0.46125400
H	6.68390700	-0.15951500	-1.62100300
H	7.54555300	1.22754500	0.25537300
H	6.69214700	0.21726100	1.42145300
F	-5.73788100	0.58766400	-1.21270400
F	-5.66031000	-0.56771100	1.36198100
H	-1.22957900	-1.17087800	1.67640700
F	-3.51849700	-3.96208800	-1.02363600
F	-3.44562200	4.09433500	0.85111800
H	-1.30088100	1.06018900	-1.68799500

^{5}Cat-2

C	5.29109500	-1.65574700	-0.30926600
C	6.59363900	-0.86918200	-0.42450400
C	6.62922500	0.29226700	0.55863400
C	5.43713300	1.22262800	0.35642200
C	4.13862500	0.44347700	0.48776100
C	4.09538100	-0.73554200	-0.50086000
N	2.90844600	1.20628800	0.28386100
N	2.78263400	-1.36324400	-0.35772400
C	2.82651900	2.49562600	0.25681000
C	2.54697600	-2.61322600	-0.53423700
C	1.62764500	3.25539200	0.08558300
C	1.25351200	-3.23700700	-0.44978600
C	1.72893200	4.65246800	-0.06962300
C	0.60669900	5.42613000	-0.25529800
C	-0.64883800	4.80162500	-0.31691300
C	-0.80362500	3.43331700	-0.16761600
C	0.34447800	2.63176100	0.09001100
C	0.05796600	-2.47315100	-0.31313200
C	-1.17897000	-3.14237900	-0.08396900
C	-1.19821300	-4.53157100	-0.13943900
C	-0.03934900	-5.27925800	-0.36783100
C	1.17693100	-4.63480600	-0.50044800
O	0.17608600	1.38011700	0.37424200
O	0.04298400	-1.17847600	-0.40104100
C	-2.13382300	2.80126900	-0.27636100

C	-2.36586300	-2.35500000	0.29736200
C	-2.36596200	1.76043500	-1.18213600
C	-3.61063800	1.18140800	-1.35452700
C	-4.66236800	1.66937400	-0.59695000
C	-4.49747000	2.69527700	0.31964100
C	-3.22993500	3.24995900	0.46564900
C	-3.64332900	-2.65362700	-0.18043200
C	-4.78344400	-1.98073600	0.22871300
C	-4.62992800	-0.97054700	1.16442400
C	-3.38774000	-0.61711700	1.66842700
C	-2.26887200	-1.30750400	1.22990400
Mn	1.37381000	0.01889400	-0.02234300
Cl	1.44335400	-0.65727000	2.22746400
O	1.56320300	0.47166500	-1.72072400
H	5.28114200	-2.46141300	-1.05624400
H	5.21762400	-2.12934200	0.68458300
H	5.47183200	2.03587100	1.09462900
H	5.48707900	1.68505500	-0.64473300
H	4.06046500	0.01736600	1.50390100
H	4.11366100	-0.30045200	-1.51686200
H	3.74994500	3.07837900	0.36163400
H	3.38409600	-3.29013900	-0.74722100
H	2.71903800	5.11002300	-0.04943200
H	0.68866200	6.50282200	-0.38245900
H	-1.53755400	5.40178200	-0.51604600
H	-2.14344200	-5.04635000	0.02096200
H	-0.09329200	-6.36477100	-0.40286600
H	2.09492600	-5.20875300	-0.63042400
H	-3.75398000	0.37235800	-2.06541000
H	-5.34800500	3.03421800	0.90515700
H	-5.75917400	-2.23602200	-0.17406700
H	-3.30972100	0.19217400	2.38992100
H	7.44765200	-1.53968700	-0.26196500
H	6.69142900	-0.48043300	-1.45168000
H	7.56530800	0.85638800	0.45430900
H	6.61183200	-0.09912500	1.58888700
H	-3.07337100	4.05051800	1.18821800
F	-1.37053800	1.33342200	-1.95132300
F	-5.86910200	1.12172100	-0.75951300
F	-5.71246700	-0.31631300	1.58670100
H	-1.29198300	-1.04867800	1.63642500
F	-3.79128200	-3.63376100	-1.08213200

^{5}Cat-3

C	5.39659200	-1.17721400	-0.66111500
C	6.58044900	-0.24390100	-0.89601400
C	6.58807200	0.89993000	0.10827100
C	5.28366800	1.68961700	0.05786400
C	4.09987200	0.76660600	0.29939200
C	4.09208800	-0.39747500	-0.70704500
N	2.77821100	1.38973900	0.22348400
N	2.87867000	-1.17011100	-0.45243700
C	2.55791000	2.66114900	0.29962500
C	2.78120100	-2.44599100	-0.56435300
C	1.28010600	3.29621900	0.22545300
C	1.57473200	-3.20745200	-0.38348100
C	1.22561400	4.69991800	0.12135300
C	0.02129200	5.34209300	-0.05675600
C	-1.14821300	4.58018300	-0.19436300
C	-1.14706200	3.19549400	-0.10358100
C	0.07557000	2.53486700	0.20439100
C	0.30414900	-2.57783400	-0.24080600
C	-0.85132600	-3.38339100	-0.03026000
C	-0.71146400	-4.76392100	-0.03615200
C	0.52768100	-5.38517100	-0.23016300
C	1.65683100	-4.60824600	-0.39468000
O	0.05464600	1.26829200	0.48275800
O	0.14117800	-1.29311200	-0.33014300
C	-2.34446000	2.39009400	-0.41629800
C	-2.16802500	-2.75354200	0.18496000
C	-2.24948500	1.27516200	-1.26392200
C	-3.37101100	0.56737100	-1.66830700
C	-4.61433400	0.96794600	-1.20330700
C	-4.76483500	2.04347500	-0.34467100
C	-3.62093500	2.73505500	0.02526100
C	-3.29568800	-3.16424100	-0.53423500
C	-4.54980400	-2.60424500	-0.32352100
C	-4.67068400	-1.60993800	0.63510500
C	-3.59104800	-1.16929000	1.38080300
C	-2.35809000	-1.75426800	1.14854700
Mn	1.35687100	0.03844700	0.01625200
Cl	1.71183400	-0.61281700	2.21695000
O	1.30808100	0.50025500	-1.70668700
H	5.39669100	-1.97199800	-1.41995400
H	5.48687500	-1.66414300	0.32489600
H	5.30764100	2.48811200	0.81214800
H	5.17197300	2.17235900	-0.92854100
H	4.16877900	0.32367000	1.30879600

H	3.95854900	0.04966700	-1.70899600
H	3.41849700	3.33490500	0.39290500
H	3.67715800	-3.02945900	-0.81235700
H	2.15626800	5.26710600	0.16677500
H	-0.02382900	6.42545100	-0.13755800
H	-2.08994000	5.08428400	-0.40479700
H	-1.59752800	-5.37373300	0.14111000
H	0.60058400	-6.47006100	-0.22600700
H	2.63367400	-5.07555500	-0.52438500
H	-3.29557100	-0.29207600	-2.32985400
H	-5.74153400	2.33980700	0.02633000
H	-5.42257100	-2.90627300	-0.89627100
H	7.51872800	-0.81150700	-0.84299600
H	6.51985300	0.16716400	-1.91735600
H	7.43671900	1.57119200	-0.07759300
H	6.72547600	0.49340700	1.12360400
F	-5.70177000	0.29606600	-1.59033800
F	-3.76791000	3.78800800	0.84099400
H	-1.26655800	0.97322100	-1.62351100
H	-3.17233300	-3.93243800	-1.29746900
F	-1.34057000	-1.37335100	1.90675000
F	-5.86250700	-1.04984500	0.84644400
H	-3.70127800	-0.38951000	2.12924800

^{5}Cat-4

C	5.33016400	-1.41380700	-0.49783300
C	6.57089800	-0.54871000	-0.69972900
C	6.60089600	0.61458100	0.28129600
C	5.34170900	1.46838200	0.16830000
C	4.10528500	0.61010500	0.38257200
C	4.07225500	-0.56702500	-0.60868700
N	2.82071600	1.29879300	0.26186800
N	2.80929600	-1.27203700	-0.40020600
C	2.65497200	2.57988500	0.29942600
C	2.63542800	-2.53497600	-0.55369800
C	1.40531400	3.26780500	0.21103500
C	1.37591000	-3.22103500	-0.44147100
C	1.41803900	4.67437700	0.12050700
C	0.24760200	5.38543300	-0.00843300
C	-0.96590000	4.68530500	-0.09492200
C	-1.03366400	3.30435500	-0.01149800
C	0.16254200	2.56543500	0.21452600
C	0.14466800	-2.51432700	-0.32546700
C	-1.06245600	-3.24866100	-0.15814100

C	-1.01159500	-4.63509300	-0.20370900
C	0.18905800	-5.33108300	-0.38504900
C	1.36988700	-4.62379100	-0.48901600
O	0.07287000	1.29888700	0.46122400
O	0.06456100	-1.21947600	-0.39717000
C	-2.32097500	2.60430800	-0.19557400
C	-2.34159900	-2.55388400	0.08886000
C	-2.46284000	1.61918700	-1.17916600
C	-3.67675700	1.02481000	-1.47534700
C	-4.79127100	1.43698200	-0.76422200
C	-4.71367100	2.39214200	0.23677700
C	-3.47465400	2.96634400	0.50522600
C	-3.50077600	-2.88089000	-0.62323800
C	-4.73587400	-2.31364200	-0.32885100
C	-4.80645900	-1.40050000	0.71180800
C	-3.68897900	-1.02482900	1.43663800
C	-2.47662500	-1.61178300	1.11679700
Mn	1.34439900	0.02275000	0.01105500
Cl	1.60980300	-0.65189900	2.22112400
O	1.47701800	0.53977000	-1.69165200
H	5.31876700	-2.22039800	-1.24388600
H	5.35355600	-1.88825800	0.49797900
H	5.37936700	2.27941300	0.90872200
H	5.29031000	1.93680900	-0.82991400
H	4.11831300	0.17923200	1.39956800
H	4.00340000	-0.12678700	-1.62019200
H	3.54301900	3.21753100	0.39007800
H	3.50125800	-3.16848000	-0.78503000
H	2.37882400	5.19065300	0.14397300
H	0.26027400	6.46997400	-0.08328800
H	-1.89163900	5.23561000	-0.26735200
H	-1.93993300	-5.18946900	-0.06411300
H	0.19136000	-6.41805300	-0.41251600
H	2.31960000	-5.14852500	-0.59952600
H	-3.75177100	0.26997100	-2.25319300
H	-5.61017300	2.67374400	0.78274600
H	-5.63451500	-2.56413200	-0.88636700
H	-3.75596400	-0.30345100	2.24607700
H	7.47454900	-1.16377400	-0.59691000
H	6.57451400	-0.15770900	-1.73071300
H	7.48956900	1.23695800	0.11298700
H	6.68119600	0.22380800	1.30882400
H	-3.42215100	-3.60035700	-1.43798700
H	-3.38998600	3.72260900	1.28526800

F	-1.42900900	-1.30331300	1.86616600
F	-1.40968800	1.27630800	-1.91190400
F	-5.97229100	0.89117800	-1.06318700
F	-5.98314800	-0.86045300	1.03227400

$^{oss}TS_{R}$

C	-5.59726700	-1.56471300	1.47952000
C	-6.81348000	-0.77279100	1.94975100
C	-7.30654200	0.18037300	0.86954100
C	-6.20180300	1.13447700	0.42188900
C	-4.99187900	0.34672700	-0.05519800
C	-4.49607500	-0.61636900	1.03674400
N	-3.83446500	1.13953300	-0.48084100
N	-3.27623200	-1.23785600	0.52650200
C	-3.89211100	2.35727700	-0.88957100
C	-2.94713900	-2.46798700	0.69516000
C	-2.78694200	3.13200000	-1.38084000
C	-1.74306200	-3.08164900	0.20240200
C	-3.01359200	4.48560600	-1.69080500
C	-1.99869600	5.28667400	-2.16813800
C	-0.71513500	4.74741800	-2.32311100
C	-0.43720100	3.42088000	-2.03283200
C	-1.49490500	2.56905300	-1.59623200
C	-0.72605900	-2.33274000	-0.45794300
C	0.35136500	-3.03088500	-1.07367200
C	0.45493800	-4.40437600	-0.88478300
C	-0.49434700	-5.12749000	-0.15430400
C	-1.59380000	-4.47091900	0.35769700
O	-1.22567500	1.30890000	-1.44743700
O	-0.69718600	-1.03243700	-0.48813400
C	0.92712500	2.86168800	-2.11332700
C	1.27571600	-2.28928500	-1.95707200
C	1.46766100	2.13355600	-1.04352400
C	2.74759800	1.60187500	-1.09361900
C	3.49658000	1.77737300	-2.24552700
C	3.02001900	2.49470400	-3.32975500
C	1.74217400	3.02746500	-3.23303100
C	2.65028300	-2.51816800	-1.96884000
C	3.52367200	-1.89614700	-2.84832300
C	2.98357200	-1.00777000	-3.76303700
C	1.62607400	-0.72670300	-3.79627300
C	0.78890400	-1.35832500	-2.89018400
F	1.27541600	3.71574400	-4.28191500
F	4.71957200	1.24039000	-2.30400100

F	3.79659300	-0.40827300	-4.63607000
F	3.17858800	-3.36781800	-1.06634900
Mn	-2.16923600	0.10731000	-0.41867300
Cl	-3.01672200	-0.90206700	-2.39793400
O	-1.78409100	0.78244400	1.05676900
H	-5.23600600	-2.20923500	2.29319000
H	-5.87297100	-2.21830100	0.63407400
H	-6.58065400	1.78412000	-0.37930000
H	-5.90266300	1.78874900	1.25903700
H	-5.26921200	-0.27019300	-0.92783200
H	-4.18620300	0.00128000	1.90043200
H	-4.85915700	2.87544600	-0.86897700
H	-3.63183700	-3.13450000	1.23436200
H	-4.01397200	4.89491100	-1.54370000
H	-2.18361000	6.33185600	-2.40447500
H	0.09575400	5.38723500	-2.66821100
H	1.28882400	-4.93251900	-1.34390500
H	-0.38343900	-6.20204500	-0.03028800
H	-2.37285400	-5.02429300	0.88359800
H	0.86232200	1.99996300	-0.14452500
H	3.17875400	1.05869400	-0.25494900
H	3.61076100	2.61609500	-4.23301900
H	4.59073900	-2.09784300	-2.81663000
H	1.23863000	-0.02105700	-4.52681300
H	-0.27864200	-1.14294400	-2.91148200
H	-6.54212000	-0.19561600	2.84933100
H	-7.61401700	-1.46098700	2.25129000
H	-7.65230400	-0.40129600	-0.00084200
H	-8.17249100	0.75398000	1.22554800
C	0.97580000	3.65302800	3.12887400
C	2.33125000	3.65189700	2.78952900
C	2.96380300	2.47884900	2.41504100
C	2.25574400	1.27367500	2.37401100
C	0.88874500	1.26868200	2.71368200
C	0.26465300	2.47039400	3.08129200
N	2.88026700	0.07762200	1.93070900
C	1.95259200	-0.86206800	1.29396300
C	0.85193300	-1.21759400	2.27601200
C	0.07886500	0.04036900	2.60517700
C	4.15286800	-0.24287200	2.43244200
O	4.64691300	0.44291900	3.30726100
C	4.95528700	-1.40093000	1.80824300
C	4.41880000	-2.77759800	2.23367800
C	6.37943800	-1.29728500	2.36024100

C	5.03411300	-1.24170100	0.28702100
H	-0.69780600	0.30779300	1.66240200
N	-0.84383700	-0.09208300	3.72315000
N	-1.71129200	-0.95412300	3.61251900
N	-2.56066700	-1.71697100	3.62930500
H	0.48147900	4.57852700	3.41676600
H	2.89890300	4.58043300	2.80545500
H	4.01598600	2.48472400	2.14727000
H	-0.79645000	2.44589000	3.32356300
H	2.48076800	-1.75149600	0.95650000
H	1.51502100	-0.39721900	0.39622300
H	1.29600400	-1.62122100	3.20234000
H	0.18863300	-1.99203100	1.86309100
H	4.29767700	-2.82165200	3.32543800
H	5.15261900	-3.54571100	1.95272200
H	3.46894600	-3.06942400	1.77379800
H	6.99950200	-2.07957300	1.90130900
H	6.82700300	-0.32213700	2.13683200
H	6.39971600	-1.42712300	3.44803700
H	5.53054000	-0.29715600	0.02193200
H	4.06458600	-1.25285800	-0.22140500
H	5.62887300	-2.06378600	-0.13702300

^{css}TS$_R$

C	-5.66648600	-1.58385200	1.37062700
C	-6.86514200	-0.80137200	1.90103100
C	-7.30602000	0.27934100	0.92249900
C	-6.15806000	1.22318100	0.57490200
C	-4.98107900	0.43105200	0.02459900
C	-4.52863900	-0.63388100	1.03444200
N	-3.79858400	1.21320700	-0.35959700
N	-3.31166300	-1.24183700	0.50127800
C	-3.86722600	2.39725200	-0.85183800
C	-2.97094600	-2.46896800	0.65089500
C	-2.76068900	3.14140500	-1.38953000
C	-1.74491000	-3.05423200	0.17284700
C	-2.99542800	4.46610400	-1.79637500
C	-1.98231300	5.24050700	-2.32141700
C	-0.69731100	4.70025600	-2.43399800
C	-0.41272500	3.39632700	-2.05253700
C	-1.46437200	2.57546500	-1.54890100
C	-0.71942200	-2.29066200	-0.46760500
C	0.37012100	-2.99064600	-1.07083200

C	0.48415100	-4.36239000	-0.87705600
C	-0.46937000	-5.09111800	-0.15916300
C	-1.58372200	-4.44143700	0.32997300
O	-1.17002400	1.33569300	-1.27479300
O	-0.68573500	-0.99490800	-0.50145800
C	0.95608000	2.84668500	-2.12048200
C	1.29604000	-2.25362100	-1.95538300
C	1.51731400	2.15211500	-1.03905400
C	2.79868100	1.62409900	-1.09013100
C	3.53448900	1.77664600	-2.25397800
C	3.04303600	2.47054600	-3.34663900
C	1.76128800	2.99246100	-3.25116100
C	2.67023200	-2.48733000	-1.96918400
C	3.54405600	-1.87620500	-2.85530000
C	3.00516200	-0.99547300	-3.77761400
C	1.64812200	-0.71147400	-3.81168500
C	0.81058900	-1.32927700	-2.89638200
F	1.28125500	3.64888900	-4.31490400
F	4.75780700	1.24157300	-2.31879400
F	3.81989900	-0.40643200	-4.65622800
F	3.20105700	-3.33115900	-1.06144400
Mn	-2.16325800	0.14593400	-0.32030200
Cl	-3.02776300	-0.76550800	-2.33530200
O	-1.82705700	0.63349500	1.21289800
H	-5.34312700	-2.31630900	2.12366000
H	-5.94572600	-2.14589800	0.46293800
H	-6.50771700	1.96443200	-0.15659400
H	-5.83356900	1.77942900	1.47100800
H	-5.29167300	-0.10516000	-0.88957300
H	-4.22224300	-0.10245800	1.95528500
H	-4.83935300	2.90556100	-0.88696200
H	-3.65768400	-3.15891700	1.15827400
H	-4.00002400	4.87623000	-1.68471800
H	-2.17290200	6.26539700	-2.63097500
H	0.11042600	5.31643000	-2.82503200
H	1.32624100	-4.88625700	-1.32553100
H	-0.35235100	-6.16447100	-0.03004600
H	-2.36697600	-5.00161800	0.84247600
H	0.92884900	2.03976600	-0.12748400
H	3.23533700	1.09905300	-0.24237600
H	3.62376100	2.57847200	-4.25791300
H	4.61049700	-2.08120100	-2.82333300
H	1.26123000	-0.01472600	-4.55091200
H	-0.25618000	-1.10869100	-2.91547500

H	-6.59124700	-0.33354200	2.86127400
H	-7.69434500	-1.48838300	2.11567700
H	-7.67229200	-0.19426600	-0.00326100
H	-8.14830700	0.85047600	1.33500400
C	0.90301900	3.59189100	3.19737800
C	2.25093700	3.62449500	2.83032300
C	2.90211400	2.46815600	2.43420800
C	2.22087400	1.24898900	2.39589700
C	0.85830500	1.20838400	2.76219300
C	0.21659700	2.39469800	3.15647600
N	2.86281900	0.06812300	1.93981800
C	1.94574500	-0.87042800	1.28837700
C	0.86030900	-1.26960100	2.27167200
C	0.09376200	-0.03588400	2.67182800
C	4.14333300	-0.23833300	2.42844300
O	4.63901600	0.45680900	3.29513800
C	4.95355400	-1.39028300	1.79983700
C	4.40584500	-2.77810800	2.17462900
C	6.36575100	-1.29941800	2.38281500
C	5.05663400	-1.20091800	0.28361400
H	-0.81034200	0.27518400	1.67335500
N	-0.84575000	-0.19550000	3.74932400
N	-1.67402700	-1.09787500	3.63217100
N	-2.49150800	-1.89419200	3.63908900
H	0.39431300	4.50373400	3.50318900
H	2.79771300	4.56541300	2.84346300
H	3.94906600	2.49839500	2.14822500
H	-0.83787300	2.34580700	3.42287400
H	2.48656900	-1.74160000	0.92672000
H	1.49490100	-0.38657900	0.40745900
H	1.32129300	-1.71231200	3.17316600
H	0.19489900	-2.03455900	1.84209000
H	4.17594800	-2.83058500	3.24853900
H	5.18011400	-3.53030000	1.96950700
H	3.51797400	-3.08882800	1.61337500
H	6.99706100	-2.06661400	1.91404100
H	6.81505000	-0.31695800	2.19966800
H	6.36313100	-1.46255200	3.46664000
H	5.55206600	-0.24992800	0.04072300
H	4.09176200	-1.21060300	-0.23403500
H	5.66007800	-2.01352400	-0.14656100

^{3}TS$_{R}$

C	-5.73574900	-1.39818900	1.68538400

C	-6.99396700	-0.61479500	2.04667900
C	-7.52772900	0.15832800	0.84845000
C	-6.47056500	1.10134300	0.28170500
C	-5.21726000	0.32398300	-0.08693900
C	-4.67802000	-0.46376000	1.12058600
N	-4.10169400	1.12037100	-0.59900200
N	-3.43912800	-1.09914300	0.67469300
C	-4.19055700	2.32420500	-1.04770000
C	-3.06827000	-2.29023400	0.98413100
C	-3.11503400	3.12036500	-1.56394600
C	-1.86809800	-2.93470700	0.52186000
C	-3.37536800	4.46834000	-1.87547500
C	-2.38140800	5.28663300	-2.36484200
C	-1.08967000	4.76897000	-2.52683100
C	-0.77914000	3.45027200	-2.22957100
C	-1.81530500	2.58098100	-1.78638700
C	-0.87899200	-2.22863900	-0.21509800
C	0.19382000	-2.94642200	-0.80004300
C	0.31457400	-4.30490600	-0.52443100
C	-0.61155700	-4.98762800	0.27009100
C	-1.70328100	-4.30769800	0.76996800
O	-1.54889000	1.31574100	-1.66196800
O	-0.88101200	-0.92365900	-0.31389900
C	0.59923000	2.92755200	-2.33075800
C	1.11641500	-2.24634800	-1.71709700
C	1.18741500	2.24500800	-1.25759300
C	2.48826500	1.76629700	-1.31587300
C	3.21238700	1.95814800	-2.48160000
C	2.68323000	2.62148400	-3.57523100
C	1.38509000	3.10017200	-3.46977800
C	2.49731500	-2.41649800	-1.64995100
C	3.38442900	-1.81820500	-2.53169800
C	2.85013700	-1.01549400	-3.52586400
C	1.48668200	-0.78770300	-3.63353000
C	0.63345900	-1.39926800	-2.72792800
F	0.87252400	3.74629800	-4.52398300
F	4.45739000	1.47840000	-2.55514100
F	3.67991400	-0.43727900	-4.39610900
F	3.01143500	-3.17704300	-0.66458900
Mn	-2.37531200	0.14741800	-0.48430000
Cl	-3.14686200	-1.14204900	-2.30920600
O	-2.02456400	1.13721800	0.89539900
H	-5.35097100	-1.91205000	2.57715600
H	-5.97361000	-2.16937200	0.93262900

H	-6.87330300	1.61982400	-0.59955800
H	-6.21311100	1.87269600	1.02801100
H	-5.45190200	-0.41267900	-0.87543600
H	-4.38848000	0.26885100	1.89552600
H	-5.17220000	2.81389600	-1.03154900
H	-3.72023600	-2.90123300	1.62079400
H	-4.38193900	4.85777500	-1.71689900
H	-2.58676800	6.32721300	-2.60426600
H	-0.29401900	5.42133200	-2.88438600
H	1.14945600	-4.85047500	-0.96137500
H	-0.48752000	-6.05114500	0.45948700
H	-2.46380500	-4.83119600	1.35036300
H	0.59965800	2.10468700	-0.34950000
H	2.95531900	1.25717100	-0.47340600
H	3.25599400	2.75234400	-4.48870800
H	4.45680200	-1.96680000	-2.44089000
H	1.11029400	-0.12496400	-4.40927200
H	-0.44035700	-1.23513700	-2.81196800
H	-6.75889900	0.08905000	2.86205500
H	-7.75855500	-1.29933500	2.43688400
H	-7.83571900	-0.55121800	0.06323700
H	-8.42467000	0.72862200	1.12397900
C	0.70016200	4.27466700	2.49723200
C	2.06119800	4.24262500	2.18465000
C	2.71777600	3.03609900	2.01711400
C	2.02813700	1.82783200	2.16193500
C	0.65899400	1.85351500	2.47875300
C	0.00931300	3.08649700	2.63483200
N	2.68052600	0.58841400	1.91644600
C	1.78076800	-0.45744000	1.42103200
C	0.66024000	-0.67531500	2.42234200
C	-0.14000700	0.60725700	2.54686200
C	3.95159800	0.38335000	2.47666300
O	4.40955800	1.20135500	3.25202400
C	4.80138700	-0.82309200	2.03508200
C	4.31896600	-2.12991500	2.68226500
C	6.22163400	-0.57489800	2.55117200
C	4.87131800	-0.91019200	0.50786000
H	-0.88208400	0.69980900	1.61041800
N	-1.03619100	0.62822800	3.70479000
N	-1.94794100	-0.19126700	3.69301000
N	-2.83418700	-0.90455600	3.79513500
H	0.18644700	5.22589400	2.62017400
H	2.61354500	5.17181400	2.05683300

H	3.77411200	3.01657500	1.76654100
H	-1.05564400	3.08264200	2.86077600
H	2.32921400	-1.38148300	1.24292800
H	1.35499500	-0.14998800	0.45254400
H	1.08566700	-0.93296000	3.40677900
H	0.01228300	-1.50686600	2.10599200
H	5.07256300	-2.91162600	2.51352900
H	3.37004100	-2.51634500	2.29415900
H	4.21372600	-2.00187800	3.76896400
H	6.87248000	-1.39606800	2.22039900
H	6.62840300	0.36793600	2.16738900
H	6.24988600	-0.52362800	3.64519700
H	5.33772800	-0.00556400	0.09099700
H	3.90084600	-1.03013300	0.01790300
H	5.48889100	-1.77213300	0.21535300

^{5}TS$_{R}$

C	-5.57755200	-1.58597800	1.46303800
C	-6.78426100	-0.78684100	1.94422000
C	-7.29002300	0.15700300	0.86257500
C	-6.18850600	1.10469900	0.39580300
C	-4.98522700	0.31187100	-0.08971600
C	-4.47645900	-0.64535400	0.99984800
N	-3.83354400	1.10103500	-0.53225300
N	-3.27723500	-1.28470100	0.46332200
C	-3.88368000	2.34854900	-0.86549100
C	-2.93850100	-2.50603000	0.67745300
C	-2.79762800	3.14196600	-1.34652300
C	-1.75044700	-3.14159100	0.17583200
C	-3.00755500	4.52189700	-1.54436800
C	-1.98842100	5.33585300	-1.98180400
C	-0.71680800	4.78281400	-2.19786100
C	-0.45574200	3.43581700	-2.01023700
C	-1.52316600	2.57088400	-1.63388400
C	-0.73152800	-2.40303500	-0.48585400
C	0.35142500	-3.09857900	-1.08626300
C	0.44615300	-4.47320500	-0.89508300
C	-0.51005300	-5.19078600	-0.16931600
C	-1.60743700	-4.52918700	0.34228400
O	-1.30503300	1.29526000	-1.60833400
O	-0.72086800	-1.09729300	-0.51507500
C	0.90239600	2.87077400	-2.14543400
C	1.29659500	-2.35814000	-1.94618800
C	1.46494500	2.10988600	-1.11144200
C	2.74663000	1.58585200	-1.20385100

C	3.47376700	1.81132100	-2.36210100
C	2.97021400	2.55787700	-3.41352500
C	1.69174400	3.07887800	-3.27545900
C	2.67204300	-2.57893900	-1.90899600
C	3.57166500	-1.94264700	-2.74987200
C	3.05870500	-1.04833600	-3.67440000
C	1.70132800	-0.77836400	-3.75897100
C	0.83618500	-1.42752000	-2.89270300
F	1.20064100	3.80372400	-4.28774300
F	4.69766800	1.28617800	-2.46965100
F	3.90248900	-0.41943800	-4.49397400
F	3.17241800	-3.42746200	-0.99118300
Mn	-2.18413700	0.02452900	-0.57813700
Cl	-2.98483000	-1.03561000	-2.51523800
O	-1.79615900	0.87072600	0.95698400
H	-5.21516400	-2.22995700	2.27564200
H	-5.86647500	-2.24147500	0.62336900
H	-6.57275000	1.75022100	-0.40624800
H	-5.87793300	1.76238100	1.22598200
H	-5.27418700	-0.30928600	-0.95680400
H	-4.13795600	-0.02656500	1.85067700
H	-4.84230200	2.87236900	-0.76470200
H	-3.60386000	-3.14521800	1.27033500
H	-3.99585600	4.93550700	-1.33901700
H	-2.15385000	6.39941600	-2.13562000
H	0.10350000	5.42983200	-2.50631400
H	1.28244100	-5.00477300	-1.34570900
H	-0.40382900	-6.26557500	-0.04363900
H	-2.38782800	-5.07701200	0.87155300
H	0.87647700	1.94825800	-0.20563400
H	3.19825800	1.02108200	-0.38930900
H	3.54592500	2.71680600	-4.32077800
H	4.63932800	-2.12862700	-2.67498900
H	1.33571200	-0.06474200	-4.49302400
H	-0.23186500	-1.22535600	-2.95611800
H	-6.49800100	-0.20330700	2.83488400
H	-7.58121600	-1.47142600	2.26291200
H	-7.64751000	-0.43100800	0.00128600
H	-8.15005400	0.73606400	1.22383900
C	0.97389400	3.71036900	3.05194800
C	2.33058100	3.70626200	2.71871800
C	2.96536200	2.52854200	2.36308200
C	2.25716600	1.32301400	2.33823400
C	0.89083600	1.31990700	2.67493300

C	0.26436500	2.52568200	3.01948900
N	2.88258300	0.12019600	1.90982000
C	1.95220200	-0.82047300	1.28024800
C	0.85446500	-1.17223900	2.26787900
C	0.07102500	0.08895800	2.57581200
C	4.14418800	-0.20411300	2.43385600
O	4.64078200	0.49991300	3.29280900
C	4.93505100	-1.39606600	1.85895100
C	4.35966000	-2.74920200	2.30761900
C	6.34868500	-1.30725100	2.43955000
C	5.05037500	-1.28108200	0.33619200
H	-0.66575800	0.30885100	1.67216100
N	-0.81345100	-0.04567300	3.73945900
N	-1.75928500	-0.81248000	3.60397500
N	-2.67538300	-1.49565900	3.59877200
H	0.47671700	4.63966100	3.32249200
H	2.89713300	4.63566700	2.72365700
H	4.01811800	2.53099100	2.09765100
H	-0.79829000	2.50339300	3.25490700
H	2.47755400	-1.71133900	0.94092200
H	1.51362800	-0.35070200	0.38477800
H	1.29935100	-1.56536100	3.19725300
H	0.19037600	-1.94730100	1.85878300
H	5.08618300	-3.53911400	2.07170900
H	3.41797700	-3.03587400	1.82788000
H	4.20482300	-2.75838500	3.39594500
H	6.96144500	-2.11537100	2.01695200
H	6.82209300	-0.34836900	2.19976500
H	6.34133500	-1.40642800	3.53077000
H	5.57274100	-0.35413600	0.05942900
H	4.09175500	-1.28719700	-0.19266800
H	5.63807300	-2.12600600	-0.05172900

^{oss}**TS$_S$**

C	5.97197500	-0.04532000	0.89672000
C	6.92256800	-1.23253300	1.01753900
C	6.95989400	-2.04461100	-0.26997100
C	5.56711000	-2.53204900	-0.65837400
C	4.61587100	-1.35380400	-0.78991000
C	4.58492100	-0.52379800	0.50552500
N	3.22395300	-1.68615400	-1.08869300
N	3.57755000	0.52013200	0.31683600
C	2.79691200	-2.83143000	-1.48111900
C	3.65547800	1.71674900	0.78131300

C	1.41946000	-3.16597400	-1.73343900
C	2.67706400	2.75334600	0.58573400
C	1.09991500	-4.51770300	-1.94260200
C	-0.20632800	-4.91341100	-2.13951300
C	-1.21886400	-3.94924900	-2.13547900
C	-0.95580600	-2.59297100	-1.96835800
C	0.39456900	-2.17590900	-1.77936000
C	1.42108000	2.50176900	-0.03729600
C	0.58245100	3.60061200	-0.37744600
C	0.95697700	4.87392900	0.03948100
C	2.14143100	5.10886100	0.74564200
C	3.00129600	4.05850200	0.99352500
O	0.64338700	-0.90172300	-1.68712500
O	0.98012100	1.29576400	-0.26257700
C	-2.04808300	-1.59707900	-1.93000600
C	-0.62384900	3.36368000	-1.19749100
C	-2.05502500	-0.53642100	-1.00976600
C	-3.10817400	0.35924500	-0.91489000
C	-4.18355000	0.21399900	-1.77610800
C	-4.22683200	-0.79656900	-2.72170400
C	-3.15774900	-1.67758400	-2.77726400
C	-1.83475500	4.02265700	-0.97681200
C	-2.96086200	3.83325700	-1.76396800
C	-2.86034800	2.95553100	-2.82958400
C	-1.68345200	2.28050500	-3.11278600
C	-0.58325100	2.48709700	-2.29584300
F	-3.21070800	-2.64128500	-3.70710500
F	-5.19403700	1.08228900	-1.70965300
F	-3.93361300	2.74863600	-3.59640200
F	-1.94572000	4.89319400	0.04151800
Mn	1.99682700	-0.19616100	-0.64477800
Cl	2.90178200	0.88972100	-2.52716300
O	1.59655200	-1.07098000	0.73954900
H	5.93212200	0.49407200	1.85379800
H	6.33897800	0.66235100	0.13351400
H	5.61668800	-3.09156000	-1.60329500
H	5.18513700	-3.22400600	0.11259600
H	4.95304100	-0.68723200	-1.60213500
H	4.19601800	-1.19012300	1.29552900
H	3.51882200	-3.64593300	-1.62486400
H	4.55143700	2.00990500	1.34458700
H	1.90396200	-5.25485900	-1.92729400
H	-0.45286700	-5.96255900	-2.28454100
H	-2.25251700	-4.26548200	-2.26246800

H	0.31170500	5.71475500	-0.20784200
H	2.39893700	6.11913700	1.05484000
H	3.95672300	4.23126800	1.49074200
H	-1.19227500	-0.39322100	-0.36338900
H	-3.08019800	1.18482200	-0.20574300
H	-5.06005600	-0.89373600	-3.41119600
H	-3.88740200	4.35613900	-1.54567300
H	-1.64532800	1.58743900	-3.95009700
H	0.34498100	1.96026200	-2.51347200
C	-1.55848400	3.13432900	2.40838300
C	-2.86986500	2.66141100	2.32105700
C	-3.14284900	1.30572900	2.37846900
C	-2.10728900	0.36807000	2.51128200
C	-0.77589600	0.84500400	2.58233400
C	-0.52890200	2.22663500	2.52711500
N	-2.37523900	-1.02165100	2.49608500
C	-1.23138000	-1.91125900	2.29155700
C	-0.06063600	-1.47670400	3.15279000
C	0.34891000	-0.09442900	2.70165600
C	-3.62773600	-1.51685000	2.90309800
O	-4.34864400	-0.85290100	3.62154900
C	-4.12586000	-2.88556800	2.38384700
C	-3.62533000	-3.26389400	0.98709000
C	-3.80863000	-3.97508300	3.41545400
C	-5.65356700	-2.76337300	2.29639000
H	0.81738000	-0.34997900	1.58580900
N	1.46217100	0.49599700	3.42194000
N	2.50263100	-0.15122100	3.49855800
N	3.52707300	-0.63169900	3.65267000
H	-1.34894100	4.19931000	2.35667600
H	-3.69293500	3.36351600	2.19658900
H	-4.16978500	0.96447300	2.31531400
H	0.50261100	2.57274200	2.58247800
H	-1.51896800	-2.92730400	2.56249500
H	-0.93008000	-1.91813200	1.23014900
H	-0.34535400	-1.45908200	4.21658000
H	0.77014300	-2.18351100	3.02446700
H	-4.23223700	-4.09710200	0.60668200
H	-3.73594200	-2.42283300	0.28854600
H	-2.58131800	-3.59339000	0.95032200
H	-4.29216000	-4.91483000	3.11419200
H	-4.19864000	-3.69343900	4.40256500
H	-2.73484800	-4.18084800	3.52090600
H	-6.09157600	-2.49336900	3.26167300

H	-5.94604400	-1.99615800	1.56686800
H	-6.07519500	-3.72324800	1.96903000
H	7.92856900	-0.88018100	1.28075200
H	6.58826400	-1.87528800	1.84930900
H	7.63919000	-2.90099800	-0.16639700
H	7.36448700	-1.42080700	-1.08387000

^{css}TS$_{S}$

C	6.03967600	-0.06153600	0.79835900
C	6.99493600	-1.24424100	0.93384200
C	6.95713600	-2.13465700	-0.30092300
C	5.54311200	-2.63424100	-0.58591900
C	4.59621800	-1.45474900	-0.73611100
C	4.63278700	-0.55649800	0.51080600
N	3.18867400	-1.77806300	-0.98320900
N	3.61430000	0.47578100	0.32331900
C	2.76470900	-2.90267600	-1.42977100
C	3.66870100	1.67020400	0.79010800
C	1.38770500	-3.20739800	-1.72541700
C	2.64947400	2.67548000	0.62760100
C	1.07168100	-4.53799200	-2.04156100
C	-0.22697700	-4.91099300	-2.31734900
C	-1.23351300	-3.94251700	-2.30070900
C	-0.97305300	-2.59973800	-2.04023500
C	0.36722800	-2.21108600	-1.73669700
C	1.38307400	2.41027300	0.01521800
C	0.54086800	3.51713100	-0.31521600
C	0.90582200	4.79043500	0.10844600
C	2.08921600	5.02777900	0.81378100
C	2.95981200	3.98079000	1.04311800
O	0.58383300	-0.95235700	-1.47639100
O	0.93633500	1.21808700	-0.23047800
C	-2.06960100	-1.60637000	-2.01701000
C	-0.65284900	3.28867400	-1.15419000
C	-2.14623400	-0.58176100	-1.05823100
C	-3.21668200	0.29690500	-0.99518800
C	-4.23747900	0.17475600	-1.92303200
C	-4.20945900	-0.79639300	-2.90871000
C	-3.12729700	-1.66162400	-2.93307600
C	-1.86093700	3.96303500	-0.96255600
C	-2.96854600	3.79363900	-1.78019300
C	-2.84992700	2.92625500	-2.85192200
C	-1.67430500	2.23844100	-3.10915800

C	-0.59509600	2.42082800	-2.25974300
F	-3.11574200	-2.58255300	-3.90694400
F	-5.26274200	1.02616000	-1.88214900
F	-3.90454700	2.73924700	-3.64943400
F	-1.98895700	4.83290800	0.05436700
Mn	2.00288300	-0.29191000	-0.52675100
Cl	2.88498400	0.73730500	-2.45166200
O	1.69952200	-0.97157300	0.93740800
H	6.05017100	0.52801900	1.72635800
H	6.36347000	0.60298800	-0.02085400
H	5.54568200	-3.24649400	-1.49875000
H	5.19599700	-3.27972000	0.23973700
H	4.91260200	-0.83490100	-1.59264200
H	4.29427800	-1.17265500	1.36360800
H	3.48069000	-3.71577000	-1.60753200
H	4.57337400	1.99376700	1.32318800
H	1.87268300	-5.27835900	-2.04962900
H	-0.47179400	-5.94710700	-2.53926200
H	-2.25922400	-4.24760900	-2.49422600
H	0.25550400	5.62874800	-0.13368300
H	2.34176100	6.03671500	1.13154200
H	3.91835300	4.15979900	1.53240100
H	-1.32784800	-0.44897600	-0.35389000
H	-3.24411900	1.09639700	-0.25753800
H	-4.99716700	-0.87600900	-3.65180800
H	-3.89223900	4.32964200	-1.58247900
H	-1.62103400	1.55770100	-3.95560200
H	0.33099000	1.88193300	-2.45560600
C	-1.52908000	3.20880100	2.45437100
C	-2.82778500	2.73282800	2.27160300
C	-3.09370800	1.37330700	2.28325100
C	-2.06530800	0.44051300	2.46647600
C	-0.74048000	0.91918900	2.62274700
C	-0.49974300	2.30530300	2.60997600
N	-2.33093000	-0.95330800	2.43067100
C	-1.17362300	-1.82969300	2.25271600
C	-0.04693100	-1.41408700	3.18131400
C	0.36552500	-0.01668300	2.79584900
C	-3.58380600	-1.44831200	2.83515500
O	-4.32754600	-0.76324100	3.50964800
C	-4.05669700	-2.84616900	2.37054700
C	-3.61166600	-3.22617400	0.95572800
C	-3.65368100	-3.90881900	3.40199100
C	-5.58991500	-2.78686400	2.35888500

H	0.94141500	-0.33492100	1.61864500
N	1.47424500	0.55859500	3.50674700
N	2.52609300	-0.07356200	3.57775600
N	3.56200400	-0.52971900	3.72865200
H	-1.32344700	4.27602500	2.44447200
H	-3.64462600	3.43407800	2.10820400
H	-4.11335700	1.02662500	2.15690700
H	0.52426300	2.65737700	2.72838200
H	-1.46387300	-2.85605200	2.47244300
H	-0.82397100	-1.79929900	1.20593200
H	-0.38490300	-1.43551700	4.23050300
H	0.79529700	-2.11184200	3.07717700
H	-4.14385000	-4.13783600	0.65025300
H	-3.86737600	-2.43364300	0.23833000
H	-2.54094300	-3.43335100	0.85342400
H	-4.14853000	-4.85907200	3.15701800
H	-3.97878500	-3.60933900	4.40741300
H	-2.57462400	-4.10722500	3.44000800
H	-5.99118100	-2.53866900	3.34604100
H	-5.95220000	-2.02987800	1.65123100
H	-5.98639800	-3.76308500	2.04857900
H	8.01520100	-0.88171600	1.11626800
H	6.70946300	-1.83516100	1.82033200
H	7.63802200	-2.98774800	-0.18117800
H	7.31892400	-1.56551900	-1.17312100

^{3}TS$_{S}$

C	5.93387700	-0.10208600	0.91971300
C	6.87991900	-1.29511300	1.00914200
C	6.96278000	-2.03189300	-0.32091900
C	5.58497000	-2.50363100	-0.77556500
C	4.62876800	-1.32559000	-0.87333800
C	4.56082000	-0.55778300	0.45916200
N	3.24514000	-1.66008000	-1.20646700
N	3.56533900	0.49902000	0.28527000
C	2.82704400	-2.80757800	-1.61102200
C	3.65930800	1.69640600	0.74813500
C	1.45614700	-3.16450800	-1.85449300
C	2.70165200	2.74986500	0.54727000
C	1.14730100	-4.52261600	-2.03748000
C	-0.16093500	-4.93088500	-2.18914700
C	-1.18325800	-3.97681700	-2.15424400
C	-0.93141800	-2.61598600	-2.00458200

C	0.42014600	-2.18539700	-1.88434800
C	1.44364400	2.50711100	-0.06965100
C	0.60904900	3.60419100	-0.41472700
C	0.99656000	4.87730100	-0.00929900
C	2.18856500	5.10921100	0.68635700
C	3.04135400	4.05536400	0.94203300
O	0.68286700	-0.91133800	-1.85524900
O	0.99829100	1.29529500	-0.27001700
C	-2.03410700	-1.63454500	-1.91715900
C	-0.61230800	3.35937100	-1.20937600
C	-2.01732900	-0.58095200	-0.98964100
C	-3.08760900	0.28654900	-0.83179100
C	-4.20868100	0.11080200	-1.62707100
C	-4.27149800	-0.88694600	-2.58567100
C	-3.18082900	-1.73348300	-2.71121400
C	-1.82321600	4.00784000	-0.96166000
C	-2.96846600	3.79578100	-1.71483500
C	-2.88637900	2.90666800	-2.77281400
C	-1.71008000	2.24300000	-3.08259200
C	-0.59041700	2.47284000	-2.29943300
F	-3.25457300	-2.68887100	-3.64813000
F	-5.25734100	0.91898000	-1.46504200
F	-3.97663300	2.67791300	-3.50894000
F	-1.91182600	4.89190300	0.04575400
Mn	1.99645700	-0.17704600	-0.75903000
Cl	2.90166000	0.98024300	-2.59883100
O	1.50102900	-1.13610000	0.60600000
H	5.85735900	0.38425300	1.90243700
H	6.32783900	0.64267000	0.20690100
H	5.66233900	-3.01355100	-1.74639100
H	5.18615600	-3.23472100	-0.05067100
H	4.97402400	-0.62026900	-1.64867000
H	4.14344600	-1.25834800	1.20310000
H	3.56084700	-3.61035200	-1.75812900
H	4.55667100	1.97244500	1.31774200
H	1.95820700	-5.25219700	-2.03360900
H	-0.40289600	-5.98365300	-2.31386700
H	-2.21730400	-4.30615800	-2.23930700
H	0.35420100	5.72053800	-0.25623700
H	2.45502100	6.12045200	0.98443800
H	3.99990100	4.22417700	1.43431100
H	-1.13272100	-0.43093200	-0.37379600
H	-3.04690700	1.10478700	-0.11459900
H	-5.14167100	-1.00655200	-3.22408800

H	-3.89322600	4.31409600	-1.47883100
H	-1.68746600	1.54279000	-3.91439400
H	0.33953200	1.96018300	-2.54127700
C	-1.50402000	3.16611300	2.43536000
C	-2.82208800	2.70931400	2.38841000
C	-3.10819400	1.35635700	2.44912900
C	-2.07922800	0.40600800	2.54746500
C	-0.74387100	0.86746600	2.58258100
C	-0.48262000	2.24376700	2.52253500
N	-2.36262100	-0.98234500	2.52887900
C	-1.23145500	-1.88099500	2.29230100
C	-0.03792900	-1.46126800	3.12866600
C	0.38293100	-0.08797800	2.65912000
C	-3.60945100	-1.46752800	2.95963400
O	-4.31097900	-0.80118700	3.69565500
C	-4.13156500	-2.82856800	2.44183500
C	-3.69652500	-3.17403900	1.01494200
C	-3.76828200	-3.94196100	3.43252900
C	-5.66178800	-2.70974200	2.43009200
H	0.79370600	-0.30436900	1.56659400
N	1.50524900	0.48842200	3.39567700
N	2.54411900	-0.16158100	3.43114000
N	3.56980000	-0.65242000	3.54444100
H	-1.28183100	4.22857100	2.38065100
H	-3.64066500	3.42101200	2.29164100
H	-4.14036800	1.02692900	2.41688100
H	0.55502600	2.57594400	2.54751700
H	-1.52300400	-2.89646800	2.56022900
H	-0.94864300	-1.87926800	1.22693700
H	-0.29982400	-1.43338000	4.19755100
H	0.78072700	-2.17907400	2.98437100
H	-4.28608400	-4.03230500	0.66345900
H	-3.88680600	-2.33387100	0.33238100
H	-2.64156300	-3.45098300	0.91504500
H	-4.29470700	-4.86471900	3.15122300
H	-4.08279900	-3.66986100	4.44893300
H	-2.69595400	-4.17653200	3.46008600
H	-6.05554100	-2.47641900	3.42379900
H	-5.99233000	-1.91851100	1.74424800
H	-6.09513100	-3.65989800	2.08954300
H	7.87513400	-0.95894300	1.32825200
H	6.51582600	-1.98352100	1.79027300
H	7.64332000	-2.89026200	-0.24659500
H	7.38712100	-1.36027800	-1.08516900

^{5}TS$_{S}$

C	5.88830300	-0.07481500	0.95646800
C	6.83245600	-1.26528800	1.08747600
C	6.92453700	-2.04058800	-0.21961500
C	5.55023500	-2.52330300	-0.67331100
C	4.59682100	-1.34711000	-0.81147900
C	4.51838300	-0.54392800	0.49907500
N	3.21717300	-1.68340400	-1.16154200
N	3.52436800	0.50705600	0.29255200
C	2.81026700	-2.83954100	-1.56228600
C	3.58917100	1.69525400	0.78225300
C	1.45119100	-3.19913100	-1.84434400
C	2.62030000	2.73676100	0.57740700
C	1.14217300	-4.55648500	-2.04080800
C	-0.16330800	-4.95954600	-2.22108700
C	-1.18586000	-4.00303600	-2.19473400
C	-0.93294400	-2.64575500	-2.02903300
C	0.41808000	-2.21877600	-1.88989700
C	1.37200000	2.48582900	-0.06157400
C	0.52318400	3.58170000	-0.38910500
C	0.89122500	4.85193800	0.03857000
C	2.07193900	5.08630200	0.75295300
C	2.93438600	4.03844000	1.00261600
O	0.67988800	-0.94746900	-1.84853600
O	0.95621000	1.27797300	-0.31481400
C	-2.02483400	-1.65364300	-1.94120700
C	-0.68831200	3.33856500	-1.19717900
C	-2.00222400	-0.61715100	-0.99398400
C	-3.05354200	0.27399100	-0.84753500
C	-4.15725400	0.14295200	-1.67530300
C	-4.23078000	-0.84642300	-2.64110800
C	-3.16006900	-1.72084900	-2.75285000
C	-1.90808900	3.96729800	-0.93942200
C	-3.04814600	3.75721600	-1.69951800
C	-2.95169100	2.89441700	-2.77813700
C	-1.76515500	2.25562400	-3.10199700
C	-0.65093500	2.47934300	-2.30872900
F	-3.23909300	-2.66703900	-3.69838200
F	-5.16603300	1.00816400	-1.55669800
F	-4.03886200	2.66772700	-3.51849500
F	-2.01198500	4.82159900	0.09189900
Mn	1.98948400	-0.18232500	-0.77591400
Cl	2.89642500	0.97643800	-2.60416000

O	1.51422600	-1.15312000	0.65103800
H	5.80640600	0.43855700	1.92466100
H	6.28633800	0.65071800	0.22624600
H	5.63725000	-3.05823500	-1.62971300
H	5.14351200	-3.23518700	0.06607900
H	4.95439700	-0.66162200	-1.59974800
H	4.09273800	-1.22406700	1.25759200
H	3.54813200	-3.64398700	-1.67275800
H	4.46611100	1.97166000	1.38239100
H	1.95109800	-5.28813200	-2.02436600
H	-0.40537600	-6.01060600	-2.35934200
H	-2.21941000	-4.32927900	-2.29780400
H	0.24146000	5.69116200	-0.20225800
H	2.32255200	6.09550400	1.07107300
H	3.88285400	4.21105400	1.51256900
H	-1.12744100	-0.50117300	-0.35624000
H	-3.00431200	1.08627700	-0.12441300
H	-5.08742600	-0.93076300	-3.30306200
H	-3.98310400	4.25088700	-1.45141100
H	-1.73072600	1.57598800	-3.95029800
H	0.28602800	1.98337500	-2.55809500
C	-1.42902700	3.17764900	2.48545400
C	-2.74728100	2.72833000	2.37140300
C	-3.04341500	1.37848100	2.41311300
C	-2.02477400	0.42008500	2.55699600
C	-0.68716200	0.87532100	2.65555600
C	-0.41814500	2.25008100	2.61510900
N	-2.31411100	-0.96416600	2.52698700
C	-1.18367700	-1.88558800	2.38259800
C	-0.02400600	-1.44890400	3.25595000
C	0.42550000	-0.09093400	2.76657700
C	-3.59993400	-1.43864800	2.84692400
O	-4.34762500	-0.77209800	3.53549400
C	-4.10217700	-2.79130100	2.28781300
C	-3.51989500	-3.21513700	0.93715100
C	-3.90293300	-3.87805400	3.35146300
C	-5.61241000	-2.61009100	2.07778600
H	0.83625400	-0.31509700	1.69490600
N	1.54753700	0.47536200	3.52152200
N	2.58653300	-0.17433800	3.52986600
N	3.60994100	-0.67601600	3.62120200
H	-1.19877400	4.23943800	2.45238100
H	-3.55450400	3.44587400	2.23224400
H	-4.07481200	1.05587700	2.33115900

H	0.61844700	2.57831200	2.68807800
H	-1.50506200	-2.88404700	2.68279600
H	-0.84720900	-1.93494400	1.33380200
H	-0.32424600	-1.39634000	4.31362900
H	0.79275100	-2.17686200	3.15727900
H	-4.08952100	-4.08018100	0.56914300
H	-3.61884200	-2.41242800	0.19352900
H	-2.46829600	-3.51931600	0.96182000
H	-4.40321700	-4.80196900	3.02932200
H	-4.34537800	-3.56619900	4.30677200
H	-2.84714400	-4.12440800	3.52960400
H	-6.11591600	-2.31738800	3.00357500
H	-5.81061900	-1.83482200	1.32415100
H	-6.04463900	-3.55323500	1.71728800
H	7.82574100	-0.92097100	1.40393700
H	6.46101700	-1.92937000	1.88609500
H	7.60309200	-2.89731200	-0.11463800
H	7.35670700	-1.39235500	-0.99960600
